# Resource Selection by the California Condor (*Gymnogyps californianus*) Relative to Terrestrial-Based Habitats and Meteorological Conditions

**DOI:** 10.1371/journal.pone.0088430

**Published:** 2014-02-11

**Authors:** James W. Rivers, J. Matthew Johnson, Susan M. Haig, Carl J. Schwarz, John W. Glendening, L. Joseph Burnett, Daniel George, Jesse Grantham

**Affiliations:** 1 Department of Forest Ecosystems and Society, Oregon State University, Corvallis, Oregon, United States of America; 2 U.S. Geological Survey Forest and Rangeland Ecosystem Science Center, Corvallis, Oregon, United States of America; 3 Department of Statistics and Actuarial Science, Simon Fraser University, Burnaby, British Columbia, Canada; 4 Salinas, California, United States of America; 5 Ventana Wildlife Society, Salinas, California, United States of America; 6 Pinnacles National Park, National Park Service, Paicines, California, United States of America; 7 California Condor Recovery Program, U.S. Fish and Wildlife Service, Ventura, California, United States of America; Universidade de São Paulo, Faculdade de Filosofia Ciências e Letras de Ribeirão Preto, Brazil

## Abstract

Condors and vultures are distinct from most other terrestrial birds because they use extensive soaring flight for their daily movements. Therefore, assessing resource selection by these avian scavengers requires quantifying the availability of terrestrial-based habitats, as well as meteorological variables that influence atmospheric conditions necessary for soaring. In this study, we undertook the first quantitative assessment of habitat- and meteorological-based resource selection in the endangered California condor (*Gymnogyps californianus*) within its California range and across the annual cycle. We found that condor use of terrestrial areas did not change markedly within the annual cycle, and that condor use was greatest for habitats where food resources and potential predators could be detected and where terrain was amenable for taking off from the ground in flight (e.g., sparse habitats, coastal areas). Condors originating from different release sites differed in their use of habitat, but this was likely due in part to variation in habitats surrounding release sites. Meteorological conditions were linked to condor use of ecological subregions, with thermal height, thermal velocity, and wind speed having both positive (selection) and negative (avoidance) effects on condor use in different areas. We found little evidence of systematic effects between individual characteristics (i.e., sex, age, breeding status) or components of the species management program (i.e., release site, rearing method) relative to meteorological conditions. Our findings indicate that habitat type and meteorological conditions can interact in complex ways to influence condor resource selection across landscapes, which is noteworthy given the extent of anthropogenic stressors that may impact condor populations (e.g., lead poisoning, wind energy development). Additional studies will be valuable to assess small-scale condor movements in light of these stressors to help minimize their risk to this critically endangered species.

## Introduction

All animals require resources critical to their survival, and determining how and why an organism selects among available resources is fundamental to understanding its ecological niche. Resource selection is considered to occur across sequential spatial scales, with the broadest level of selection being that of a geographic range (i.e., first-order selection), followed by selection of individual home ranges (i.e., second-order selection), selection of coarse-scale habitats within the home range (i.e., third-order selection), and selection of microhabitats within coarse-scale habitats (i.e., fourth-order selection; [9], [24], [33]).Traditionally, studies of vertebrate resource selection have focused on quantifying use of terrestrial resources because most terrestrial-based species have their needs met by resources that are located on or near ground level. Nevertheless, a number of animals have resource requirements that extend beyond terrestrial habitats, especially for organisms that use extensive, long-ranging flight [14].

Animals that fly above the earth's surface do so within the convective “boundary layer” of the atmosphere, and meteorological conditions that occur within this stratum can strongly influence space use and movements for some taxa. Animal flight can be facilitated by two types of vertical air movement within the boundary layer: thermal lift and orographic lift [5], [6], [32], [42], [44], [57]. Thermal lift occurs when solar radiation heats the earth's surface and creates convective thermals of vertically rising air within the boundary layer [18]. Warm, rising air provides lift used by large birds to move vertically via soaring flight within convective thermals, and soaring in thermals is typically combined with descending flights between thermals that allow individuals to move across landscapes through the use of so-called “thermal streets” [15], [20], [42], [52], [59]. Thermals vary in their strength, vertical height, and horizontal spacing [65], all of which may influence their suitability for flying animals [42], [52]. In contrast to thermal lift, orographic lift occurs when horizontal surface winds meet pronounced features on the landscape, causing wind currents to rise vertically and generate lift [18]. Horizontal wind speeds also vary in their suitability for flight, such that greater wind speeds provide stronger updrafts along sloping topography; however, such winds can produce turbulence that inhibits flight at high speeds. Although animals with soaring flight use thermal lift and orographic lift to move across large areas, meteorological conditions vary across temporal and spatial scales [65] and, in turn, influence the degree to which specific areas are used by flying animals [15], [51].

The California condor (*Gymnogyps californianus*, hereafter condor) is the largest landbird in North America and also one of the most critically endangered [56]. Because of their large size, condors are unable to use flapping flight during long-distance movements and instead rely on buoyant, vertically-moving air currents to facilitate energetically inexpensive soaring [20], [40], [50], [56]. Condors feed almost exclusively on carrion and therefore move over vast areas to locate this ephemeral and patchily-distributed resource [38], [48]. Currently, we know very little about variation in condor use of terrestrial-based habitats as they move across the landscape and the extent to which meteorological conditions influence their use of space [38], [56]. This presents a serious issue for conservation efforts for this critically endangered species because condors are recolonizing their historic range in California, including locations where condors are exposed to spent lead ammunition in animal carrion, the biggest threat to their recovery [17], [47].

Wind energy developments can pose serious hazards for wildlife [28], [45], especially for large flying animals that may collide with wind turbines during flight [2], [21], [31], [34], [55]. This issue is of particular concern to condors for two reasons. First, areas of high wind availability serve as ideal locations for siting wind turbines yet are especially attractive to birds that use soaring flight [6], [30], [58]. Second, condors exemplify the slow end of slow-fast life history continuum and have experienced a long and steady population decline [56], [62] such that the world's population of condors currently consists of approximately 400 individuals, approximately half of which are free-flying individuals [61]. Thus, any mortality events, including those that may occur at wind turbines, serve as substantial losses to the population.

In this study, we provide the first quantitative analysis of resource selection of the California condor throughout the annual cycle and across its range in California. We used an extensive, long-term dataset that comprises high-resolution GPS location data to [1] quantify condor selection of terrestrial-based habitats as demarcated by coarse-scale landcover type (e.g., grassland, coniferous forest) [2], assess the relationship between condor use and three meteorological conditions that influence flight conditions for soaring birds (i.e., thermal height, thermal velocity, and wind speed), and [3] evaluate how condor selection of these resources varies across the annual cycle and relative to individual characteristics (i.e., age, sex, breeding status) and factors related to recovery program actions (i.e., rearing method, release location). Specifically, we predicted that condors would select terrestrial habitats thought to be important for foraging and movement, such as open grasslands, and avoid habitats that pose challenges to flight and foraging (e.g., conifer forest). In addition, we predicted that thermal characteristics would have stronger selection by condors as previous work with vulture movements has shown thermals are important for the long-distance movements often made by condors [6], [32], [38], [42], [43]. Finally, we predicted no differences in resource selection of condors relative to sex, age, breeding status, or rearing method. However, because our previous work found strong differences in monthly home range size of condors originating from different release sites [48], we predicted resource selection patterns would differ relative to release site. Our investigation is useful for conservation of this species because it provides data regarding areas that condors are likely to occupy that may assist in delineating areas for wind energy developments and minimize this risk factor to the current condor population. In addition, our study also provides an important framework regarding terrestrial space use by condors that can be used to link habitat use to the risk of lead poisoning across the landscapes in which condors occur.

## Materials and Methods

### Ethics Statement

The condor is a federally listed endangered species, so extreme care was taken during all capture and handling procedures to minimize stress and disturbance. This study was carried out in strict accordance with the recommendations in the Guidelines to the Use of Wild Birds in Research of the Ornithological Council. There is no animal care committee that reviews research conducted under endangered species recovery permits; therefore, condor field program permits were reviewed and approved by the US Fish and Wildlife Service Permit Coordinator, the US Fish and Wildlife Service California Condor Coordinator, and the US Fish and Wildlife Service Region 8 Endangered Species Division. The use of GPS transmitters was authorized as a recovery action under section 10(a)(1)(A) with permits issued to the three release sites: Ventana Wildlife Society (# TE-026659), the Hopper Mountain National Wildlife Refuge Complex (# TE-108507), and Pinnacles National Park (# TE-157291). In addition, this work was authorized in the state of California under a separate Memorandum of Understanding between managers of each release site and the California Department of Fish and Game under sections 650 and 670.7, Title 14, California Code of Regulations.

### California Condor Release Locations and GPS Transmitter Data

**Figure 1:**
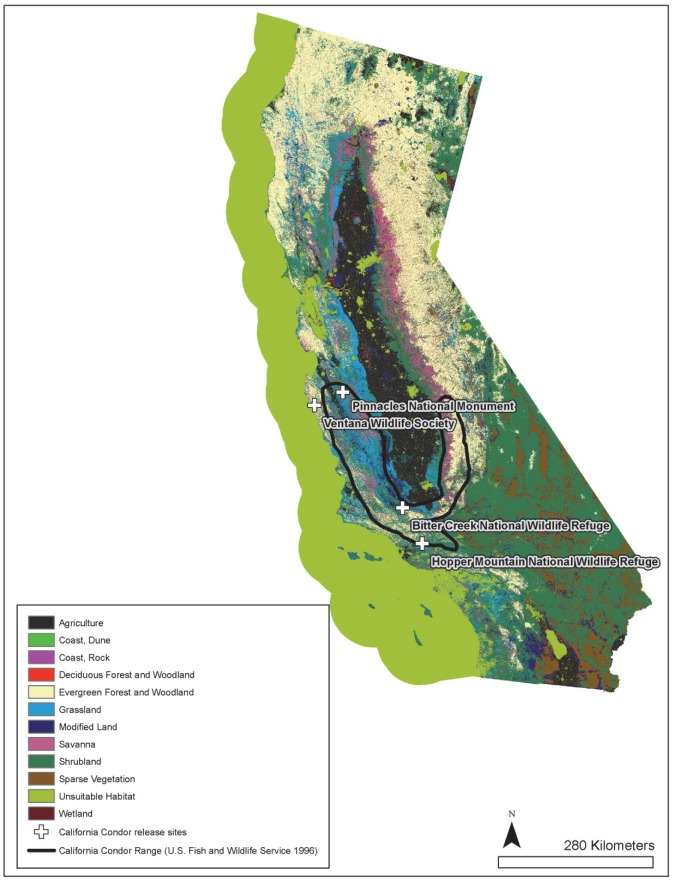
Habitat classifications of landcover in California. The range of primary concern according to the 1984 California Condor Recovery Plan [60] is shown by a solid black outline with condor release sites considered in this study (crosses). Note that Bitter Creek NWR and Hopper Mountain NWR are combined for analysis because they are both part of the Hopper Mountain National Wildlife Refuge Complex.

We used data from Global Positioning System (GPS) transmitters collected from July 2003–December 2010 to assess resource selection of condors that originated from three release sites in California (Fig. 1). Hopper Mountain National Wildlife Refuge and Bittercreek National Wildlife Refuge are part of the Hopper Mountain National Wildlife Refuge Complex and managed by the U.S. Fish and Wildlife Service (hereafter Hopper Mountain); Pinnacles National Park (hereafter Pinnacles) is managed by the National Park Service; and the Big Sur release site (hereafter Big Sur) is managed by Ventana Wildlife Society. Hopper Mountain is located inland in southern California, Pinnacles is located inland in central California's Coast Range, and Big Sur is located along the central California coast.

We captured and fitted condors with GPS transmitters (Argos/GPS PTT-100; Microwave Telemetry, Inc., Columbia, Maryland) throughout the course of the study. GPS transmitters were programmed to collect location data at hourly intervals each day from 0500–2000 h PST with a resolution of ±18 m (based on manufacturer specifications). Although we attempted to assign transmitters to sex and age classes in a balanced manner, management needs required the non-random assignment of transmitters in some cases. The number of individuals that were fitted with GPS transmitters began with two individuals in 2003 and increased to 50 individuals in 2010, reflecting the growth of the free-flying condor population in California. Because lead exposure is a serious threat to free-ranging condors [17], [47] we regularly re-captured condors to collect blood and evaluate lead levels; individuals with high levels were retained in captivity for varying lengths of time. This created gaps in location data for some individuals and led us to restrict our analysis of resource use to individuals that had a minimum of 100 locations per month to ensure adequate sampling. We used month as the temporal scale for our analysis because this time period provided an opportunity to assess potential changes in resource use across the annual cycle and enabled us to incorporate meteorological data, which were available in monthly summaries from January 2007–December 2009 (see below).

### Utilization Distributions and Home Range Delineation

We used location data to calculate utilization distributions (UDs) on a monthly basis, using individual condors as our sampling unit. UDs quantify relative space use of an individual by predicting its probability of occurrence as a function of relocation points within an area of interest, such as a monthly home range [63]. Thus, an animal's use of space can be envisioned as a three dimensional plot, with the height of the UD at any particular location within an area of interest (e.g., monthly home range) being a function of the likelihood an animal will use that location, with a greater UD height indicating a greater likelihood of use [35]. To calculate UDs, we used 99% fixed kernel density analysis [22], [54]. Initially, we evaluated the reference (*h*_ref_) and the least squares cross-validation (*h*_lscv_) smoothing parameters [25], [64] for use with our dataset. However, both smoothing parameters failed during initial analysis (see [48]) so we calculated utilization distributions by initially estimating *h*_ref_ with the Home Range Tools in ArcGIS [49], and then used Hawth's Analysis Tools for ArcGIS [4] to calculate 99% fixed-kernel monthly home ranges using a grid cell size of 100 m. We used an ad hoc smoothing parameter (*h*_adhoc_) to choose the smallest increment of *h*_ref_ that resulted in a contiguous 99% kernel polygon (i.e., 0.3*h_ref_ = *h*_adhoc_) as this minimizes overestimation of the outward boundary of the utilization distribution [3], [12]. We deemed this a reasonable approach because [1] field observations found that using the *h*_ref_ overestimated condor space use in habitats immediately surrounding high use areas that contained many overlapping location points, and [2] condors often concentrated their perching and roosting at the same distinct locations throughout the annual cycle, leading to location data that contained a large number of overlapping individual location points.

### Terrestrial-based Habitat and Meteorological Data

We used two independent datasets to quantify the terrestrial-based habitat and meteorological conditions available to free-ranging condors. In the first, we obtained Geographic Information Systems (GIS) landcover data from the U.S. Department of Agriculture that spanned the geographic range of condors in California and covered 47 ecological subregions (hereafter, ecoregions) as delineated by [11]. Next, we took 244 distinct landcover classifications from [13] for the state of California and reclassified them into 12 distinct habitat types (Document S1) to eliminate redundancy in landcover classifications and to express landcover in units that were ecologically relevant to condors. We then overlaid habitat data on ecoregion data so that we could assess resource selection on two nested spatial scales (habitat was nested within ecoregion). We used this approach because wind resource data were available at a coarser spatial scale than habitat data, requiring analysis on the ecoregion scale (see below).

In the second dataset, we obtained output from the North American Mesoscale (NAM) weather prognostication model run by NOAA's National Centers for Environmental Prediction (NCEP). This model forecasts atmospheric properties over the United States at a resolution of 12 km [23]. This model solves the atmospheric primitive differential equations, in particular incorporating surface factors such as solar heating, soil temperature, soil moisture, and vegetative type which vary over the 12 km NAM grid. Because this output did not directly provide the parameters most relevant to soaring flight, we used it to estimate three types of wind resources: (1) maximum thermal height, equivalent to the boundary layer height under convective conditions [18], [2] convective thermal velocity, which depends upon the surface heating rate and boundary layer depth [18], and (3) wind speed, which was averaged vertically over the boundary layer depth. Values were computed near mid-day (21Z) and then averaged into monthly values at each point in the 12 km NAM grid. Meteorological data were only available for the years 2007–2009, so we used data from these years to calculate an average monthly value for each 12×12 km cell for each of the three wind meteorological parameters (i.e., thermal height, thermal velocity, and wind speed).

### Quantifying Condor Resource Selection

We used UDs to predict the probability of occurrence within each habitat type within each monthly home range, which served as proportional, habitat-specific measures of resource use. We then compared habitat use measures to the proportion that each habitat was available for monthly home range estimates by calculating resource selection ratios [i.e., *ln*(rf)] as follows:
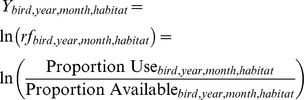
Values of *ln*(rf)>0 indicate a habitat whose use exceeded its availability (i.e., the habitat is selected), values of *ln*(rf) = 0 indicate a habitat used in proportion to its availability, and values of *ln*(rf)<0 indicate a habitat whose use was less than its availability (i.e., the habitat is avoided). We used a *ln* transformation to make the analysis “symmetric” around 1 (e.g., a selection ratio of ½ is the same distance away from 1 as a selection ratio of 2).

We also used UDs to predict the probability of occurrence at the ecoregion level within the monthly home range as a measure of use, compared it to the area of the ecoregion within the monthly home range as a measure of availability, and related the *ln*(rf) to the availability of meteorological parameters in each ecoregion. Values for meteorological parameters were found by determining which 12×12 km cells overlapped each ecoregion within the monthly home range and then calculating an average over all 12×12 km cells within an ecoregion for each meteorological parameter. We calculated a selection ratio each month for each bird's use of each ecoregion relative to the area of each ecoregion that fell within that month's home range:
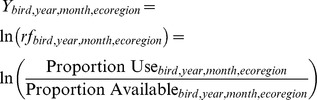



### Statistical Analysis

We performed two levels of statistical analysis. In the first, we restricted our examination to assess the relative use of a habitat relative to its availability for each bird-year-month combination and did not consider meteorological parameters. After calculating a selection ratio, *ln*(rf), for each habitat, we then used a repeated-measures mixed linear modeling approach to model resource use for each habitat relative to intrinsic characteristics of individuals (i.e., age, sex, breeding status [two levels each]) and factors related to the recovery program (i.e., rearing method [two levels], release location [three levels]). For this analysis, we classified individuals as either immature (i.e., juvenile [0–2 years], sub-adult [3–5 years]) or adult [≥6 years]) because breeding does not occur regularly until 6 years of age [56]. We classified adults as breeders if they frequented a nesting site and were found to have laid an egg. We classified the rearing method of individuals as either raised in the wild or reared in captivity. We used the PROC MIXED procedure in SAS/STAT version 9.3 for Windows to account for the repeated nature of the measurements, with *ln*(rf) as the repeated measure; age, sex, breeding status, release site, and rearing method as fixed categorical effects; and individual bird was included as a random factor. Initially, we found that an autoregressive covariance structure outperformed a compound symmetrical covariance structure so it was retained for all subsequent models. Because our data were unbalanced, we calculated least-squares marginal means (LSMEANS) for effect sizes, with a Tukey-Kramer adjustment for all multiple comparisons, and used the Kenward-Rogers method to calculate degrees of freedom for contrasts and estimates. We note that although spatial autocorrelation arises through the use of a fixed-kernel procedure to construct the UD, we used individual condors as our sampling unit. This allowed us to ignore spatial autocorrelation between individual locations because previous work has demonstrated that individual model coefficients are unbiased even when autocorrelation is present [1], [16], [27], [29], [36].

In the second analysis, we again computed a relative use of each ecoregion to its availability for each bird-year-month-ecoregion combination. We then fit a mixed-effects linear model for each ecoregion (again using an autoregressive covariance structure to account for repeated measurements on the same bird over time) where the *ln*(rf) was a function of bird characteristics, month (as a categorical variable), time, and the meteorological parameters. We used an information-theoretic approach [8] because bird characteristics were not of interest for this part of our analysis. We created an initial model set containing the effects of bird-specific characteristics (i.e., sex, age, breeding status, release site, rearing method) and combinations of the meteorological parameters. All models were fit using maximum likelihood and ranked by AICc [8]. Model weights were used to estimate the model-averaged estimates and model-averaged unconditional standard error of the coefficients for the meteorological parameters which include variation due to model uncertainty. We did not account for the correlation in use among ecoregions (i.e., if relative use of one ecoregion by a bird in a month goes up, relative use must necessarily go down in other ecoregions), nor were we able to incorporate habitat information in models that included meteorological variables on the level of ecoregion because meteorological variables were estimated at different spatial scales. We note that to assess the relative effect of each meteorological parameter our model averaging approach required that we hold the other two meteorological parameters constant.

We report least squares marginal means and associated 95% confidence intervals (CIs) unless otherwise noted. We only investigated effects whose *P*<0.01 in the first analysis to reduce Type I errors that stem from testing multiple hypotheses. For the second analysis, we used the model-averaged confidence intervals for the effects of the meteorological parameters in each ecoregion to determine if the value of 0 (indicating no effect) was included in the interval. We also computed the sum of the model weights for models that contained the meteorological parameters as a measure of importance of each meteorological parameter.

## Results

We collected GPS locations from 74 individual condors; males had greater representation in our dataset than females (43 vs. 31 individuals). Slightly more than half of all individuals (i.e., 53%) were released from Hopper Mountain, with 27% released from Big Sur and 20% released from Pinnacles. We examined a mean of 18 (range: 1–72 months) monthly home ranges per individual with a mean of 328 (range: 100–517) individual locations per month.

**Figure 2:**
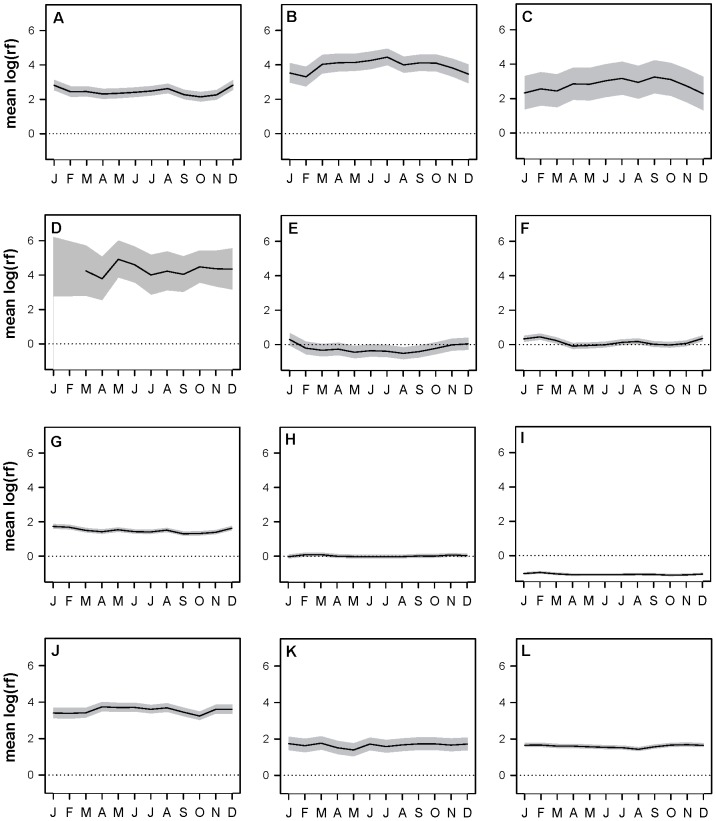
Coarse-scale condor habitat use. Mean selection ratios for each of 12 habitat types assessed across the annual cycle. Gray shaded area represents 95% confidence intervals. A = agriculture, B = coastal dune, C = coastal rock, D = deciduous forest, E = evergreen forest, F = grassland, G = modified land, H = savanna, I = shrubland, J = sparse vegetation, K = unsuitable habitat, L = wetland.

Results from our mixed model analysis showed strong evidence of a month and release site effect across most habitat types but no evidence of a sex, age class, breeding status, or rearing effect for most habitats (Table 1). There was some evidence of a change in the extent of habitat use within the annual cycle; however, the estimated marginal mean *ln*(rf) by month, averaged over all other effects in the model, indicated that month effects were relatively small compared to the effect of the habitat itself (Fig. 2). There was consistent evidence that condors selected some habitats during the course of the study (e.g., dune and rock habitats in coastal areas), whereas other habitats were used significantly less than their availability (e.g., shrubland, evergreen forest; Document S2). It should be noted that habitat use plots do not account for area, so an increase in use for one habitat does not necessarily correlate with a decrease in use for other habitats. We found some evidence of temporal trends in habitat use over the course of the study (Document S2), although it is unclear whether changes over time were due to changes in preference of individuals as they aged; however, we found no evidence of a large effect of age (Document S2). In addition, there was an inconsistent effect of release site on use of different habitats (Document S2).

**Table 1:** Resource selection ratios [i.e., *ln*(rf)] for 12 terrestrial-based habitat types for the California condor in California.

	Effect						
	Month	Release site	Sex	Sex*Month	Age class	Breeder	Rearing method
Habitat	*P*-value	*P*-value	*P*-value	*P*-value	*P*-value	*P*-value	*P*-value
Agriculture	**<0.0001**	**<0.0001**	0.1822	0.3575	0.4151	0.1125	0.5772
Coast (dune)	**0.0018**	**<0.0001**	0.0362	0.9651	0.7095	0.1199	—
Coast (rock)	**<0.0001**	**<0.0001**	0.2573	0.1854	0.8045	0.9819	0.0790
Deciduous forest	0.2443	0.0141	0.8113	0.9419	0.5210	0.6348	0.7283
Evergreen forest	**<0.0001**	**<0.0001**	0.9318	0.0342	0.5328	0.0627	0.6330
Grassland	**<0.0001**	**<0.0001**	0.4211	**0.0003**	0.8399	0.0442	0.7494
Modified land	**<0.0001**	**<0.0001**	0.5513	0.1363	0.6731	0.2808	0.2658
Savanna	0.4893	**<0.0001**	0.4208	0.1834	0.7896	0.0582	0.2988
Shrubland	0.0955	**<0.0001**	0.7892	0.0584	0.8069	0.0005	0.3628
Sparse vegetation	**0.0039**	**<0.0001**	0.8786	0.6609	0.1007	0.9641	**0.0031**
Unsuitable habitat	0.1056	**<0.0001**	0.1523	0.8310	0.4458	0.7092	0.6505
Wetland	**0.0002**	**<0.0001**	0.9471	0.0925	0.9222	0.4383	0.8881

Significant *P*-values (i.e., <0.01) are highlighted in bold text.

**Figure 3:**
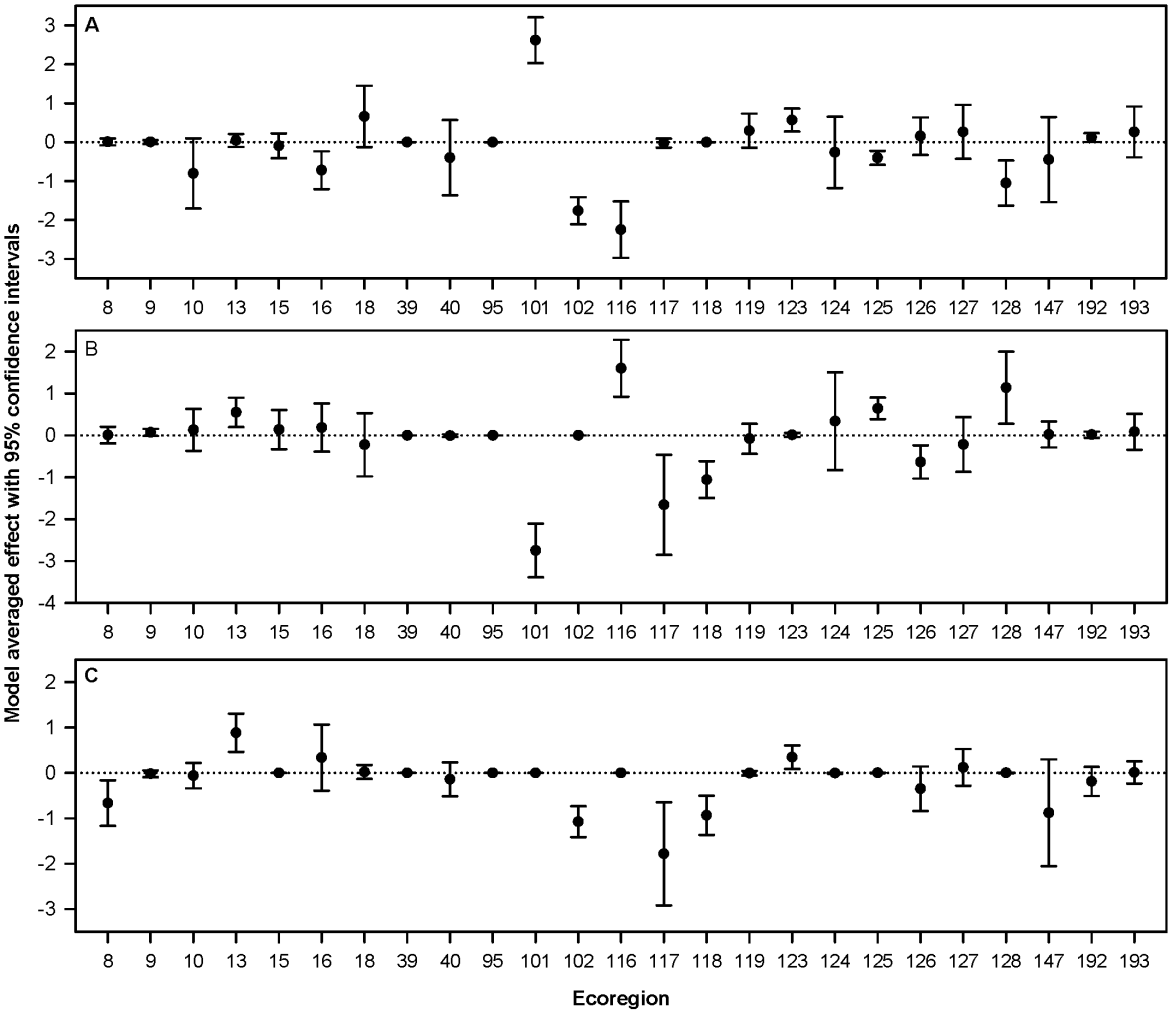
Meteorological parameters influenced condor space use. Estimated mean marginal effects and associated 95% CIs for each of three monthly-averaged meteorological parameters by ecoregion. A = thermal height (km), B = thermal velocity (m/s), C = wind speed (m/s).

**Figure 4:**
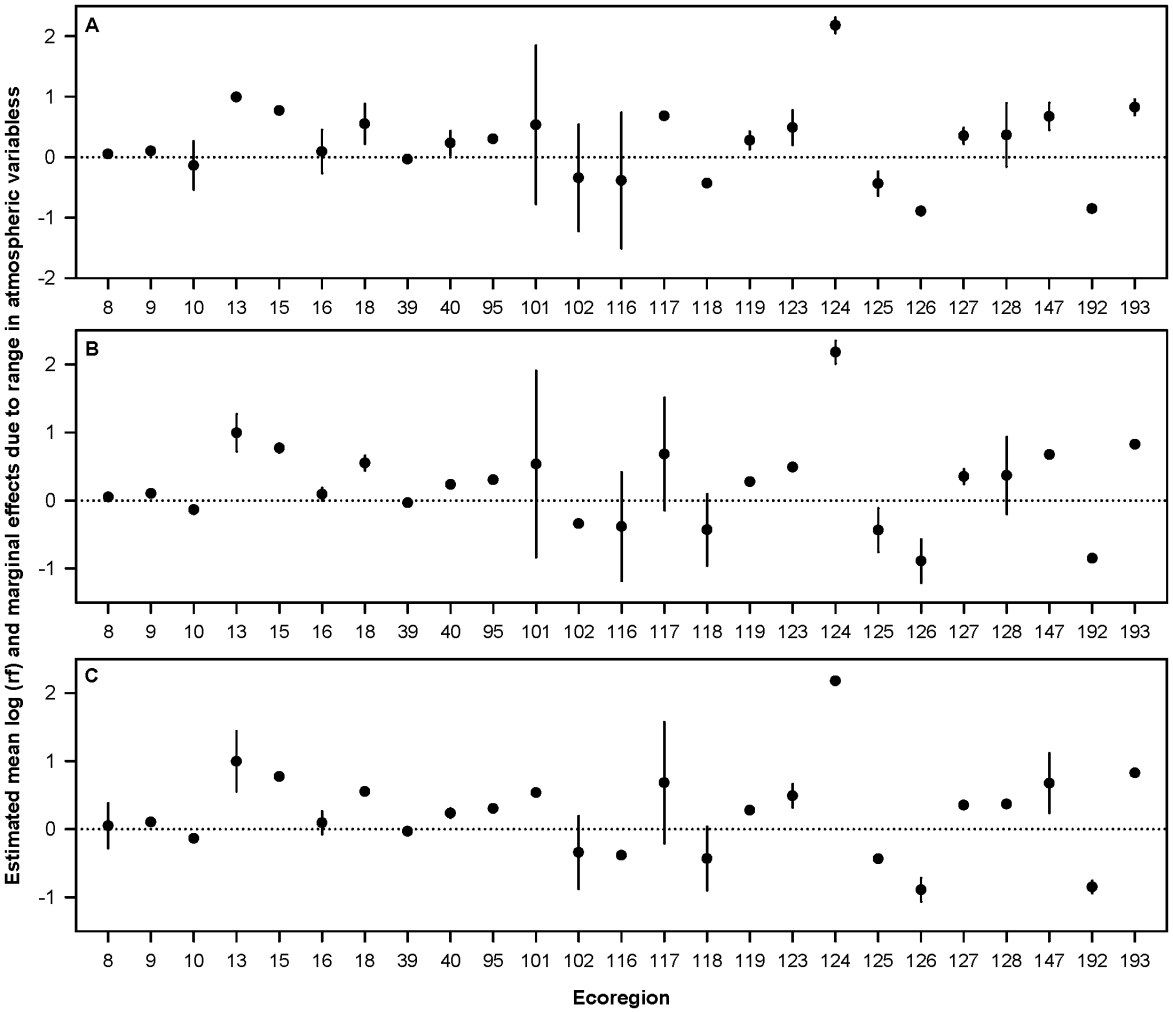
Condor selection ratios by ecoregion. Mean monthly *ln*(rf) values for each ecoregion (filled points) and the effect of the meteorological parameters and the range of monthly average values for each meteorological value (vertical bars). Thus, the vertical bars are not error estimates but instead represent the range of mean selection ratio across the annual cycle for each ecoregion. A = thermal height (km), B = thermal velocity (m/s), C = wind speed (m/s).

Based on our preliminary analysis of the relationship between *ln*(rf) and meteorological parameters, we eliminated 22 ecoregions because of inadequate data resulting in 25 ecoregions which we examined further (Documents S3, S4). Overall, ecoregions varied markedly in the variation of mean values for meteorological parameters across the annual cycle (Table 2). Results from the mixed model analysis showed some evidence of a month and release site effect across a subset of ecoregions, and for most ecoregions there was no evidence of a sex, age, breeding status, or rearing effect (Table 3). We found models that included at least one of the meteorological parameters often had the largest weight among the model set for many ecoregions (Document S5). Most of the model weight favored models with meteorological parameters, but meteorological parameters were not equally important in all ecoregions (Table 4, Fig. 3). Finally, changes in meteorological parameters across the months were linked with changes in usage in some ecoregions (Fig. 4, Documents S6, S7). For example, in ecoregion 18, the model averaged coefficient of thermal height (km) of 1.080 (see Table 4) indicates that for every 1 km of change in the thermal height, the *ln*(rf) increased by 1.080, or exp(1.080) = 2.95 times.

**Table 2:** Maximum and minimum average values for meteorological parameters observed across the annual cycle by ecoregion.

	Thermal height (km)		Thermal velocity (m/s)		Wind speed (m/s)	
Ecoregion	Min	Max	Min	Max	Min	Max
8	0.71	1.79	1.32	2.78	2.52	5.56
9	0.22	0.43	0.62	0.96	4.48	6.84
10	0.01	0.46	0.44	1.20	4.56	7.59
13	0.71	1.22	1.74	2.49	4.71	6.94
15	0.86	1.41	1.74	2.68	4.98	7.64
16	0.92	1.44	1.71	2.70	4.56	7.86
18	0.69	1.30	1.40	2.51	3.07	5.00
39	1.49	3.14	1.49	3.26	3.31	6.07
40	1.58	3.18	1.59	3.30	3.14	6.12
95	1.44	2.96	1.44	3.27	3.91	6.99
101	1.26	2.44	1.33	3.06	3.11	6.02
102	1.68	3.37	1.56	3.37	3.37	6.38
116	0.83	2.01	1.39	2.94	2.56	5.80
117	1.02	2.30	1.39	3.09	2.27	6.00
118	1.03	1.83	1.40	2.62	2.69	6.00
119	0.67	1.78	1.39	2.90	3.03	5.53
123	1.61	2.92	1.60	3.24	3.02	6.03
124	1.56	2.90	1.61	3.25	3.00	6.00
125	1.06	1.59	1.71	2.64	4.47	7.50
126	1.58	2.92	1.60	3.15	3.55	6.84
127	0.99	1.86	1.64	2.79	4.45	8.03
128	0.71	1.38	1.39	2.52	3.09	5.17
147	1.37	2.97	1.54	3.21	4.11	7.72
192	0.03	0.05	0.28	0.55	5.15	8.71
193	1.42	3.06	1.51	3.35	4.14	7.43

**Table 3:** Summary of main effects tests for resource selection ratios [i.e., *ln*(rf)] for 25 ecoregions within the geographic range of the California condor in California.

	Effect						
	Month	Release site	Sex	Sex*Month	Age class	Breeder	Rearing method
Ecoregion	*P*-value	*P*-value	*P*-value	*P*-value	*P*-value	*P*-value	*P*-value
8	0.0045	0.4483	0.5121	0.9912	0.5859	0.1608	—
9	0.2847	0.1892	0.2008	0.7818	0.3063	0.3642	—
10	0.9621	0.0101	0.6920	0.5020	0.3345	0.0125	0.3138
13	**<0.0001**	0.9586	0.5311	0.0273	0.0408	**<0.0001**	0.4239
15	**<0.0001**	**<0.0001**	0.3965	0.8806	0.1573	0.1588	0.6373
16	0.0953	0.2163	0.4318	0.5188	**0.0008**	0.0633	0.6822
18	0.6008	0.5063	0.0284	0.4302	0.8578	0.8077	0.2760
39	**<0.0001**	0.0100	0.4088	0.2591	0.0727	0.1724	0.3928
40	**<0.0001**	0.1849	0.5433	0.9642	0.7856	0.7305	0.2927
95	**<0.0001**	0.0822	0.3638	0.3677	0.1403	0.3802	0.5309
101	**0.0016**	0.5534	0.7215	0.6332	0.2344	0.6091	0.7192
102	**<0.0001**	**0.0051**	0.8744	0.0360	0.5336	0.2331	0.4938
116	0.1611	0.0121	0.2216	0.4477	0.0711	0.7947	—
117	0.0896	0.4663	0.1602	0.5138	0.6775	0.0244	—
118	0.2852	**0.0099**	0.9345	0.7368	0.8832	0.0765	0.5288
119	0.1953	0.1635	0.9862	0.4481	0.4847	0.5123	—
123	0.0770	**<0.0001**	0.8520	0.7376	0.9412	0.4959	0.5868
124	**<0.0001**	**0.0002**	0.2732	0.5612	0.4190	0.6011	0.5563
125	**0.0002**	**<0.0001**	0.9459	0.8724	**<0.0001**	**0.0033**	0.7672
126	**<0.0001**	0.6012	0.9113	0.9943	**<0.0001**	**0.0035**	0.8615
127	0.0337	0.0191	0.2613	0.1148	0.0372	0.7334	**0.0098**
128	0.1659	0.9402	0.4217	0.1814	0.5349	0.3153	0.5627
147	0.3388	0.7824	0.3057	0.5684	0.6589	0.0630	0.4583
192	0.1031	0.2506	0.1294	0.8957	0.5947	0.7376	—
193	0.0650	0.5373	0.8538	0.2130	0.7225	0.2278	0.1533

See Document S3 for the names that correspond to ecoregion codes. Significant *P*-values (i.e., <0.01) are highlighted in bold text.

**Table 4:** Model weighted estimates of meteorological parameters for 25 ecoregions in which condors were observed.

	Thermal height (km)			Thermal velocity (m/s)			Wind speed (m/s)		
Ecoregion	Estimate	95% CI	Weight	Estimate	95% CI	Weight	Estimate	95% CI	Weight
8	0.008	(−0.075, 0.091)	0.15	0.006	(−0.129, 0.142)	0.40	**−0.218**	**(−0.384, −0.053)**	**0.94**
9	0.021	(−0.213, 0.256)	0.31	0.205	(−0.039, 0.450)	0.84	−0.008	(−0.039, 0.024)	0.39
10	−1.788	(−3.800, 0.225)	0.98	0.174	(−0.489, 0.838)	0.30	−0.020	(−0.112, 0.072)	0.22
13	0.088	(−0.239, 0.415)	0.09	**0.734**	**(0.264, 1.204)**	**0.91**	**0.397**	**(0.208, 0.586)**	**1.00**
15	−0.168	(−0.745, 0.409)	0.16	0.146	(−0.354, 0.646)	0.16	0.000	(−0.000, 0.000)	0.00
16	**−1.363**	**(−2.282, −0.444)**	**1.00**	0.189	(−0.393, 0.770)	0.32	0.102	(−0.118, 0.323)	0.55
18	1.080	(−0.206, 2.366)	0.83	−0.200	(−0.879, 0.479)	0.38	0.011	(−0.068, 0.091)	0.16
39	0.000	(−0.000, 0.000)	0.00	−0.000	(−0.000, 0.000)	0.00	−0.000	(−0.000, 0.000)	0.00
40	−0.250	(−0.857, 0.357)	0.40	−0.004	(−0.024, 0.015)	0.01	−0.047	(−0.173, 0.078)	0.38
95	−0.000	(−0.000, 0.000)	0.00	0.000	(−0.000, 0.000)	0.00	0.000	(−0.000, 0.000)	0.00
101	**2.233**	**(1.732, 2.733)**	**1.00**	**−1.590**	**(−1.960, −1.220)**	**1.00**	0.000	(−0.000, 0.000)	0.00
102	**−1.043**	**(−1.250, −0.837)**	**1.00**	0.000	(−0.000, 0.000)	0.00	**−0.356**	**(−0.468, −0.244)**	**1.00**
116	**−1.891**	**(−2.501, −1.281)**	**1.00**	**1.030**	**(0.592, 1.467)**	**1.00**	0.000	(−0.000, 0.000)	0.00
117	−0.019	(−0.109, 0.072)	0.03	**−0.978**	**(−1.682, −0.273)**	**0.96**	**−0.478**	**(−0.782, −0.173)**	**0.98**
118	−0.000	(−0.001, 0.001)	0.00	**−0.867**	**(−1.226, −0.507)**	**1.00**	**−0.282**	**(−0.412, −0.152)**	**1.00**
119	0.266	(−0.128, 0.660)	0.81	−0.053	(−0.290, 0.184)	0.45	−0.003	(−0.022, 0.017)	0.20
123	**0.435**	**(0.211, 0.660)**	0.97	0.007	(−0.024, 0.037)	0.04	**0.115**	**(0.029, 0.201)**	**0.96**
124	−0.194	(−0.876, 0.488)	0.15	0.206	(−0.504, 0.916)	0.15	−0.001	(−0.005, 0.003)	0.01
125	**−0.753**	**(−1.091, −0.415)**	**0.99**	**0.696**	**(0.418, 0.974)**	**1.00**	0.001	(−0.002, 0.003)	0.01
126	0.115	(−0.243 0.472)	0.26	**−0.412**	**(−0.668, −0.155)**	**0.99**	−0.106	(−0.255, 0.043)	0.72
127	0.303	(−0.494, 1.099)	0.51	−0.188	(−0.753, 0.377)	0.46	0.034	(−0.079, 0.147)	0.34
128	**−1.578**	**(−2.452, −0.705)**	**0.98**	**1.009**	**(0.246, 1.772)**	**0.95**	0.001	(−0.004, 0.005)	0.01
147	−0.280	(−0.963, 0.402)	0.53	0.014	(−0.173, 0.200)	0.25	−0.244	(−0.570, 0.083)	0.84
192	**6.562**	**(0.251, 12.873)**	**0.89**	0.059	(−0.216, 0.334)	0.15	−0.052	(−0.142, 0.038)	0.70
193	0.160	(−0.237, 0.558)	0.53	0.047	(−0.186, 0.280)	0.38	0.003	(−0.072, 0.078)	0.32

Estimates whose 95% confidence intervals did not overlap with zero are highlighted in bold text.

## Discussion

### Resource selection relative to terrestrial-based habitats

We found that free-ranging individual condors varied significantly in the terrestrial habitats they used within monthly home ranges when we assessed habitat use separately from meteorological data. Selection ratios were greatest for coastal dune, deciduous forest (including oak woodland), and sparse vegetation habitats; lowest for grassland and savanna habitats; and condors avoided evergreen forest and shrubland. Previous authors indicated that grassland and oak (*Quercus* spp.) savanna comprises historically important foraging areas for condors [10], [26], [56], so it was somewhat surprising that our analysis revealed that condors did not exhibit strong selection of either grassland or savanna habitats within their home range. This may be because selection of grassland and savanna habitats occurs at broader scales than we examined (i.e., first- or second-order selection; see [24]), or because previous authors merely described use of habitats and did not report selection relative to availability. Selection for sparse vegetation and coastal habitats likely reflects several important features of condor foraging ecology: habitats where food resources (i.e., animal carcasses) and potential predators can be detected, and habitats that have terrain that is amenable for taking off from the ground in flight [56]. Strong selection for coastal habitats is noteworthy for two reasons. First, condor diets during recent times (i.e., 1993 to 2001) were reported to have shifted away from marine mammals and towards terrestrial animals, especially domestic cattle [10]. However, since 1999, condors have been observed foraging on coastal marine mammals (e.g., gray whale [*Eschrichtius robustus*] and California sea lion [*Zalophus californianus*]) in central California and concern has increased regarding whether environmental contaminants found in marine mammal tissues threaten condor populations in this region [7]. Thus, use of coastal habitats by the condors in our dataset, coupled with foraging observations of condors on marine mammal resources, suggests a return to feeding in coastal areas in recent times. Second, coastal habitats comprise a very small portion of the total landcover in California (∼0.1%) but the high use and selection of these areas by condors in central California indicates it is an especially important habitat for this species, probably due to a combination of foraging resource and onshore winds that facilitate soaring flight.

We found little evidence that intrinsic characteristics of individuals and factors related to the recovery program, aside from release site, influenced the use of terrestrial-based habitats. Although we did detect a significant effect of month, habitat selection ratios were rather consistent throughout the year for most habitats, indicating that the month effects are relatively small when compared to the type of habitat. In contrast, we did find a significant and consistent effect of release site on habitat use by condors. Release sites are spatially distinct and exhibit coarse-scale differences in habitat types (Fig. 1), so these effects are likely influenced by differences in habitat availability surrounding the release sites, and they may not be indicative of consistent differences in habitat selection by condors among the different release sites.

### Resource selection relative to meteorological conditions

Our results provide the first quantitative evidence that resource selection by condors is linked to meteorological parameters in a manner that is thought to facilitate soaring flight. These effects were present despite the relatively coarse scale at which weather data were available and an analysis based on monthly averages of meteorological parameters, both of which are expected to reduce our ability to detect significant effects. Thus, our results indicate that meteorological conditions can have a particularly strong effect on condor use of some ecoregions. Our data indicate that the thermal characteristics we measured (i.e. thermal height and velocity) generally had a stronger influence on selection by condors among ecoregions than wind speed averaged across the depth of the boundary layer. Selection for thermal characteristics is expected for condors, which are similar to vultures and other large birds in their use of thermals for large-scale movements [6], [41]–[43], [57]. Thermals are exploited by soaring birds because they allow individuals to minimize energetic output during large-scale movements [43], [53], such as searching for carrion. Despite the recognized importance of thermals for movement, California condors are likely to use orthographic lift to facilitate soaring flight as has been shown in a Peruvian coastal population of the closely-related Andean condor (*Vultur gryphus* [37], [44]. Orthographic lift is likely to be used most often in coastal areas (e.g., Big Sur region) where on-shore wind conditions create lift and facilitate foraging of marine mammal carrion [7], in addition to mountaintop areas that experience updrafts that are strong enough to support the weight of condors (ca. 8.2 kg [56]). Thus, consideration of the diverse habitats in which we detected condors indicates that all of the atmospheric properties we measured are likely to be important for condors throughout their California range.

Our results also indicate that wind resources were linked to differences in the extent of condor use at the ecoregion level. The magnitude of model averaged effects for the three meteorological parameters were often similar among ecoregions, and for many ecoregions effects did not differ from zero. However, meteorological parameters did have a significant influence on condor use for several geographically distinct ecoregions (e.g., 101, 116, 125, 128; see Fig. 4, Document S4), and these effects included selection for and avoidance of ecoregions relative to the atmospheric parameter being examined. We also detected a link between meteorological parameters and individual characteristics as well as factors related to the recovery program; however, the strength of these relationships varied substantially relative to habitat type and ecoregion, with month and release site having the strongest effect. Thus, a complex picture is emerging regarding how individual condors vary in their use of space relative to meteorological variables, how these relationships can change across landscape scales, and how they differ relative to individual and program-related characteristics. It is worth noting that measures of model-averaged effect size (Figure 3) and selection ratios and the degree of selection exhibited by condors (Figure 4) can vary independently of each other. For example, consider ecoregion 117 in panels Figure 3C and Figure 4C. In Figure 3C, the value for ecoregion 117 indicates that there was a significant effect size for condor selection for wind speed in this location (i.e., there is less use when wind speed increases). In Figure 4, the value for ecoregion 117 indicates that the mean range of selection ratios across the annual cycle was rather broad and covered zero. Taken together, this indicates that condors appeared to avoid this ecoregion during times of high winds yet find it suitable otherwise. These results highlight how meteorological variables are dynamic throughout the annual cycle within given ecoregions and indicate such changes can have strong influence on ecoregion use by condors. Furthermore, they suggest research that considers decisions made by an individual on small spatial and temporal scales may be especially useful for furthering our understanding of resource selection in this species [19], [39].

### Implications of resource selection for condor conservation

The California condor is noteworthy because it is one of the most endangered birds in the world and, as demonstrated in this study, its use of space is influenced by meteorological conditions. Our analysis indicates that condors use a wide range of terrestrial habitats in California, and their movements in some areas are influenced in part by meteorological conditions. These results therefore add significantly to previous data and observations on condor movement and spatial ecology [38], [48] by demonstrating that condors are not restricted in their use of any single habitat but instead use all available coarse-scale habitat types in California. Lead poisoning is considered to be the most serious threat to recovery of the condor population at the current time [17], [47]; however, the spatial extent of lead availability on the landscape and its potential to poison condors is currently unknown. In addition to the lead issue, concern has also increased in recent years regarding potential impacts of wind energy developments on condors in California, where the greatest number of free-flying individuals currently reside [61]. This has arisen in part because of recent legislation that requires California to increase the amount of electricity generated from renewable energy resources to 33% of total sales of electricity by the end of 2020 [46]. Our results are informative in both of these contexts because they demonstrate that condors alter their use of habitat types across the annual cycle and relative to local meteorological conditions, and that condors use all available habitats at least to some degree during the course of the year. Thus, additional studies that focus on documenting condor lead exposure across the landscape will be especially valuable to understand the spatio-temporal dynamics of lead exposure and condor poisoning events. In addition, study of finer scale movements of condors in areas being evaluated for wind development would inform siting considerations, development of curtailment measures, design of deterrence devices, and other measures to reduce the risk of collision of condors with anthropogenic structures linked to wind energy developments. Finally, movement studies that go beyond space use and incorporate a temporal scale will be especially valuable for understanding decisions condors make when selecting resources. Such studies have already shown great promise in enhancing our understanding of the fine-scale decisions made by obligate scavengers during large-scale movements [6], [53]), and should provide important data that can help with the conservation and management of this critically endangered species.

## Supporting Information

Document S1 **Table of 244 distinct landcover classifications taken from** [**13**] **for California and reclassified into 12 distinct habitat types.**
(PDF)

Document S2 **Plots of marginal mean** ***ln*** **(rf) by habitat for sex effects, age and age class effects, release site, rearing method, and breeding status.**
(PDF)

Document S3 **Ecoregions as delineated by** [**11**] **and used to assess California Condor resource selection relative to meteorological parameters.**
(PDF)

Document S4 **Map of 25 ecoregions in which California Condors were observed in reasonable numbers to quantify meteorological parameters in the study.**
(PDF)

Document S5 **Summary of the number of ecoregion models and their parameters that had ΔAIC≤3 from candidate models.**
(PDF)

Document S6 **Plots for three meteorological parameters and raw** ***ln*** **(rf) values plotted against months in the annual cycle for each of the 25 California ecoregions examined in the study.**
(PDF)

Document S7 **Plots for three meteorological parameters and mean** ***ln*** **(rf) values plotted against months in the annual cycle for each of the 25 California ecoregions examined in the study.**
(PDF)
