# Supplementary material for: Resource Selection by the California Condor (Gymnogyps californianus) Relative to Terrestrial-Based Habitats and Meteorological Conditions
Source: PLoS One. 2014 Feb 11;9(2):e88430. doi: 10.1371/journal.pone.0088430 (PMC3921182; doi:10.1371/journal.pone.0088430)
Supplement: Document S1 — Table of 244 distinct landcover classifications taken from [13] for California and reclassified into 12 distinct habitat types. (PDF) [file pone.0088430.s001.pdf]

Document S1. Table of 244 distinct landcover classifications taken from Comer et al. (2003) for the state of California and reclassified into 12 distinct habitat types to eliminate redundancy in landcover classifications and to express landcover in units that were ecologically relevant to condors.

Habitat type	Original name
Agriculture	Ag Crop Production
Coast, Dune	Mediterranean California Southern Coastal Dune
Coast, Dune	Mediterranean California Northern Coastal Dune
Coast, Dune	North Pacific Maritime Coastal Sand Dune and Strand
Coast, Rock	North Pacific Coastal Cliff and Bluff
Coast, Rock	Mediterranean California Coastal Bluff
Deciduous Forest	North Pacific Oak Woodland
Deciduous Forest	Northern Rocky Mountain Western Larch Savanna
Deciduous Forest	Rocky Mountain Aspen Forest and Woodland
Deciduous Forest	Rocky Mountain Bigtooth Maple Ravine Woodland
Deciduous Forest	Northwestern Great Plains Aspen Forest and Parkland
Evergreen Forest	Central and Southern California Mixed Evergreen Woodland
Evergreen Forest	California Coastal Redwood Forest
Evergreen Forest	Colorado Plateau Pinyon-Juniper Woodland
Evergreen Forest	Columbia Plateau Western Juniper Woodland and Savanna
Evergreen Forest	East Cascades Mesic Montane Mixed-Conifer Forest and Woodland
Evergreen Forest	Great Basin Pinyon-Juniper Woodland
Evergreen Forest	Inter-Mountain Basins Subalpine Limber-Bristlecone Pine Woodland
Evergreen Forest	Klamath-Siskiyou Lower Montane Serpentine Mixed Conifer Woodland
Evergreen Forest	Klamath-Siskiyou Upper Montane Serpentine Mixed Conifer Woodland
Evergreen Forest	Madrean Encinal
Evergreen Forest	Madrean Lower Montane Pine-Oak Forest and Woodland
Evergreen Forest	Madrean Pinyon-Juniper Woodland
Evergreen Forest	Madrean Upper Montane Conifer-Oak Forest and Woodland
Evergreen Forest	Mediterranean California Dry-Mesic Mixed Conifer Forest and Woodland
Evergreen Forest	Mediterranean California Mesic Mixed Conifer Forest and Woodland
Evergreen Forest	Mediterranean California Mixed Oak Woodland
Evergreen Forest	Mediterranean California Lower Montane Black Oak-Conifer Forest and Woodland
Evergreen Forest	California Montane Jeffrey Pine-(Ponderosa Pine) Woodland
Evergreen Forest	Mediterranean California Red Fir Forest
Evergreen Forest	Mediterranean California Subalpine Woodland
Evergreen Forest	North Pacific Dry Douglas-fir-(Madrone) Forest and Woodland
Evergreen Forest	North Pacific Hypermaritime Sitka Spruce Forest
Evergreen Forest	North Pacific Maritime Dry-Mesic Douglas-fir-Western Hemlock Forest
Evergreen Forest	North Pacific Maritime Mesic Subalpine Parkland
Evergreen Forest	North Pacific Maritime Mesic-Wet Douglas-fir-Western Hemlock Forest
Evergreen Forest	North Pacific Mountain Hemlock Forest
Evergreen Forest	North Pacific Mesic Western Hemlock-Silver Fir Forest
Evergreen Forest	Mediterranean California Mixed Evergreen Forest
Evergreen Forest	Northern California Mesic Subalpine Woodland
Evergreen Forest	Northern Rocky Mountain Dry-Mesic Montane Mixed Conifer Forest
Evergreen Forest	Northern Rocky Mountain Subalpine Woodland and Parkland
Evergreen Forest	Northern Rocky Mountain Mesic Montane Mixed Conifer Forest
Evergreen Forest	Rocky Mountain Foothill Limber Pine-Juniper Woodland
Evergreen Forest	Rocky Mountain Lodgepole Pine Forest
Evergreen Forest	Southern Rocky Mountain Dry-Mesic Montane Mixed Conifer Forest and Woodland
Evergreen Forest	Southern Rocky Mountain Mesic Montane Mixed Conifer Forest and Woodland
Evergreen Forest	Northern Rocky Mountain Ponderosa Pine Woodland and Savanna
Evergreen Forest	Southern Rocky Mountain Ponderosa Pine Woodland
Evergreen Forest	Rocky Mountain Subalpine Dry-Mesic Spruce-Fir Forest and Woodland
Evergreen Forest	Rocky Mountain Subalpine Mesic-Wet Spruce-Fir Forest and Woodland
Evergreen Forest	Rocky Mountain Subalpine-Montane Limber-Bristlecone Pine Woodland
Evergreen Forest	Sierra Nevada Subalpine Lodgepole Pine Forest and Woodland
Evergreen Forest	Southern Rocky Mountain Pinyon-Juniper Woodland

Evergreen Forest	Middle Rocky Mountain Montane Douglas-fir Forest and Woodland
Evergreen Forest	Rocky Mountain Poor-Site Lodgepole Pine Forest
Evergreen Forest	California Coastal Closed-Cone Conifer Forest and Woodland
Evergreen Forest	Sierran-Intermontane Desert Western White Pine-White Fir Woodland
Evergreen Forest	North Pacific Hypermaritime Western Red-cedar-Western Hemlock Forest
Evergreen Forest	North Pacific Dry-Mesic Silver Fir-Western Hemlock-Douglas-fir Forest
Evergreen Forest	East Cascades Oak-Ponderosa Pine Forest and Woodland
Evergreen Forest	Inter-Mountain Basins Aspen-Mixed Conifer Forest and Woodland
Evergreen Forest	Inter-Mountain Basins Curl-leaf Mountain Mahogany Woodland and Shrubland
Evergreen Forest	North Pacific Broadleaf Landslide Forest and Shrubland
Evergreen Forest	North Pacific Wooded Volcanic Flowage
Grassland	California Annual Grassland
Grassland	California Central Valley and Southern Coastal Grassland
Grassland	California Mesic Serpentine Grassland
Grassland	California Northern Coastal Grassland
Grassland	Chihuahuan Sandy Plains Semi-Desert Grassland
Grassland	Columbia Basin Foothill and Canyon Dry Grassland
Grassland	Inter-Mountain Basins Semi-Desert Grassland
Grassland	Mediterranean California Alpine Dry Tundra
Grassland	Mediterranean California Subalpine Meadow
Grassland	North Pacific Montane Grassland
Grassland	Northern Rocky Mountain Lower Montane, Foothill and Valley Grassland
Grassland	Northern Rocky Mountain Subalpine-Upper Montane Grassland
Grassland	Northwestern Great Plains Mixedgrass Prairie
Grassland	Columbia Basin Palouse Prairie
Grassland	Rocky Mountain Alpine Fell-Field
Grassland	Rocky Mountain Alpine Turf
Grassland	Rocky Mountain Subalpine-Montane Mesic Meadow
Grassland	Southern Rocky Mountain Montane-Subalpine Grassland
Grassland	North Pacific Alpine and Subalpine Dry Grassland
Grassland	North Pacific Hypermaritime Shrub and Herbaceous Headland
Grassland	North Pacific Herbaceous Bald and Bluff
Grassland	Chihuahuan Loamy Plains Desert Grassland
Modified Land	Non-Specific Disturbed
Modified Land	Recently Burned
Modified Land	Recently Logged Areas
Modified Land	Recently Logged Timberland-Herbaceous Cover
Modified Land	Recently Logged Timberland-Shrubland Cover
Modified Land	Recently Logged Timberland-Woodland Cover
Modified Land	Recently Burned Herbaceous
Modified Land	Ruderal Upland - Old Field
Modified Land	Introduced Upland Vegetation - Treed
Modified Land	Introduced Upland Vegetation - Shrub
Modified Land	Introduced Upland Vegetation - Annual and Biennial Forbland
Modified Land	Introduced Upland Vegetation - Annual Grassland
Modified Land	Introduced Upland Vegetation - Perennial Grassland and Forbland
Modified Land	Introduced Riparian Vegetation
Modified Land	Introduced Wetland Vegetation
Modified Land	Recently Burned Forest and Woodland
Modified Land	Harvested forest-tree regeneration
Modified Land	Recently Logged Timberland
Savanna	California Central Valley Mixed Oak Savanna
Savanna	California Coastal Live Oak Woodland and Savanna
Savanna	California Lower Montane Blue Oak-Foothill Pine Woodland and Savanna
Savanna	Inter-Mountain Basins Juniper Savanna
Savanna	Madrean Juniper Savanna
Savanna	Southern Rocky Mountain Ponderosa Pine Savanna

Savanna	Southern California Oak Woodland and Savanna
Savanna	Willamette Valley Upland Prairie and Savanna
Savanna	Northern Rocky Mountain Foothill Conifer Wooded Steppe
Shrubland	Mediterranean California Mesic Serpentine Woodland and Chaparral
Shrubland	Colorado Plateau Mixed Low Sagebrush Shrubland
Shrubland	Columbia Plateau Scabland Shrubland
Shrubland	Inter-Mountain Basins Mat Saltbush Shrubland
Shrubland	Mediterranean California Alpine Fell-Field
Shrubland	North Pacific Dry and Mesic Alpine Dwarf-Shrubland, Fell-field and Meadow
Shrubland	Rocky Mountain Alpine Dwarf-Shrubland
Shrubland	Sierra Nevada Alpine Dwarf-Shrubland
Shrubland	Wyoming Basins Dwarf Sagebrush Shrubland and Steppe
Shrubland	Chihuahuan Creosotebush Desert Scrub
Shrubland	Chihuahuan Mixed Salt Desert Scrub
Shrubland	Chihuahuan Stabilized Coppice Dune and Sand Flat Scrub
Shrubland	Chihuahuan Succulent Desert Scrub
Shrubland	Colorado Plateau Blackbrush-Mormon-tea Shrubland
Shrubland	Great Basin Xeric Mixed Sagebrush Shrubland
Shrubland	Inter-Mountain Basins Big Sagebrush Shrubland
Shrubland	Inter-Mountain Basins Mixed Salt Desert Scrub
Shrubland	Mojave Mid-Elevation Mixed Desert Scrub
Shrubland	North Pacific Avalanche Chute Shrubland
Shrubland	North Pacific Montane Shrubland
Shrubland	Rocky Mountain Lower Montane-Foothill Shrubland
Shrubland	Sonora-Mojave Creosotebush-White Bursage Desert Scrub
Shrubland	Sonora-Mojave Mixed Salt Desert Scrub
Shrubland	Sonoran Mid-Elevation Desert Scrub
Shrubland	Southern California Coastal Scrub
Shrubland	Southern Colorado Plateau Sand Shrubland
Shrubland	Apacherian-Chihuahuan Mesquite Upland Scrub
Shrubland	California Maritime Chaparral
Shrubland	California Mesic Chaparral
Shrubland	California Montane Woodland and Chaparral
Shrubland	California Xeric Serpentine Chaparral
Shrubland	Chihuahuan Mixed Desert and Thorn Scrub
Shrubland	Madrean Oriental Chaparral
Shrubland	Colorado Plateau Pinyon-Juniper Shrubland
Shrubland	Great Basin Semi-Desert Chaparral
Shrubland	Mogollon Chaparral
Shrubland	Northern and Central California Dry-Mesic Chaparral
Shrubland	Northern Rocky Mountain Montane-Foothill Deciduous Shrubland
Shrubland	Rocky Mountain Gambel Oak-Mixed Montane Shrubland
Shrubland	Sonora-Mojave Semi-Desert Chaparral
Shrubland	Sonoran Paloverde-Mixed Cacti Desert Scrub
Shrubland	Southern California Dry-Mesic Chaparral
Shrubland	Northern Rocky Mountain Subalpine Deciduous Shrubland
Shrubland	Northern Rocky Mountain Avalanche Chute Shrubland
Shrubland	Klamath-Siskiyou Xeromorphic Serpentine Savanna and Chaparral
Shrubland	Apacherian-Chihuahuan Semi-Desert Grassland and Steppe
Shrubland	Columbia Plateau Steppe and Grassland
Shrubland	Columbia Plateau Low Sagebrush Steppe

Shrubland	Inter-Mountain Basins Big Sagebrush Steppe
Shrubland	Inter-Mountain Basins Montane Sagebrush Steppe
Shrubland	Inter-Mountain Basins Semi-Desert Shrub-Steppe
Shrubland	Northern California Coastal Scrub
Shrubland	North American Warm Desert Wash
Sparse Vegetation	Temperate Pacific Intertidal Flat
Sparse Vegetation	Temperate Pacific Freshwater Mudflat
Sparse Vegetation	North Pacific Volcanic Rock and Cinder Land
Sparse Vegetation	North Pacific Montane Massive Bedrock, Cliff and Talus
Sparse Vegetation	North Pacific Serpentine Barren
Sparse Vegetation	Mediterranean California Serpentine Barrens
Sparse Vegetation	Southern California Coast Ranges Cliff and Canyon
Sparse Vegetation	North Pacific Alpine and Subalpine Bedrock and Scree
Sparse Vegetation	North American Warm Desert Bedrock Cliff and Outcrop
Sparse Vegetation	North American Warm Desert Active and Stabilized Dune
Sparse Vegetation	North American Warm Desert Badland
Sparse Vegetation	Inter-Mountain Basins Volcanic Rock and Cinder Land
Sparse Vegetation	Rocky Mountain Cliff, Canyon and Massive Bedrock
Sparse Vegetation	Rocky Mountain Alpine Bedrock and Scree
Sparse Vegetation	Inter-Mountain Basins Shale Badland
Sparse Vegetation	North American Warm Desert Pavement
Sparse Vegetation	Inter-Mountain Basins Wash
Sparse Vegetation	Inter-Mountain Basins Active and Stabilized Dune
Sparse Vegetation	North American Warm Desert Playa
Sparse Vegetation	Central California Coast Ranges Cliff and Canyon
Sparse Vegetation	Klamath-Siskiyou Cliff and Outcrop
Sparse Vegetation	Sierra Nevada Cliff and Canyon
Sparse Vegetation	Mediterranean California Alpine Bedrock and Scree
Sparse Vegetation	Inter-Mountain Basins Cliff and Canyon
Sparse Vegetation	Columbia Plateau Ash and Tuff Badland
Sparse Vegetation	Inter-Mountain Basins Playa
Sparse Vegetation	North American Warm Desert Volcanic Rockland
Sparse Vegetation	Colorado Plateau Mixed Bedrock Canyon and Tableland
Unsuitable Habitat	Open Water
Unsuitable Habitat	Developed-Open Space
Unsuitable Habitat	Developed-Low Intensity
Unsuitable Habitat	Developed-Medium Intensity
Unsuitable Habitat	Developed-High Intensity
Unsuitable Habitat	North American Glacier and Ice Field
Wetland	North Pacific Lowland Mixed Hardwood Conifer Forest and Woodland
Wetland	California Central Valley Riparian Woodland and Shrubland
Wetland	Inter-Mountain Basins Greasewood Flat
Wetland	North Pacific Lowland Riparian Forest and Shrubland
Wetland	North Pacific Montane Riparian Woodland and Shrubland
Wetland	Northern Rocky Mountain Conifer Swamp
Wetland	Western Great Plains Floodplain
Wetland	Northern Rocky Mountain Lower Montane Riparian Woodland and Shrubland
Wetland	Rocky Mountain Lower Montane-Foothill Riparian Woodland and Shrubland
Wetland	North Pacific Bog and Fen
Wetland	Great Basin Foothill and Lower Montane Riparian Woodland and Shrubland
Wetland	Columbia Basin Foothill Riparian Woodland and Shrubland
Wetland	Rocky Mountain Subalpine-Montane Riparian Woodland
Wetland	North American Warm Desert Lower Montane Riparian Woodland and Shrubland
Wetland	North Pacific Shrub Swamp

Wetland	North American Warm Desert Riparian Mesquite Bosque
Wetland	North American Warm Desert Riparian Woodland and Shrubland
Wetland	Rocky Mountain Subalpine-Montane Riparian Shrubland
Wetland	Mediterranean California Alkali Marsh
Wetland	North Pacific Hardwood-Conifer Swamp
Wetland	Rocky Mountain Alpine-Montane Wet Meadow
Wetland	Western Great Plains Open Freshwater Depression Wetland
Wetland	Temperate Pacific Freshwater Aquatic Bed
Wetland	North Pacific Intertidal Freshwater Wetland
Wetland	Willamette Valley Wet Prairie
Wetland	North American Arid West Emergent Marsh
Wetland	North Pacific Maritime Eelgrass Bed
Wetland	Columbia Plateau Vernal Pool
Wetland	Rocky Mountain Subalpine-Montane Fen
Wetland	Mediterranean California Subalpine-Montane Fen
Wetland	Northern California Claypan Vernal Pool
Wetland	Mediterranean California Serpentine Fen
Wetland	Western Great Plains Saline Depression Wetland
Wetland	Temperate Pacific Freshwater Emergent Marsh
Wetland	Temperate Pacific Subalpine-Montane Wet Meadow
Wetland	Temperate Pacific Tidal Salt and Brackish Marsh
Wetland	Inter-Mountain Basins Alkaline Closed Depression
Wetland	Columbia Plateau Silver Sagebrush Seasonally Flooded Shrub-Steppe
Wetland	Mediterranean California Serpentine Foothill and Lower Montane Riparian Woodland and Seep
Wetland	Northwestern Great Plains Riparian
Wetland	Western Great Plains Riparian
Wetland	Mediterranean California Foothill and Lower Montane Riparian Woodland
Wetland	Chihuahuan-Sonoran Desert Bottomland and Swale Grassland
