# Supplementary material for: Resource Selection by the California Condor (Gymnogyps californianus) Relative to Terrestrial-Based Habitats and Meteorological Conditions
Source: PLoS One. 2014 Feb 11;9(2):e88430. doi: 10.1371/journal.pone.0088430 (PMC3921182; doi:10.1371/journal.pone.0088430)
Supplement: Document S2 — Plots of marginal mean ln (rf) by habitat for sex effects, age and age class effects, release site, rearing method, and breeding status. (PDF) [file pone.0088430.s002.pdf]

Document S2. Plots of marginal mean ln(rf) by habitat type for sex effects over the course of the study (pp. 1-2), sex effects relative to condor age (pp. 3-4), age class (1= juvenile, 2 = adult) (pp. 5-6), release site (FWS = US Fish and Wildlife Service, PNM = Pinnacles National Monument, VWS = Ventana Wildlife Society) (pp. 7-8), rearing method (0 = captive reared, 1 = raised in wild) (pp. 9-10, and breeding status (0 = non-breeder, 1 = breeder) (pp. 11-12). Habitat codes are as follows: ag = agriculture, c_d = coast (dune), c_r = coast (rock), d_f = deciduous forest, e_g = evergreen forest, g = grassland, m_l = modified land, sav = savanna, shr = shrubland, spa = sparse vegetation, un = unsuitable habitat, and wet = wetland.

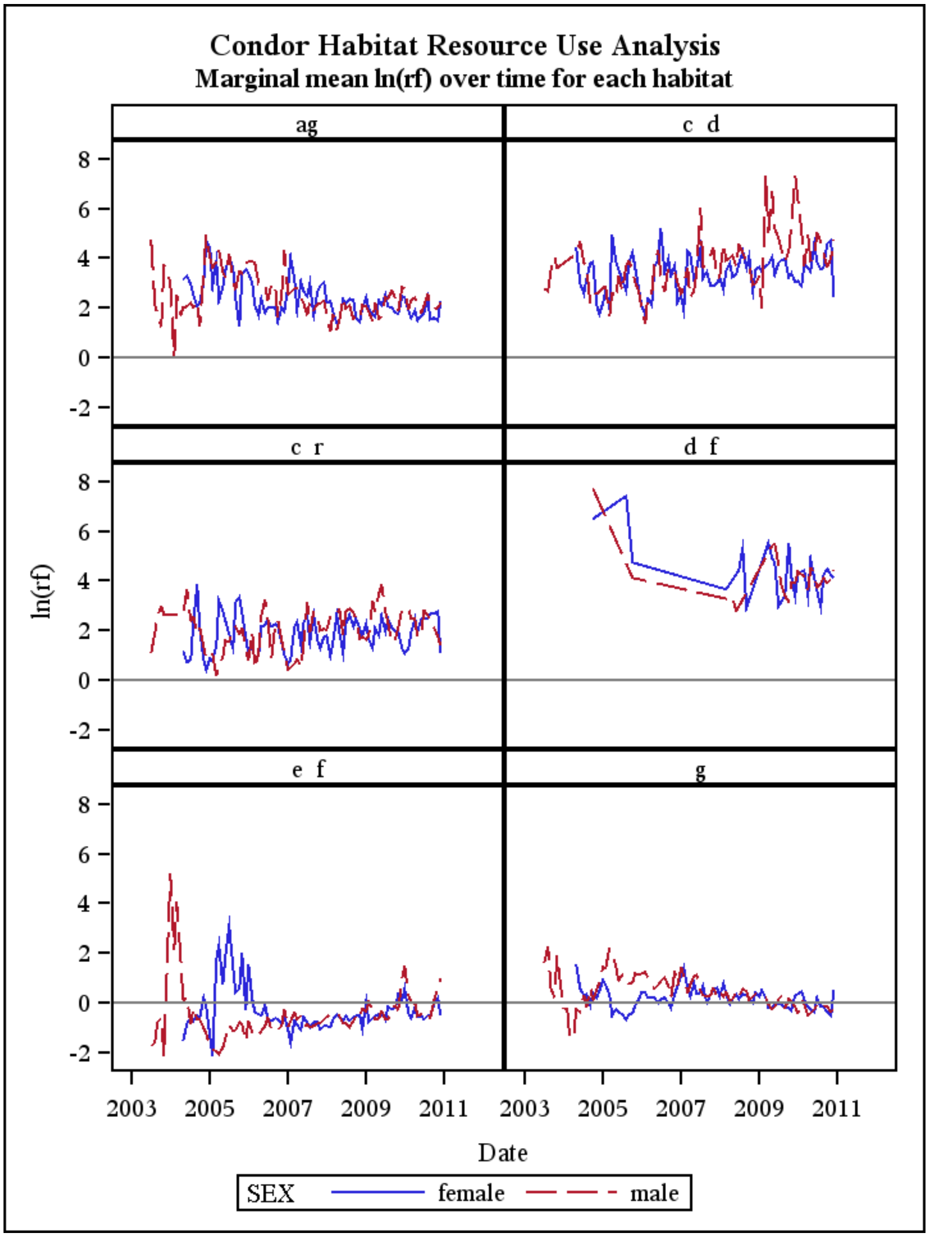
Condor Habitat Resource Use Analysis
Marginal mean ln(rf) over time for each habitat
ag
c d
c r
d f
e f
g
ln(rf)
8
6
4
2
0
-2
2003
2005
2007
2009
2011
Date
SEX
female
male

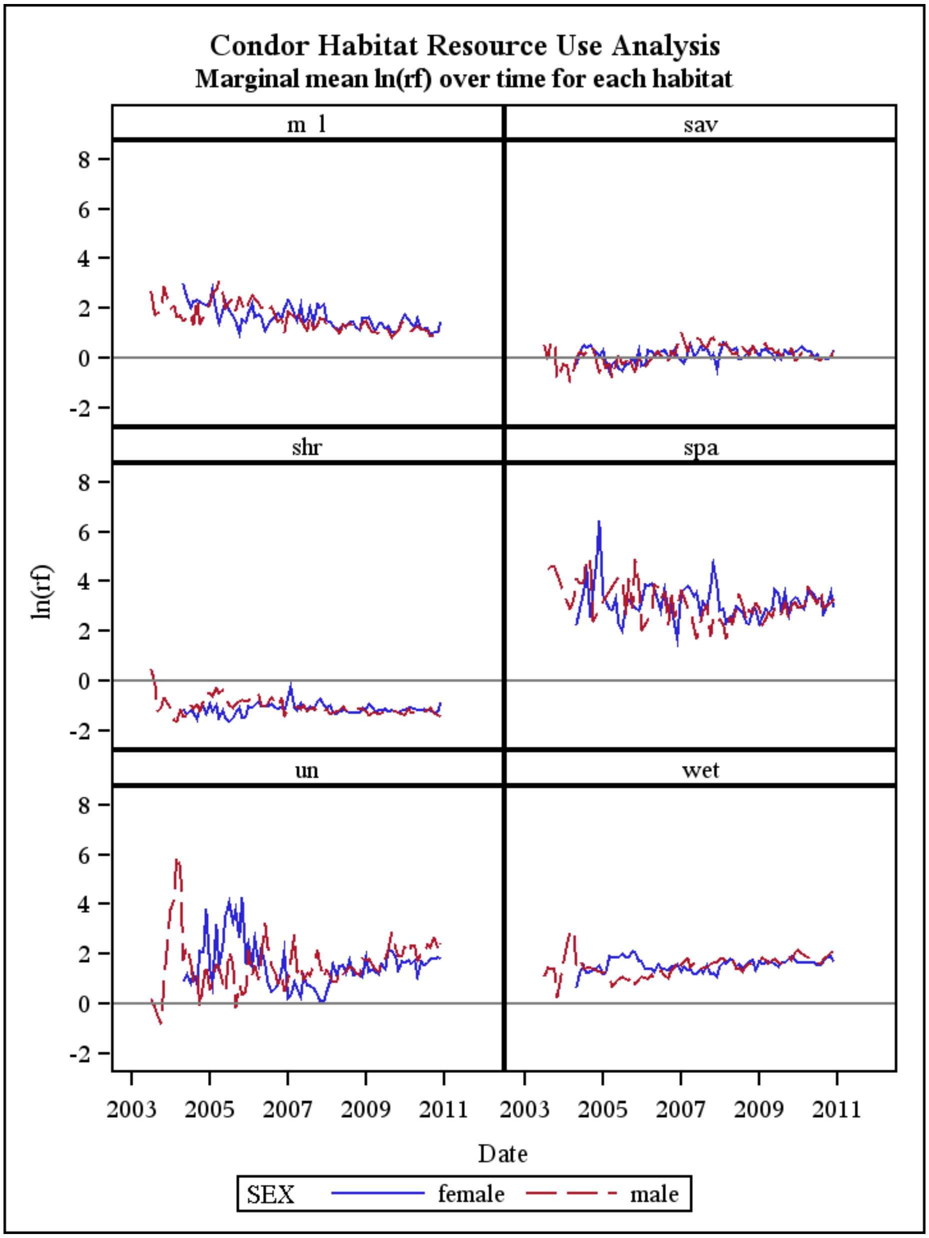
Condor Habitat Resource Use Analysis
Marginal mean ln(rf) over time for each habitat
m 1
sav
shr
spa
un
wet
ln(rf)
8
6
4
2
0
-2
2003
2005
2007
2009
2011
Date
SEX
female
male

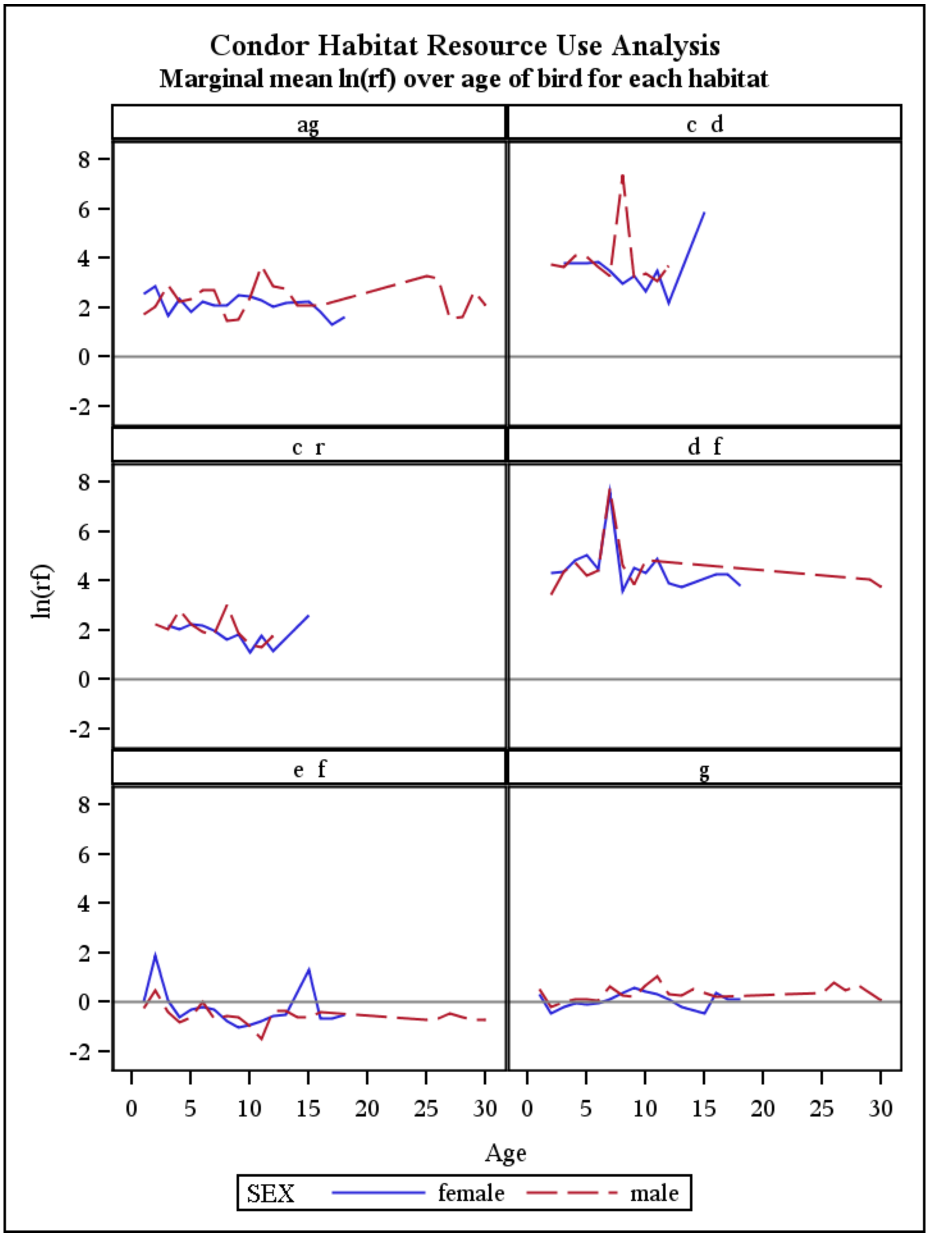
Condor Habitat Resource Use Analysis
Marginal mean ln(rf) over age of bird for each habitat
ag
c d
c r
d f
e f
g
ln(rf)
8
6
4
2
0
-2
0 5 10 15 20 25 30
Age
SEX female male

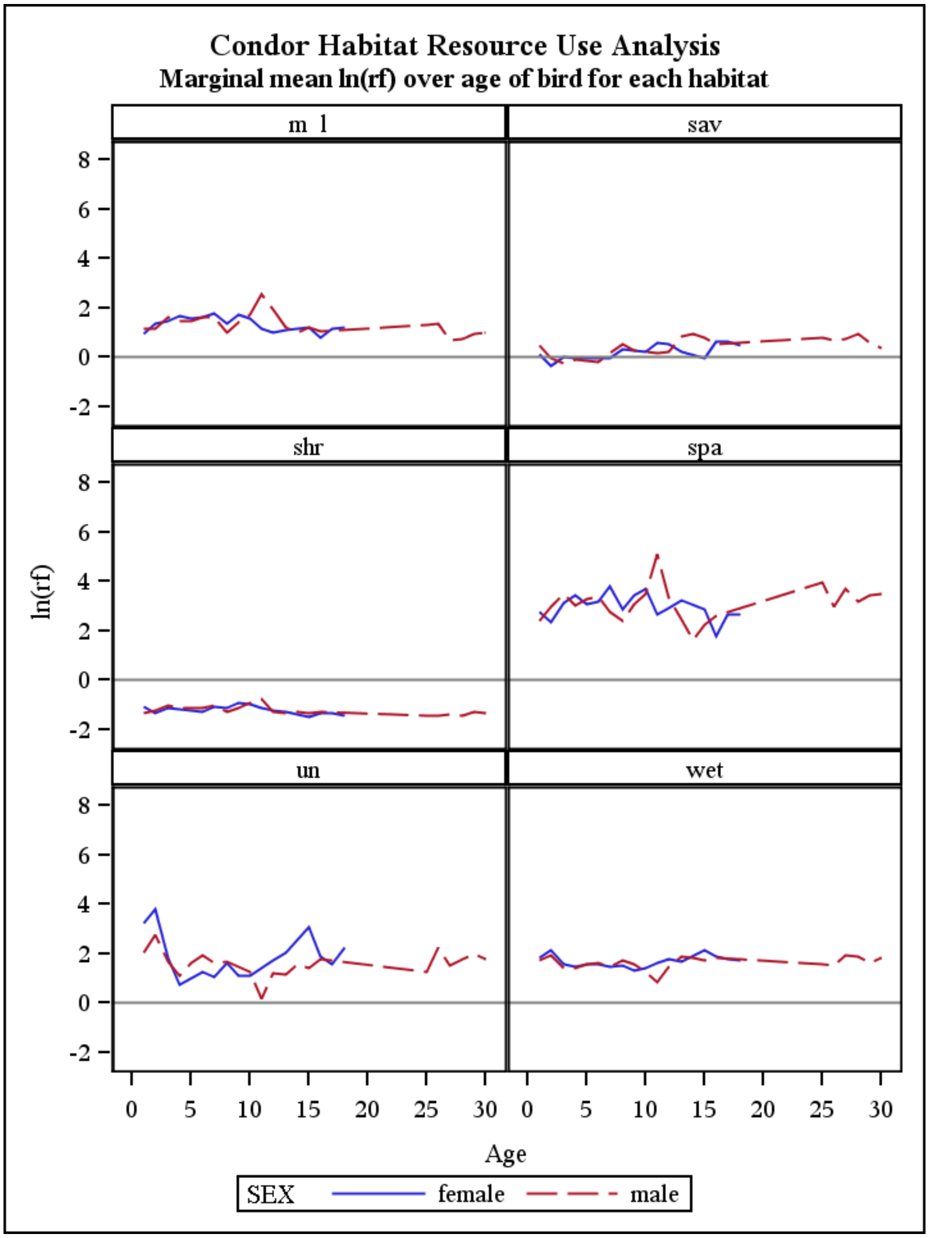
Condor Habitat Resource Use Analysis
Marginal mean ln(rf) over age of bird for each habitat
m 1
sav
shr
spa
un
wet
ln(rf)
8
6
4
2
0
-2
0
5
10
15
20
25
30
Age
SEX
female
male

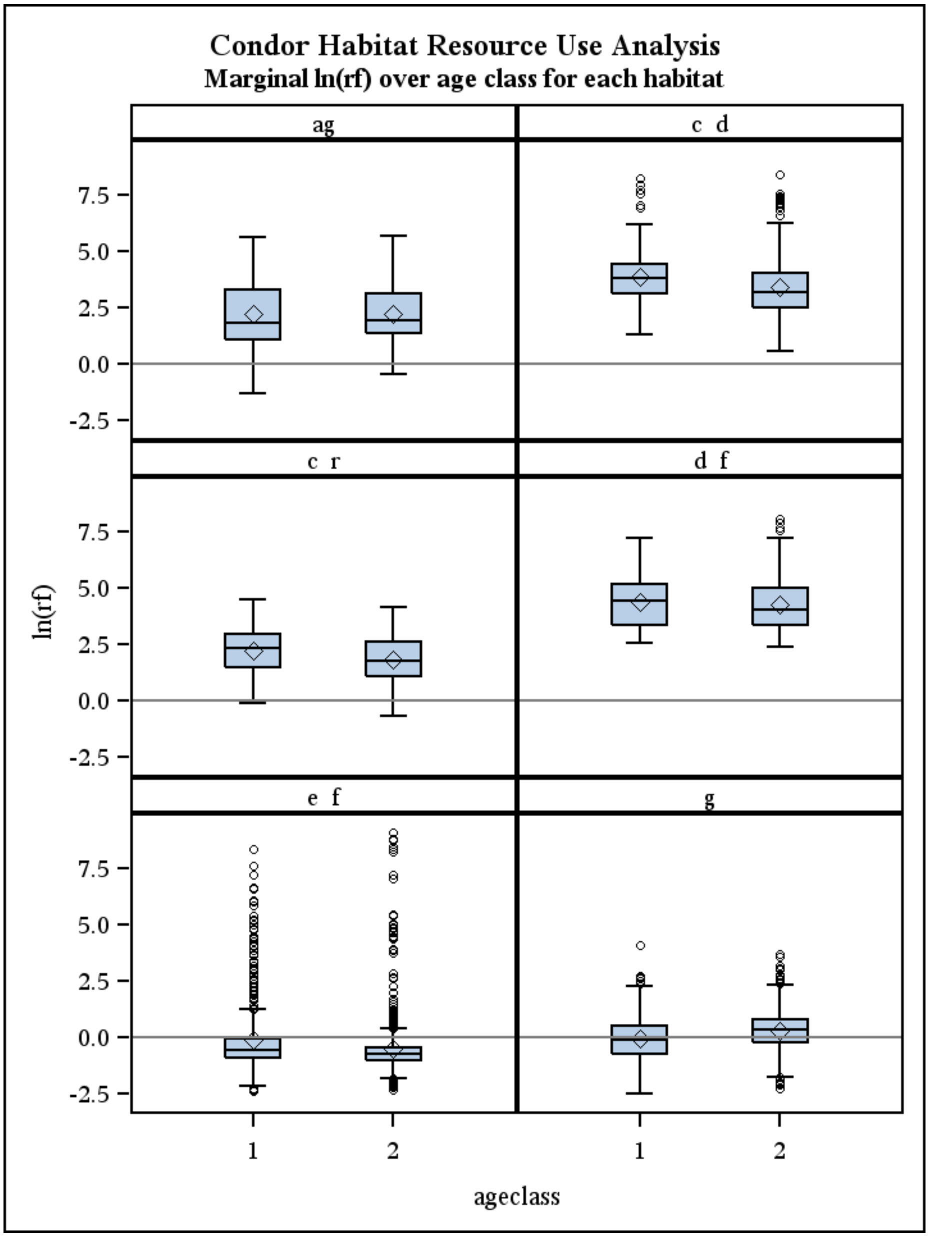
Condor Habitat Resource Use Analysis
Marginal ln(rf) over age class for each habitat
ag
c d
c r
d f
e f
g
ln(rf)
7.5
5.0
2.5
0.0
-2.5
1
2
ageclass

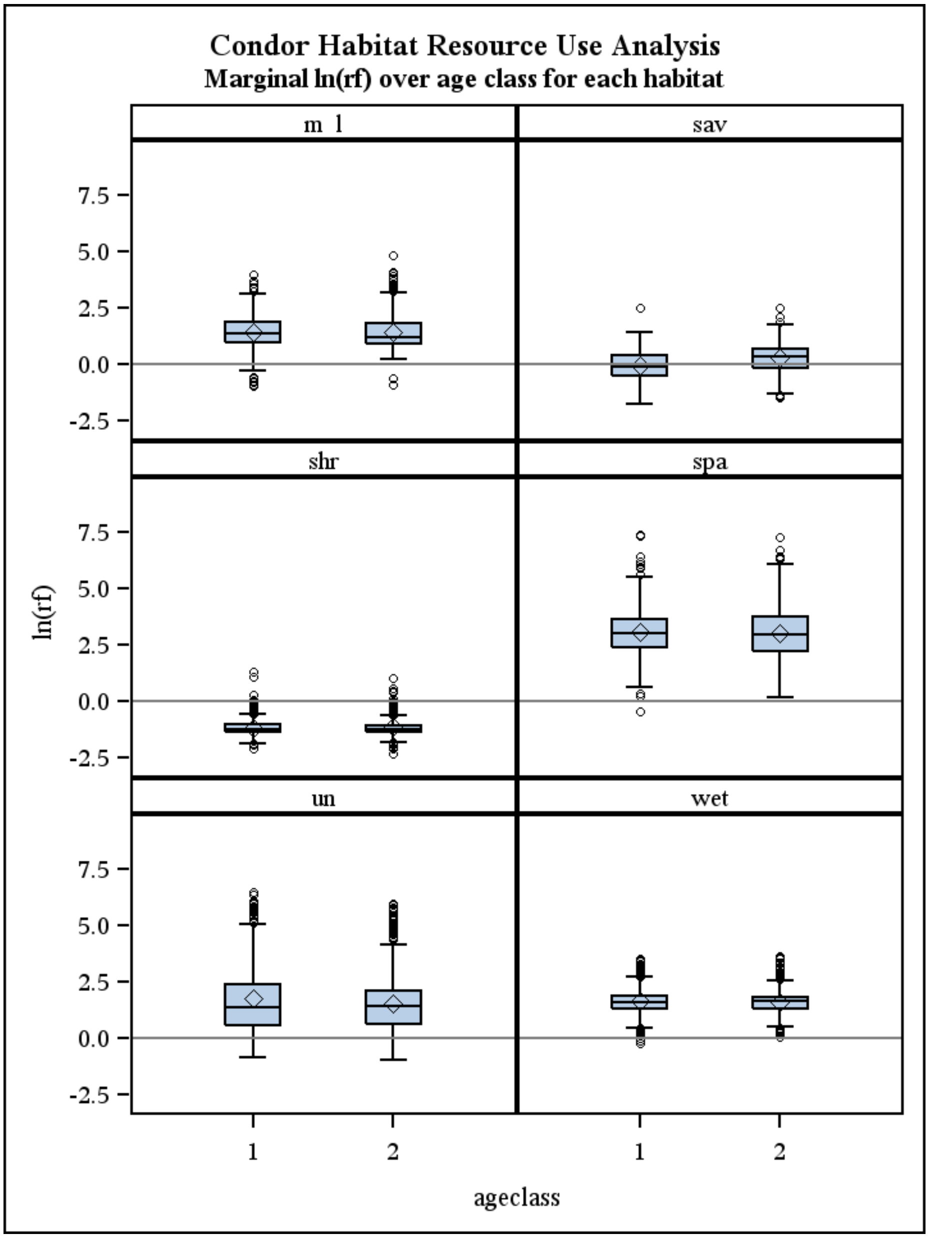
Condor Habitat Resource Use Analysis
Marginal ln(rf) over age class for each habitat
m_l
sav
shr
spa
un
wet
ln(rf)
7.5
5.0
2.5
0.0
-2.5
1
2
ageclass

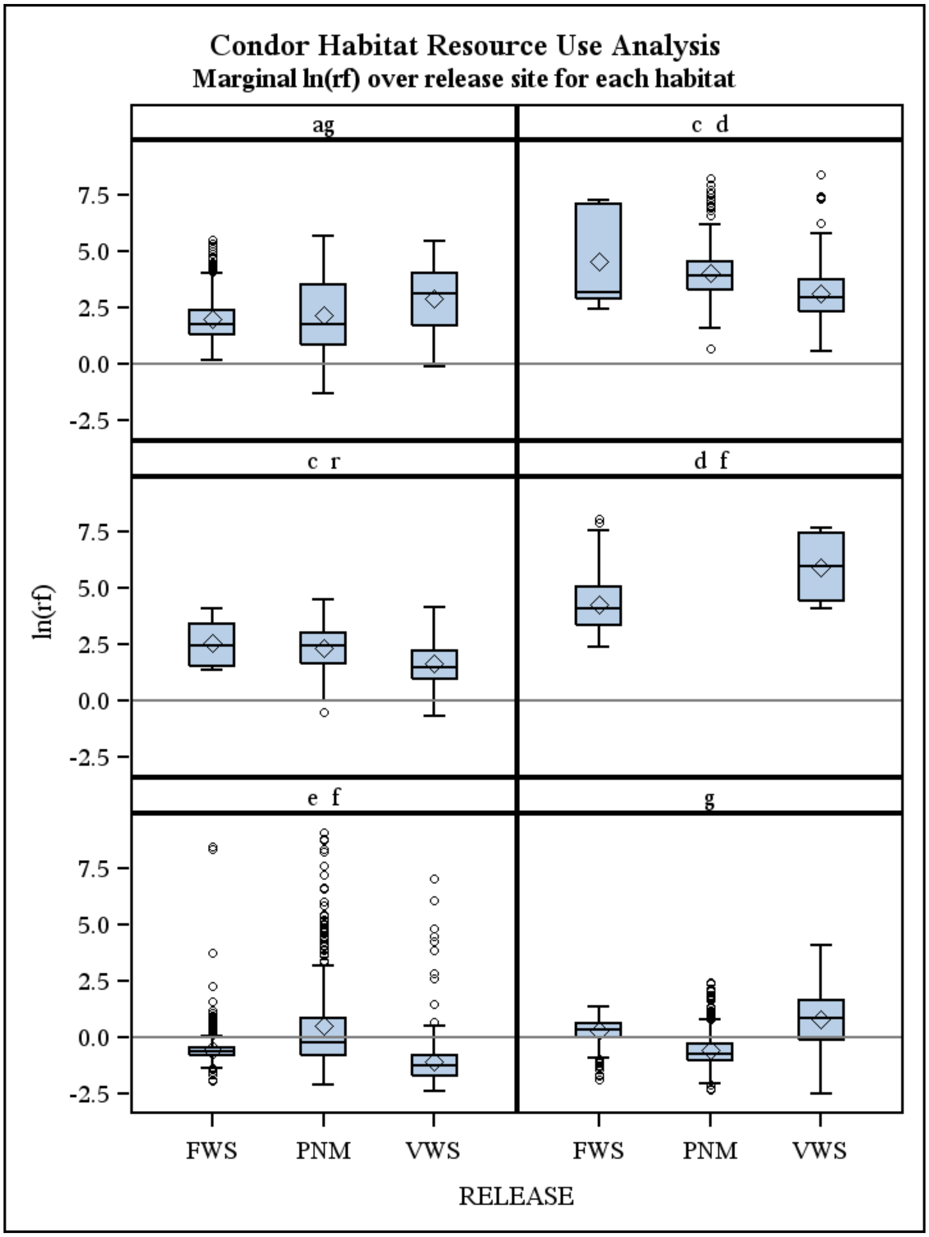

Condor Habitat Resource Use Analysis
Marginal ln(rf) over release site for each habitat
ag
c d
c r
d f
e f
g
7.5
5.0
2.5
0.0
-2.5
ln(rf)
FWS
PNM
VWS
RELEASE

Condor Habitat Resource Use Analysis
Marginal ln(rf) over release site for each habitat

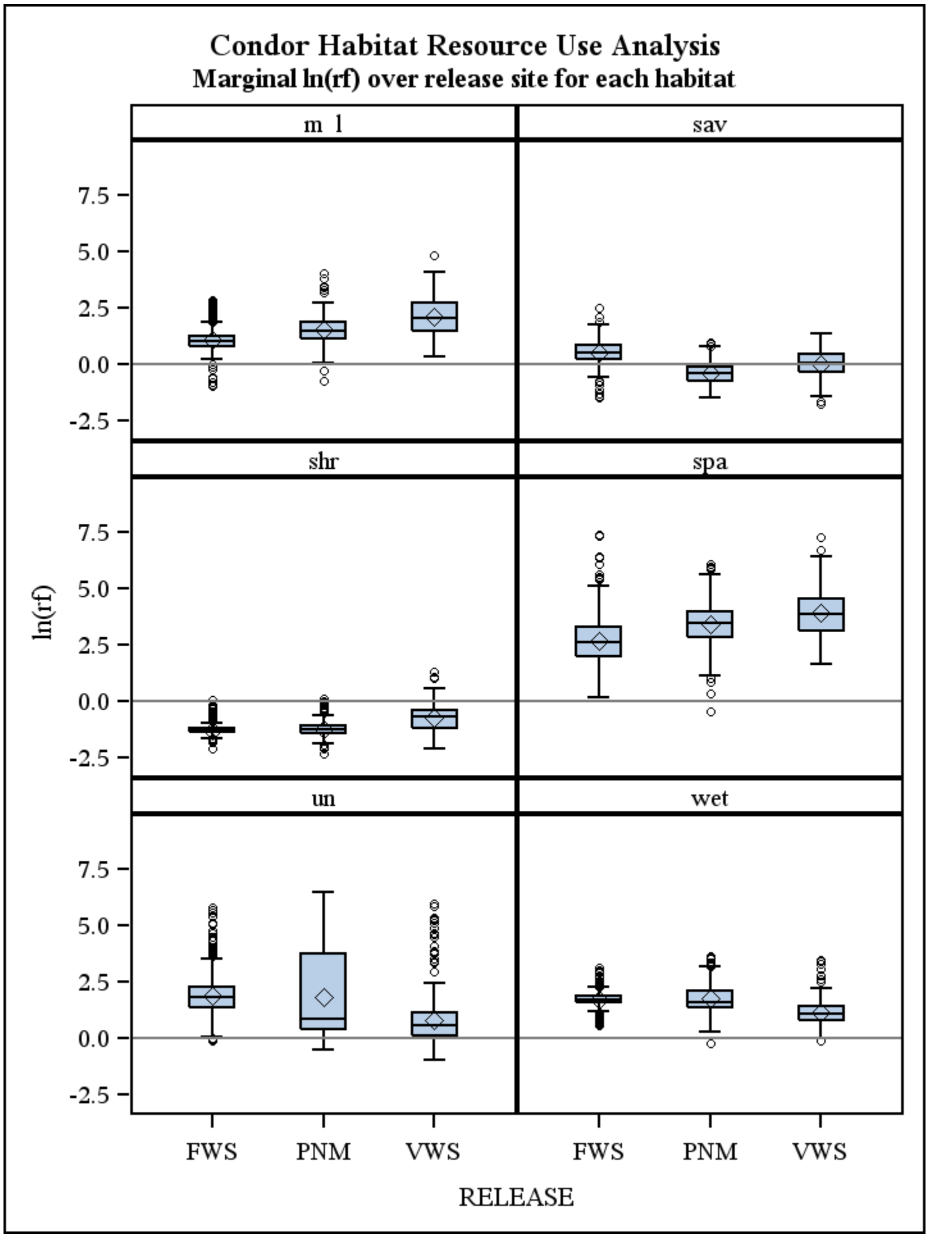

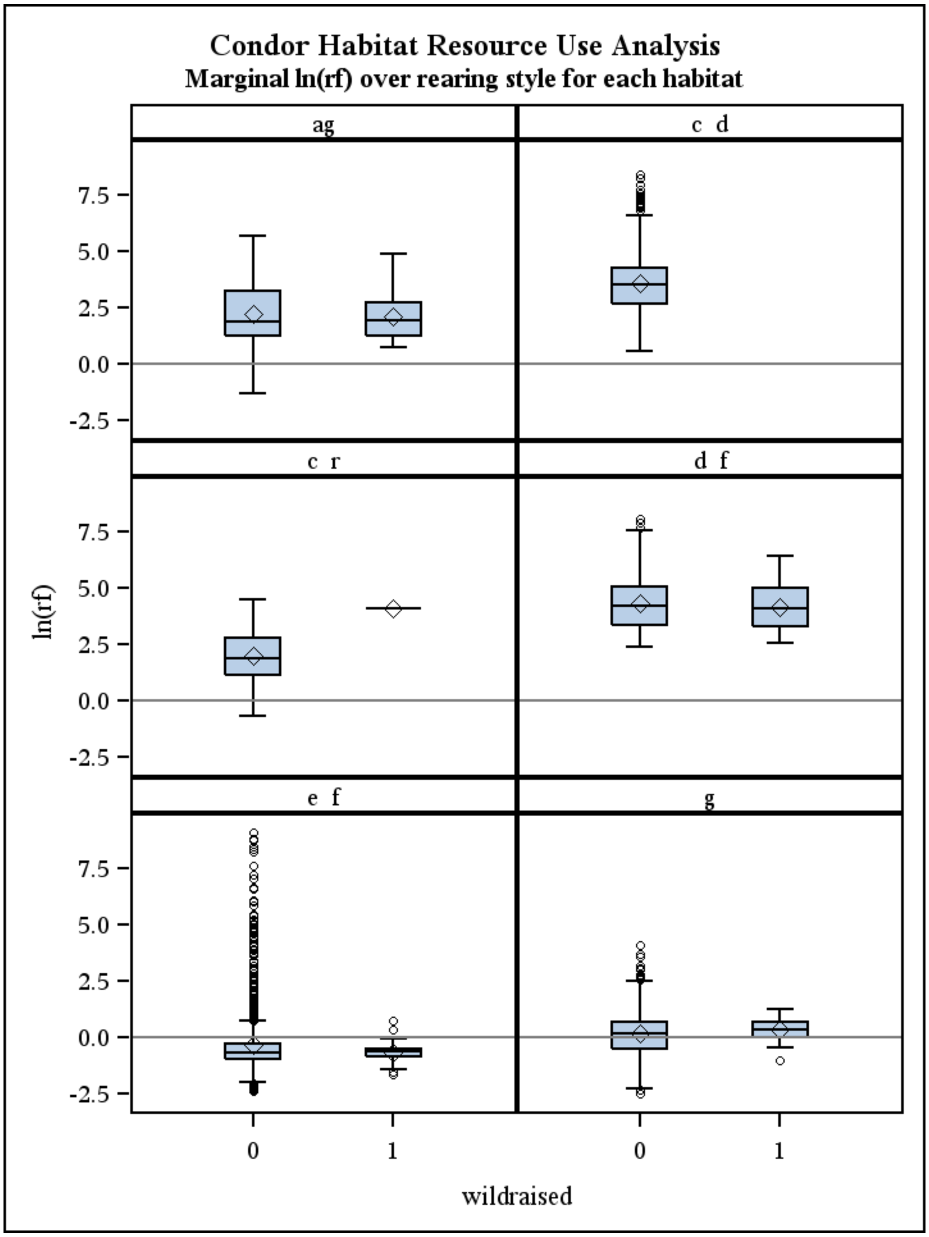

Condor Habitat Resource Use Analysis
Marginal ln(rf) over rearing style for each habitat
ag
c d
c r
d f
e f
g
7.5
5.0
2.5
0.0
-2.5
ln(rf)
0
1
wildraised

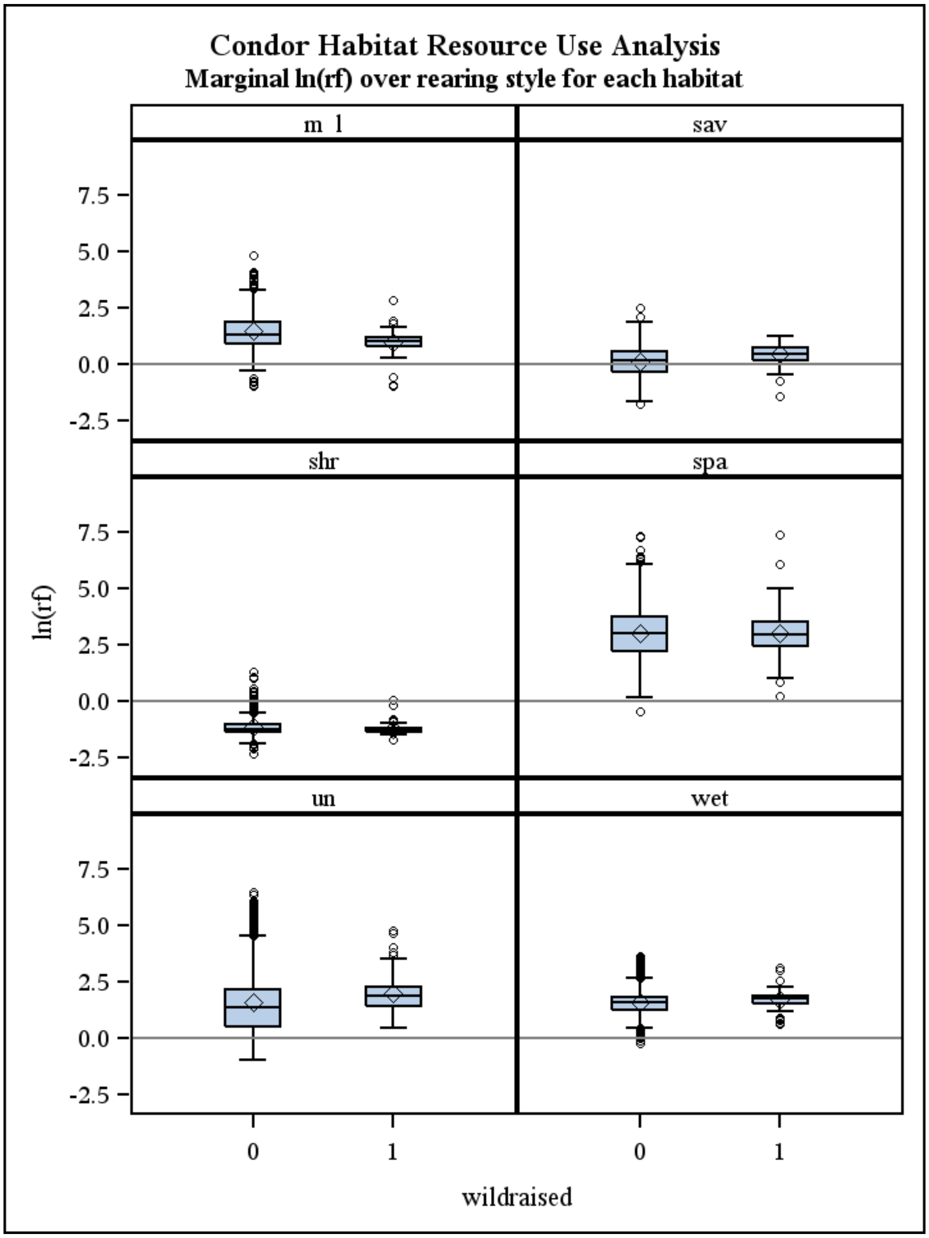
Condor Habitat Resource Use Analysis
Marginal ln(rf) over rearing style for each habitat
m 1
sav
shr
spa
un
wet
ln(rf)
7.5
5.0
2.5
0.0
-2.5
0
1
wildraised

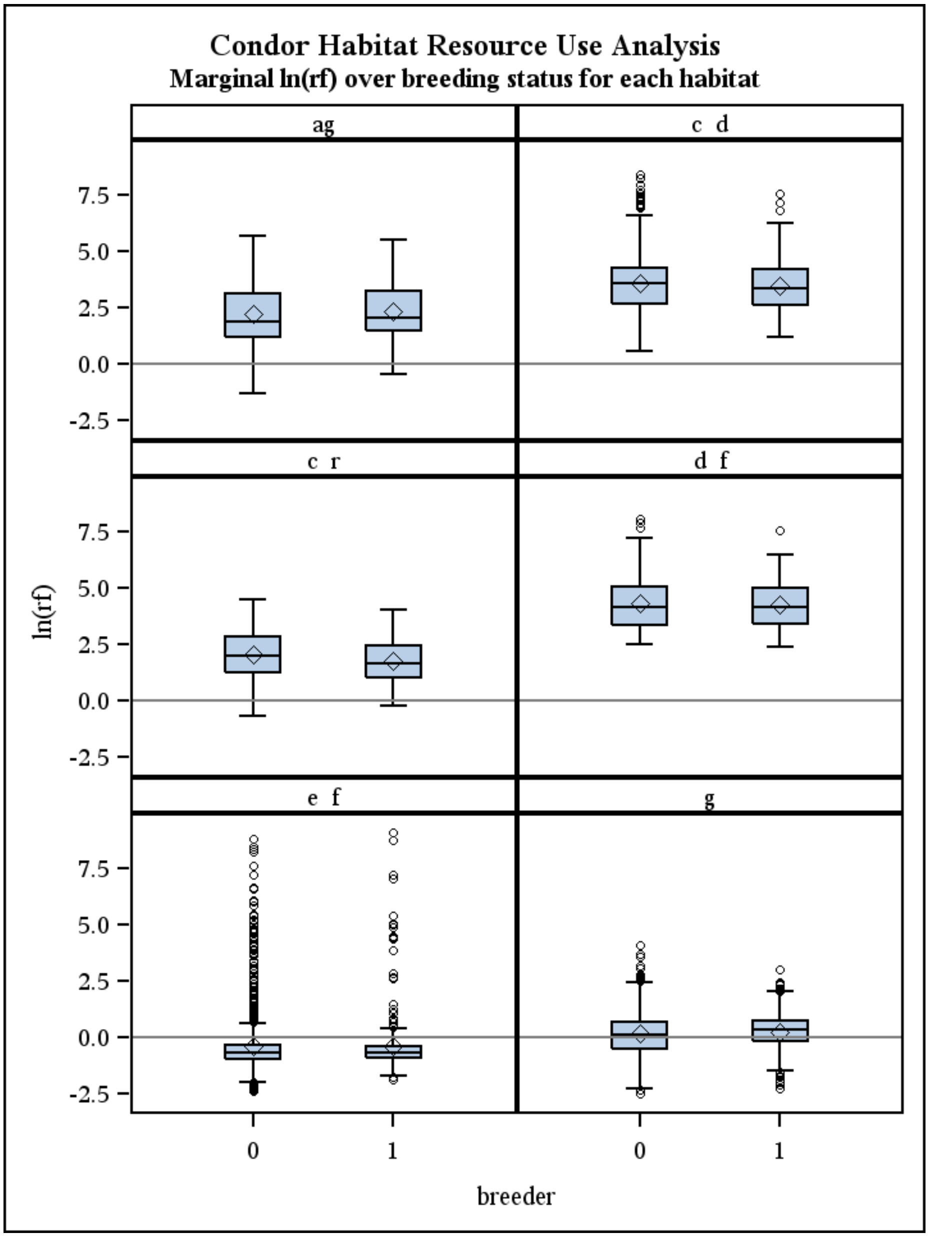

Condor Habitat Resource Use Analysis
Marginal ln(rf) over breeding status for each habitat
ag
c d
c r
d f
e f
g
ln(rf)
7.5
5.0
2.5
0.0
-2.5
0
1
breeder

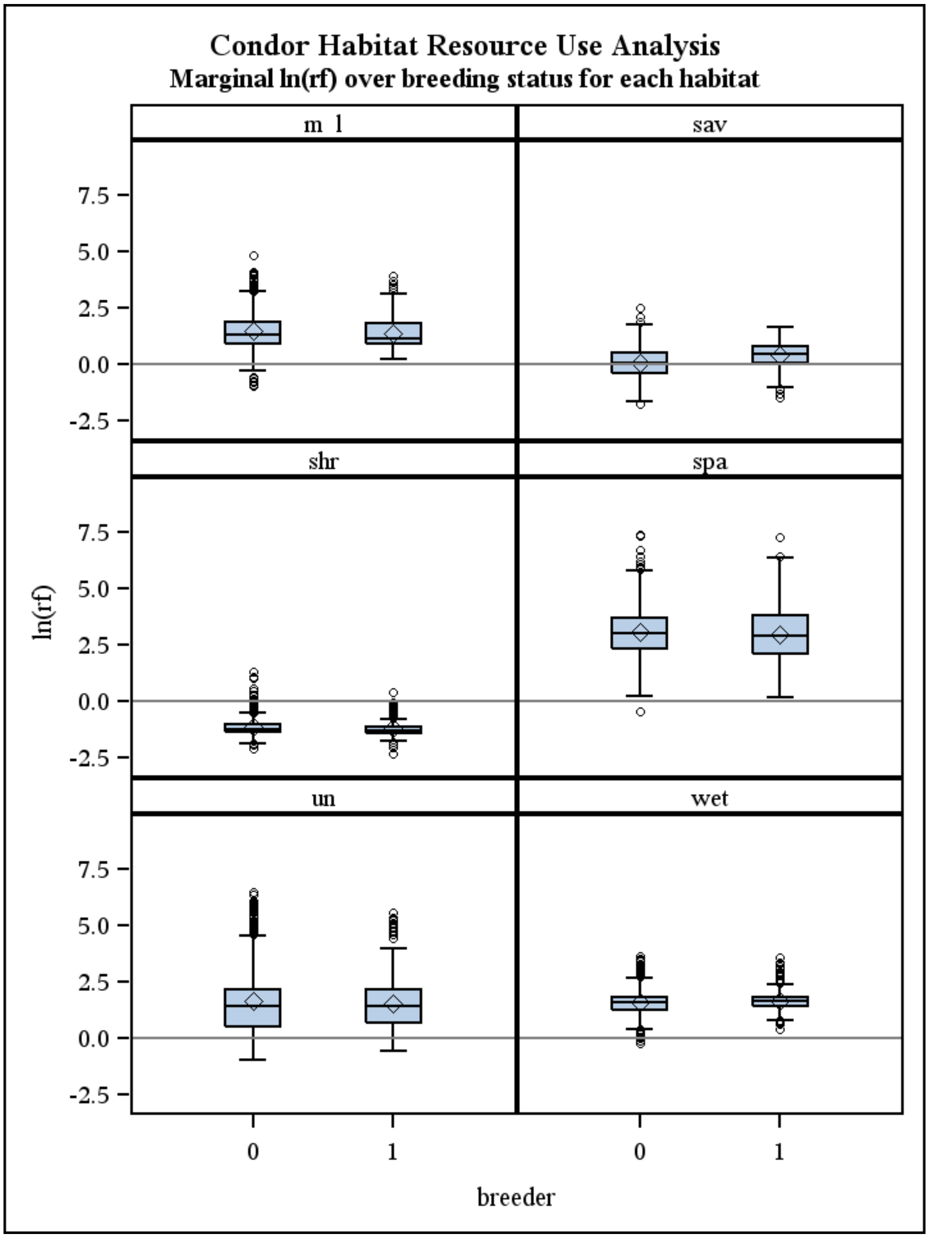
Condor Habitat Resource Use Analysis
Marginal ln(rf) over breeding status for each habitat
m 1
sav
shr
spa
un
wet
ln(rf)
7.5
5.0
2.5
0.0
-2.5
0
1
breeder
