# Supplementary material for: Resource Selection by the California Condor (Gymnogyps californianus) Relative to Terrestrial-Based Habitats and Meteorological Conditions
Source: PLoS One. 2014 Feb 11;9(2):e88430. doi: 10.1371/journal.pone.0088430 (PMC3921182; doi:10.1371/journal.pone.0088430)
Supplement: Document S3 — Ecoregions as delineated by [11] and used to assess California Condor resource selection relative to meteorological parameters. (PDF) [file pone.0088430.s003.pdf]

Document S3. Ecoregions in the state of California as delineated by Cleland et al. (2007) and used to assess California Condor resource selection relative to atmospheric parameters. Codes correspond with the numbered ecoregions in Document S3.

Ecoregion	Code
Watsonville Plain-Salinas Valley	8
North Coastal Santa Lucia Range	9
South Coastal Santa Lucia Range	10
Santa Ynez-Sulphur Mountains	13
Oxnard Plain-Santa Paula Valley	15
Simi Valley-Santa Susana Mountains	16
Los Angeles Plain	18
South Valley Alluvium and Basins	39
Elk Hills and South Valley Terraces	40
Tehachapi-Piute Mountains	95
Southern Granitic Foothills	101
San Emigdio Mountains	102
Diablo Range	116
Eastern Hills	117
Interior Santa Lucia Range	118
Gabilan Range	119
Caliente Range-Cuyama Valley	123
Temblor Range	124
San Rafael-Topatopa Mountains	125
Northern Transverse Ranges	126
Sierra Pelona-Mint Canyon	127
San Gabriel Mountains	128
High Desert Plains and Hills	147
Water	192
Eastern Slopes	193
