# Supplementary material for: Resource Selection by the California Condor (Gymnogyps californianus) Relative to Terrestrial-Based Habitats and Meteorological Conditions
Source: PLoS One. 2014 Feb 11;9(2):e88430. doi: 10.1371/journal.pone.0088430 (PMC3921182; doi:10.1371/journal.pone.0088430)
Supplement: Document S4 — Map of 25 ecoregions in which California Condors were observed in reasonable numbers to quantify meteorological parameters in the study. (PDF) [file pone.0088430.s004.pdf]

Document S4. Map of 25 ecoregions in which California Condors were observed in reasonable numbers to quantify meteorological parameters in the study. Numbers correspond to ecoregions listed in Table 1, and the white polygon outlines the historic California Condor range based on USFWS (1996). Abbreviations: VWS = Ventana Wildlife Society, PNM = Pinnacles National Monument, BCNWR = Bitter Creek National Wildlife Refuge, HMNWR = Hopper Mountain National Wildlife Refuge.

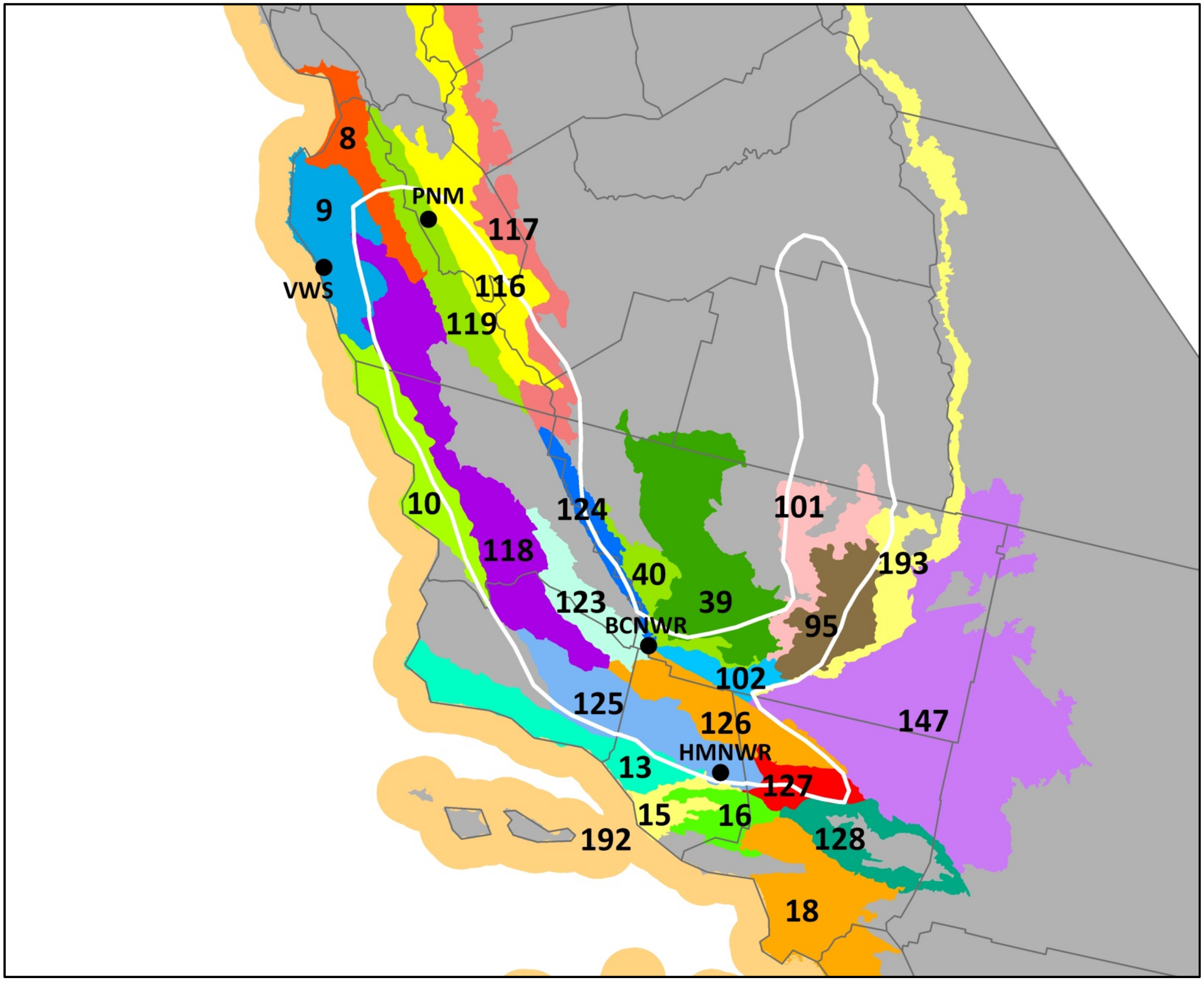

8
9
PNM
117
VWS
116
119
124
101
10
118
40
193
123
39
95
BCNWR
102
147
125
126
HMNWR
13
127
15
16
128
192
18
