# Supplementary material for: Resource Selection by the California Condor (Gymnogyps californianus) Relative to Terrestrial-Based Habitats and Meteorological Conditions
Source: PLoS One. 2014 Feb 11;9(2):e88430. doi: 10.1371/journal.pone.0088430 (PMC3921182; doi:10.1371/journal.pone.0088430)
Supplement: Document S5 — Summary of the number of ecoregion models and their parameters that had ΔAIC≤3 from candidate models. (PDF) [file pone.0088430.s005.pdf]

Document S5. Summary of the number of ecoregion models and their parameters that had $\Delta AIC \leq 3$ from candidate models.

Model	count
thermal height + thermal velocity + age + rearing style + breeding status + release site + date	9
thermal height + wind speed + rearing style + breeding status + release site + date	8
thermal height + thermal velocity + rearing style + breeding status + release site	7
thermal height + wind speed + age + rearing style + breeding status + release site + date	7
thermal height + thermal velocity + rearing style + breeding status + release site + date	6
thermal height + rearing style + breeding status + release site	6
thermal height + rearing style + breeding status + release site + date	6
thermal height + wind speed + rearing style + breeding status + release site	6
month + breeding status + release site + date	5
month + release site + date	5
thermal velocity + wind speed + age + rearing style + breeding status + release site + date	5
month + release site	3
thermal height + thermal velocity + wind speed + age + rearing style + breeding status + release site	3
thermal height + rearing style + age + breeding status + release site + date	3
date	2
month + date	2
month + rearing style + breeding status + release site + date	2
thermal height + thermal velocity + wind speed + rearing style + breeding status + release site	2
thermal velocity + rearing style + breeding status + release site	2
thermal velocity + rearing style + breeding status + release site + date	2
thermal velocity + wind speed + rearing style + breeding status + release site	2
thermal velocity + wind speed + rearing style + breeding status + release site + date	2
wind speed + rearing style + breeding status + release site	2
wind speed + rearing style + breeding status + release site + date	2

month	1
month + breeding status + release site	1
month + rearing style + breeding status + release site	1
wind speed + rearing style + age + breeding status + release site + date	1
