# Supplementary material for: Resource Selection by the California Condor (Gymnogyps californianus) Relative to Terrestrial-Based Habitats and Meteorological Conditions
Source: PLoS One. 2014 Feb 11;9(2):e88430. doi: 10.1371/journal.pone.0088430 (PMC3921182; doi:10.1371/journal.pone.0088430)

Document S6. The following figures contain plots for each of three meteorological parameters (i.e., thermal height, thermal velocity, and wind speed; left axis) and raw ln(rf) values (right axis) plotted against months in the annual cycle for each of the 25 California ecoregions examined in the study. LOESS smoothers are plotted for both sets of data.

EcoRegion=8

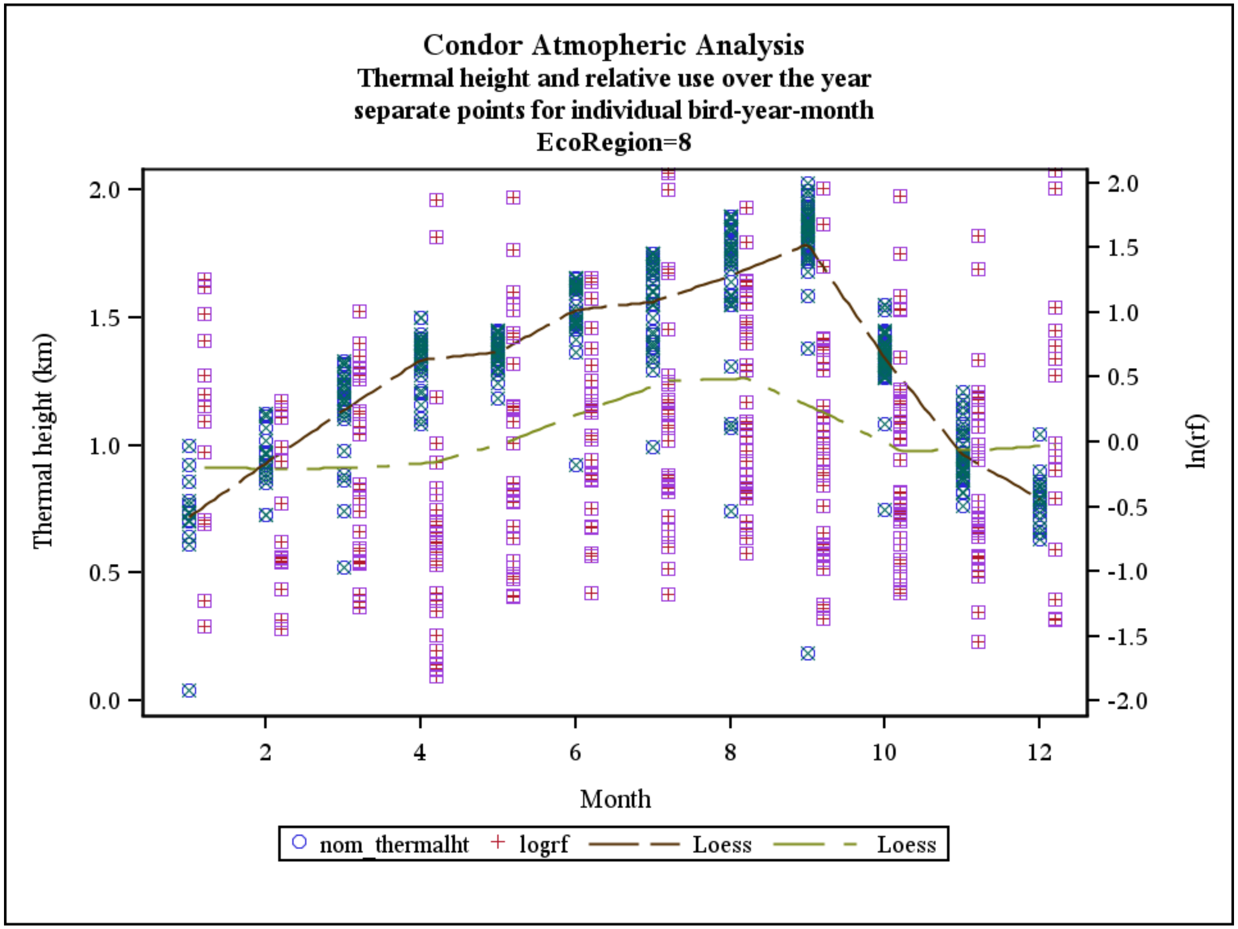

Condor Atmopheric Analysis
Thermal height and relative use over the year
separate points for individual bird-year-month
EcoRegion=8
Thermal height (km)
2.0
1.5
1.0
0.5
0.0
2
4
6
8
10
12
Month
ln(rf)
2.0
1.5
1.0
0.5
0.0
-0.5
-1.0
-1.5
-2.0
nom_thermalht
logrf
Loess
Loess

EcoRegion=9

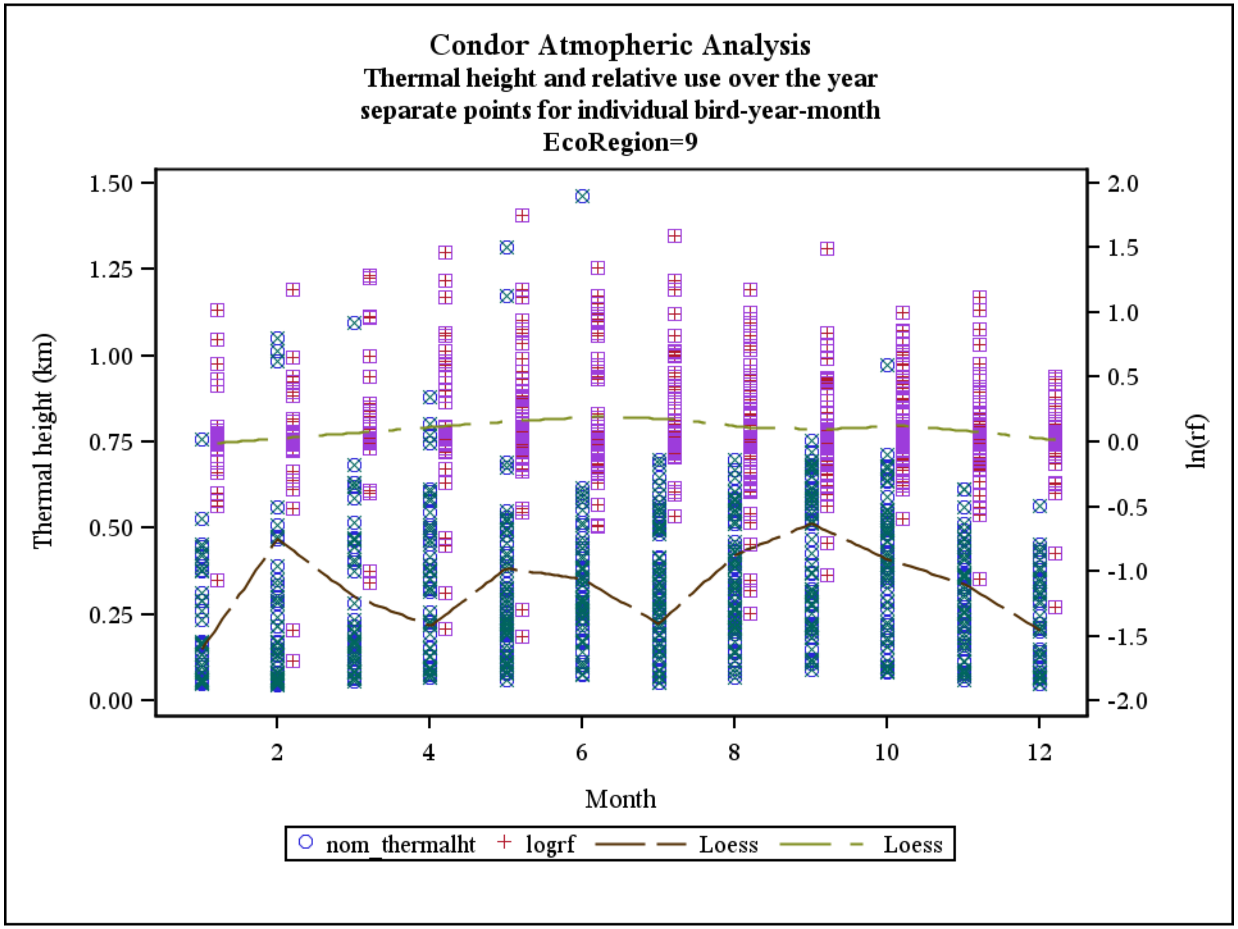
Condor Atmopheric Analysis
Thermal height and relative use over the year
separate points for individual bird-year-month
EcoRegion=9
Thermal height (km)
1.50
1.25
1.00
0.75
0.50
0.25
0.00
ln(rf)
2.0
1.5
1.0
0.5
0.0
-0.5
-1.0
-1.5
-2.0
2
4
6
8
10
12
Month
nom_thermalht
logrf
Loess
Loess

EcoRegion=10

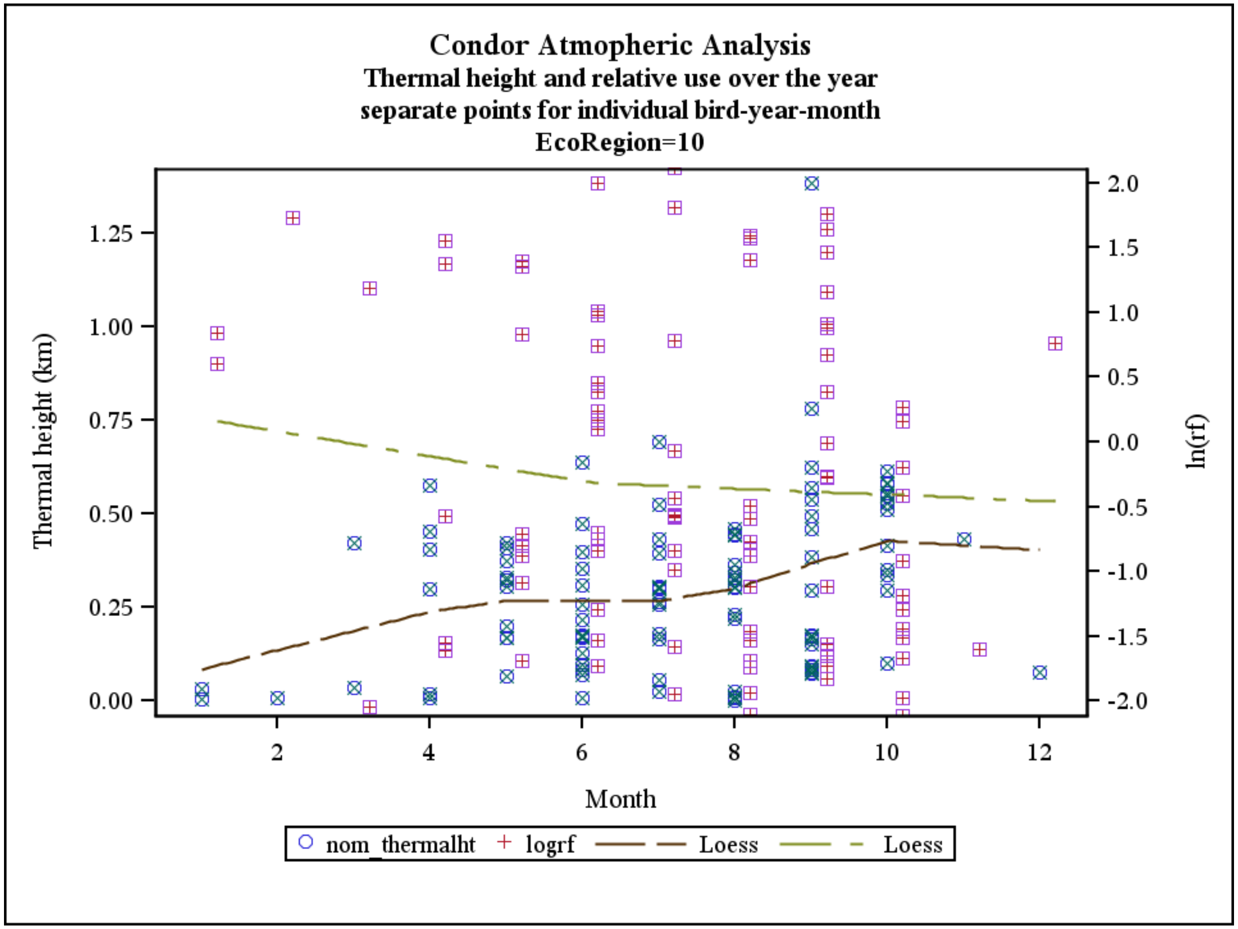

EcoRegion=13

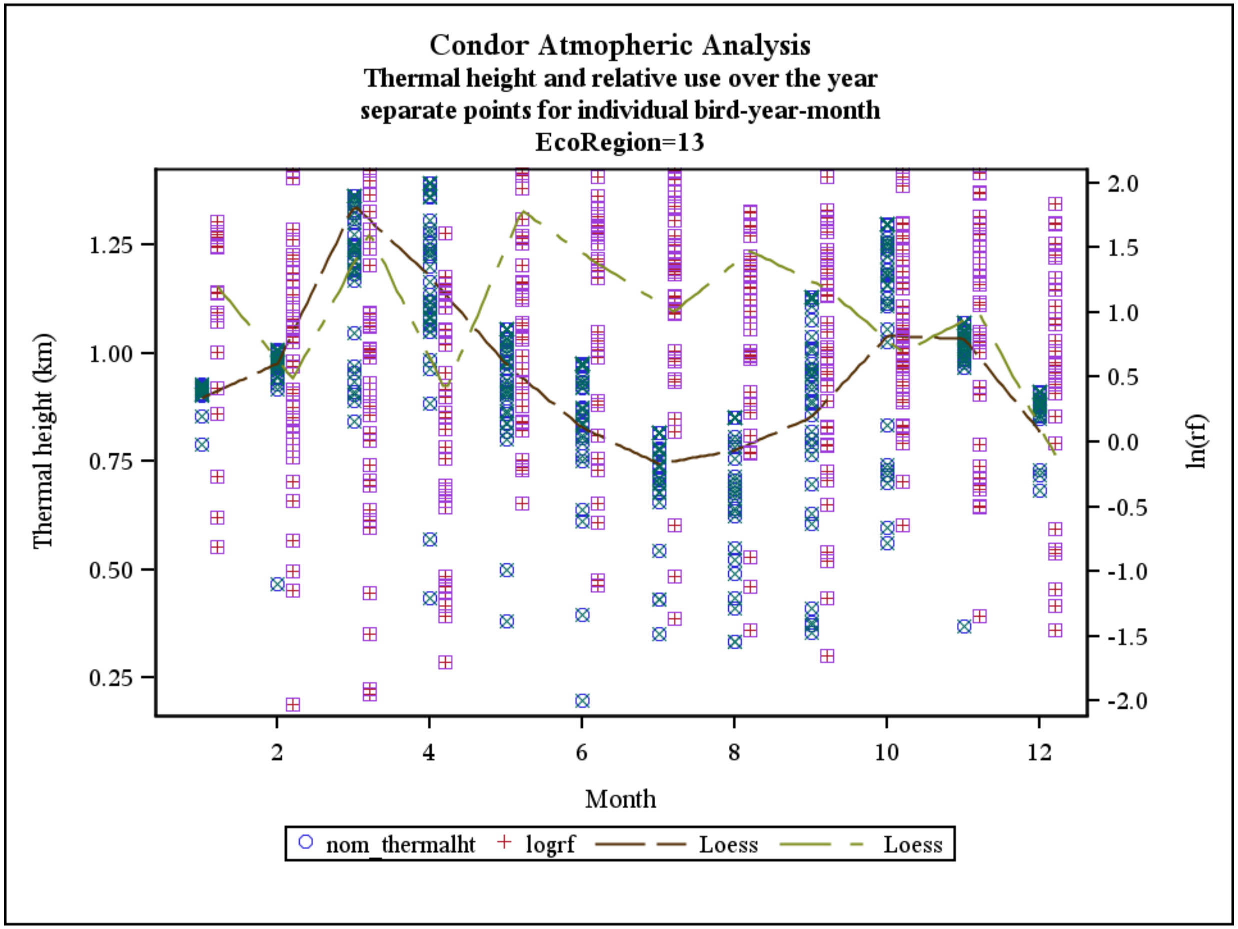

EcoRegion=15

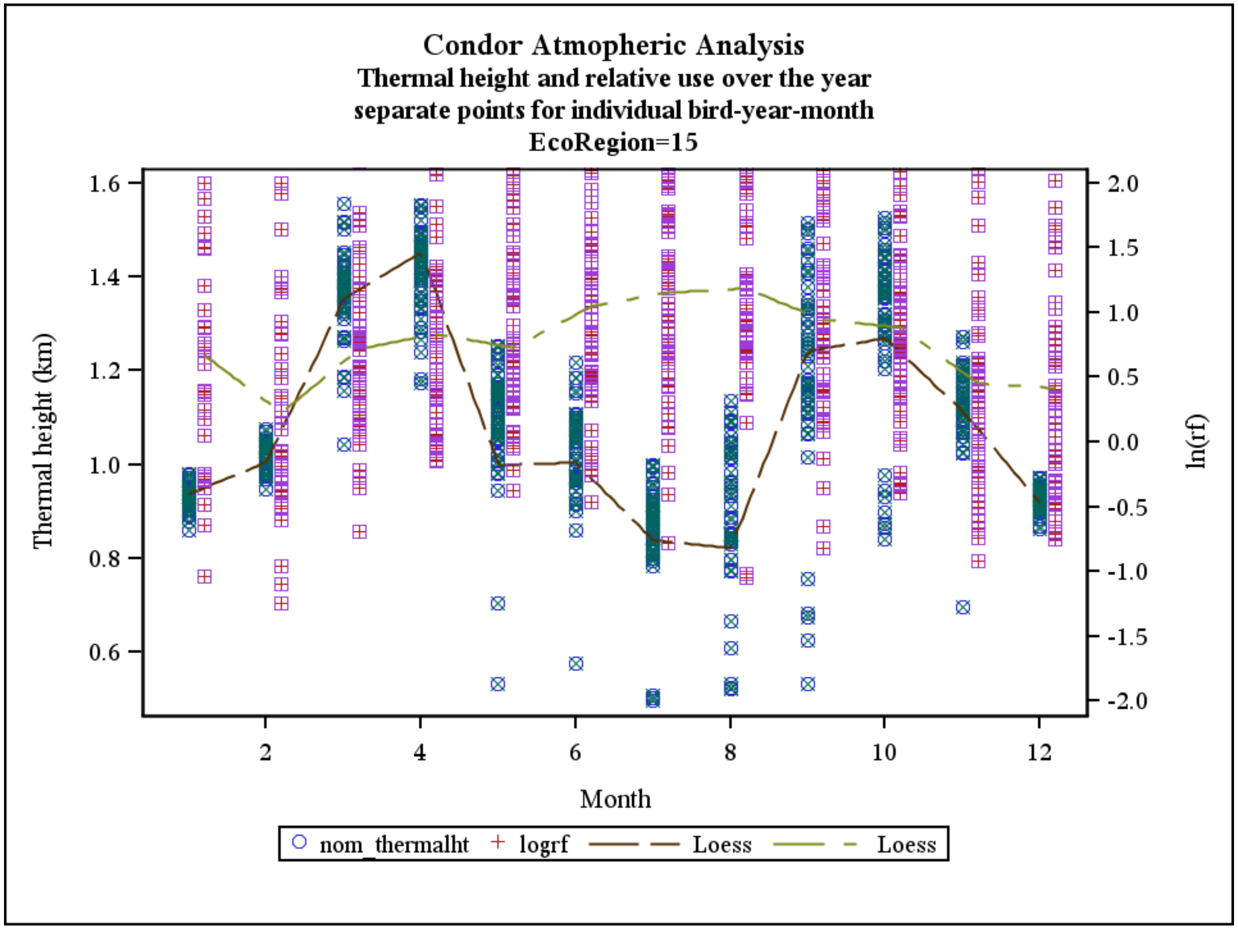

EcoRegion=16

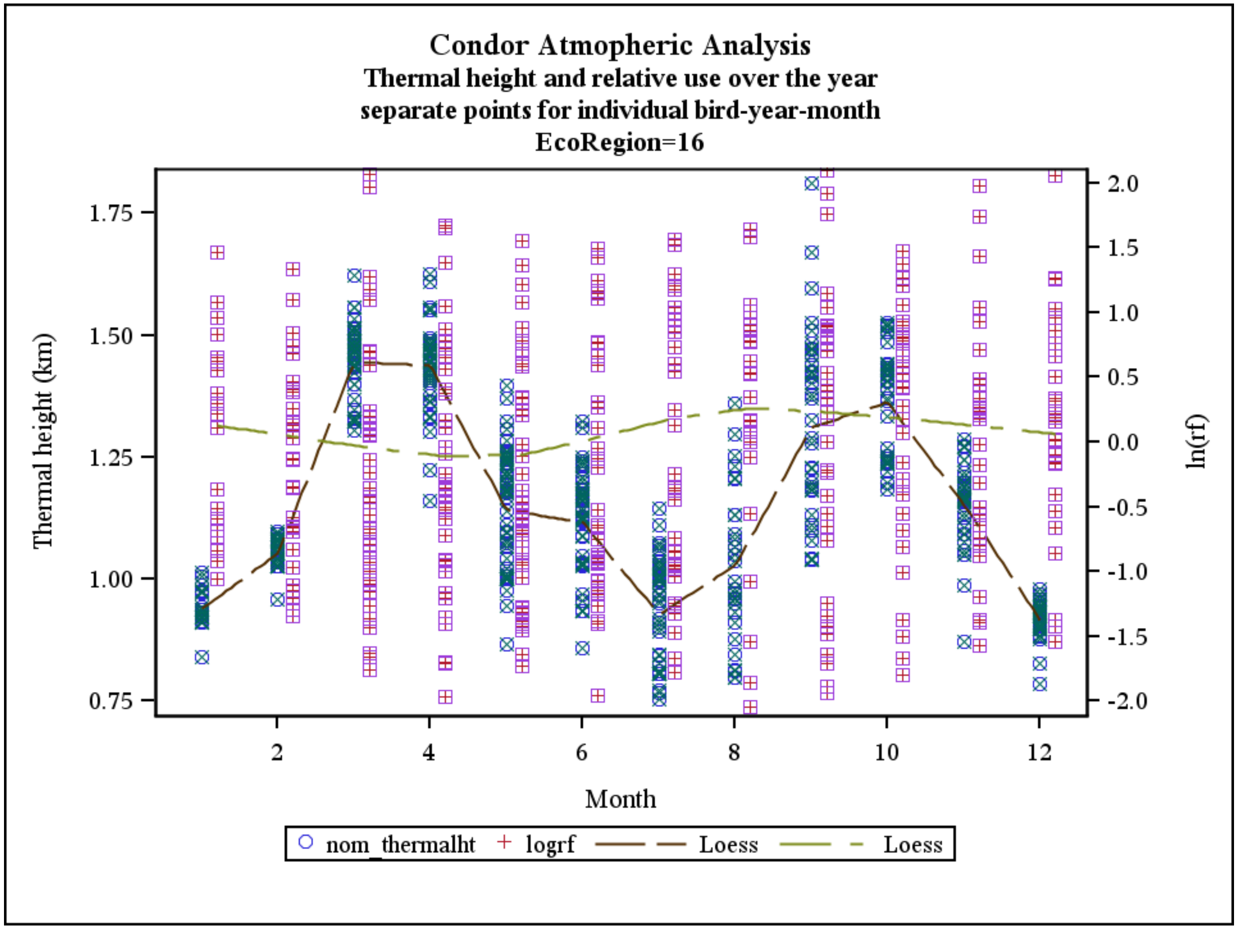

EcoRegion=18

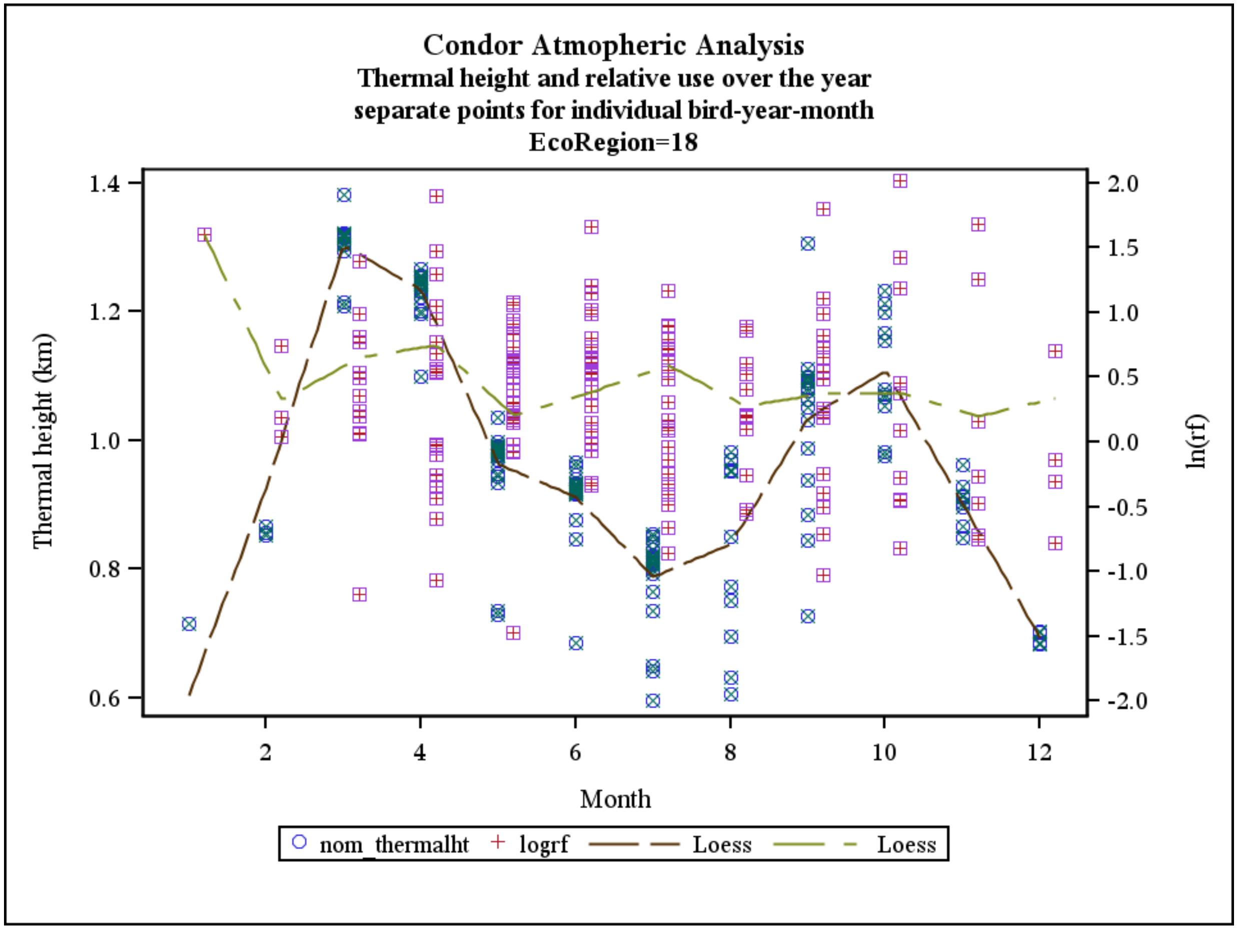

EcoRegion=39

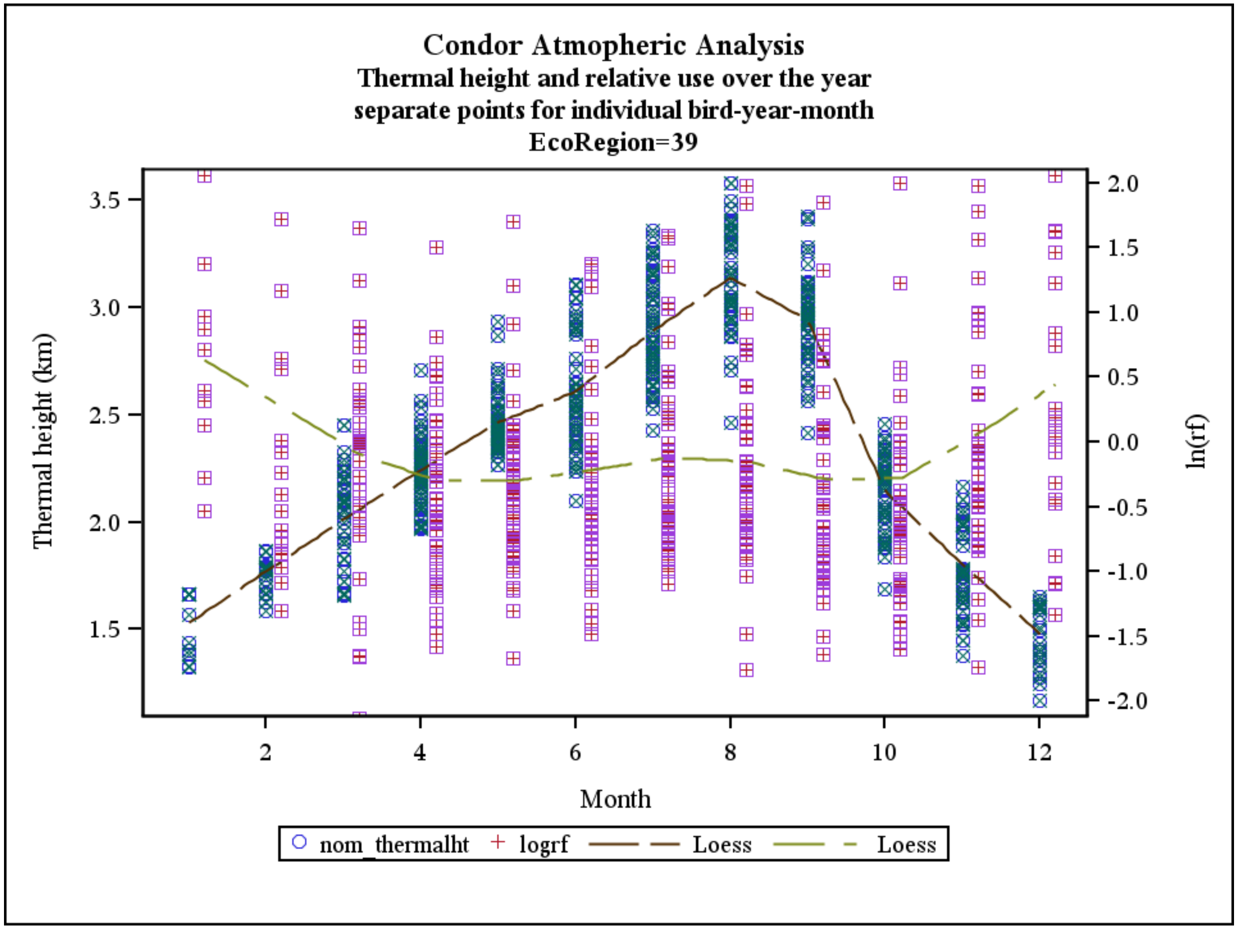

EcoRegion=40

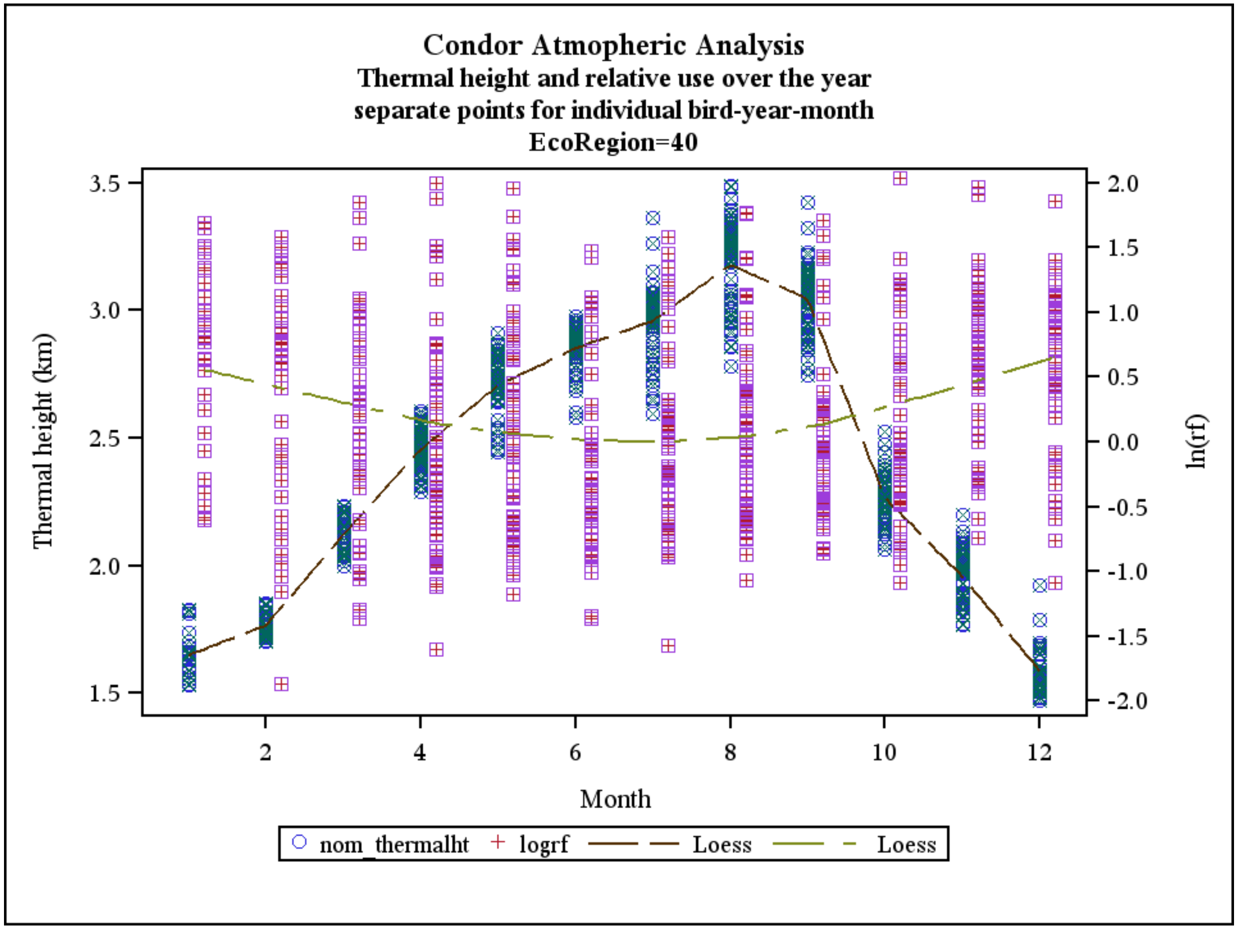

EcoRegion=95

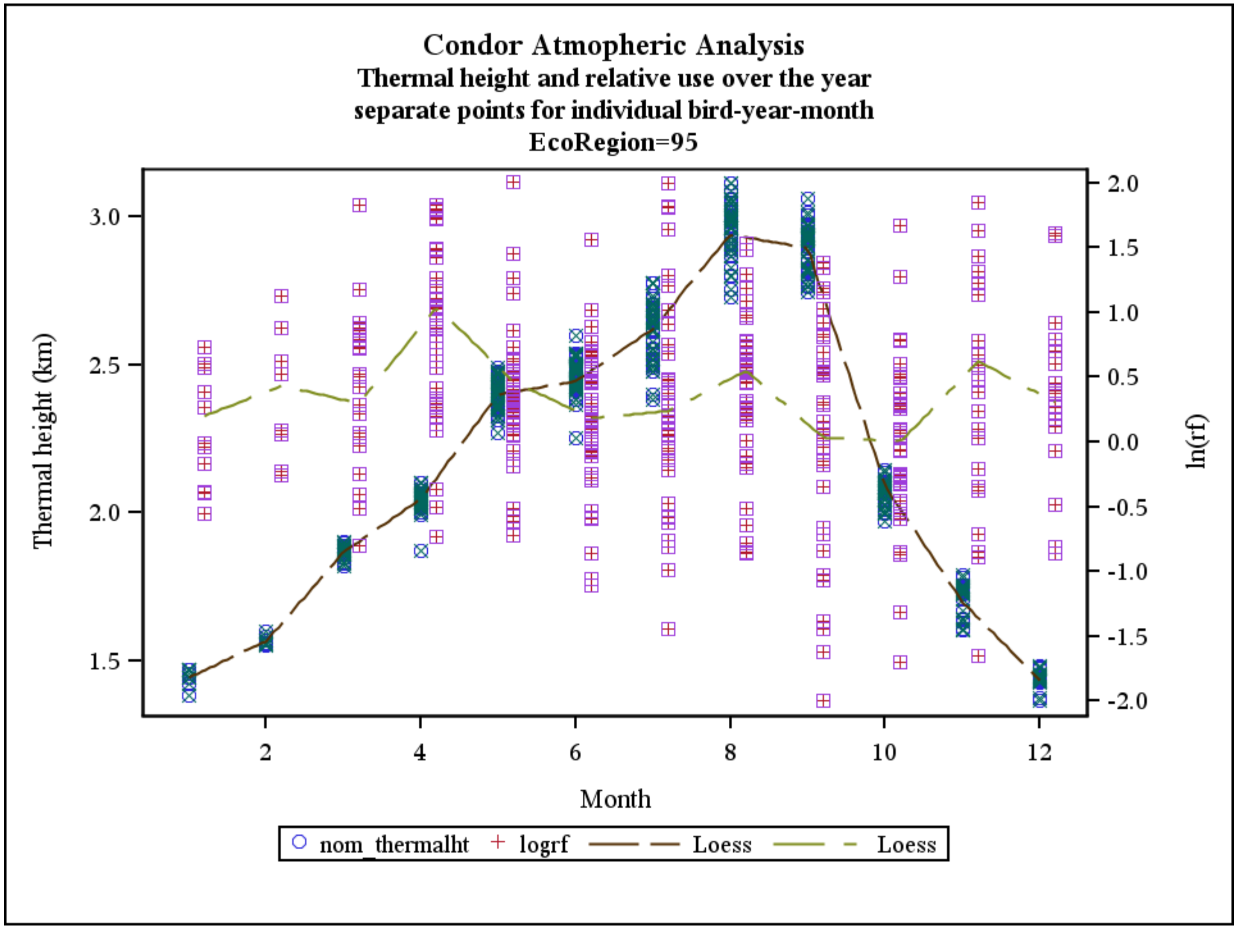

EcoRegion=101

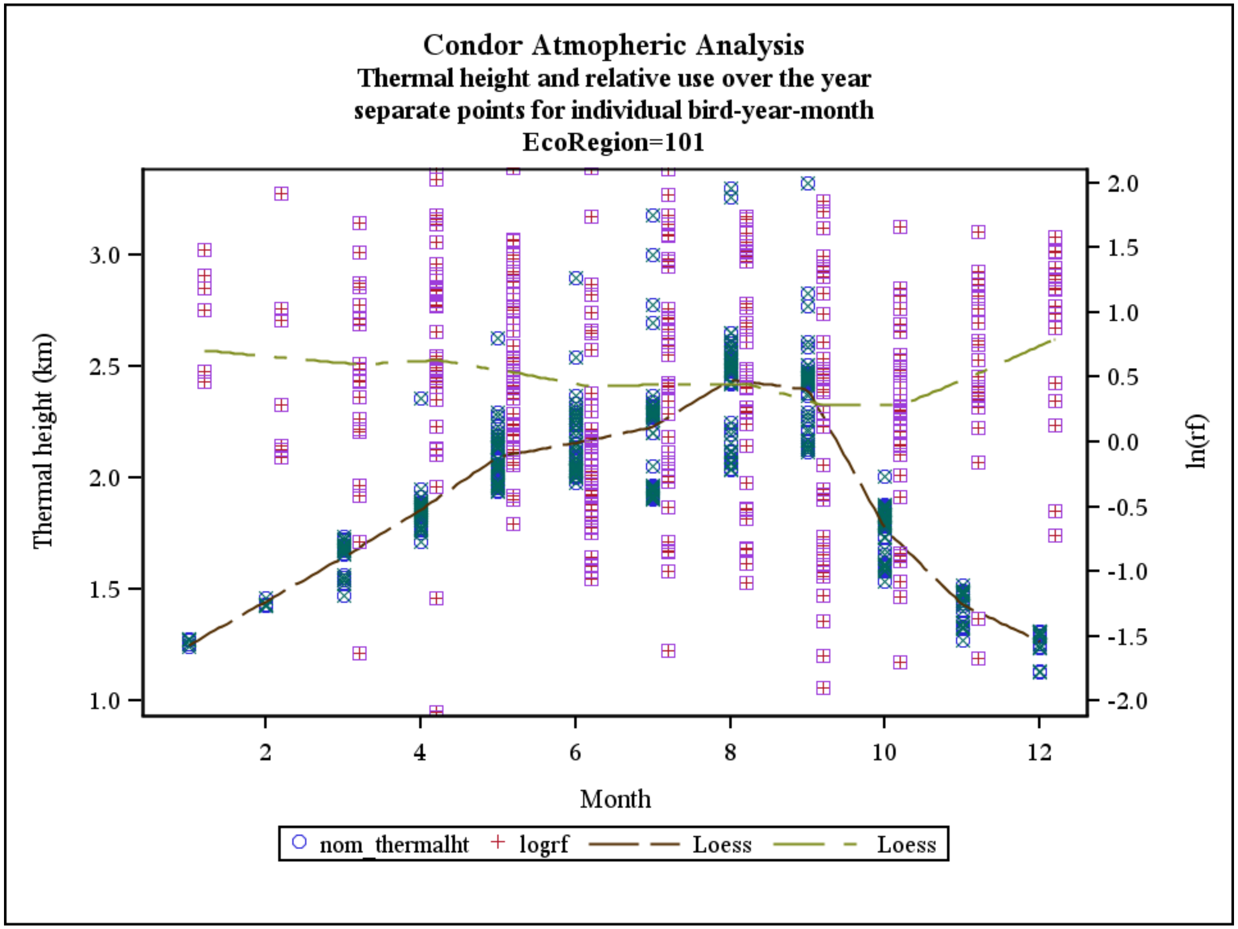

EcoRegion=102

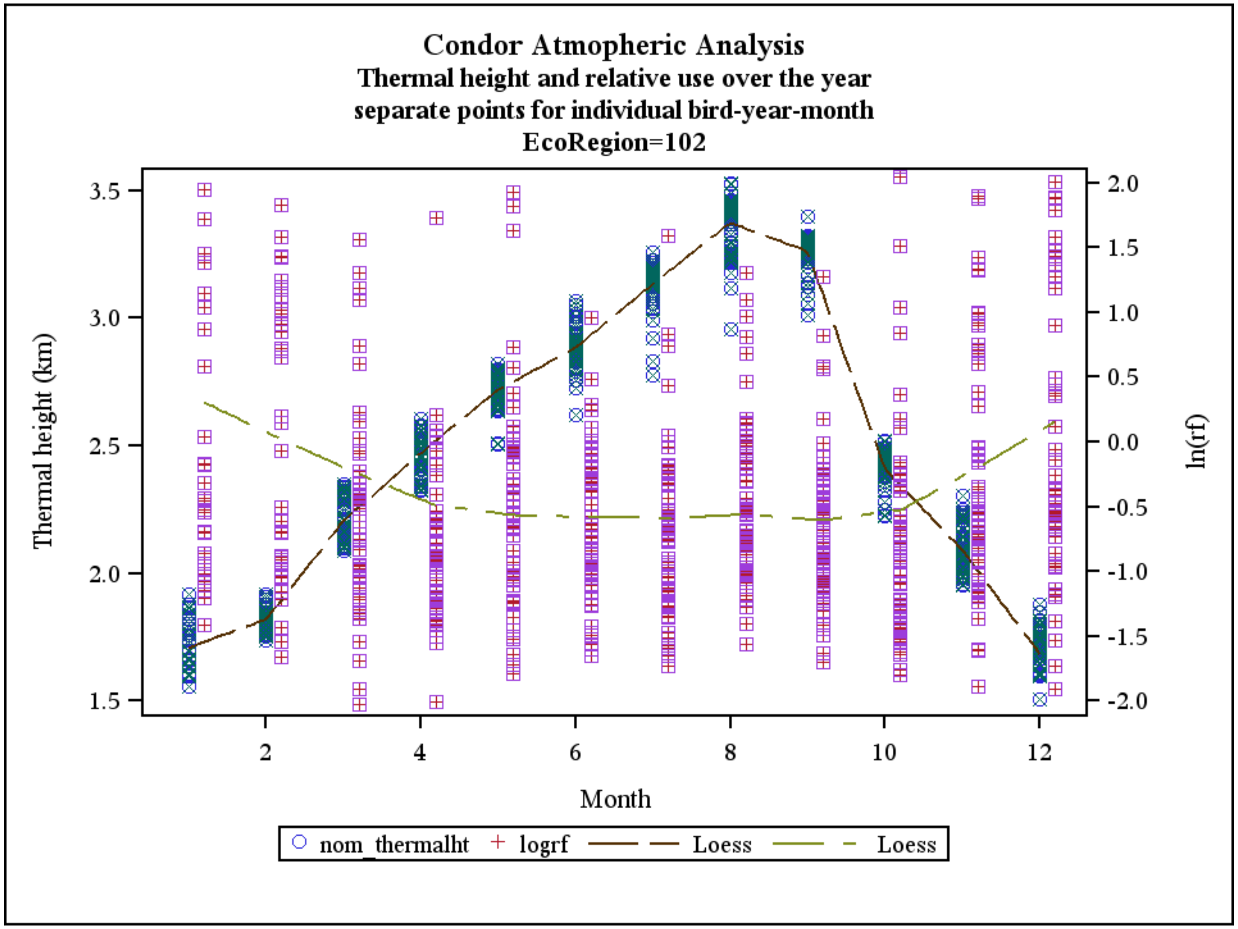

EcoRegion=116

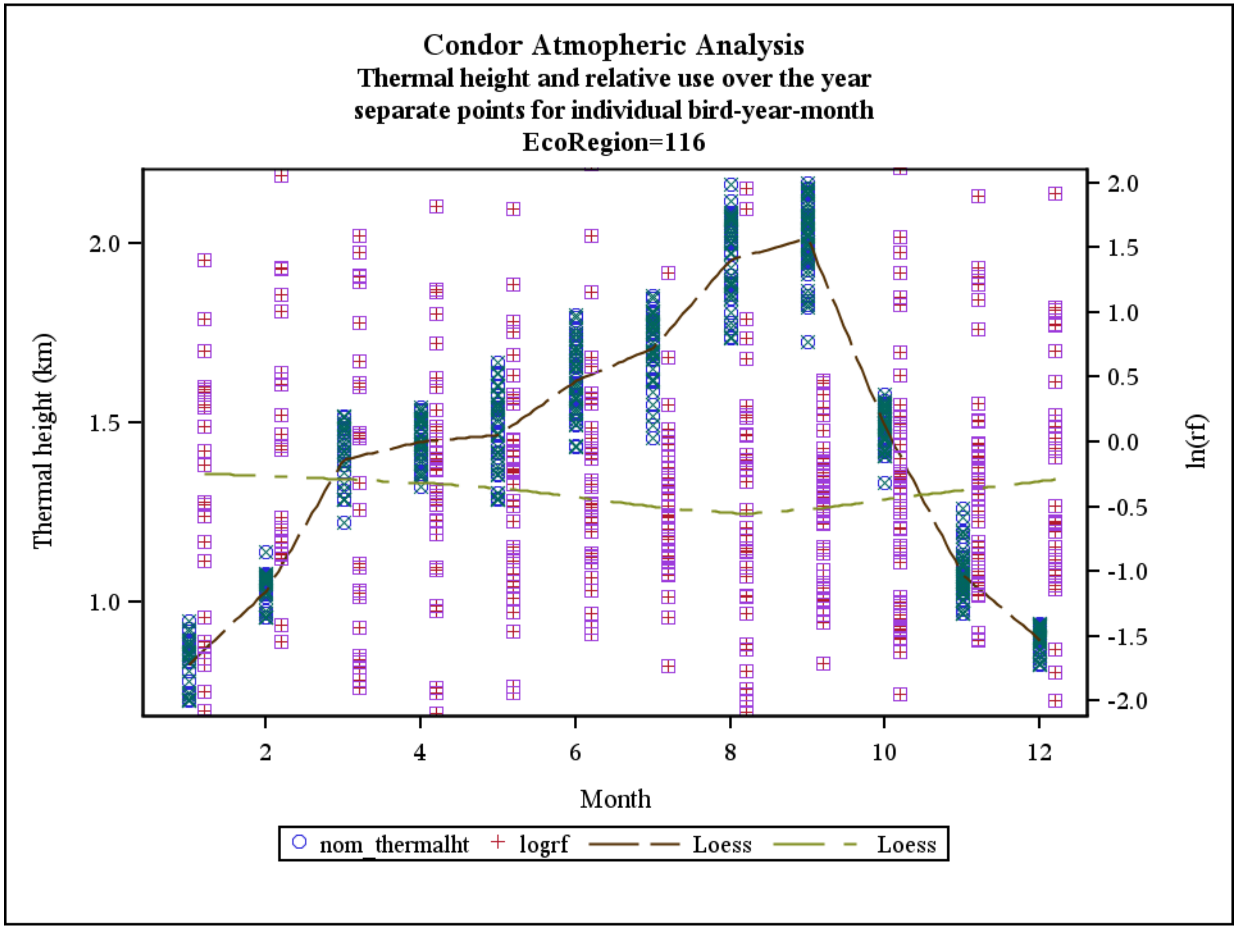

EcoRegion=117

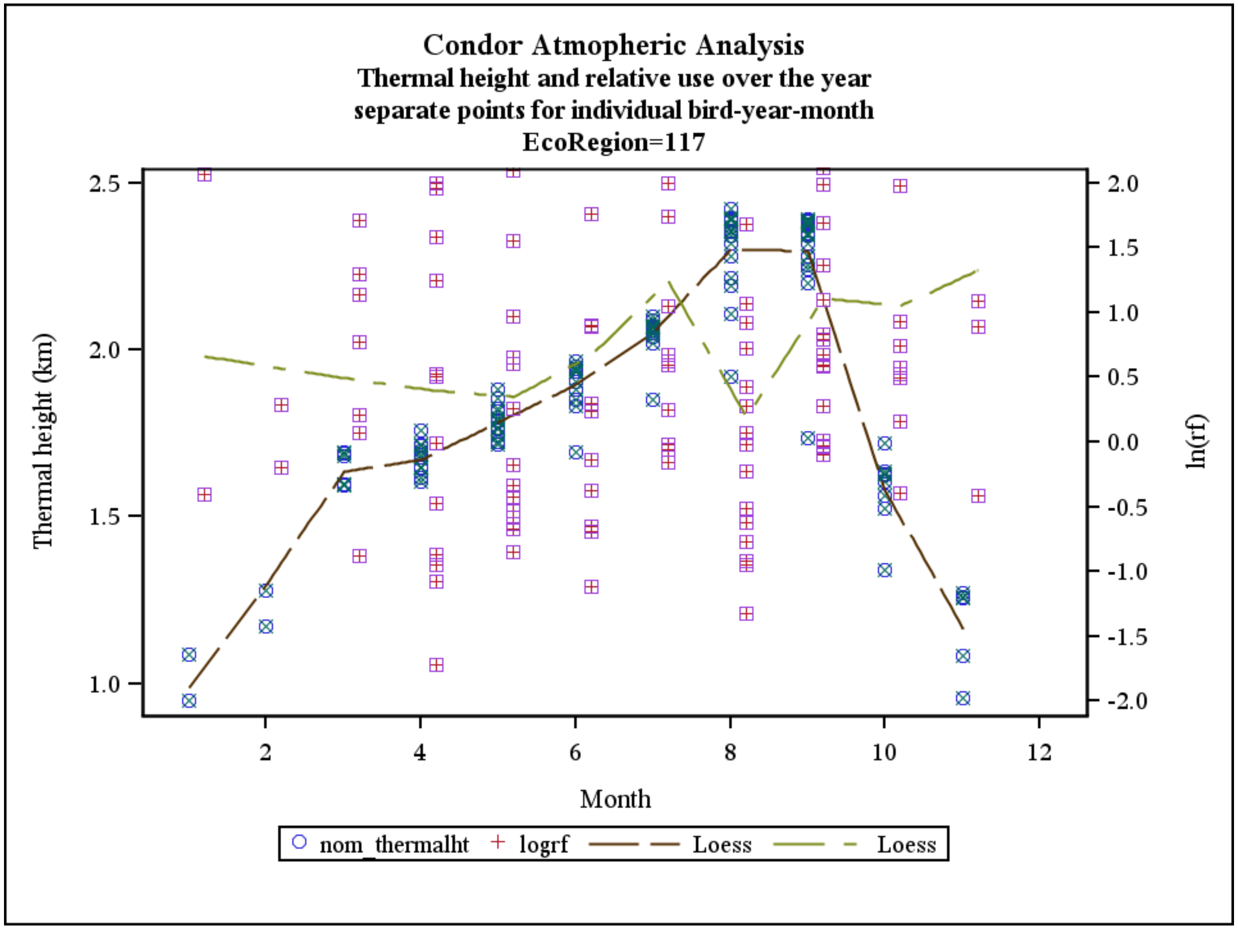

EcoRegion=118

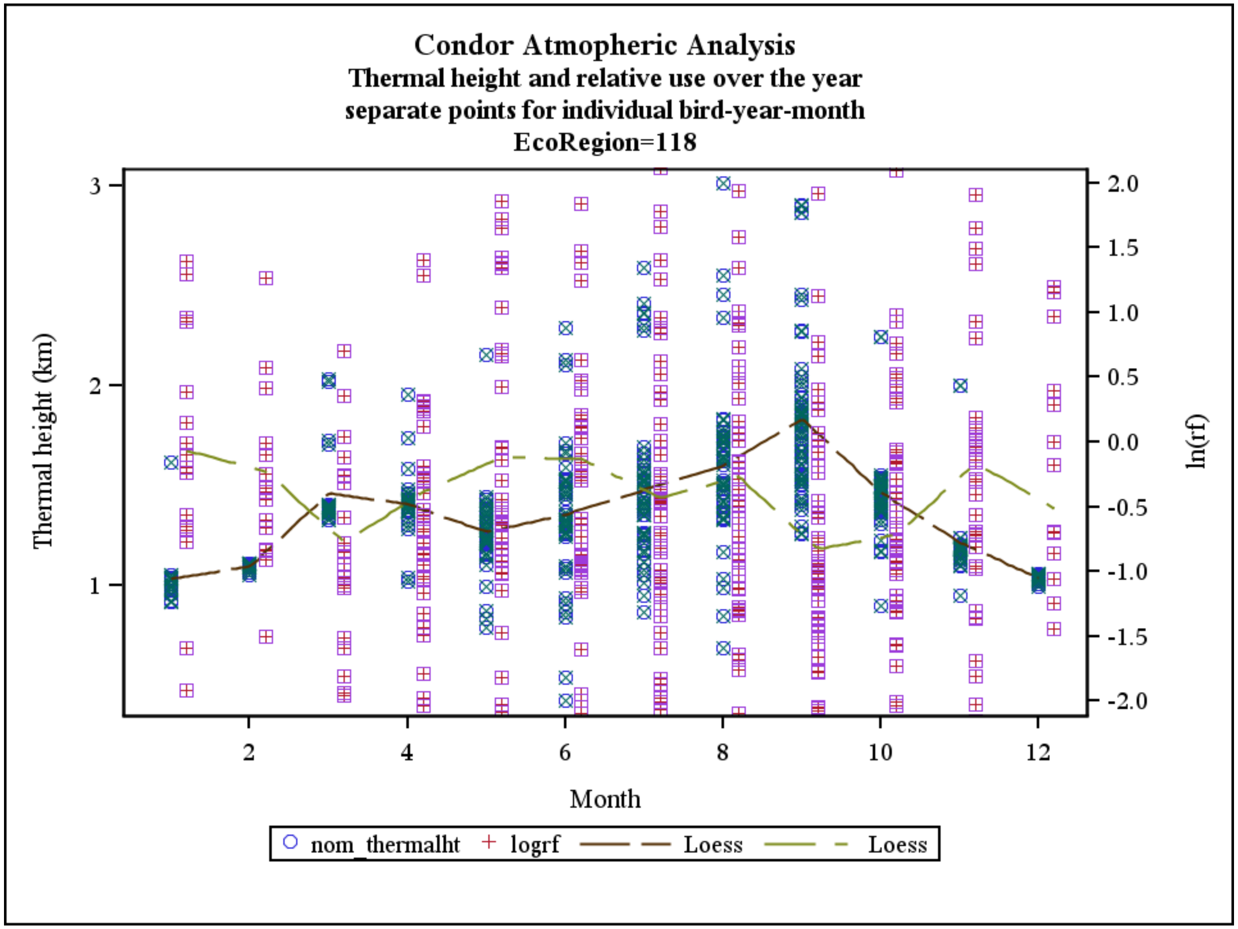

EcoRegion=119

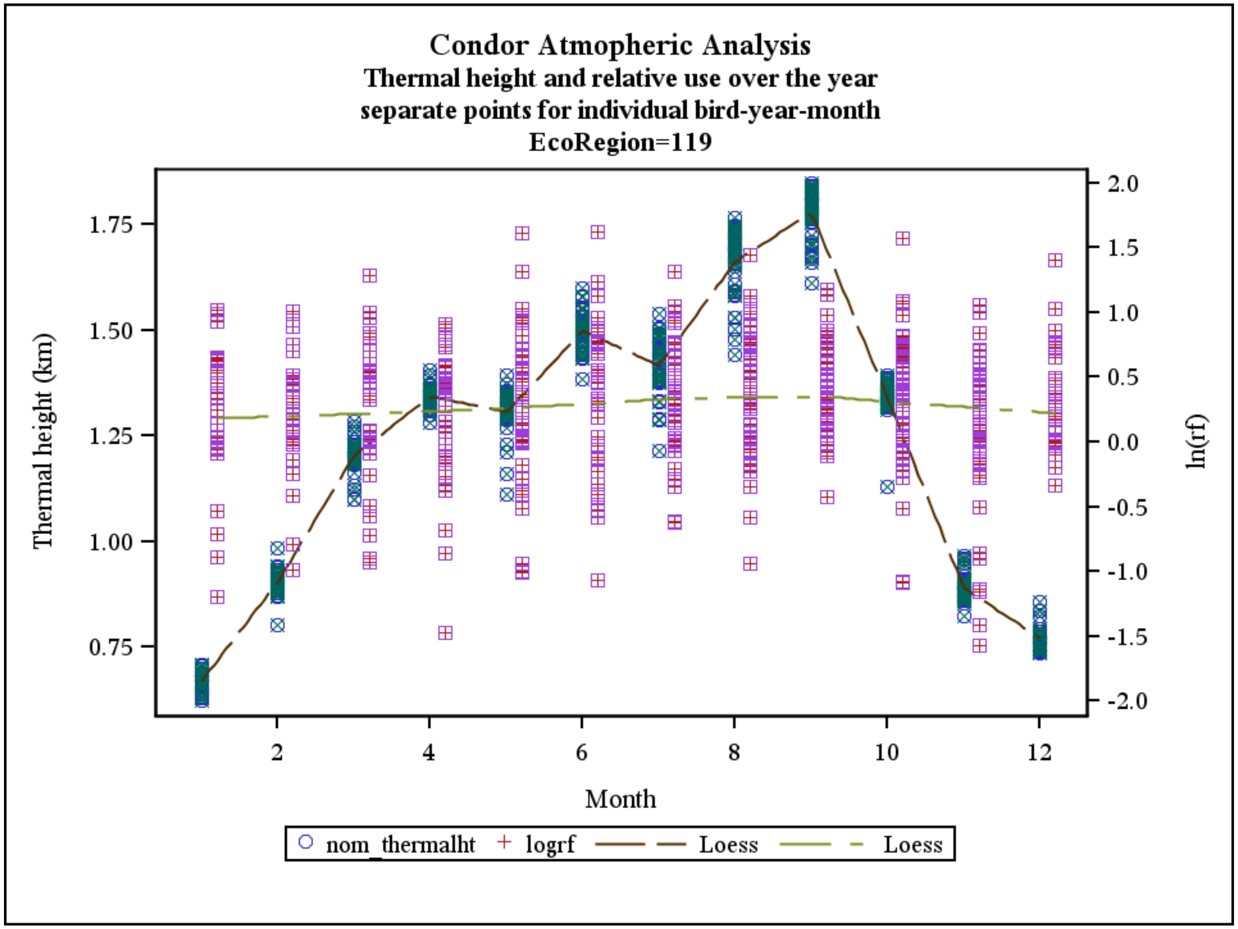

EcoRegion=123

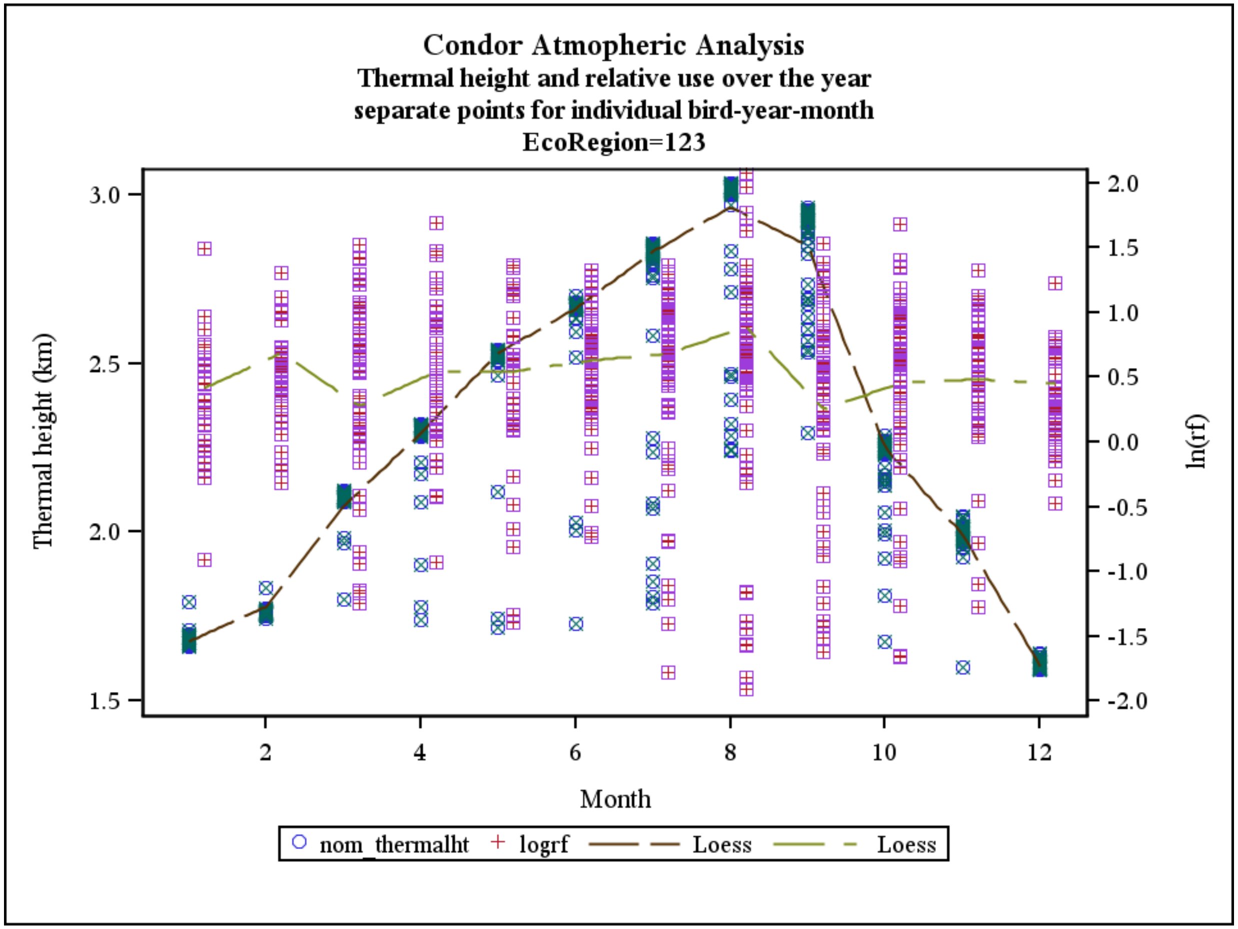

EcoRegion=124

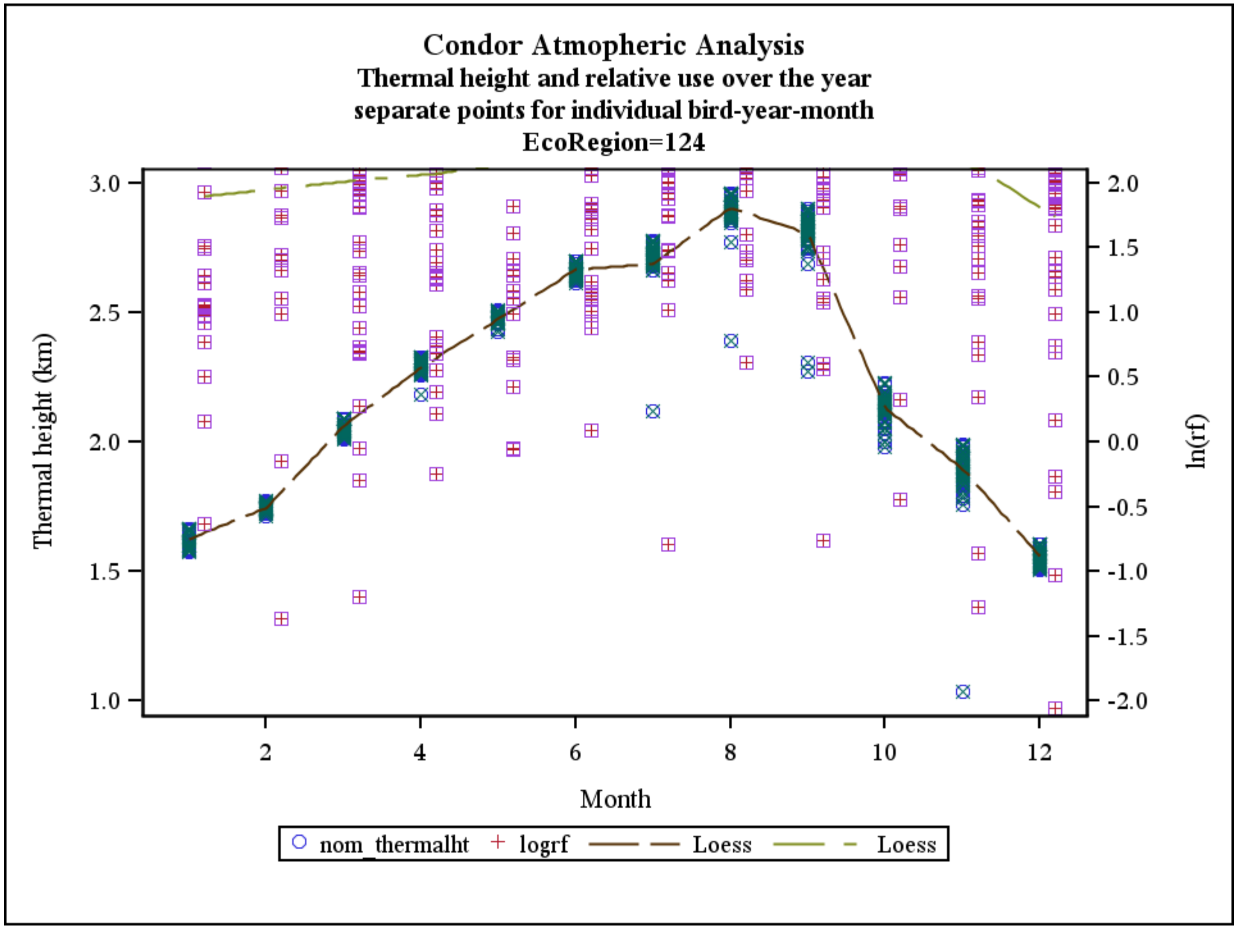

EcoRegion=125

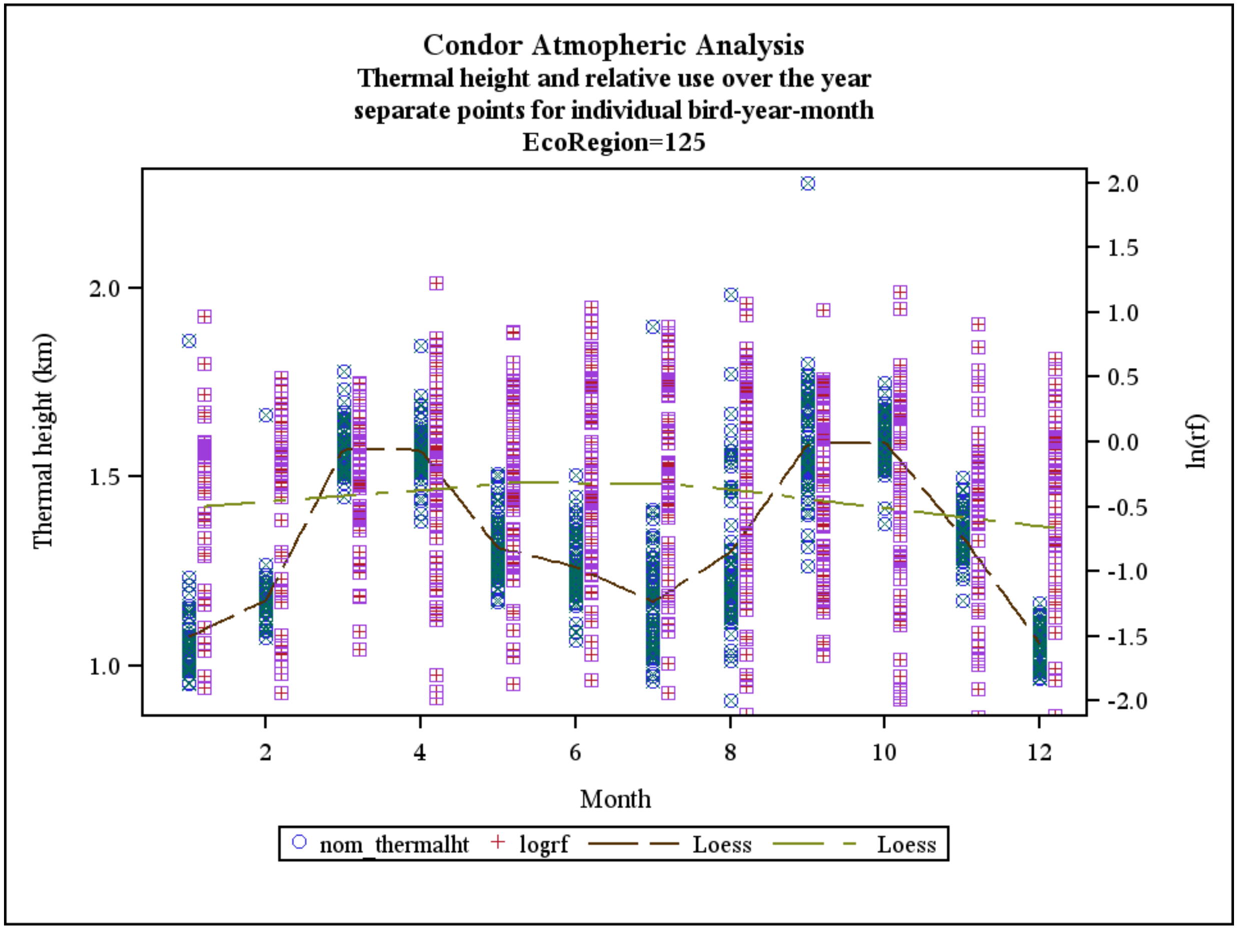

EcoRegion=126

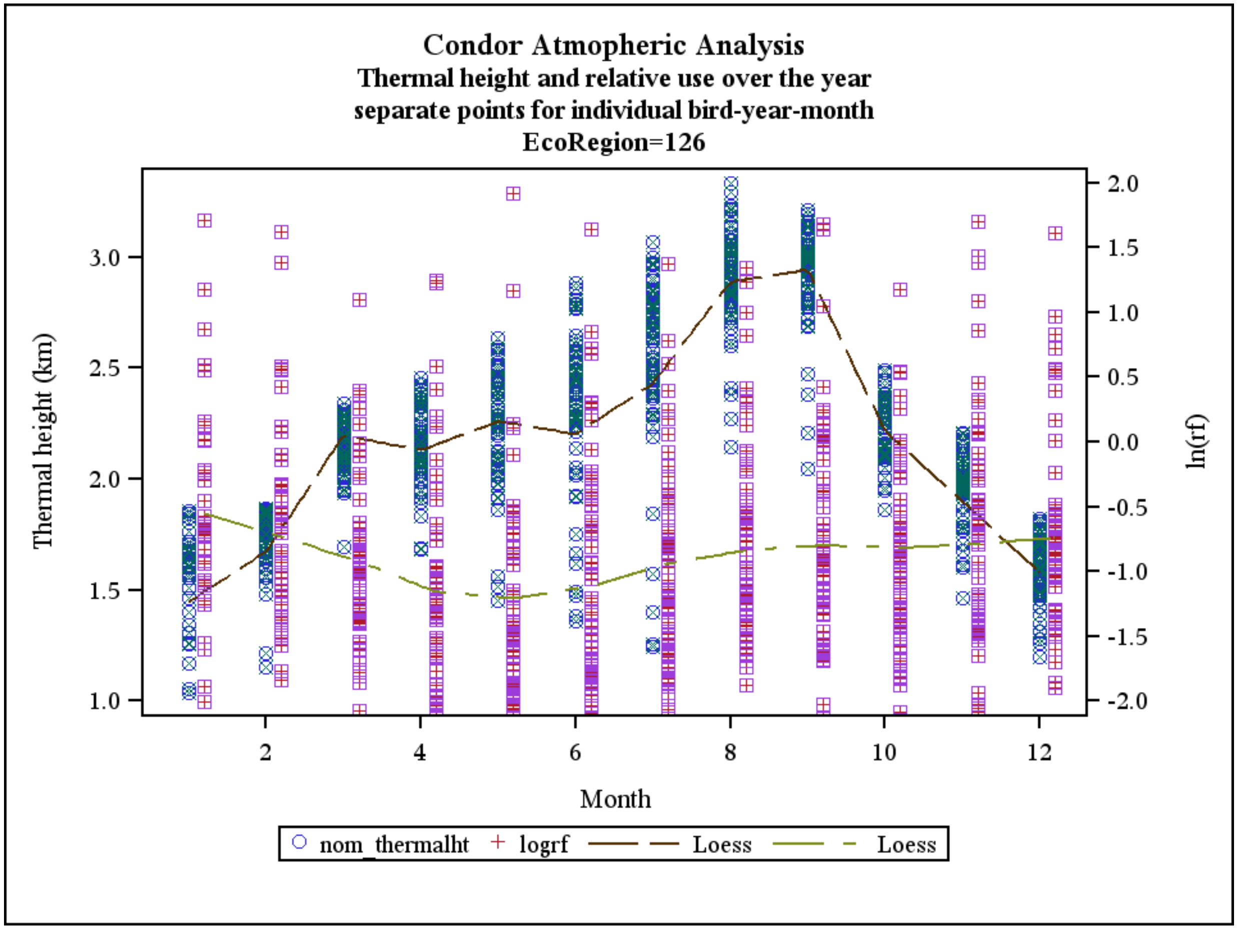

EcoRegion=127

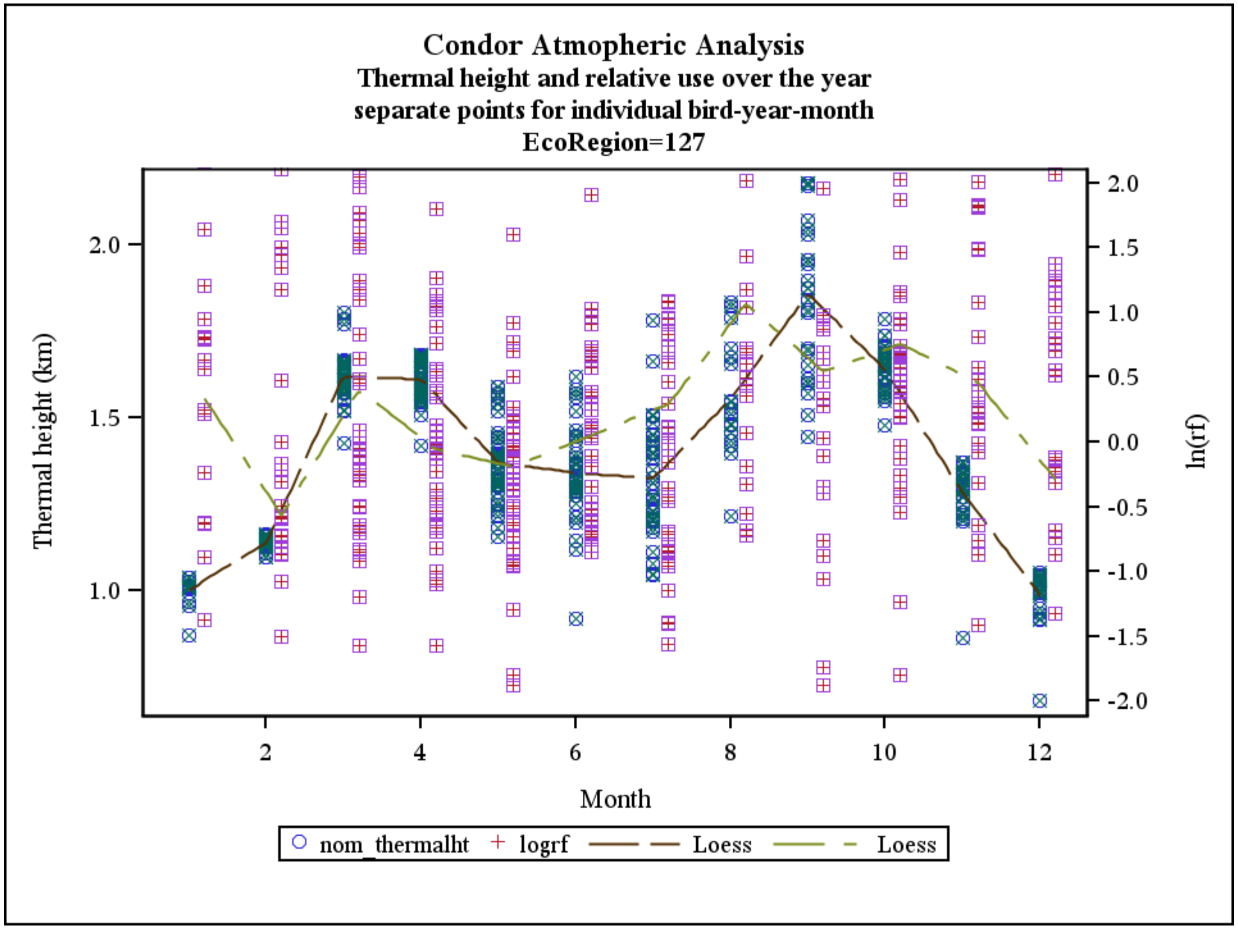

EcoRegion=128

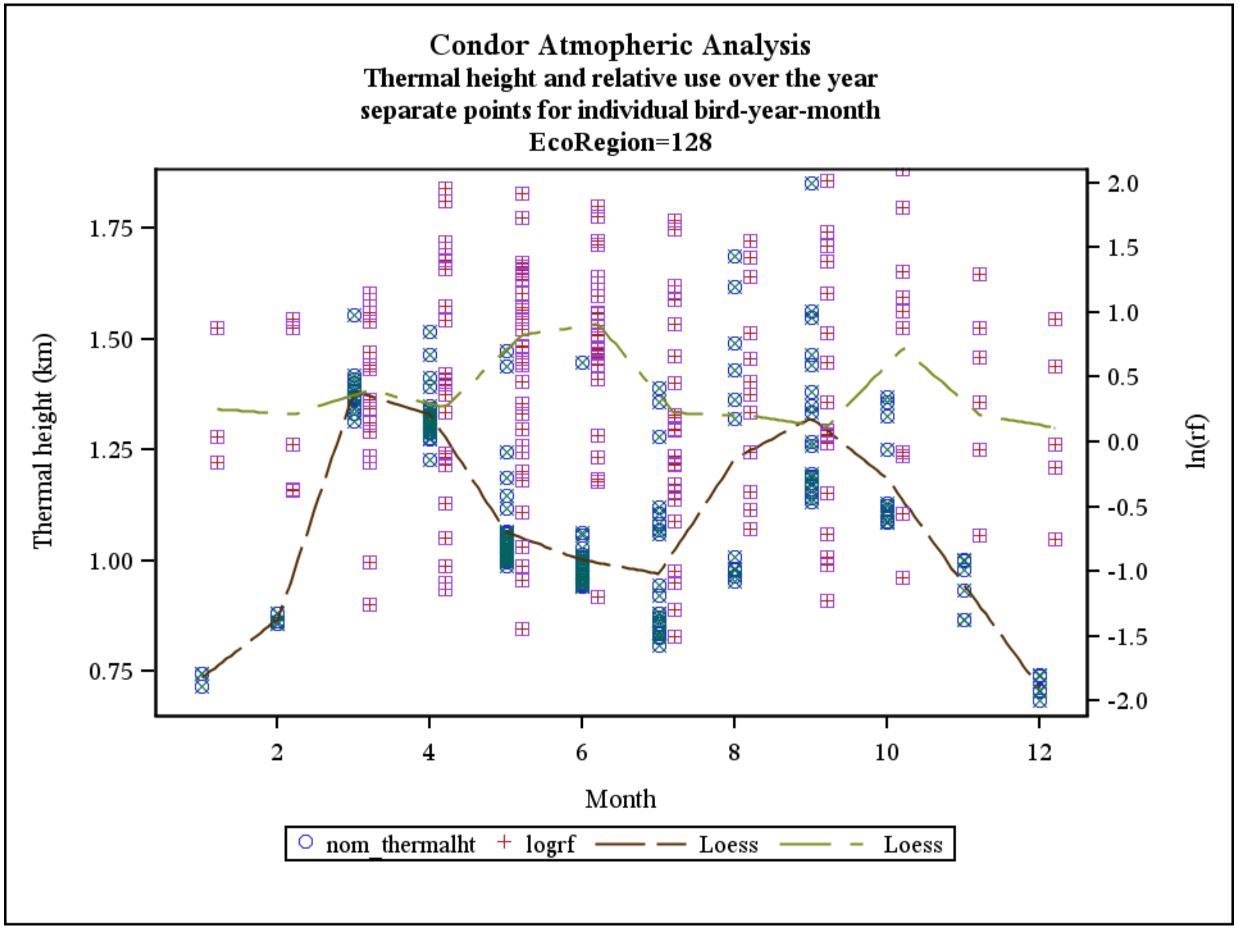

EcoRegion=147

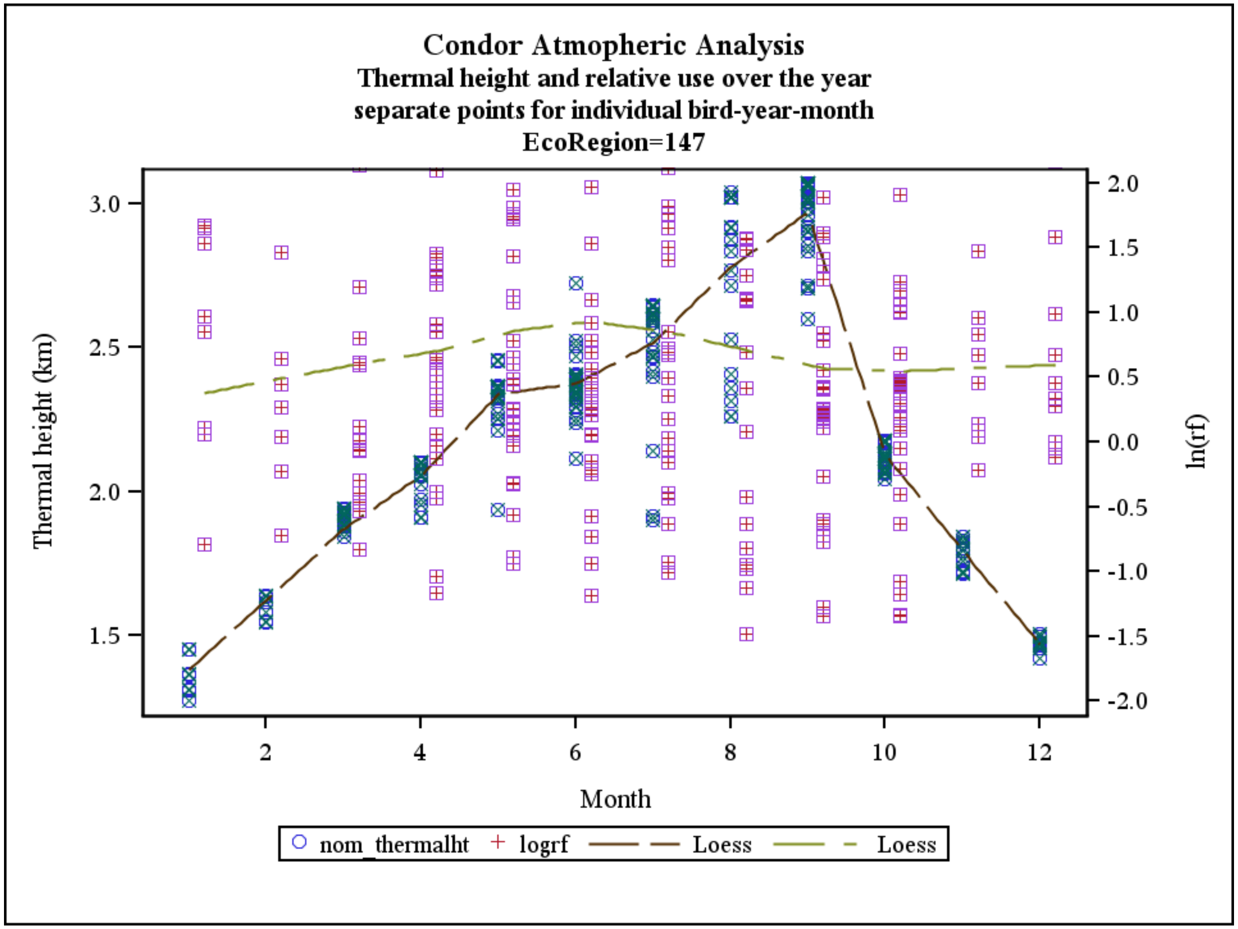

EcoRegion=192

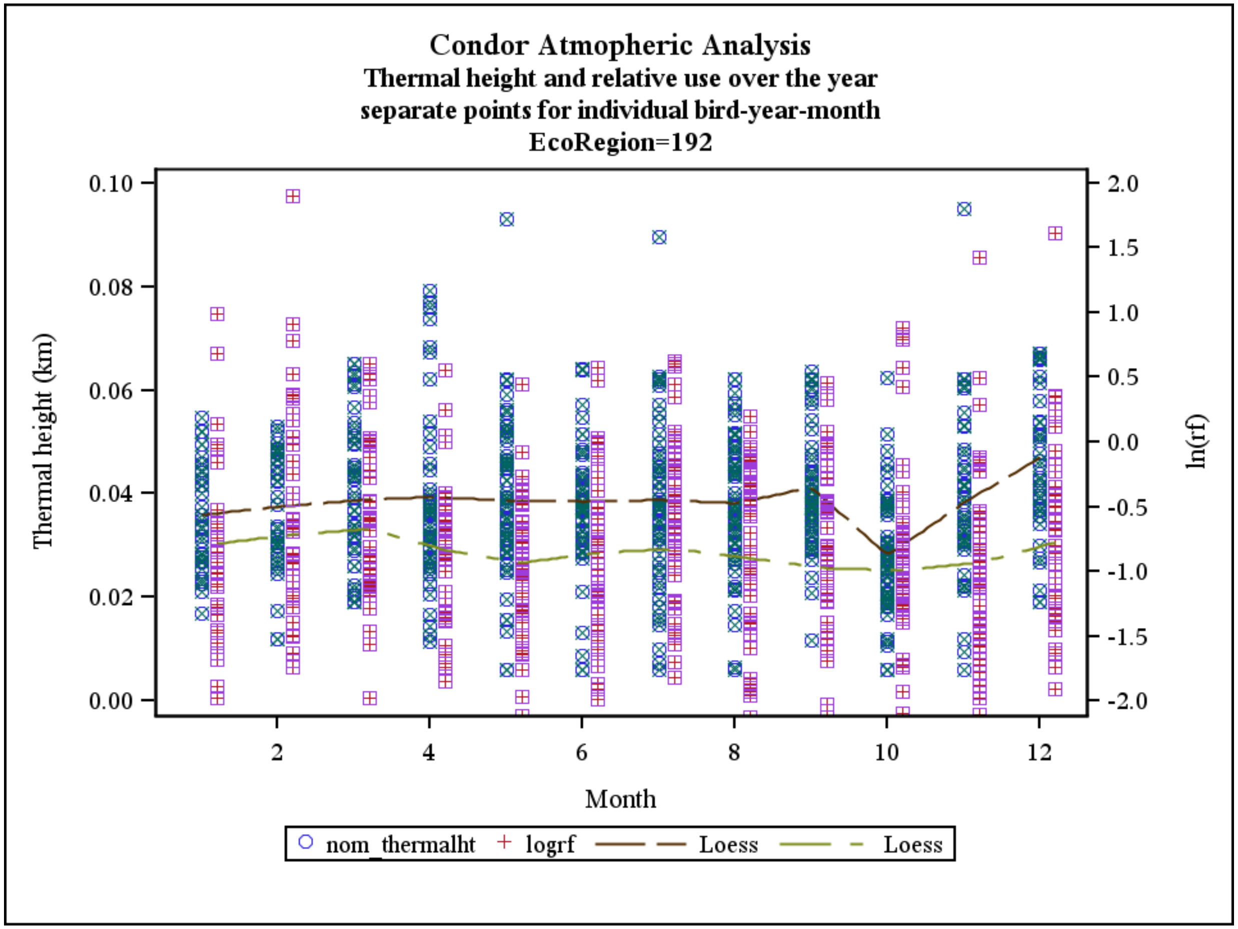

EcoRegion=193

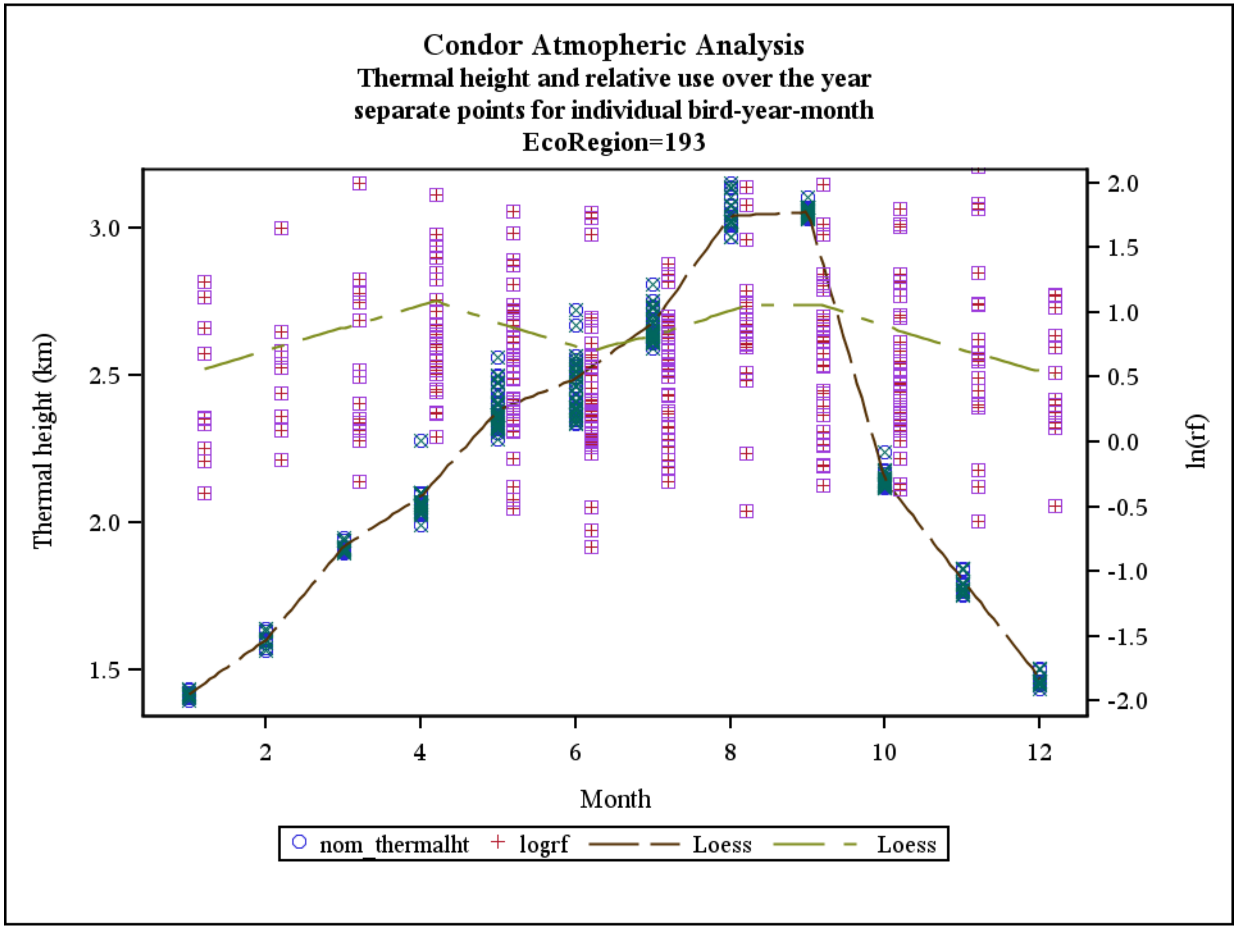

EcoRegion=8

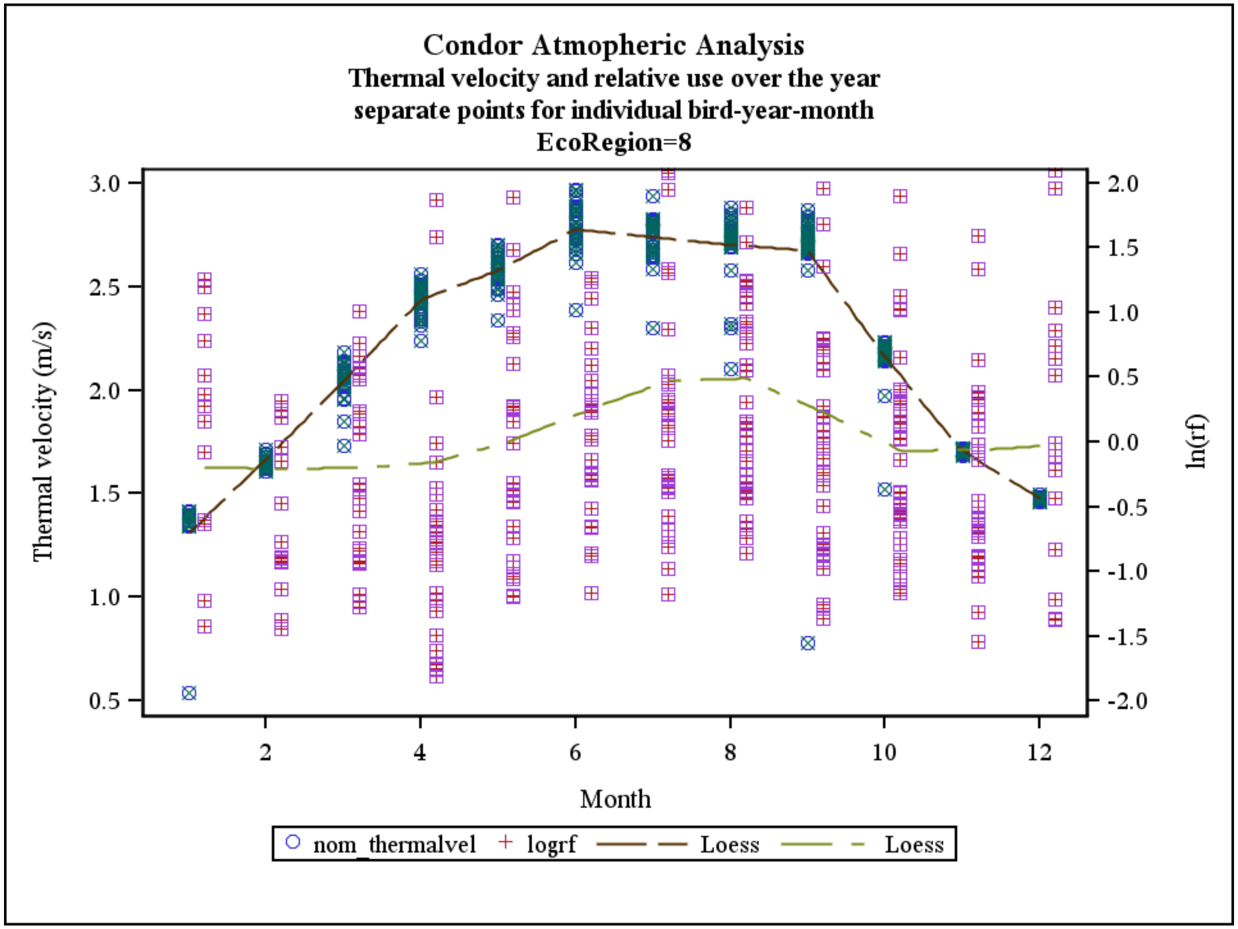
Condor Atmopheric Analysis
Thermal velocity and relative use over the year
separate points for individual bird-year-month
EcoRegion=8
Thermal velocity (m/s)
3.0
2.5
2.0
1.5
1.0
0.5
2
4
6
8
10
12
Month
ln(rf)
2.0
1.5
1.0
0.5
0.0
-0.5
-1.0
-1.5
-2.0
nom_thermalvel
logrf
Loess
Loess

EcoRegion=9

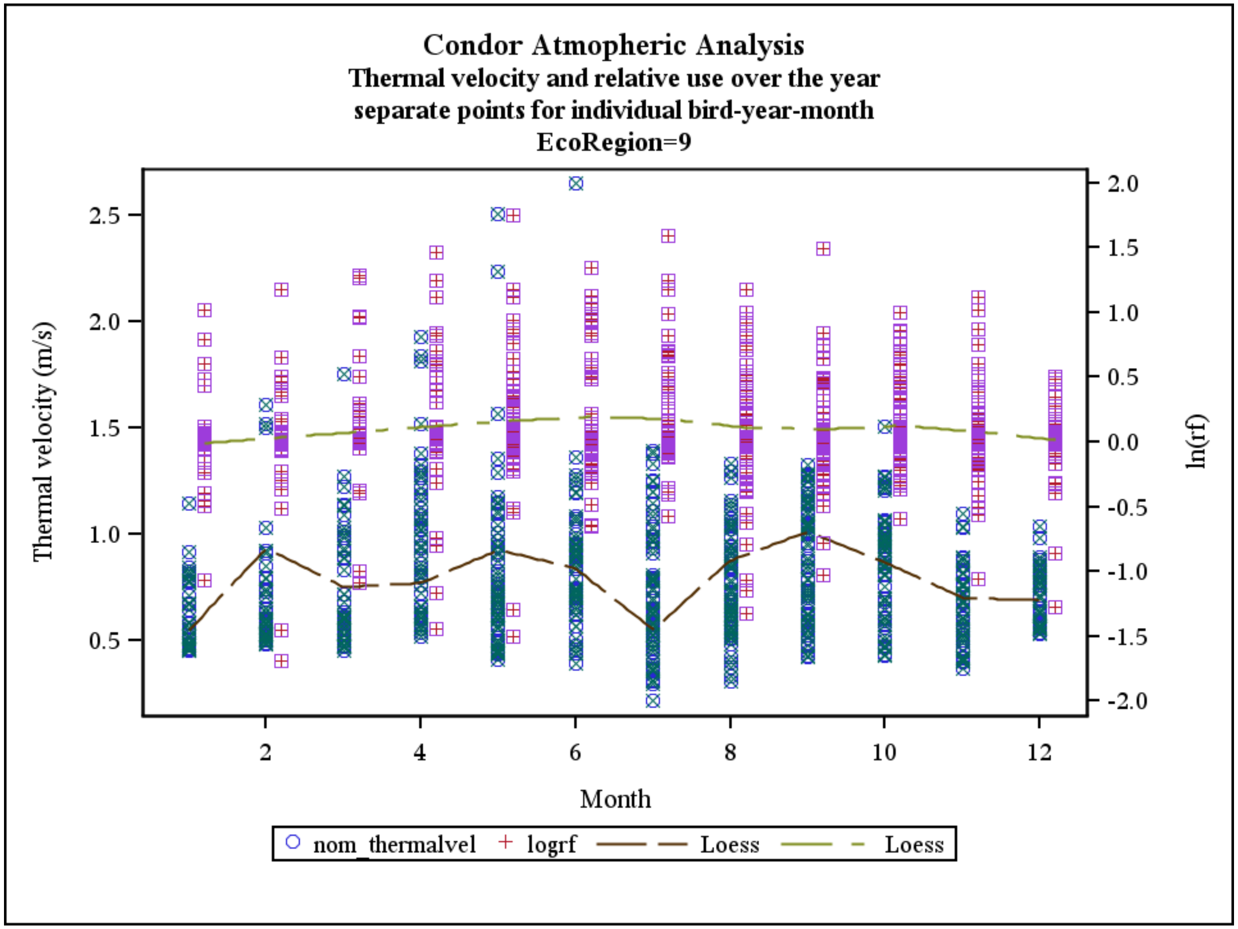
Condor Atmopheric Analysis
Thermal velocity and relative use over the year
separate points for individual bird-year-month
EcoRegion=9
Thermal velocity (m/s)
ln(rf)
Month
2.5
2.0
1.5
1.0
0.5
2.0
1.5
1.0
0.5
0.0
-0.5
-1.0
-1.5
-2.0
2
4
6
8
10
12
nom_thermalvel
logrf
Loess
Loess

EcoRegion=10

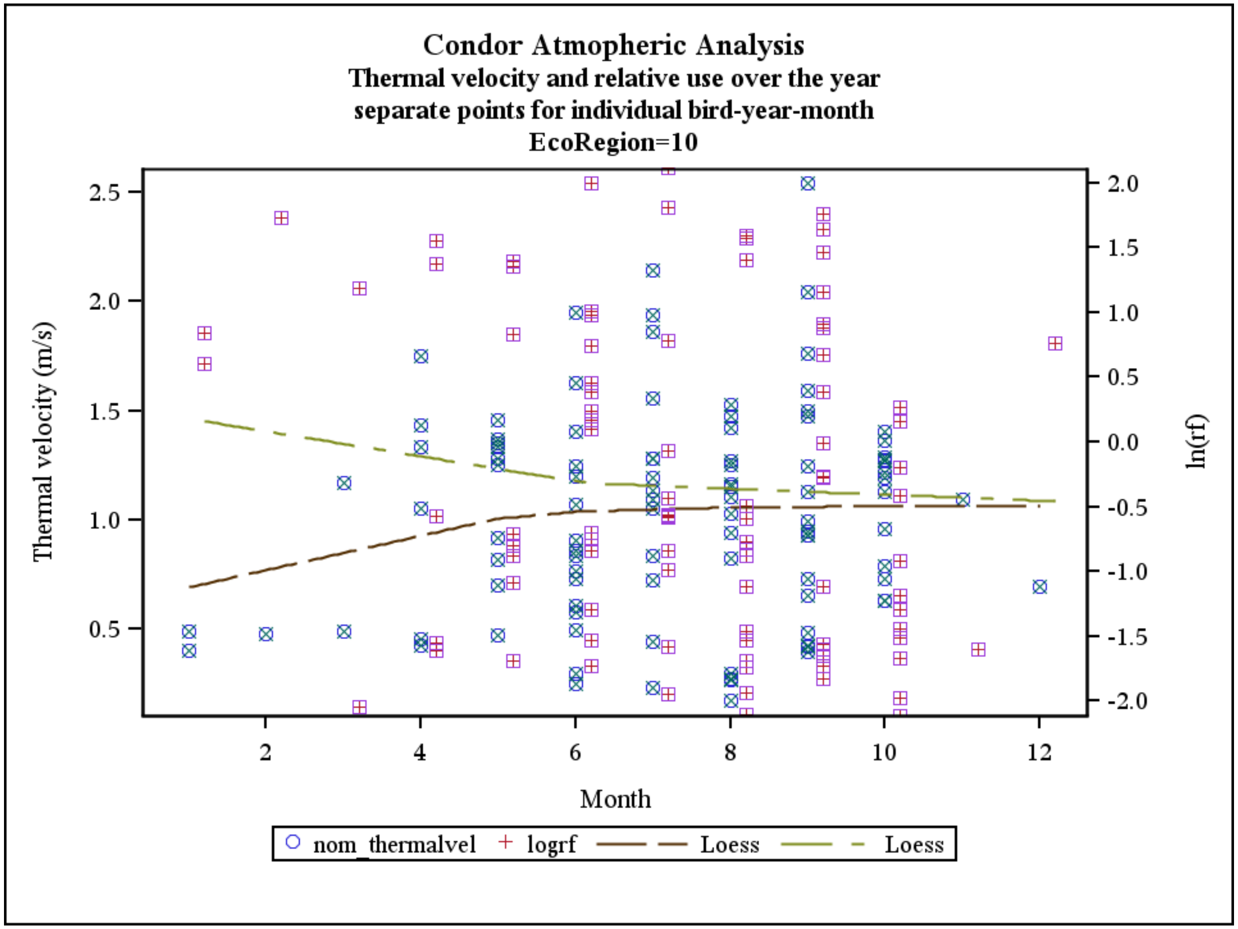

EcoRegion=13

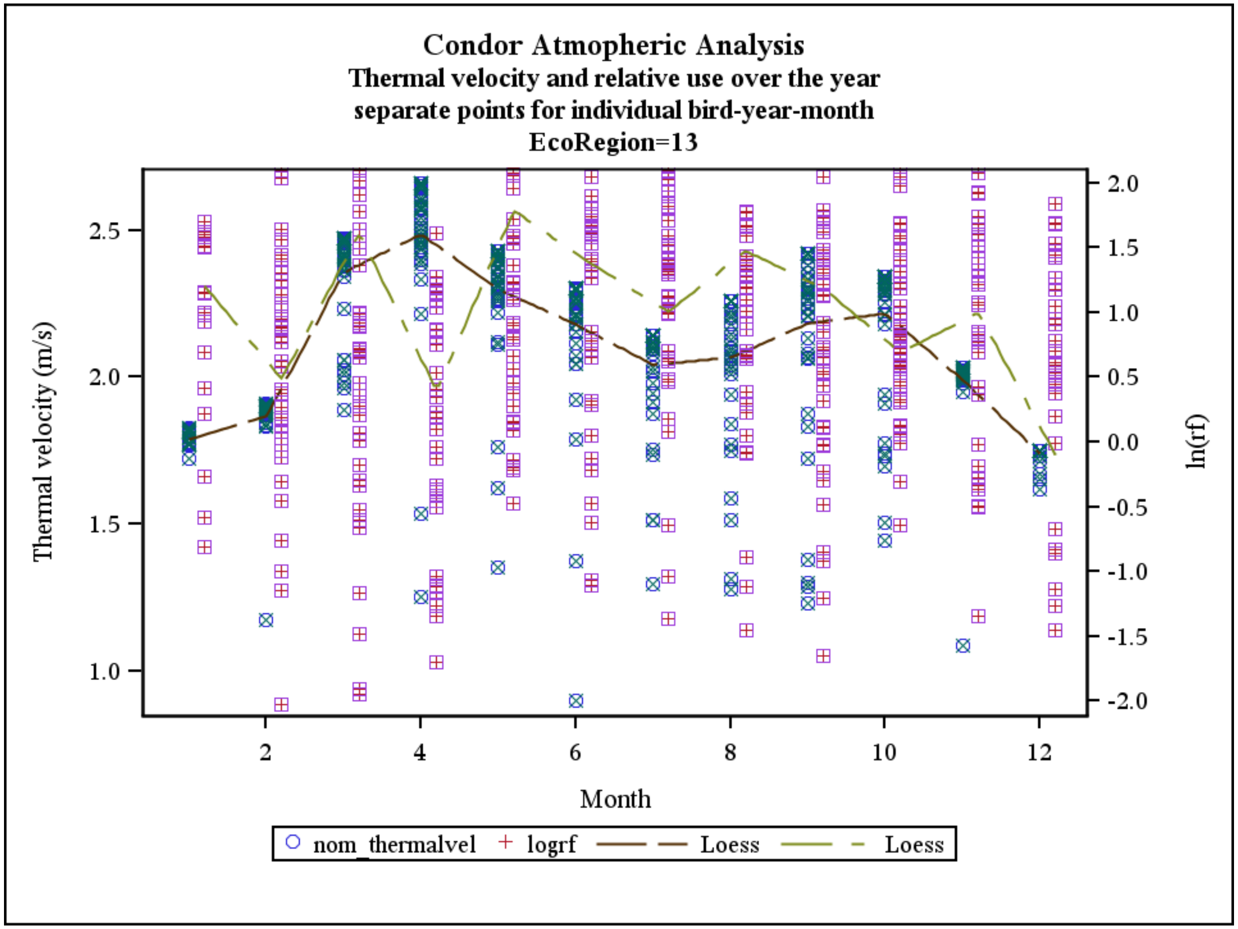

EcoRegion=15

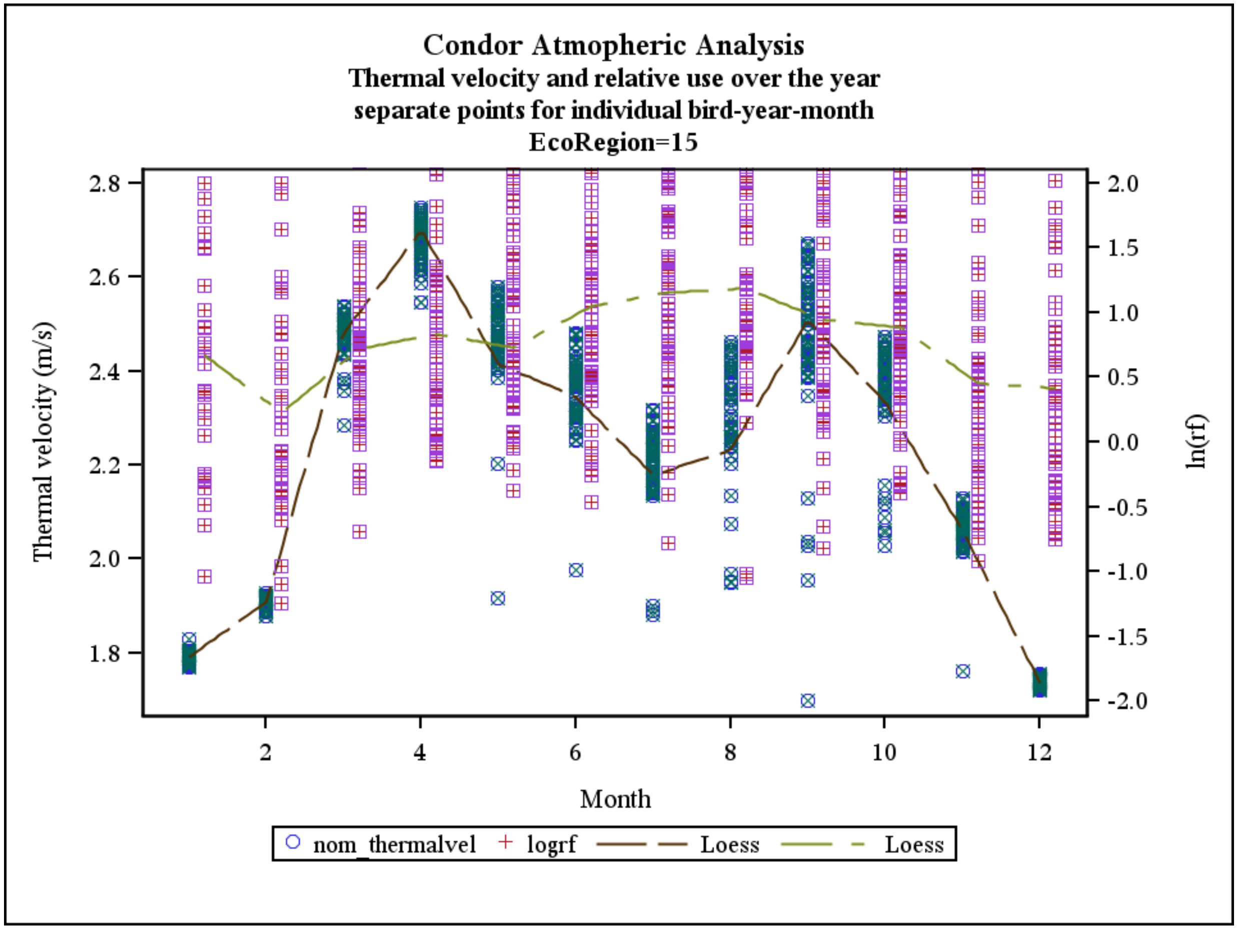

EcoRegion=16

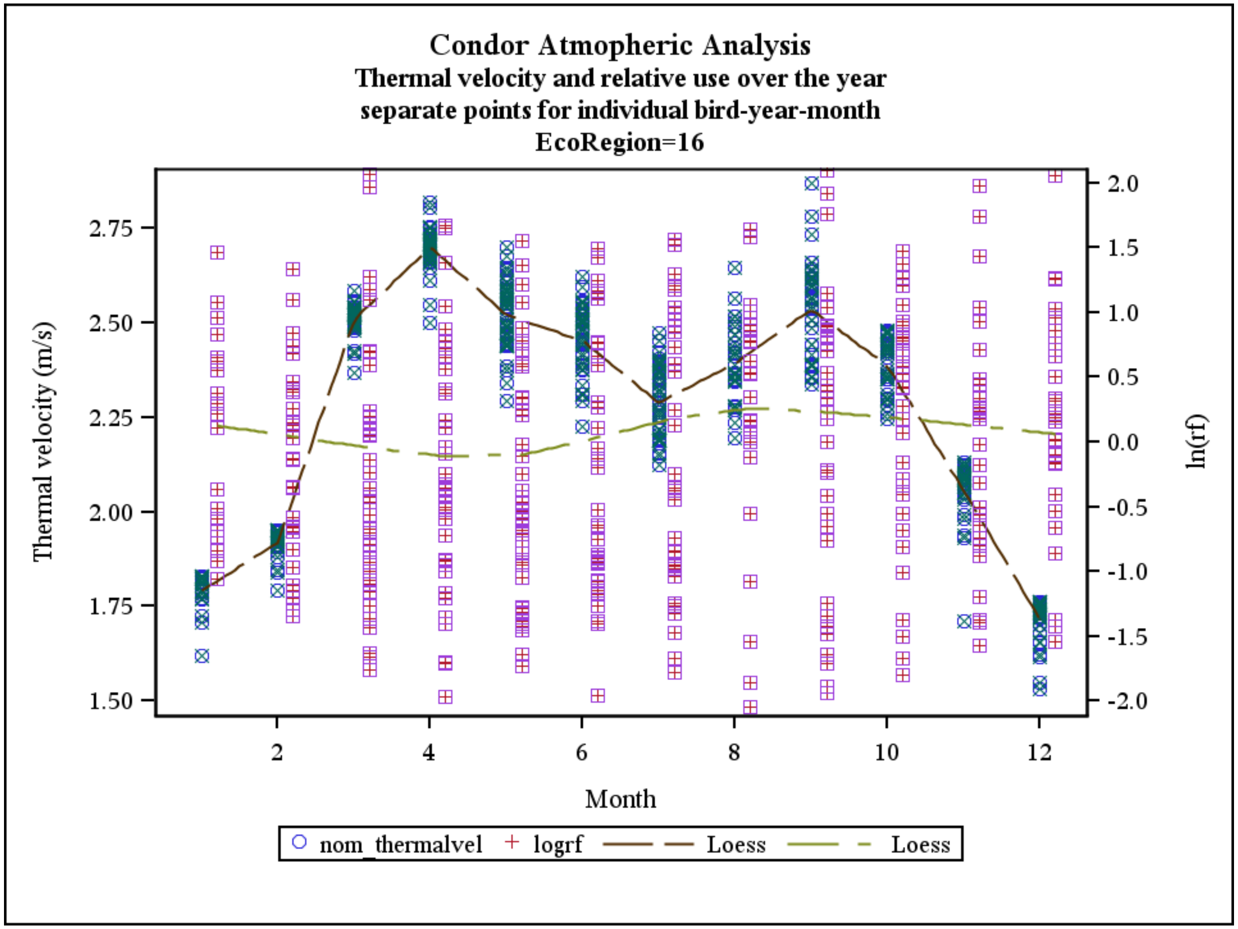

EcoRegion=18

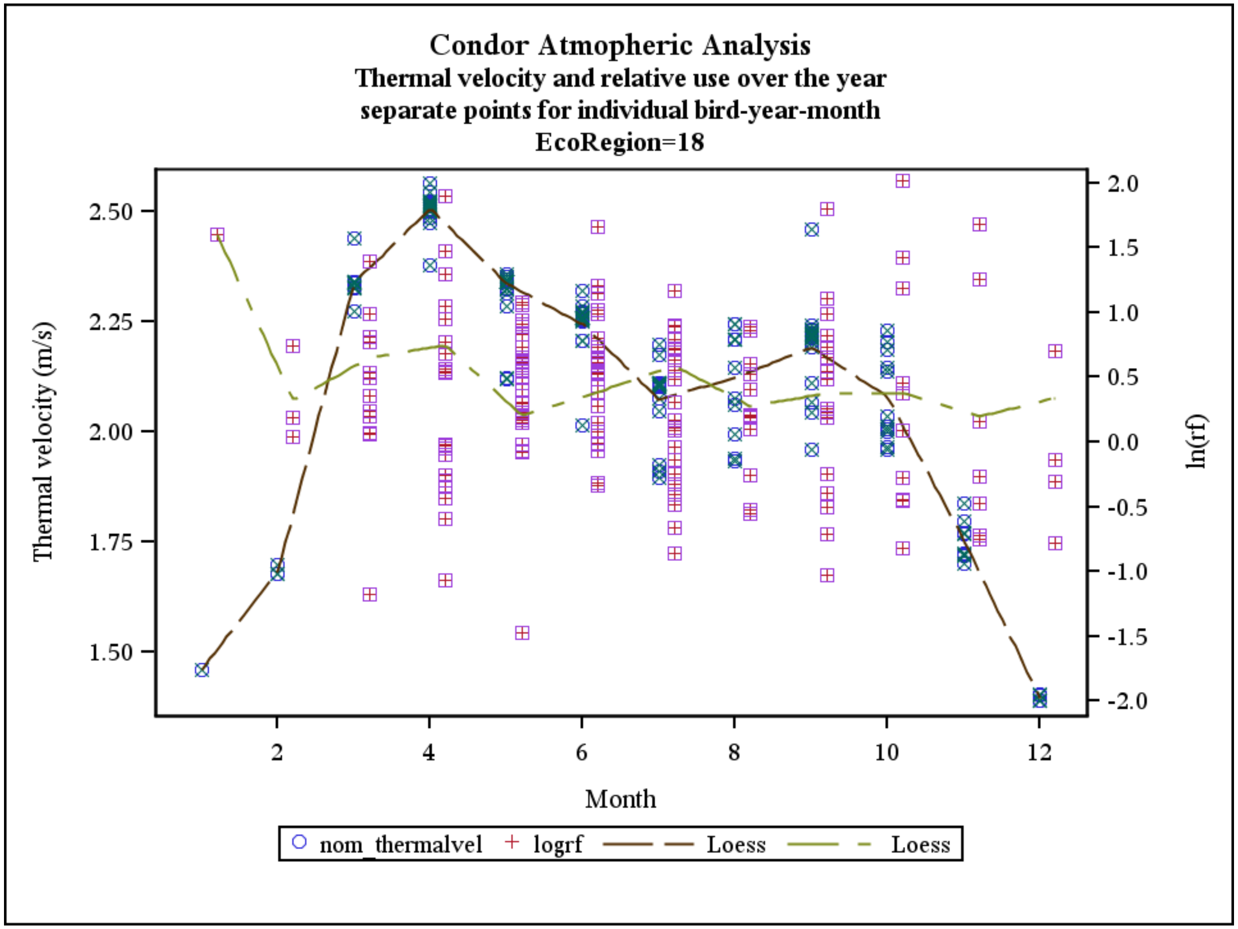

EcoRegion=39

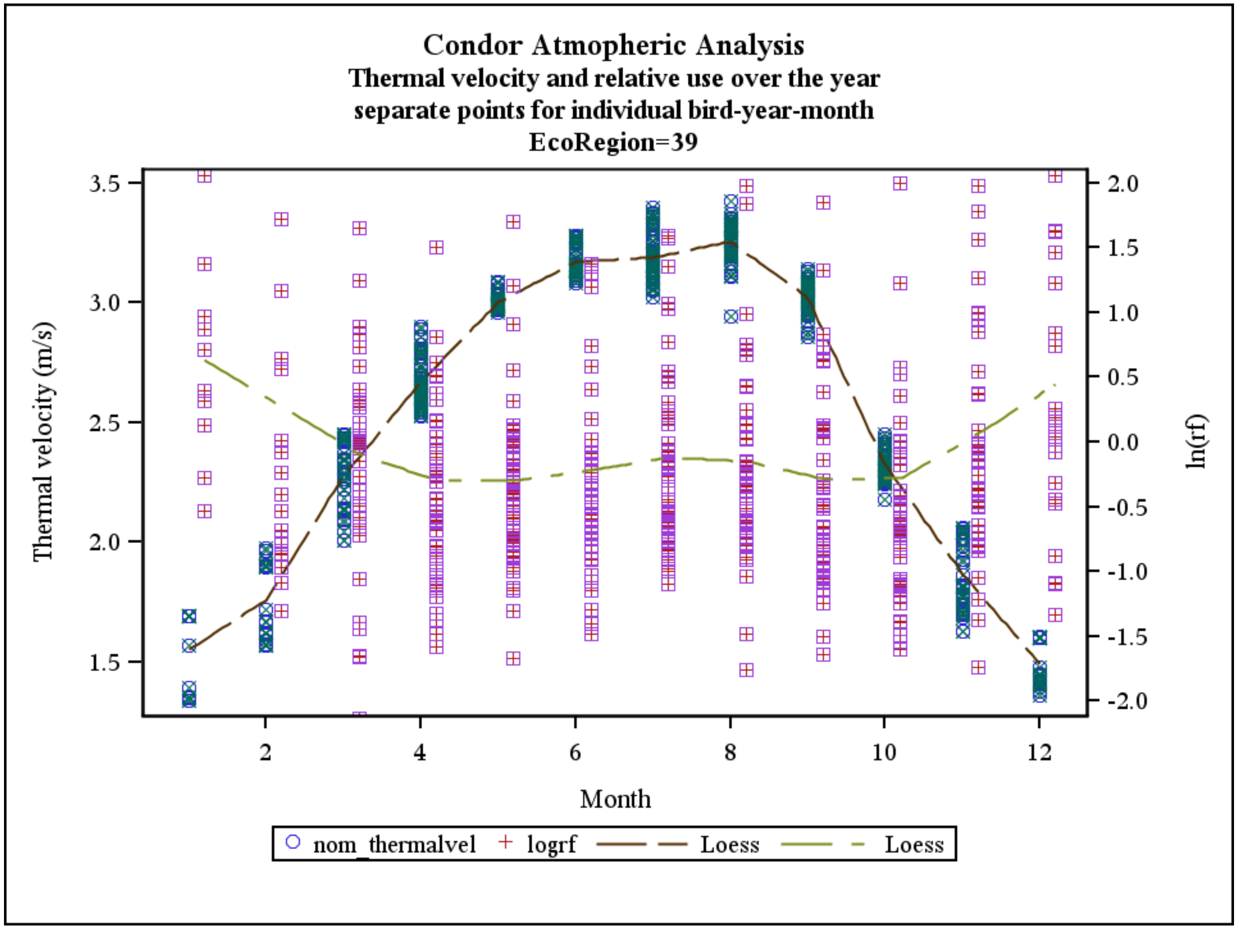

EcoRegion=40

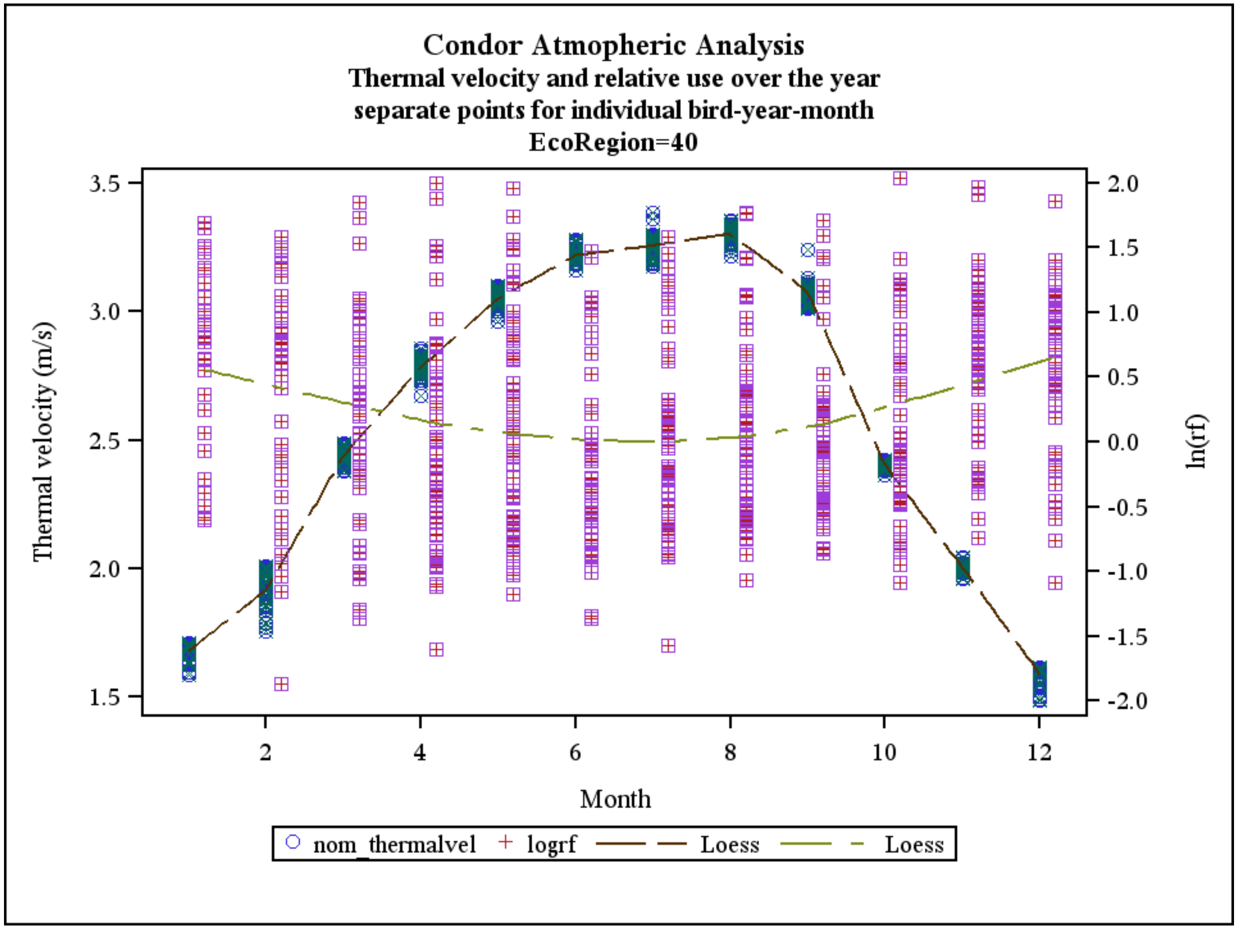

EcoRegion=95

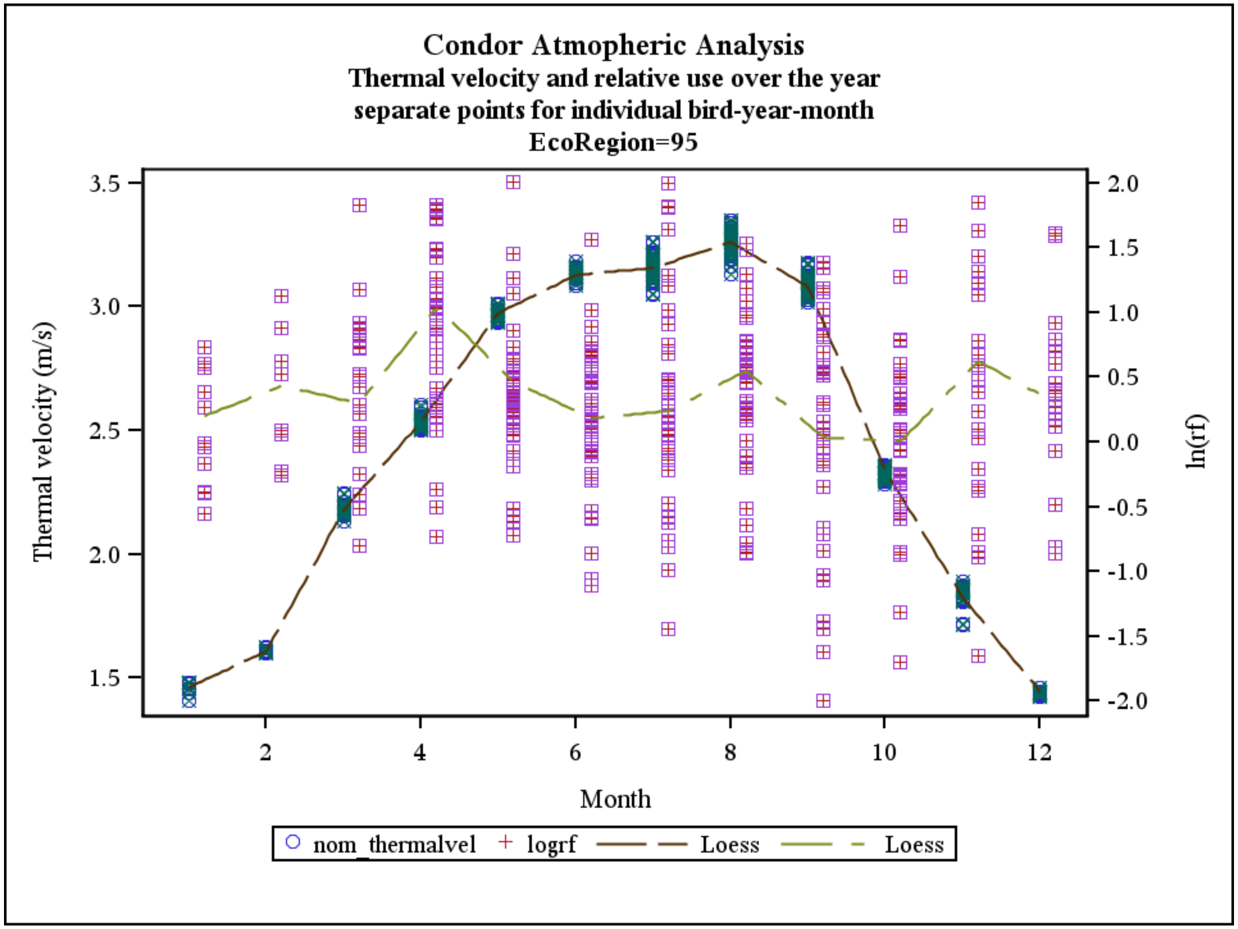

EcoRegion=101

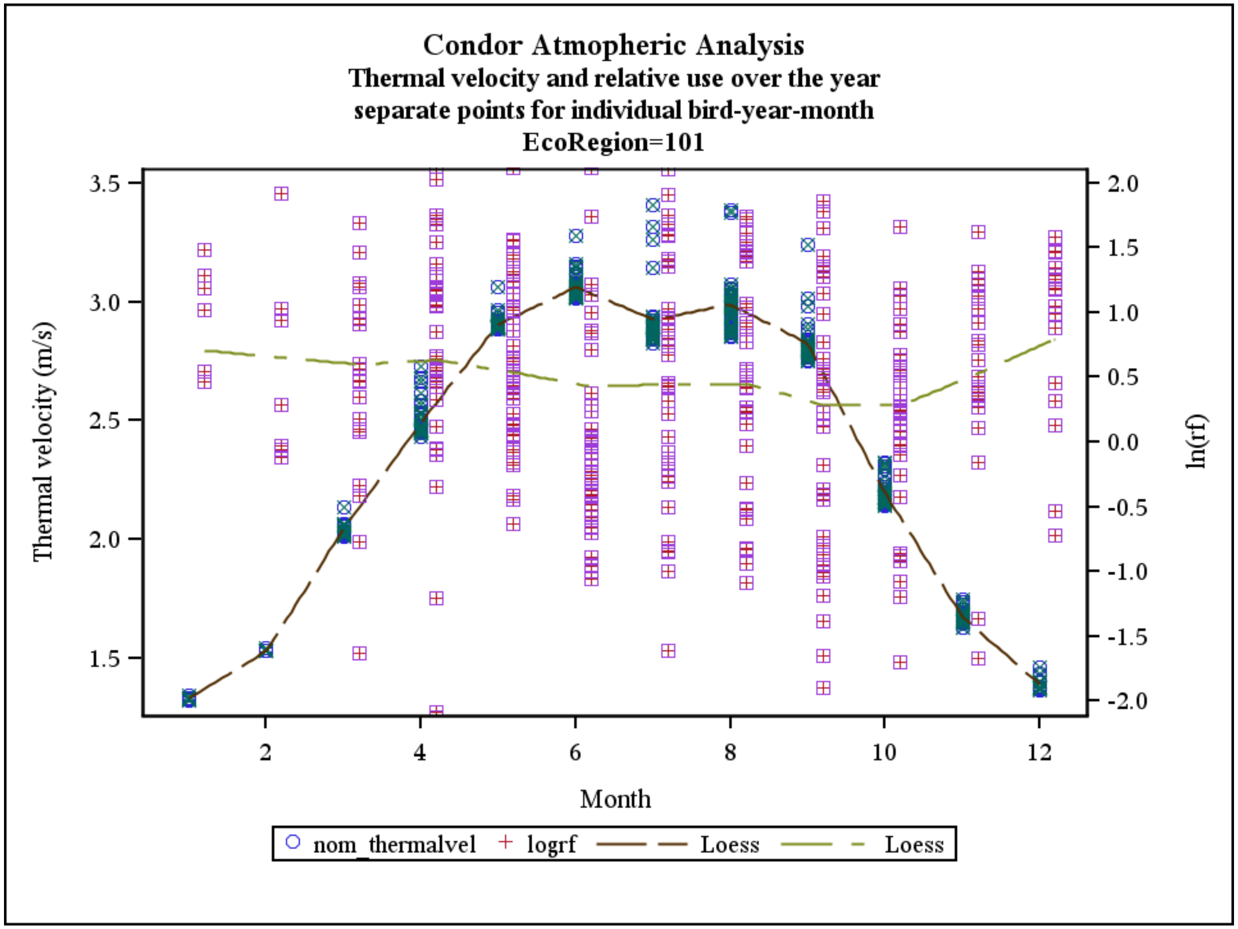

EcoRegion=102

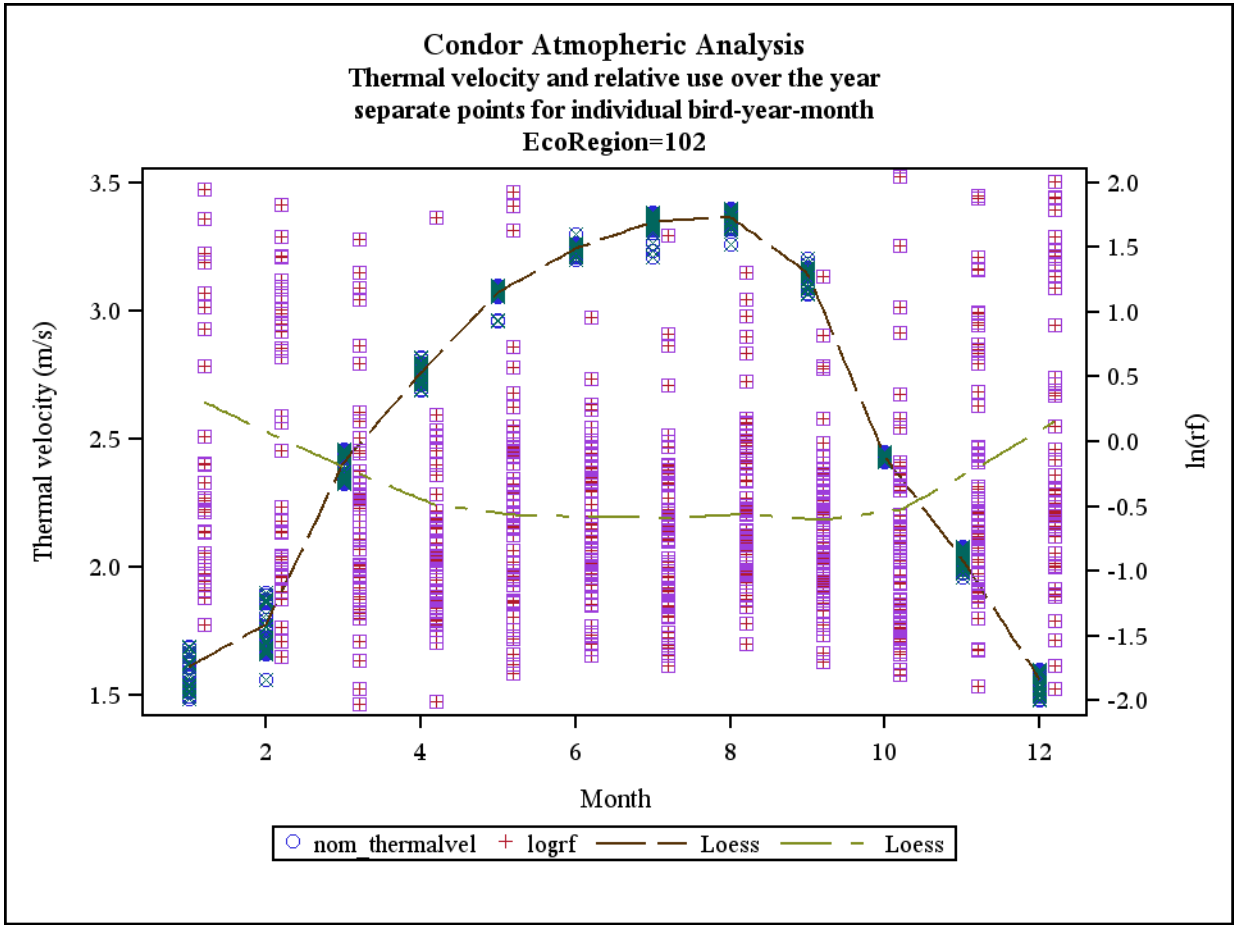

EcoRegion=116

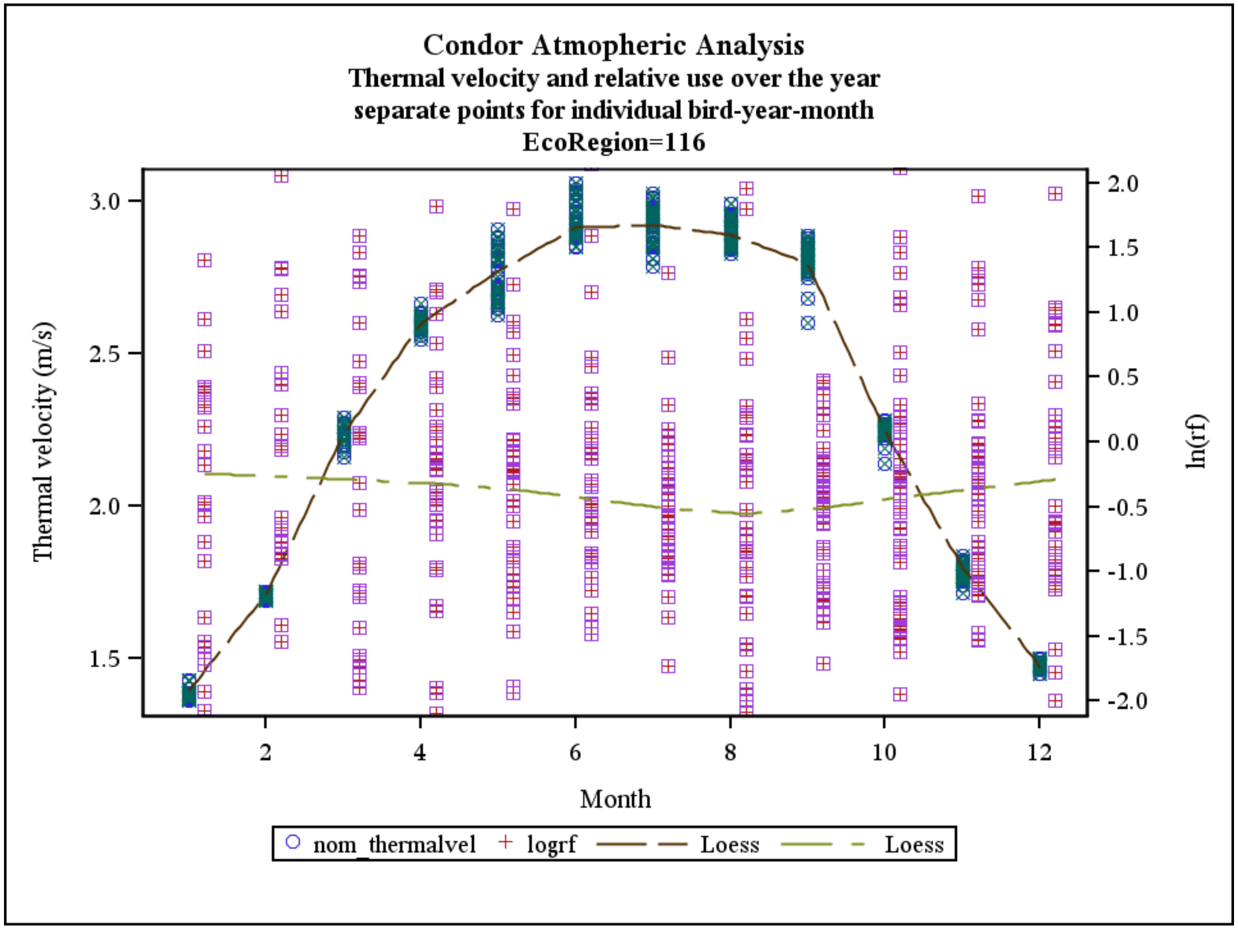

EcoRegion=117

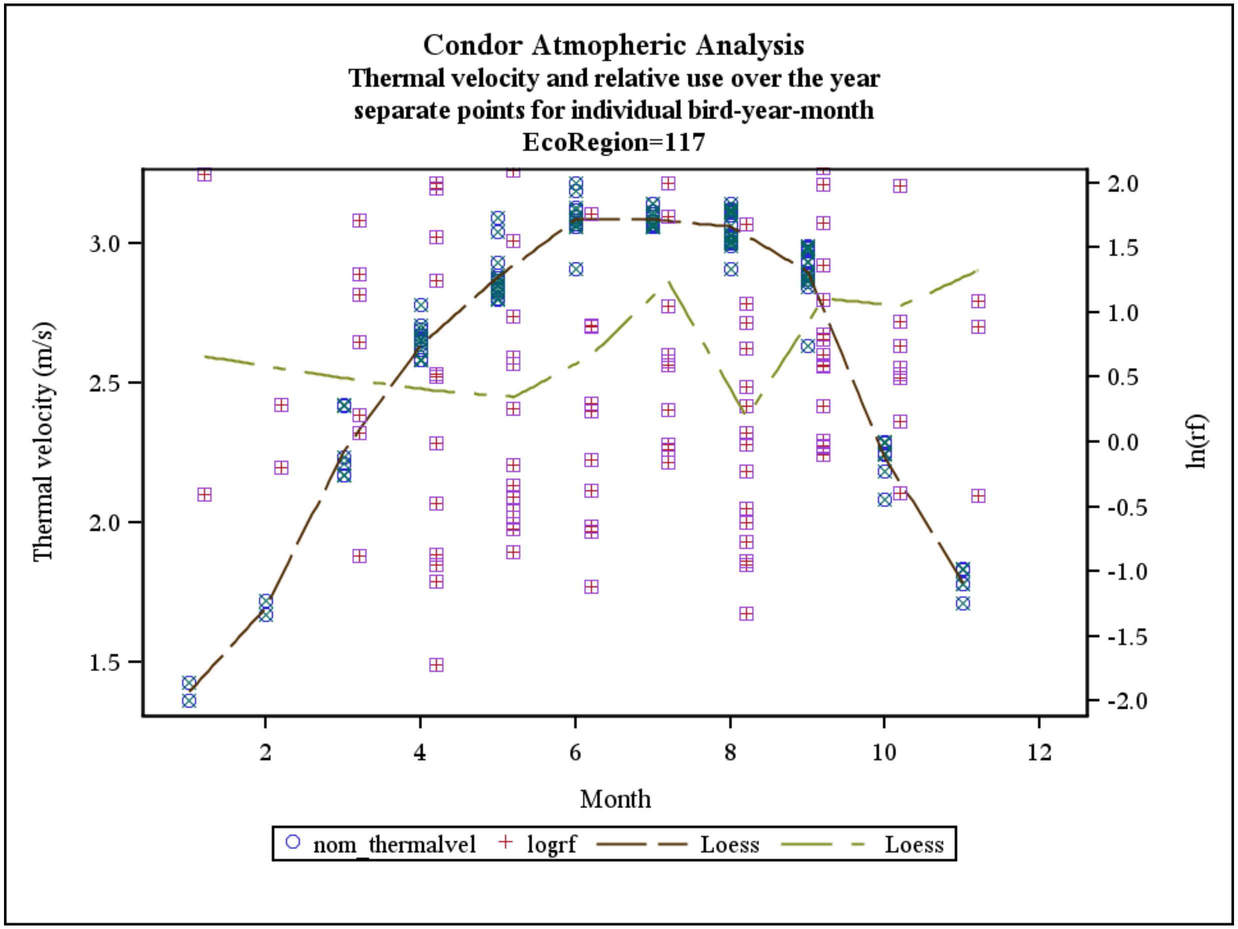

EcoRegion=118

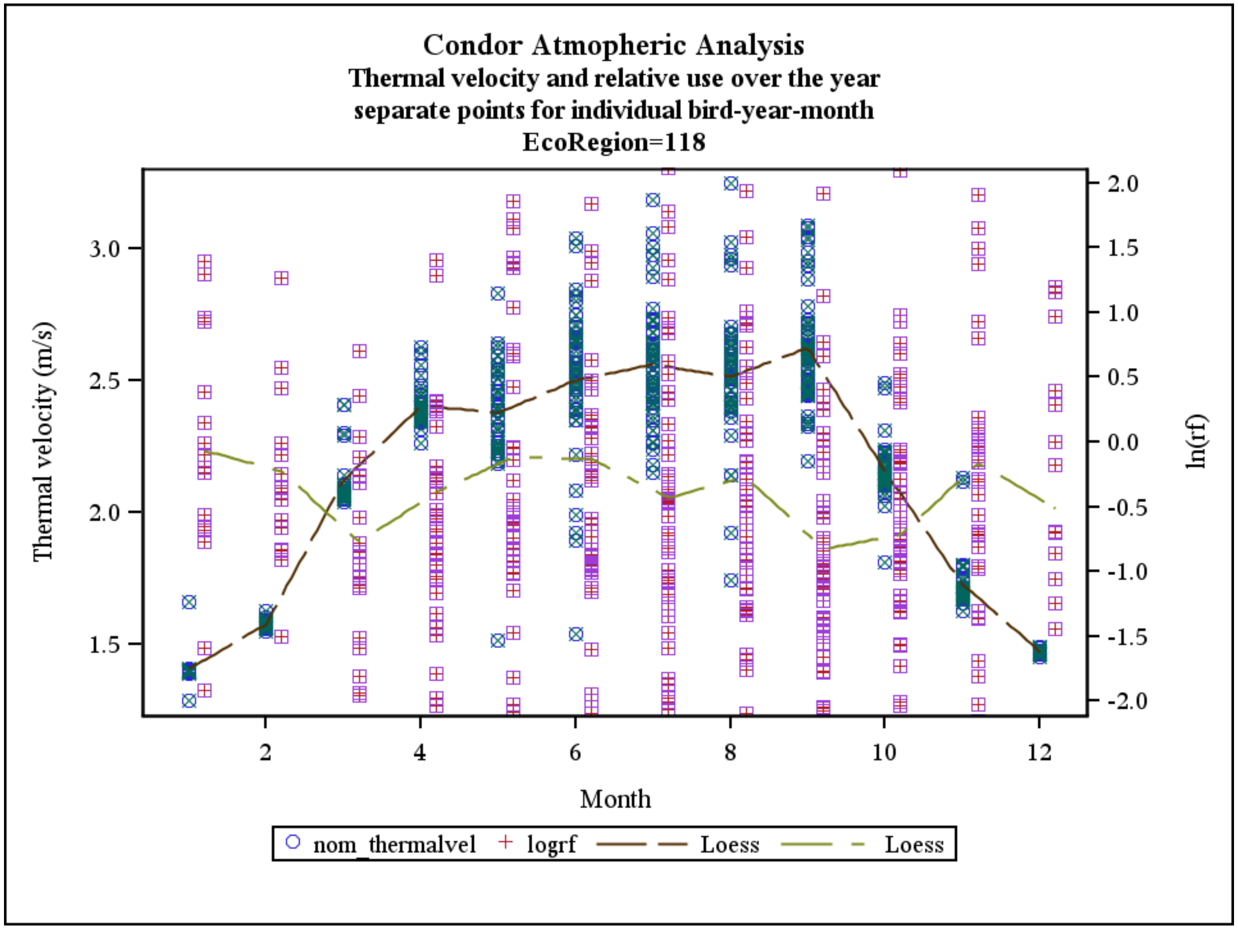

EcoRegion=119

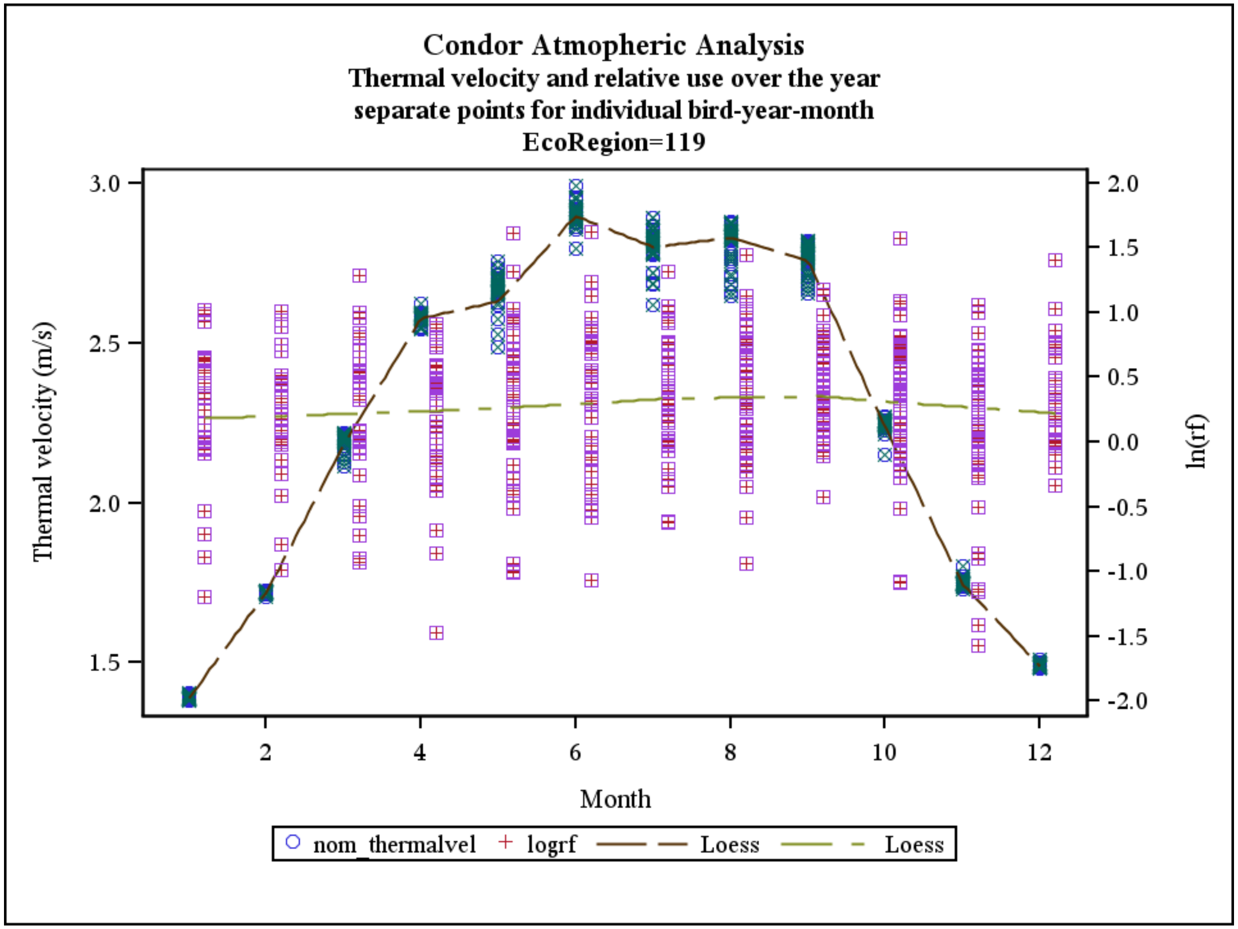

EcoRegion=123

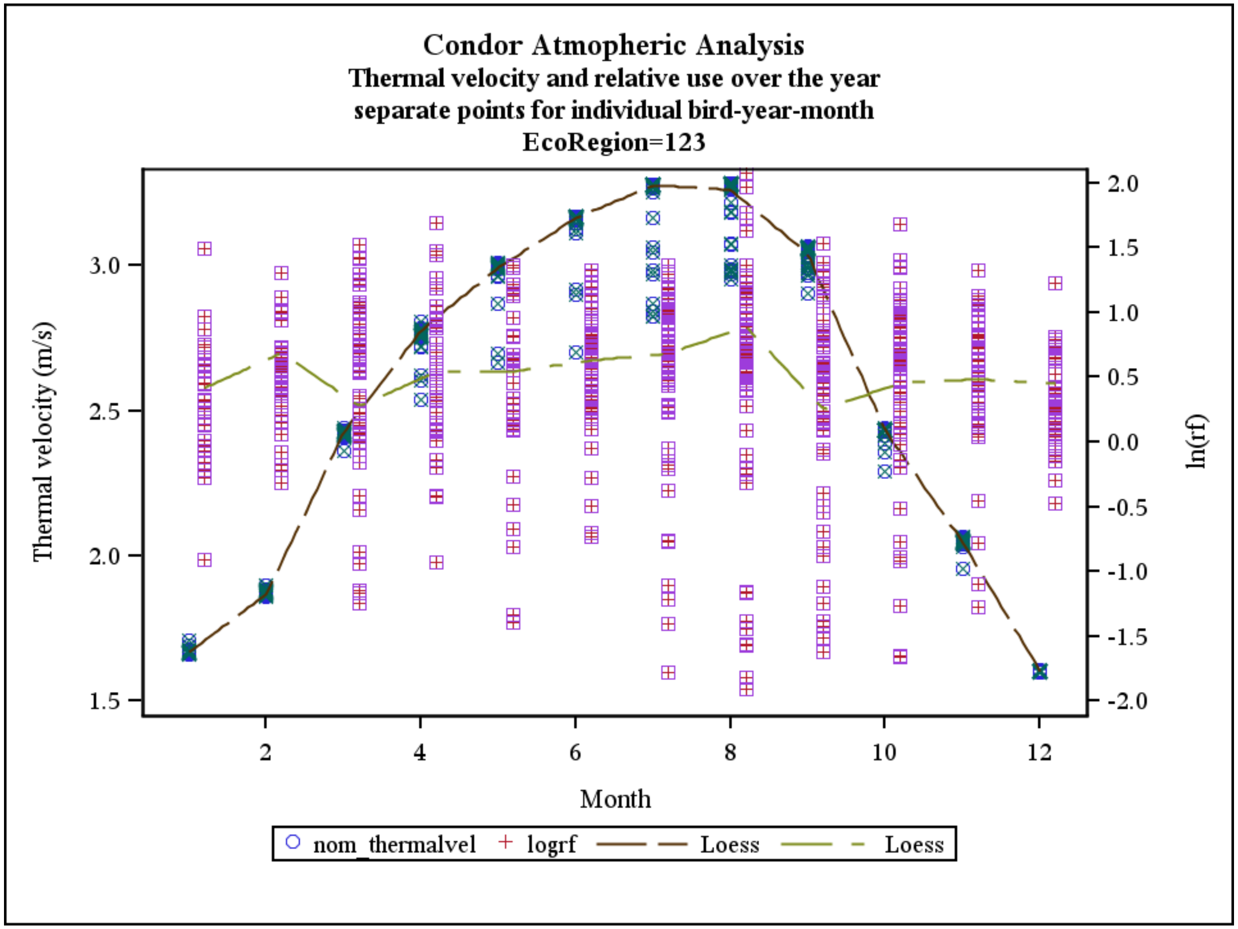

EcoRegion=124

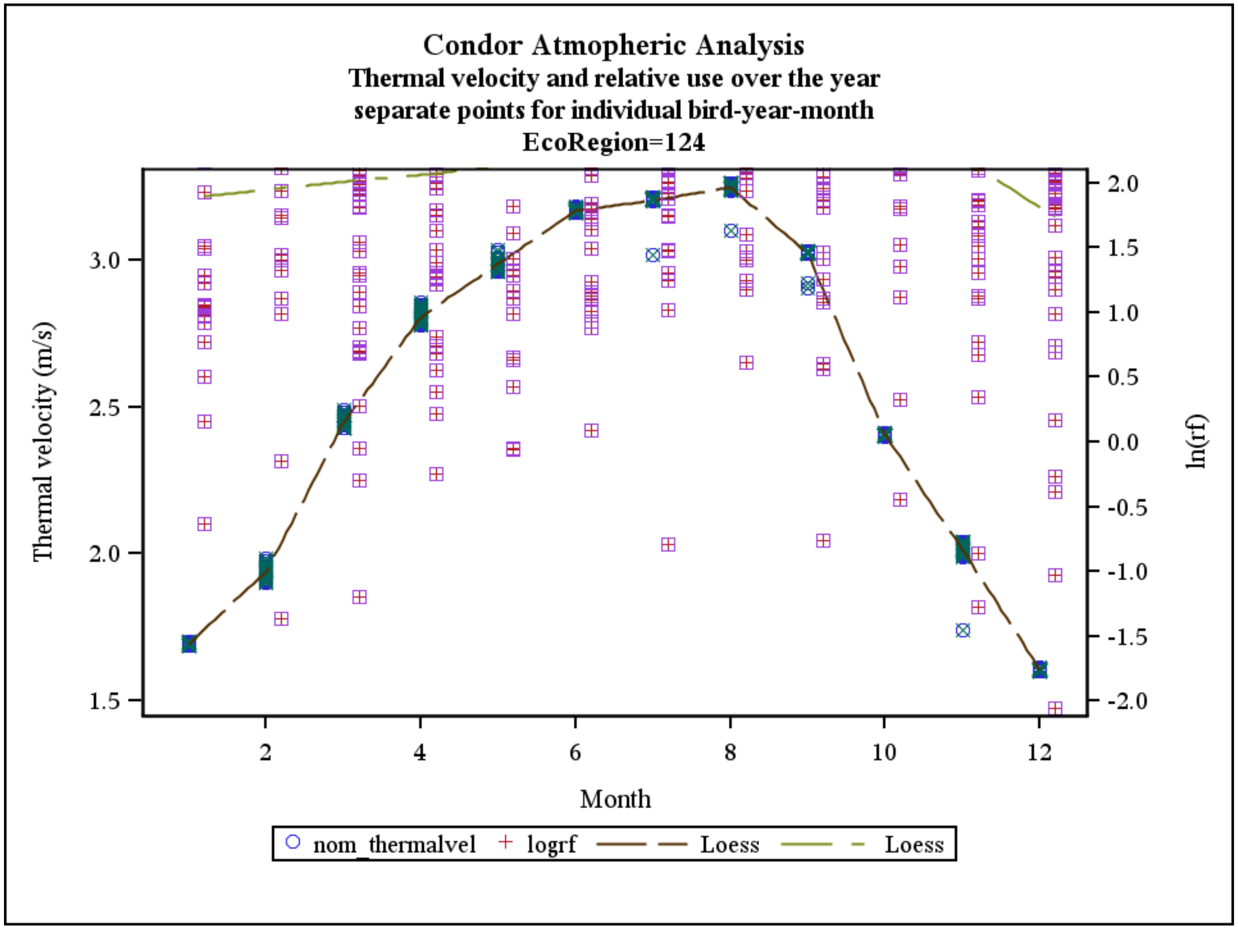

EcoRegion=125

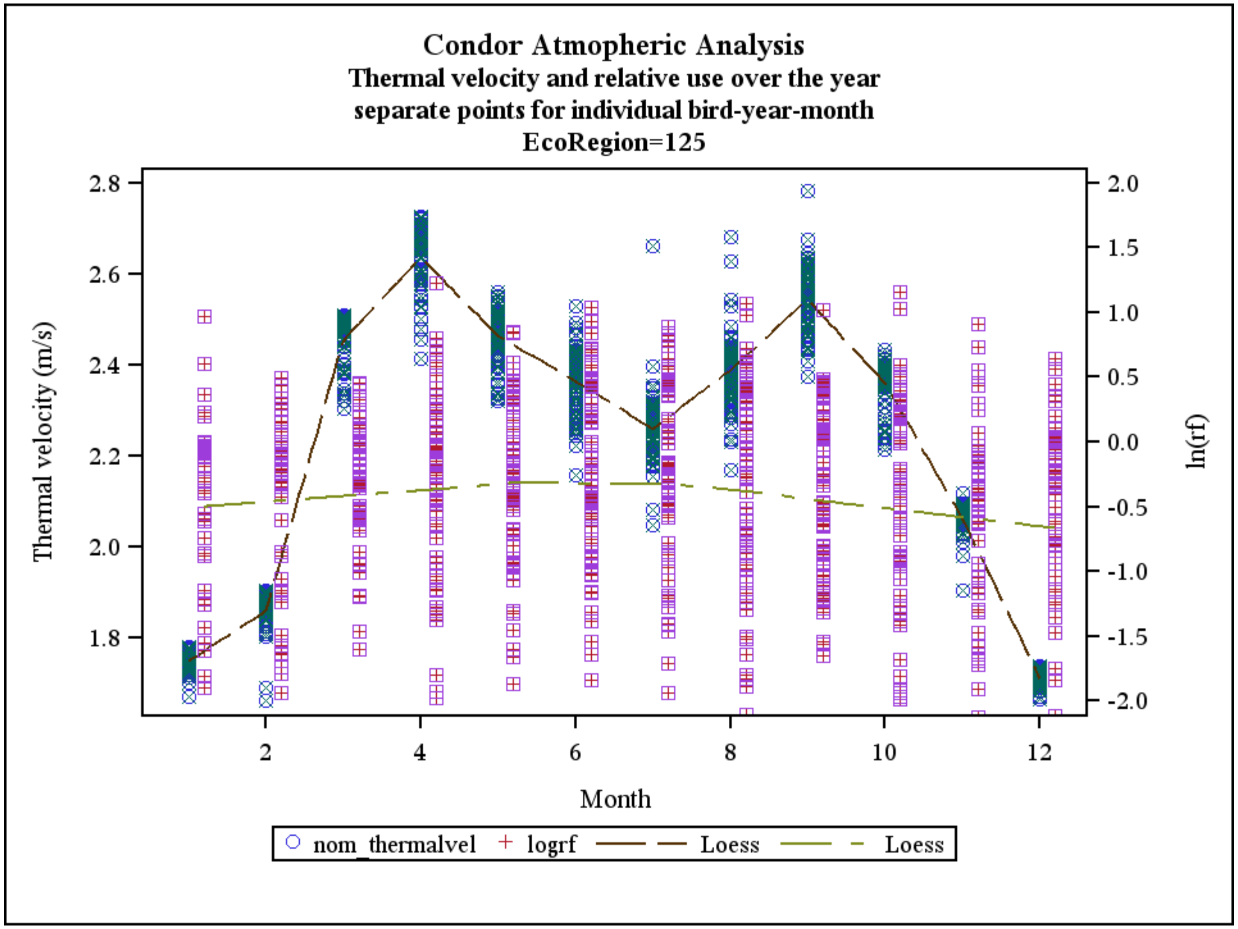

EcoRegion=126

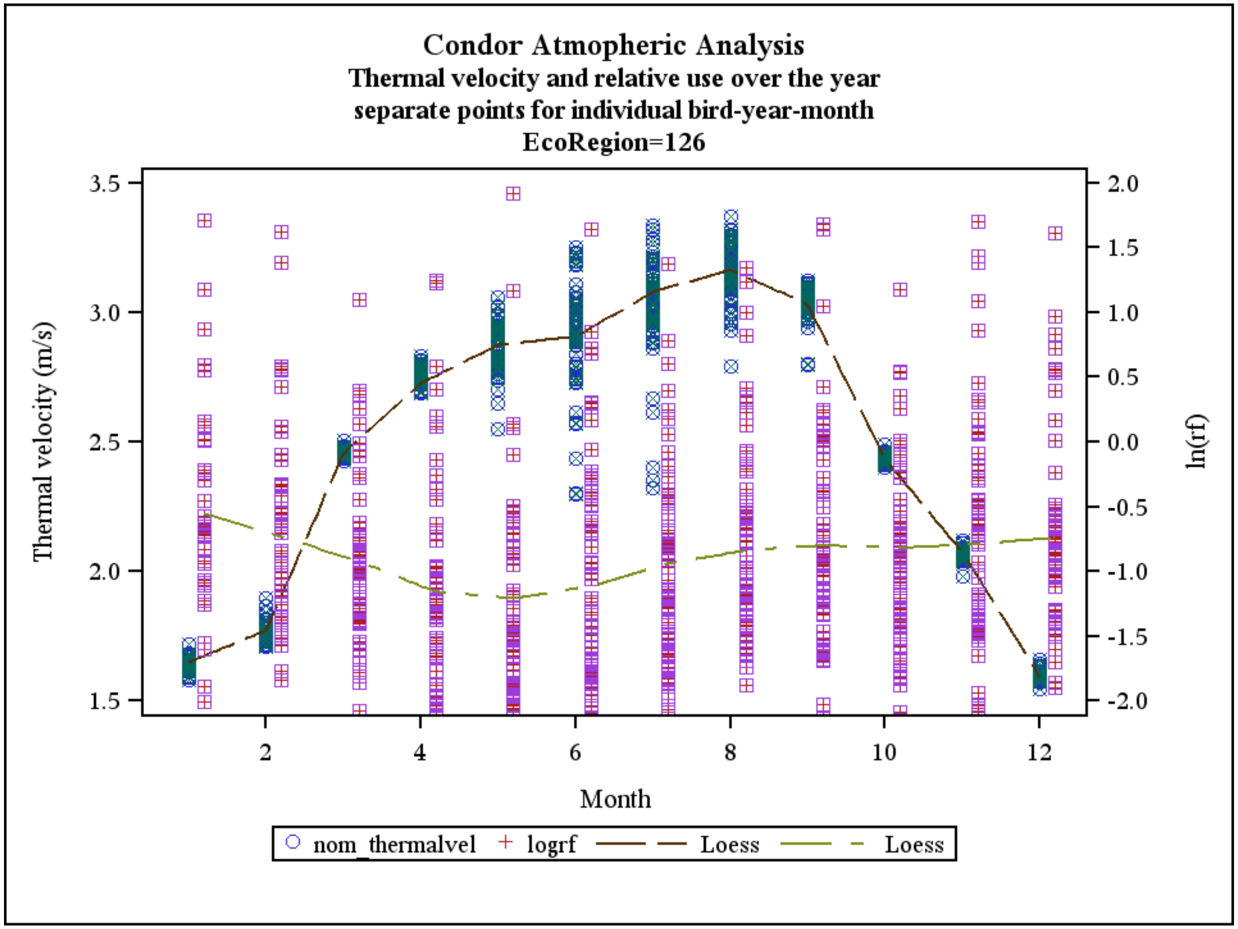

EcoRegion=127

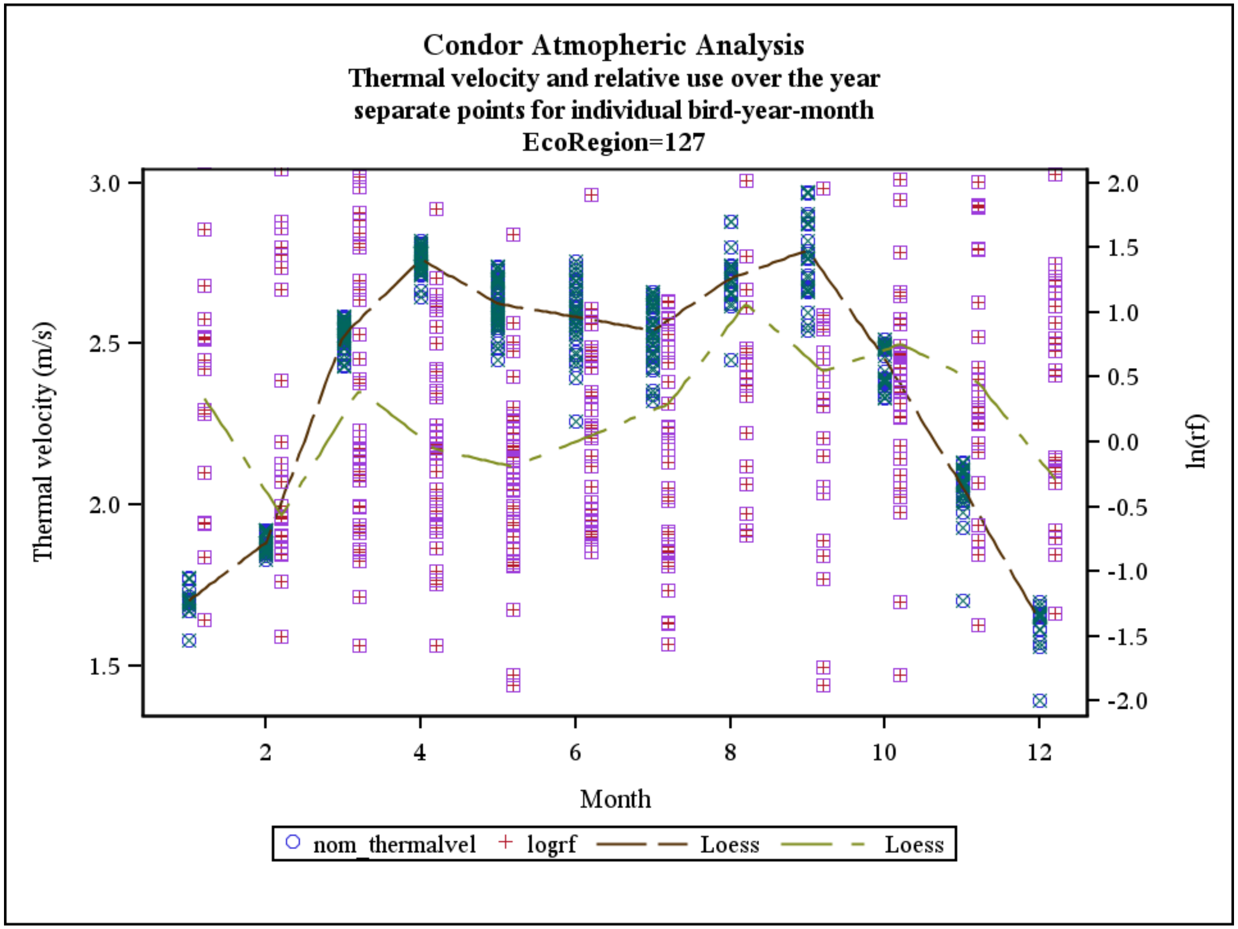

EcoRegion=128

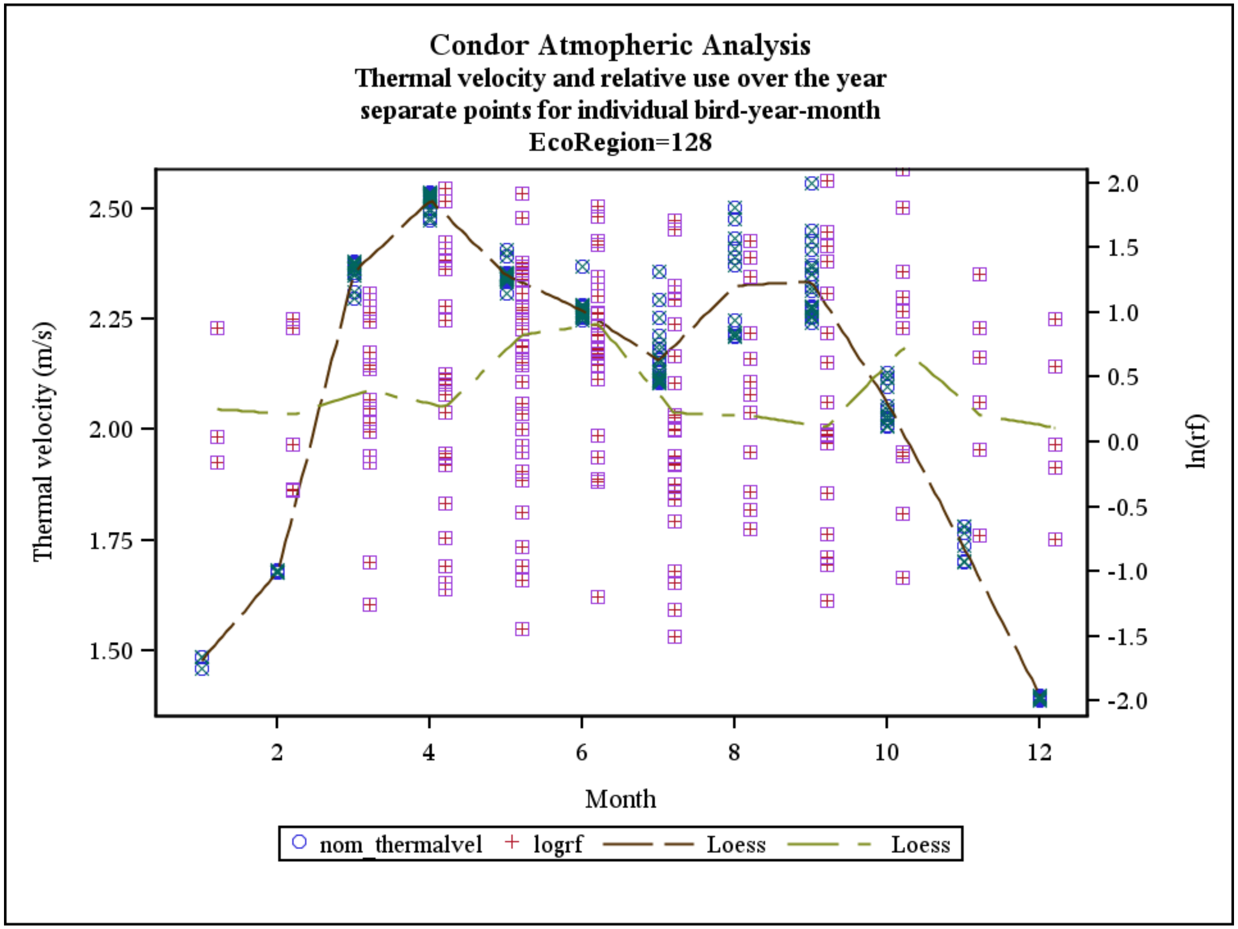

EcoRegion=147

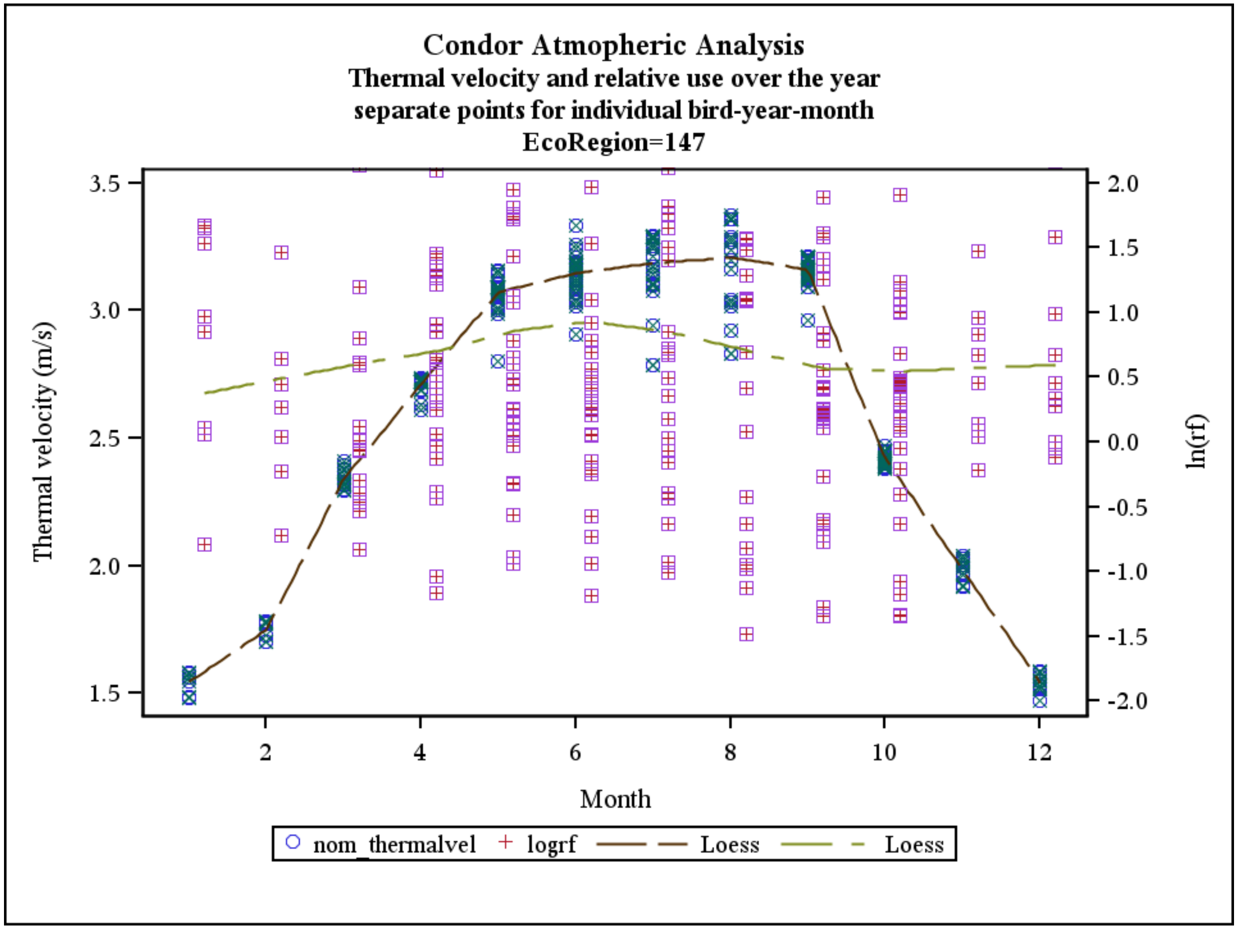

EcoRegion=192

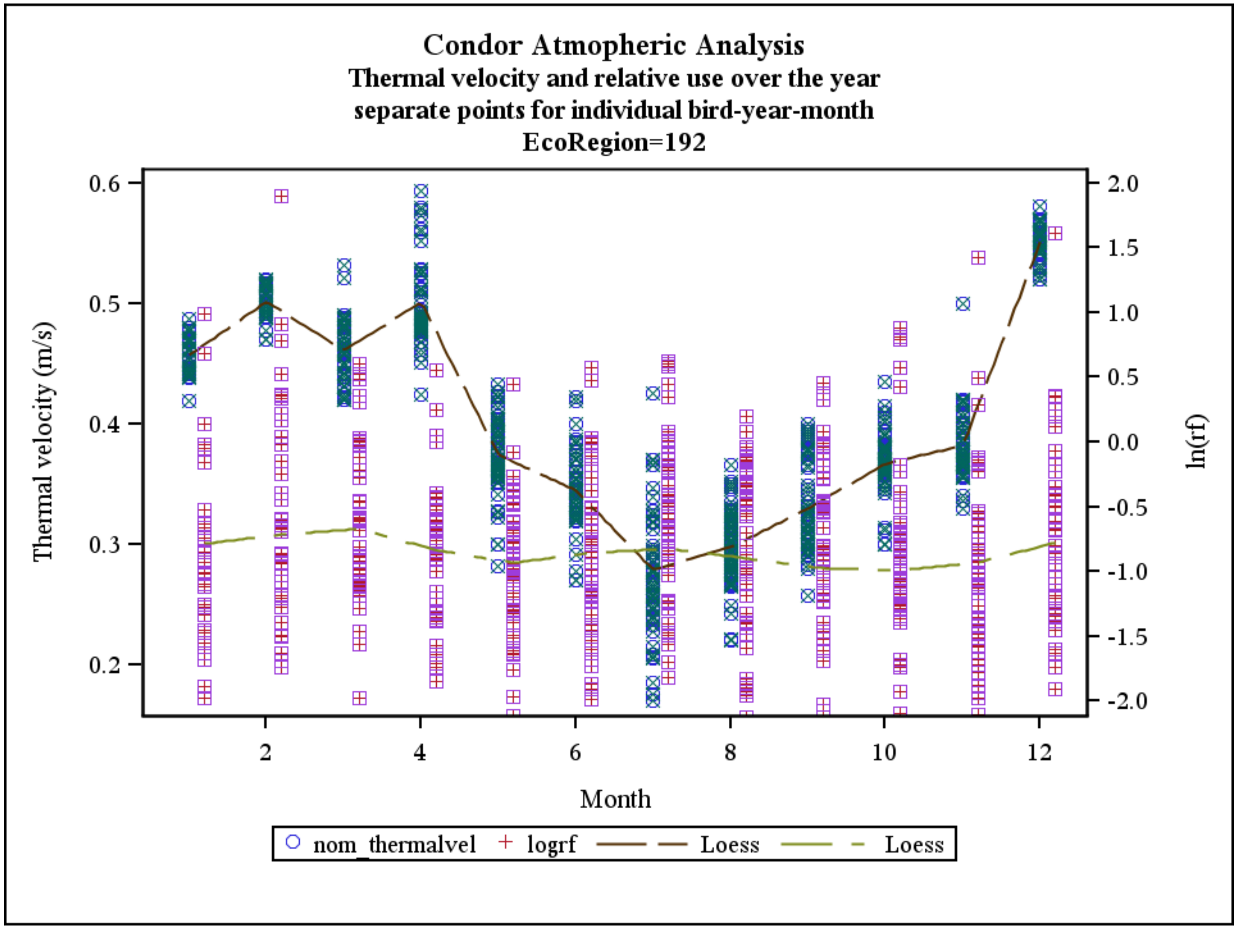

EcoRegion=193

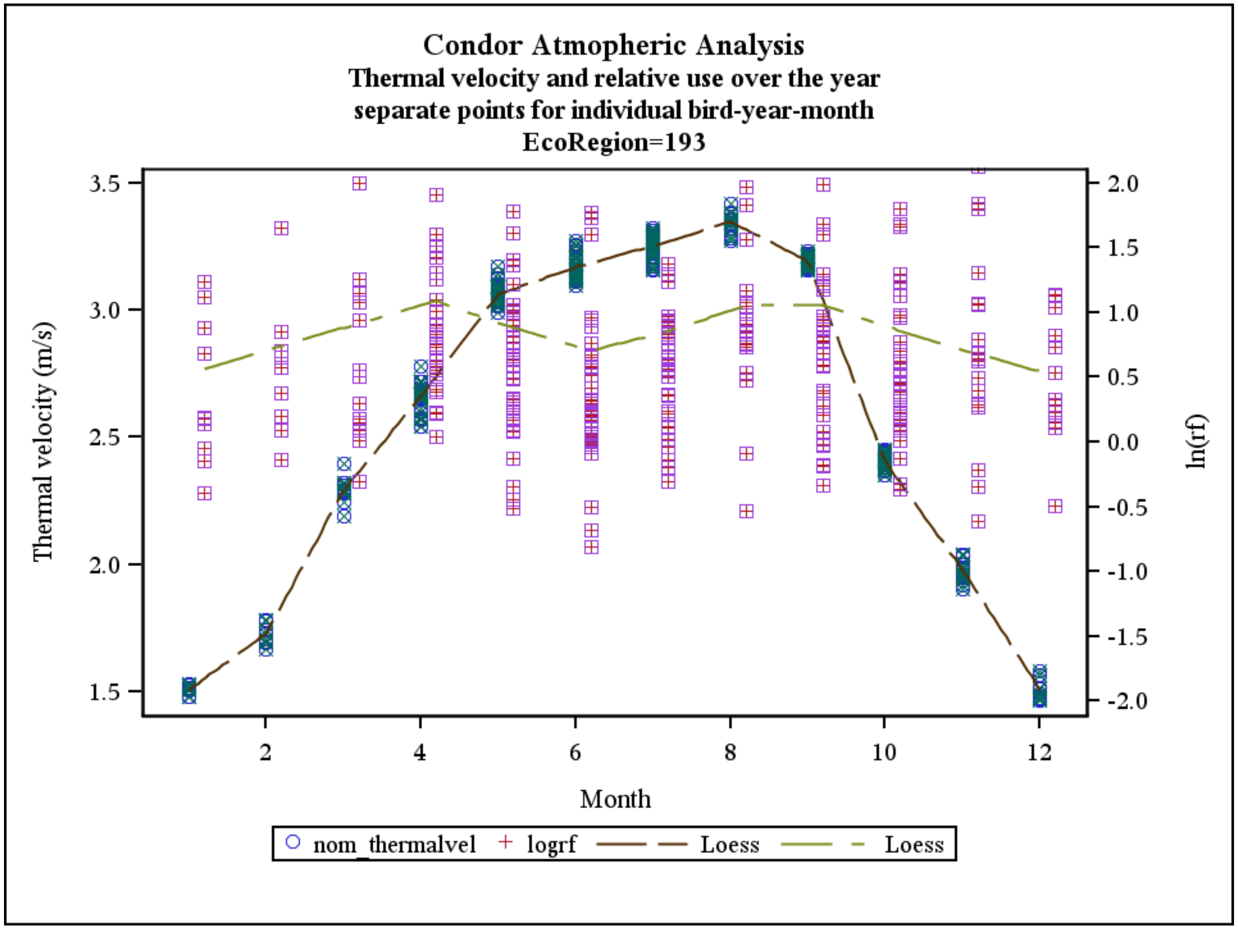

EcoRegion=8

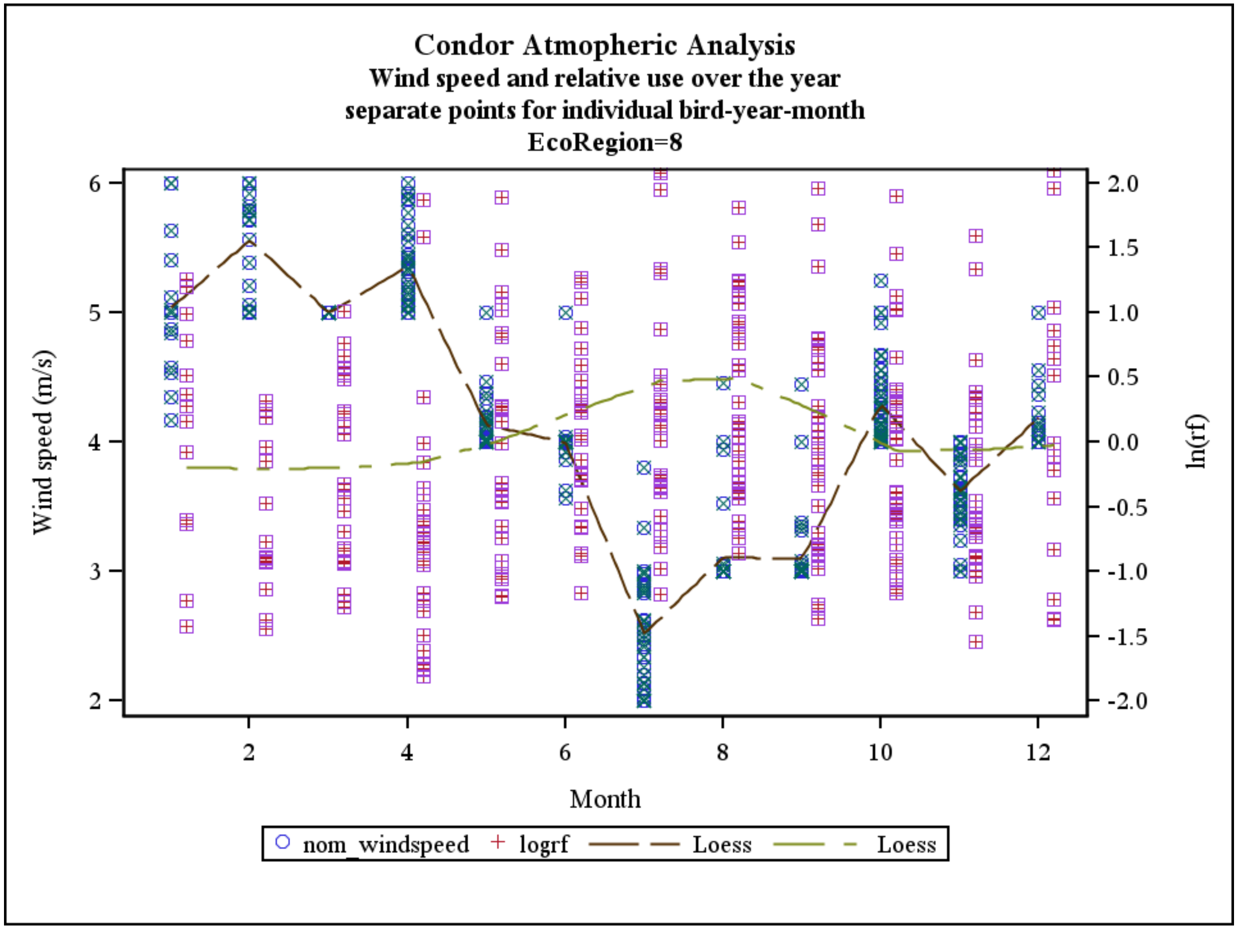
Condor Atmopheric Analysis
Wind speed and relative use over the year
separate points for individual bird-year-month
EcoRegion=8
Wind speed (m/s)
ln(rf)
Month
2
3
4
5
6
2
4
6
8
10
12
2.0
1.5
1.0
0.5
0.0
-0.5
-1.0
-1.5
-2.0
nom_windspeed
logrf
Loess
Loess

EcoRegion=9

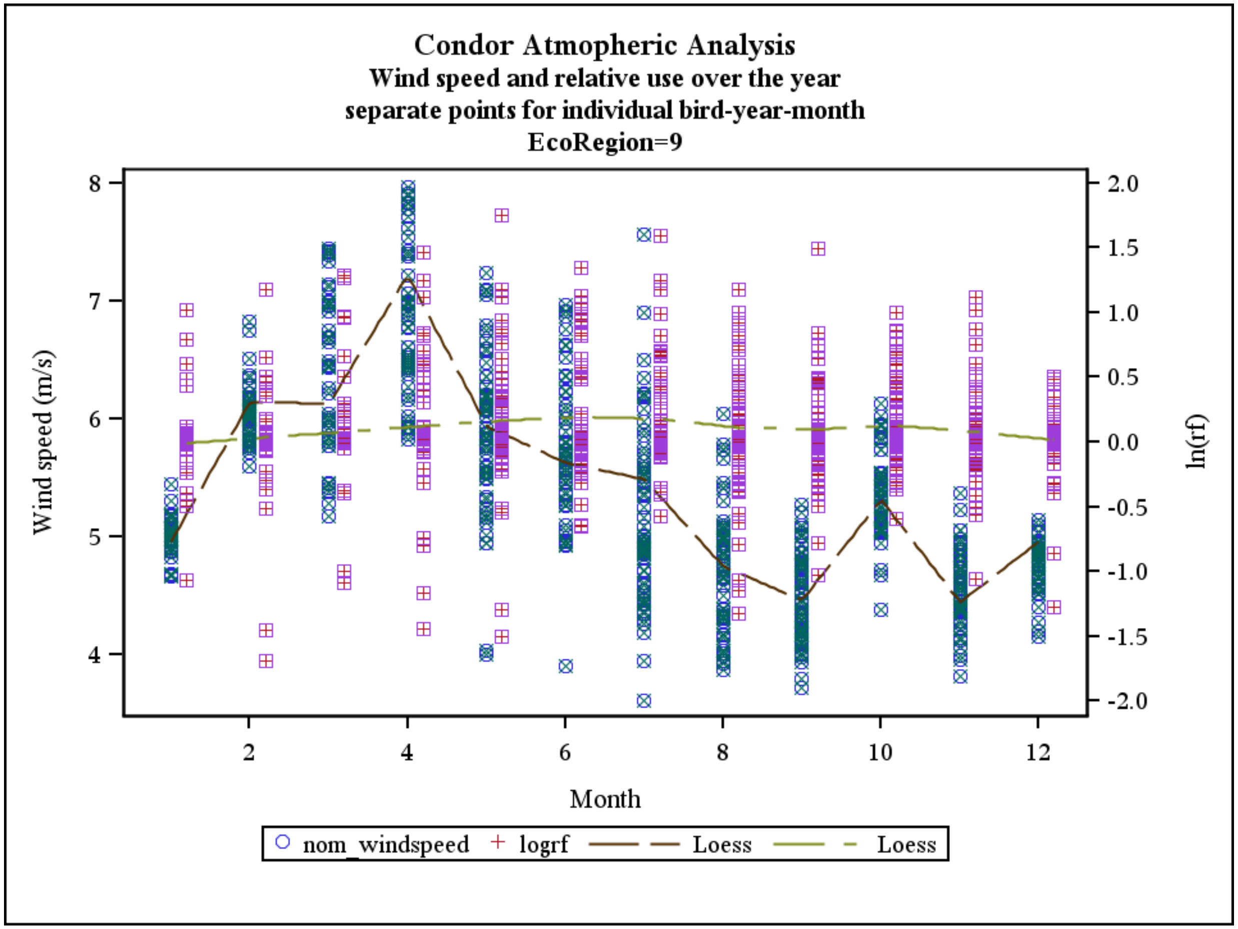
Condor Atmopheric Analysis
Wind speed and relative use over the year
separate points for individual bird-year-month
EcoRegion=9
Wind speed (m/s)
8
7
6
5
4
2.0
1.5
1.0
0.5
0.0
-0.5
-1.0
-1.5
-2.0
ln(rf)
2
4
6
8
10
12
Month
nom_windspeed
logrf
Loess
Loess

EcoRegion=10

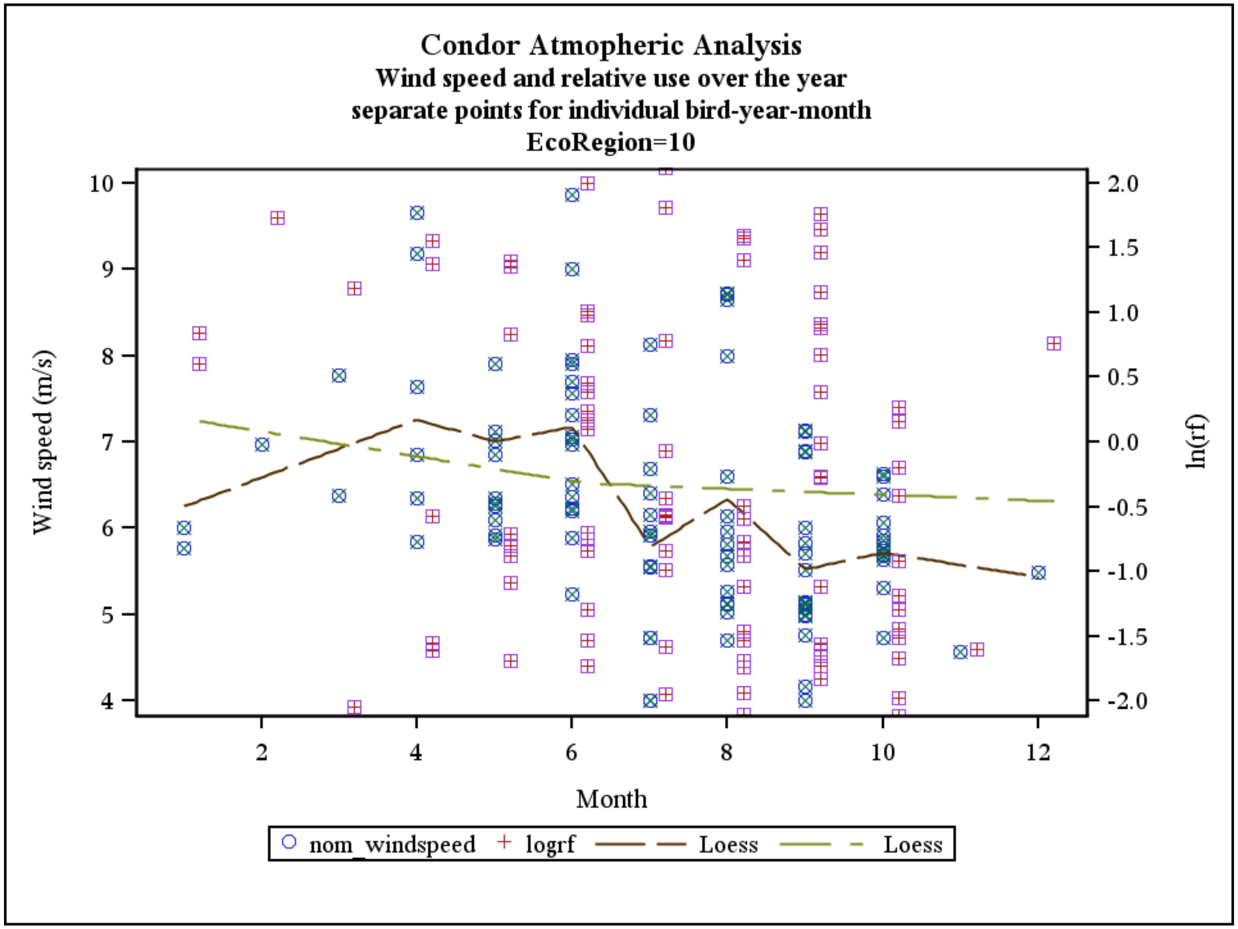

EcoRegion=13

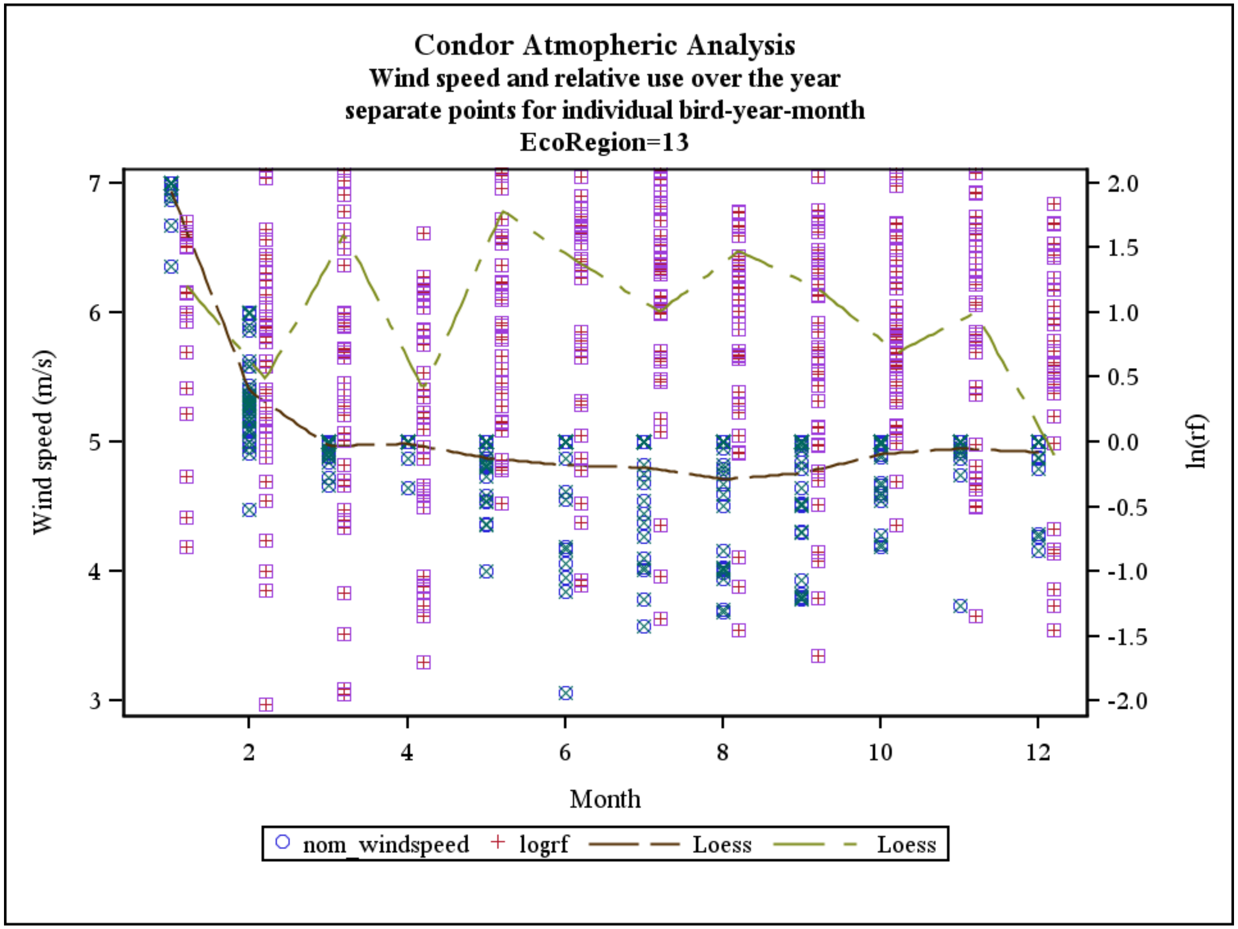

EcoRegion=15

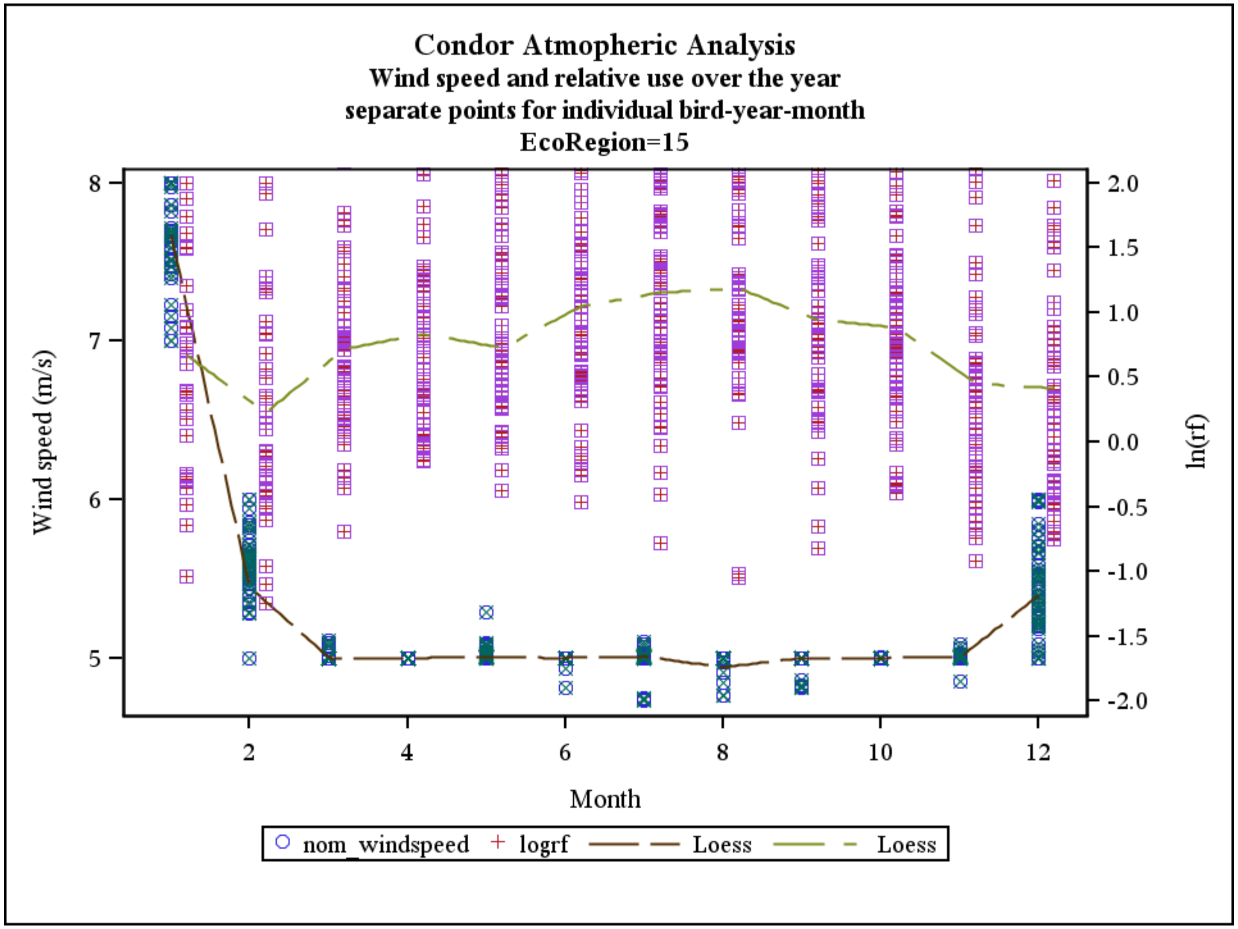

EcoRegion=16

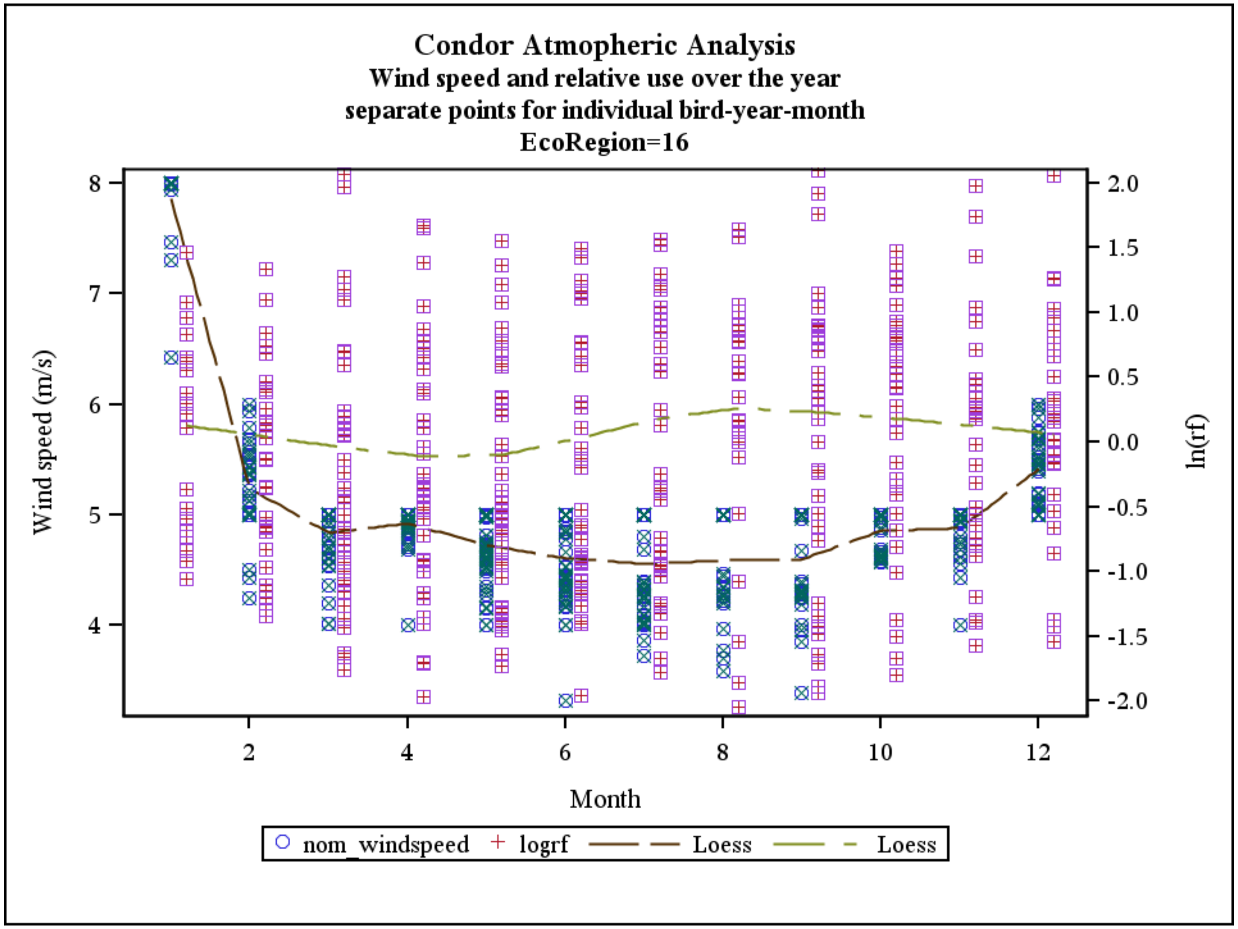

EcoRegion=18

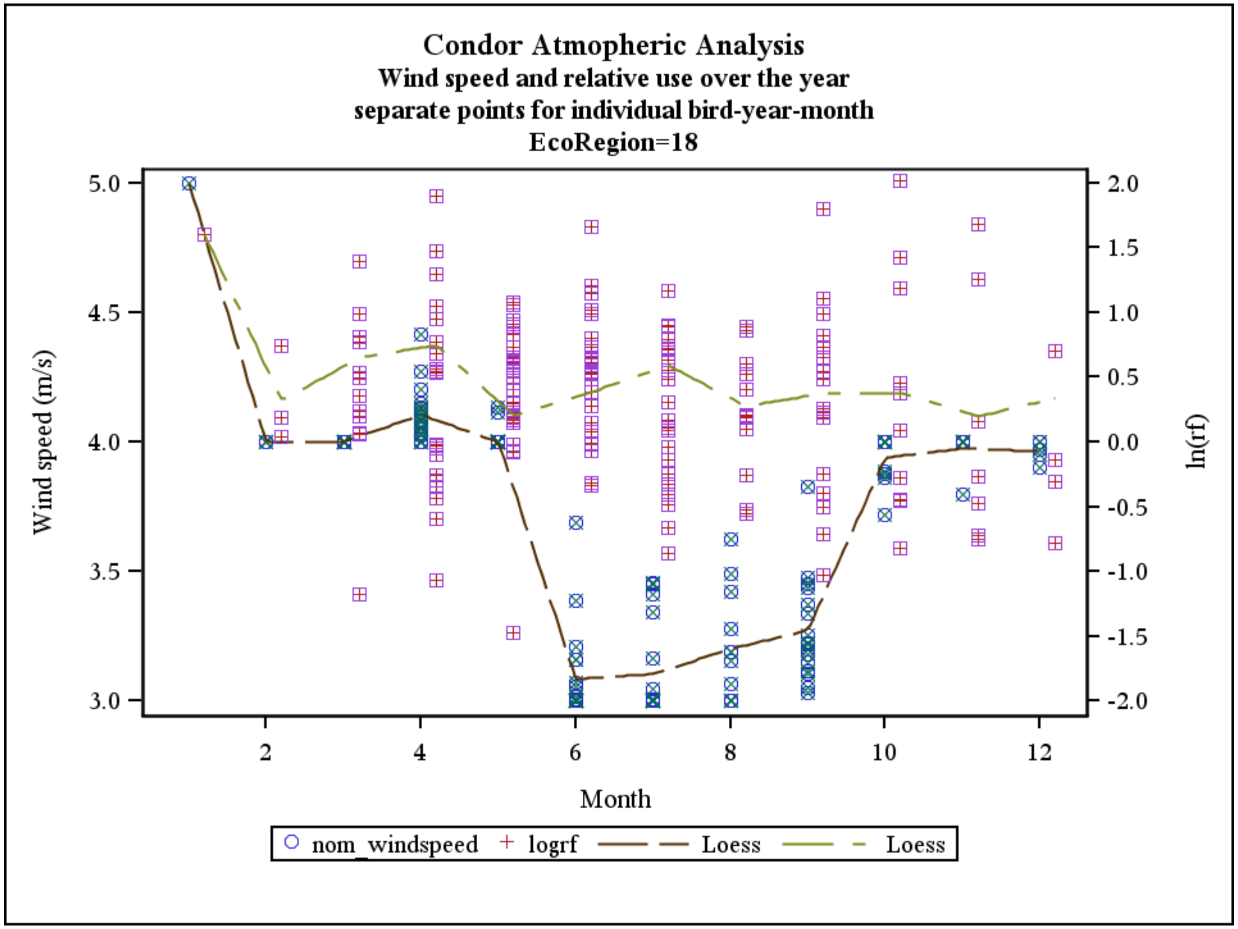

EcoRegion=39

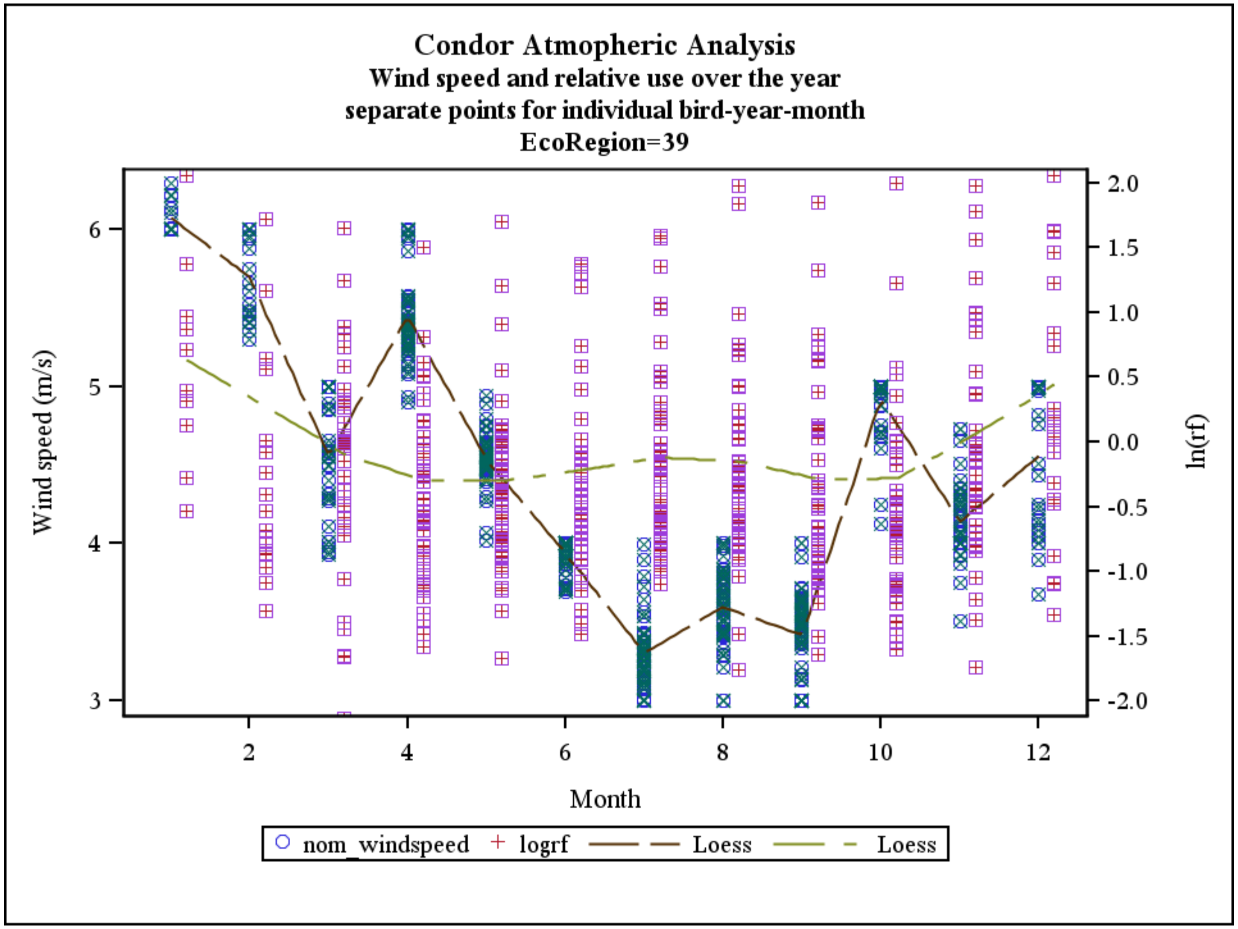

EcoRegion=40

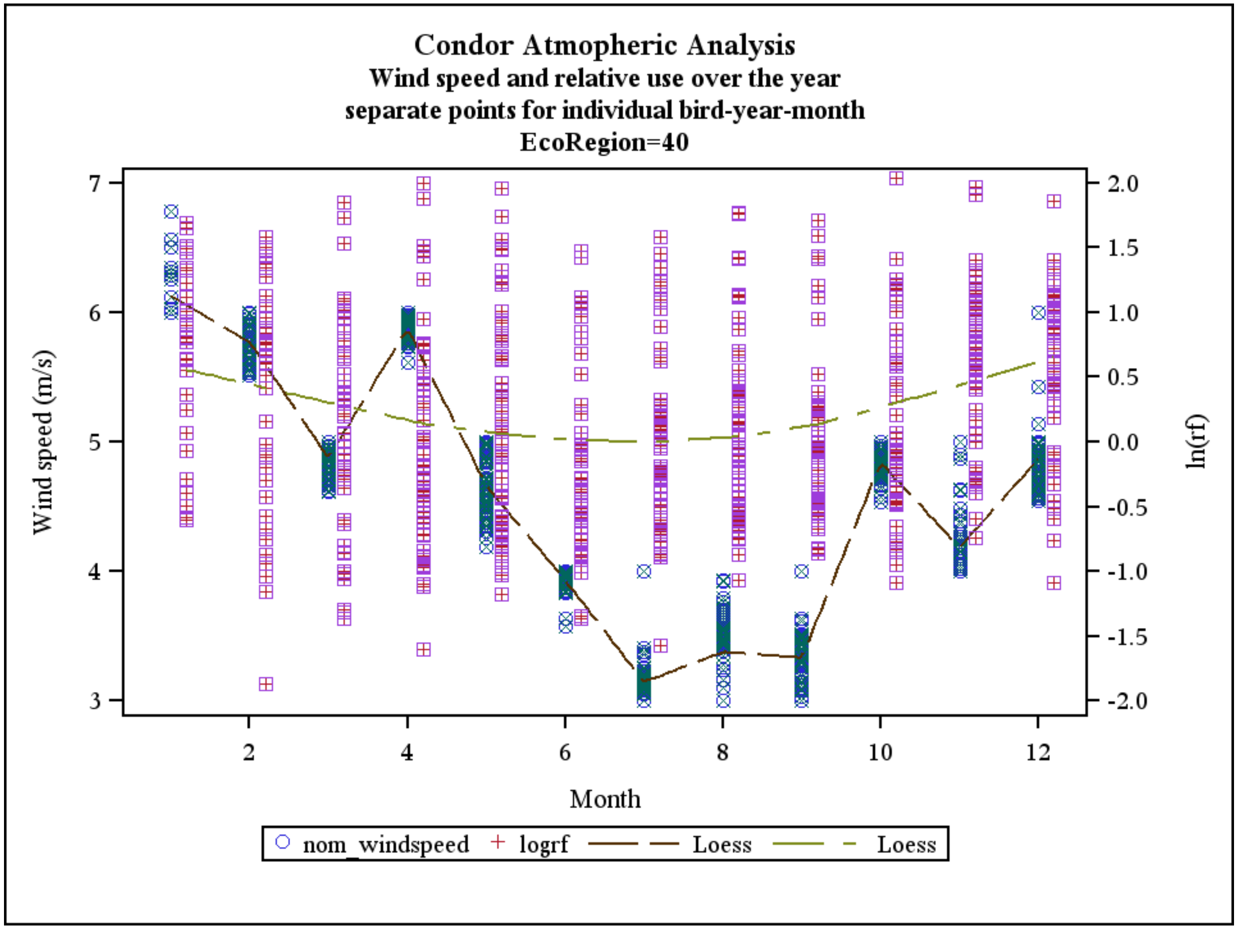

EcoRegion=95

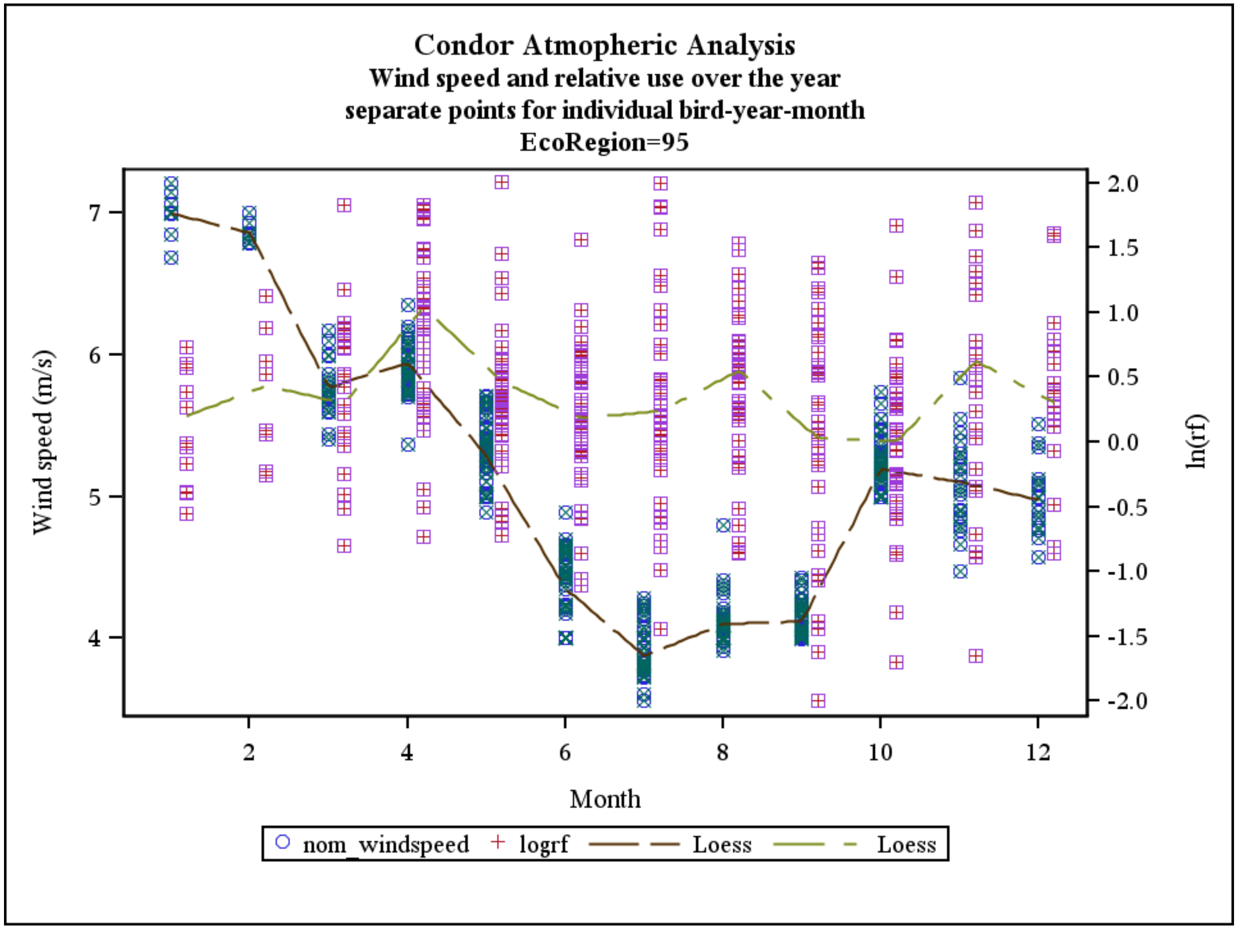

EcoRegion=101

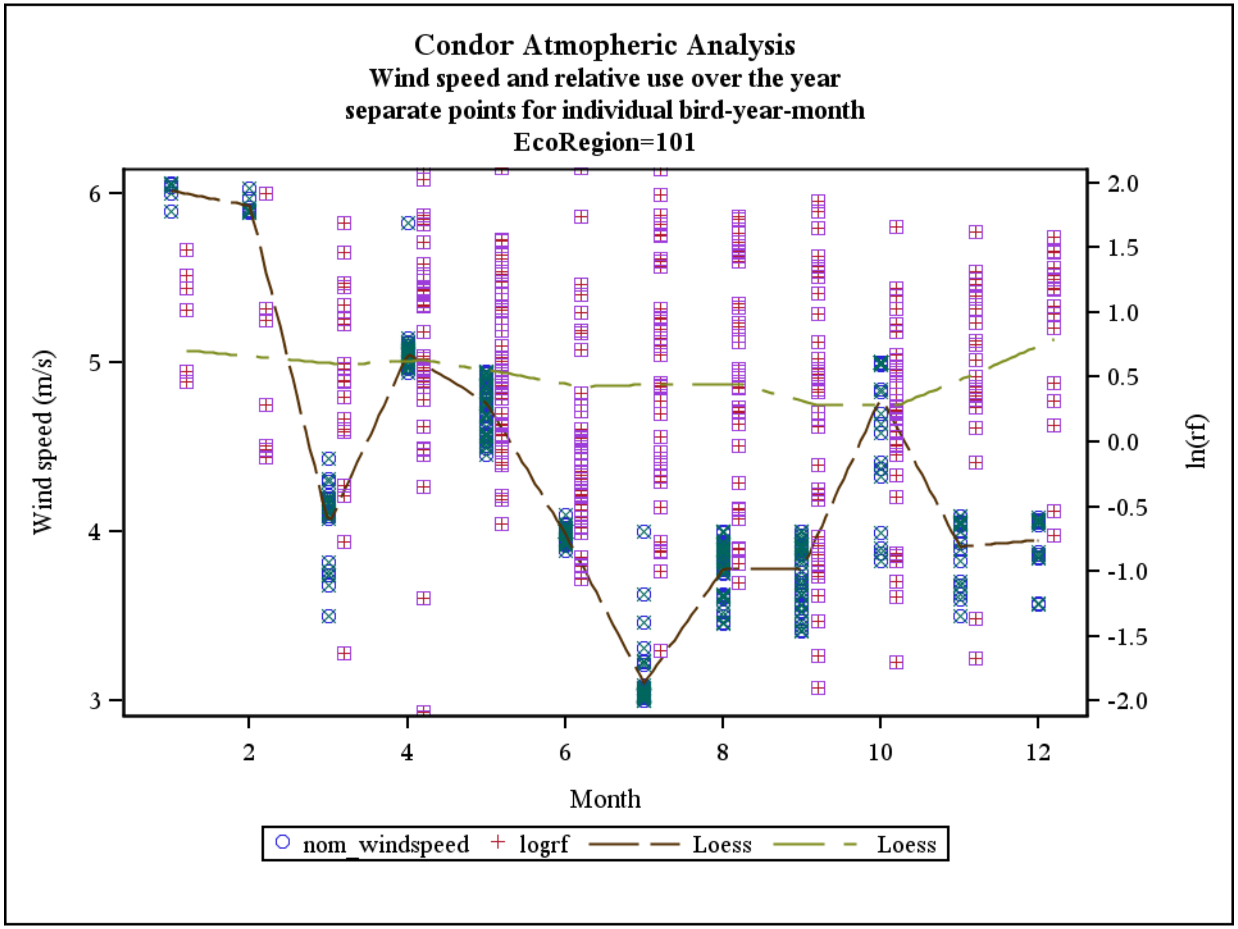

EcoRegion=102

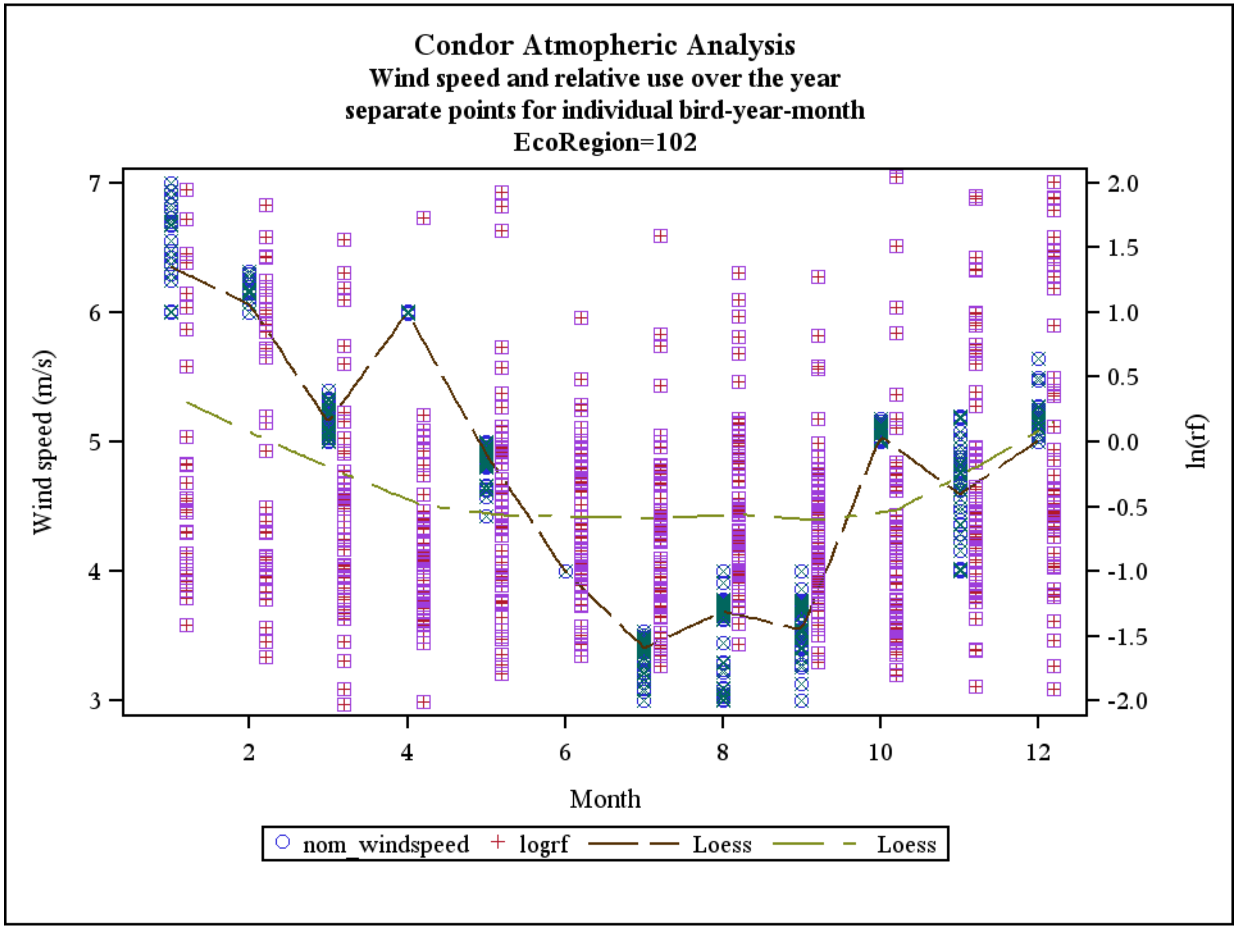

EcoRegion=116

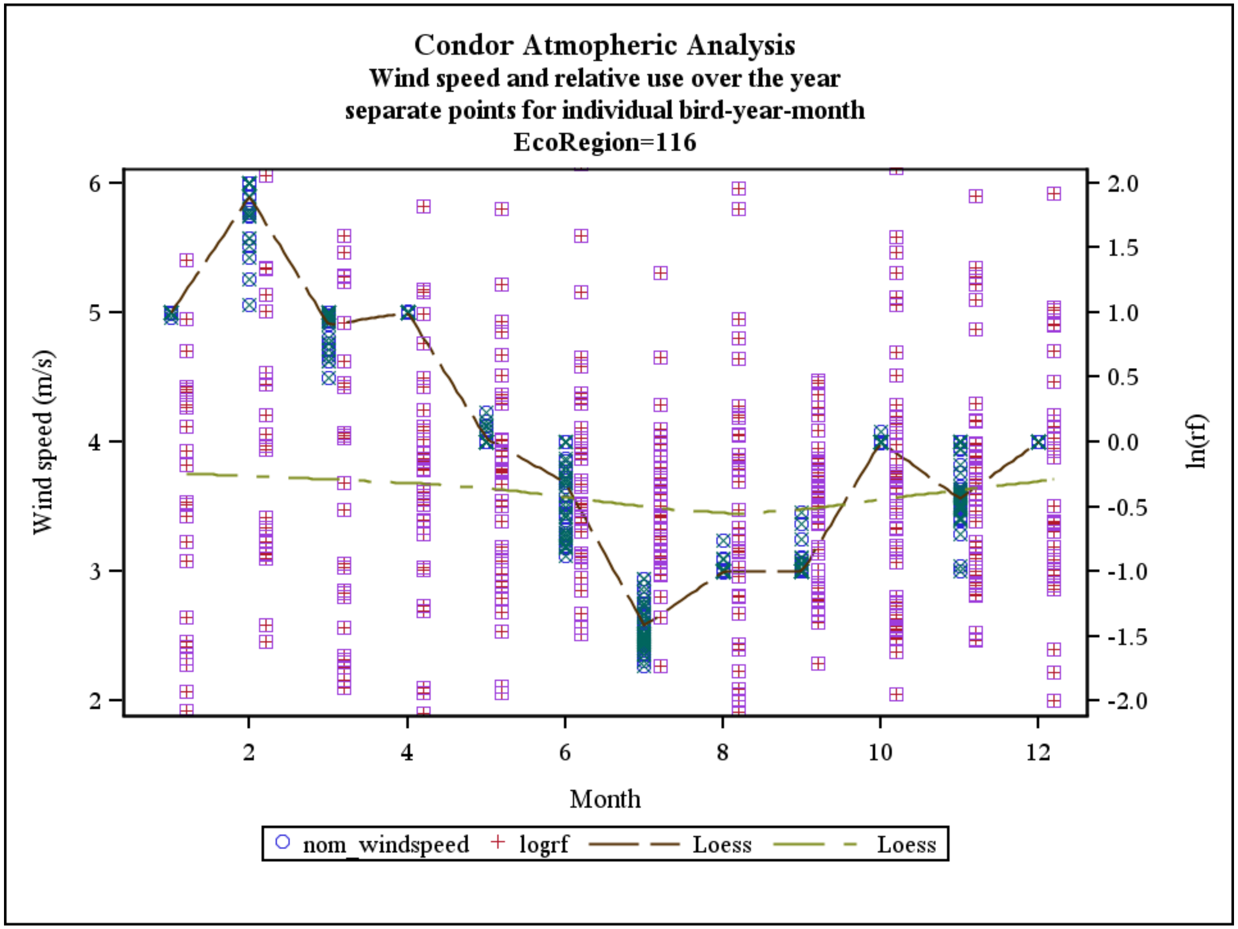

EcoRegion=117

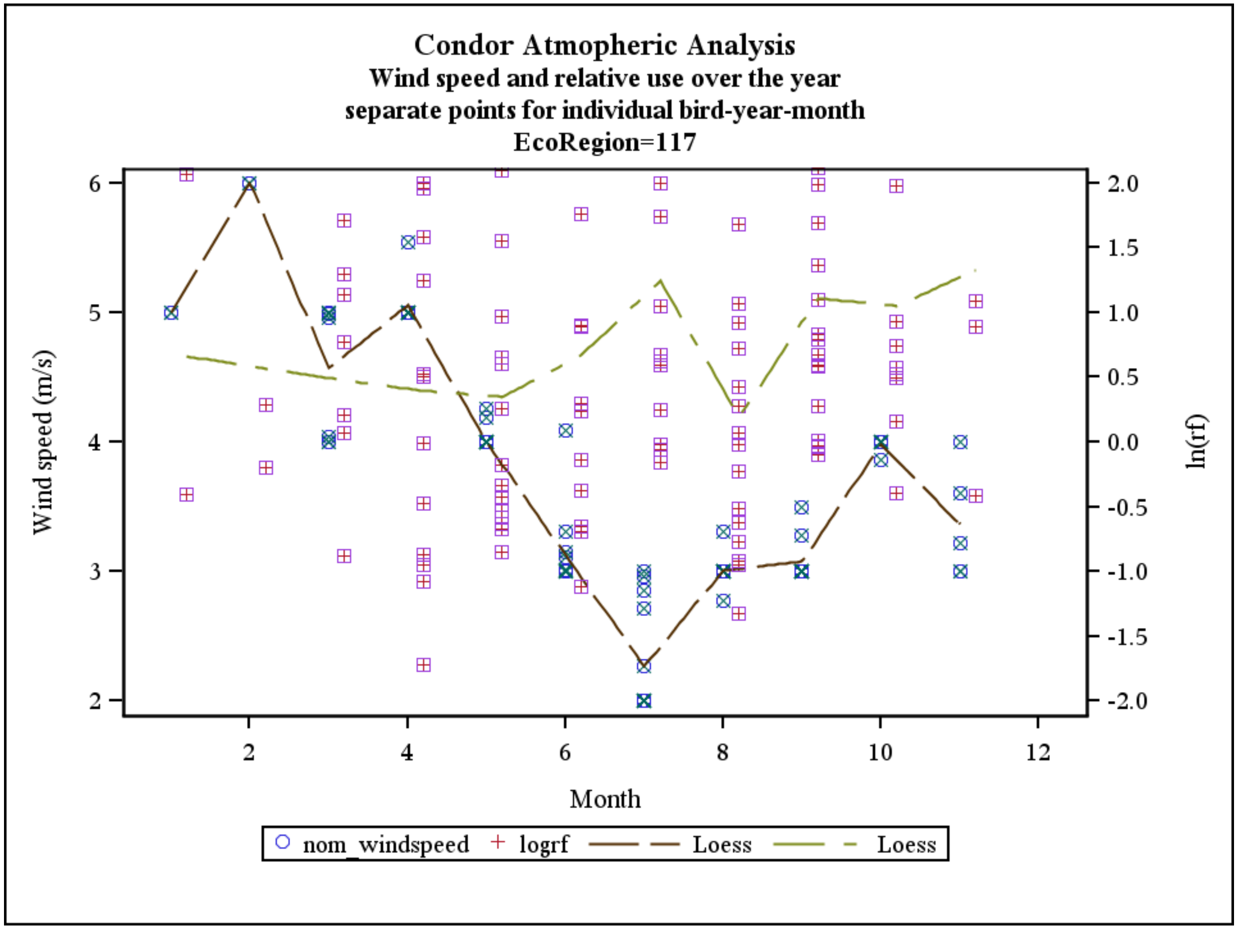

EcoRegion=118

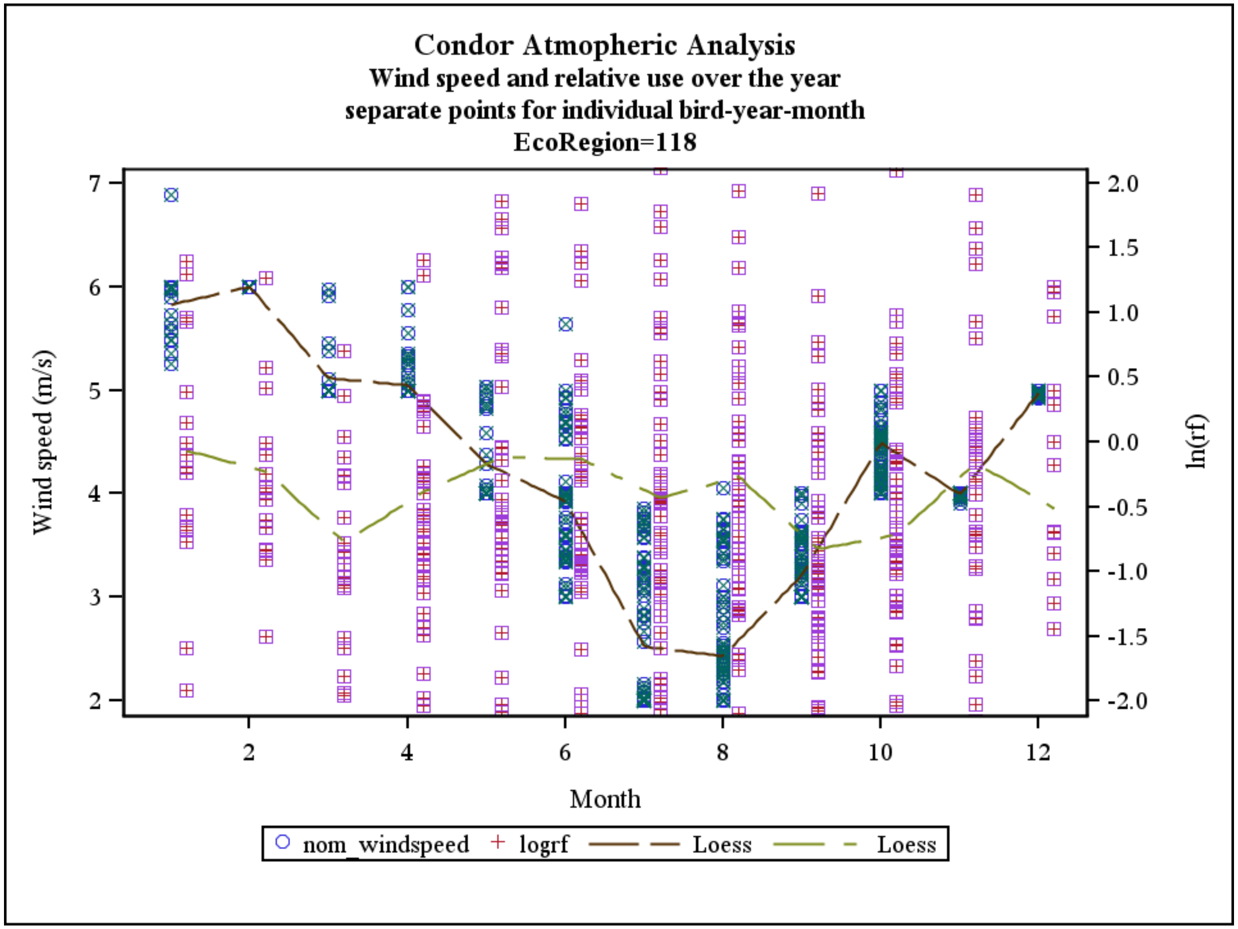

EcoRegion=119

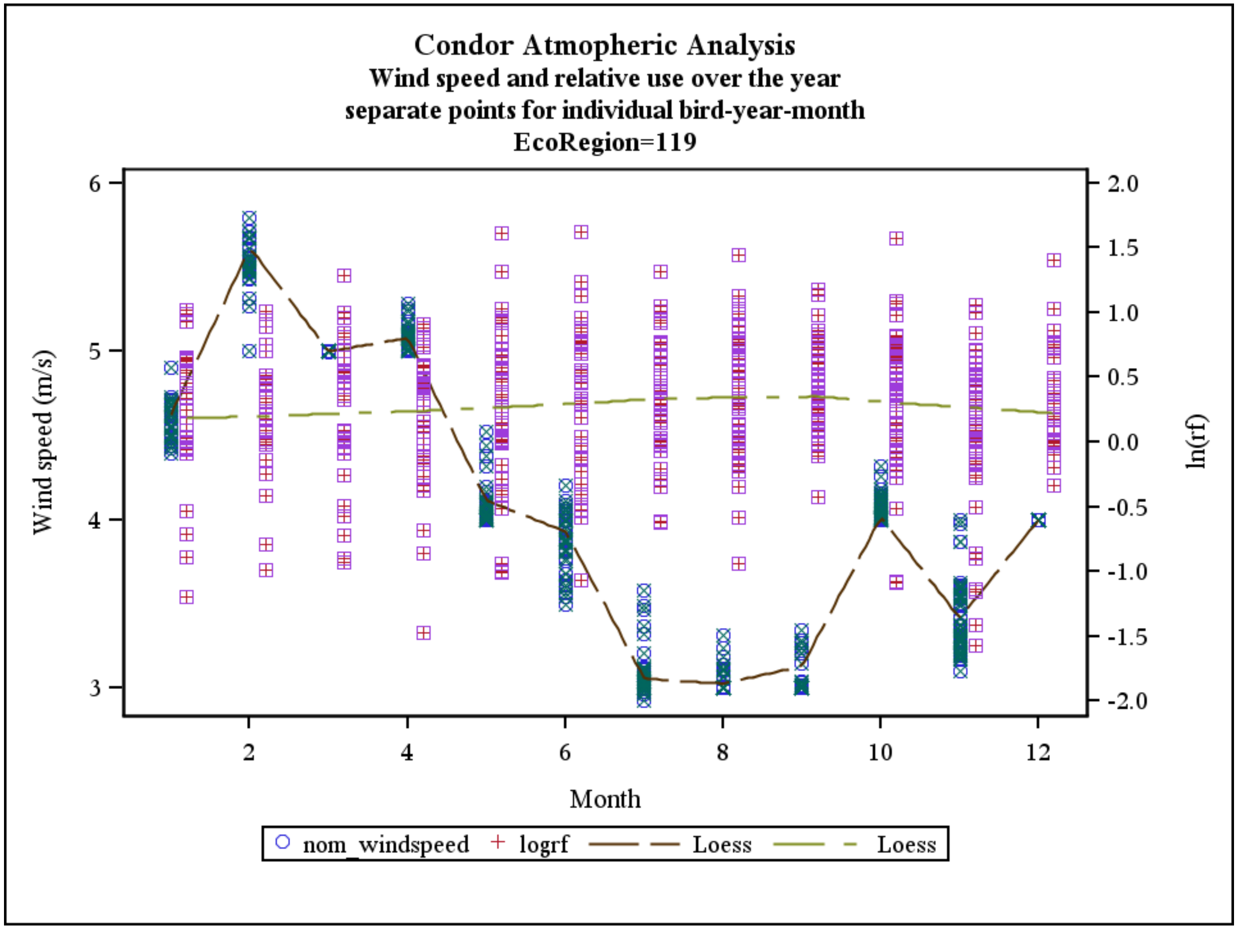

EcoRegion=123

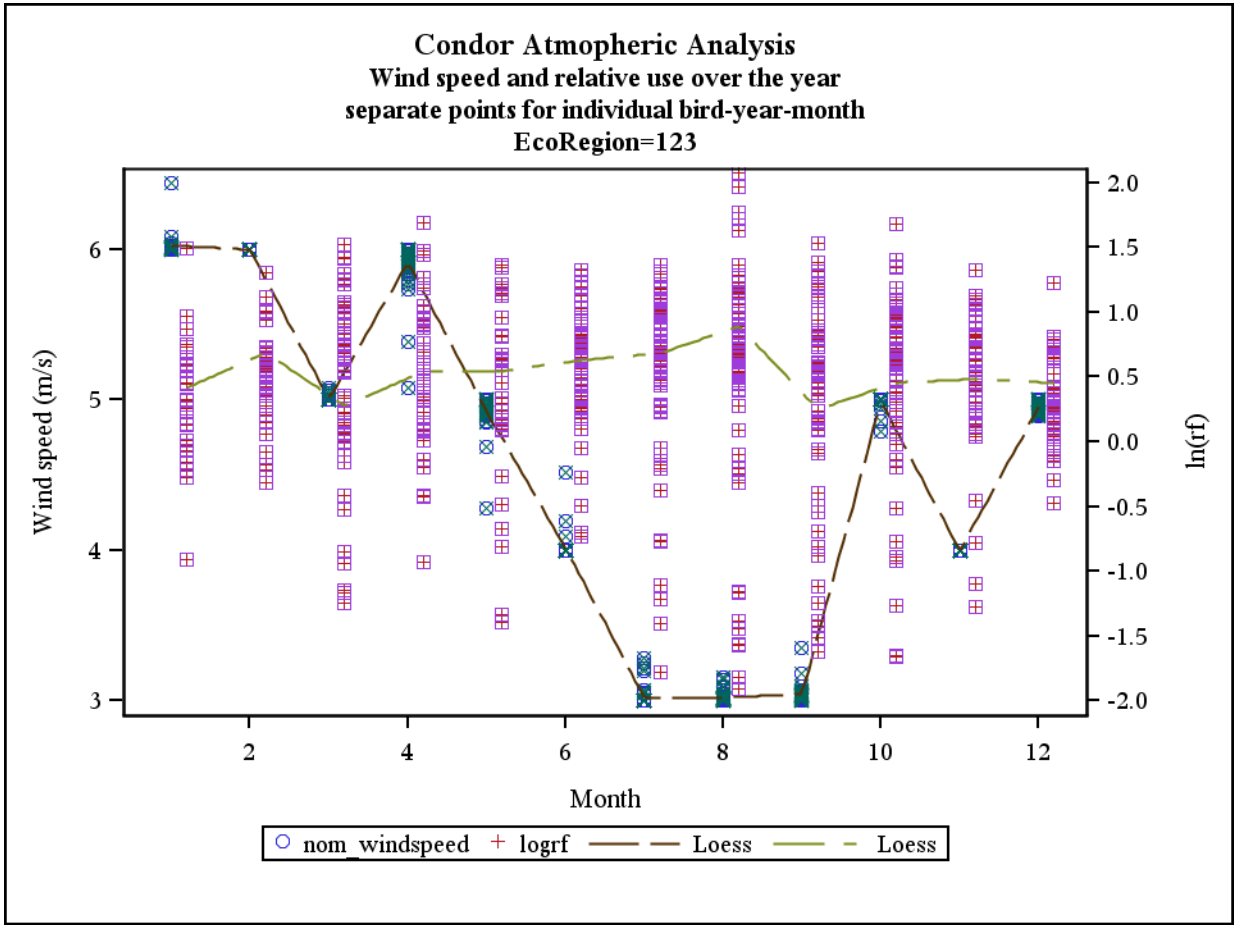

EcoRegion=124

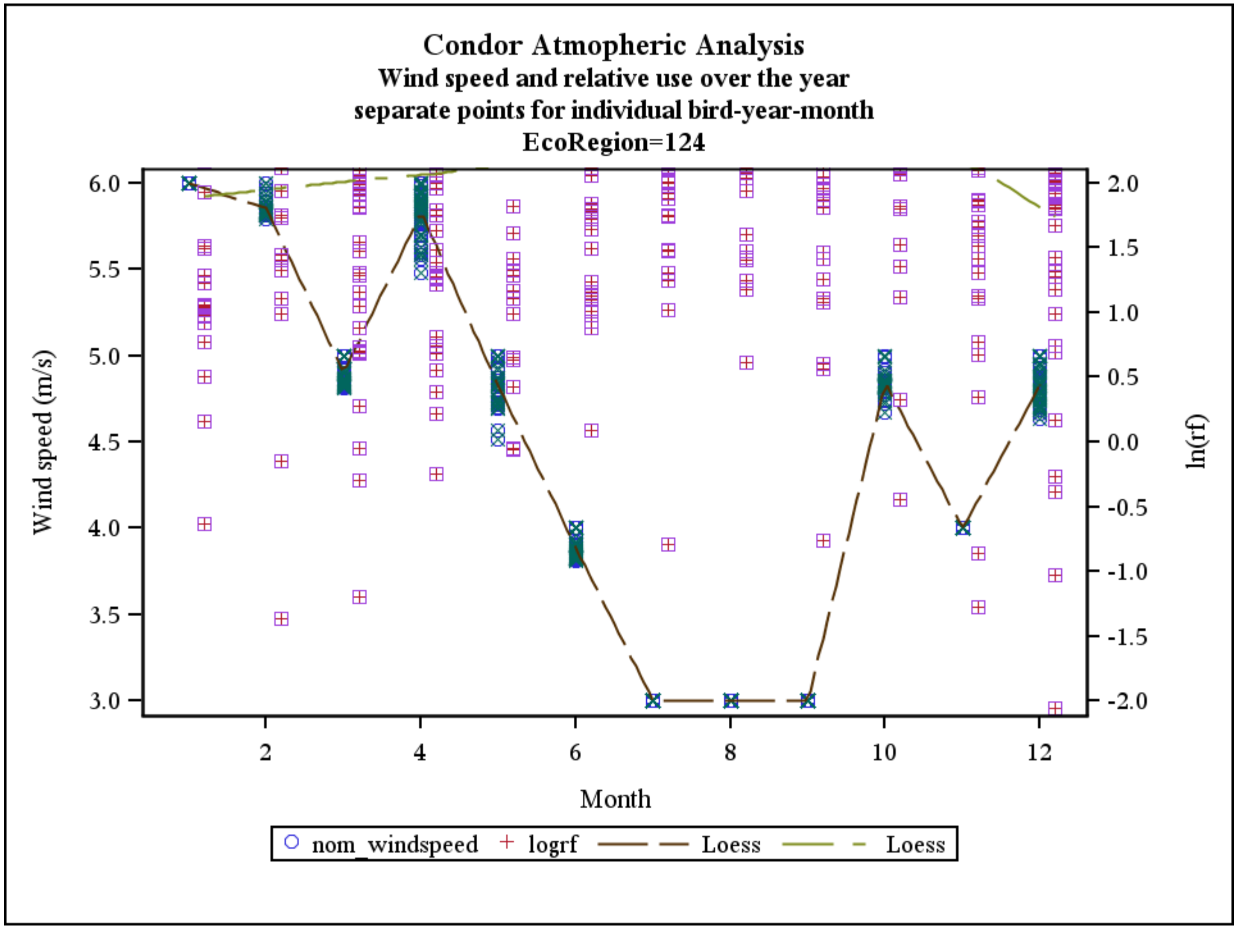

EcoRegion=125

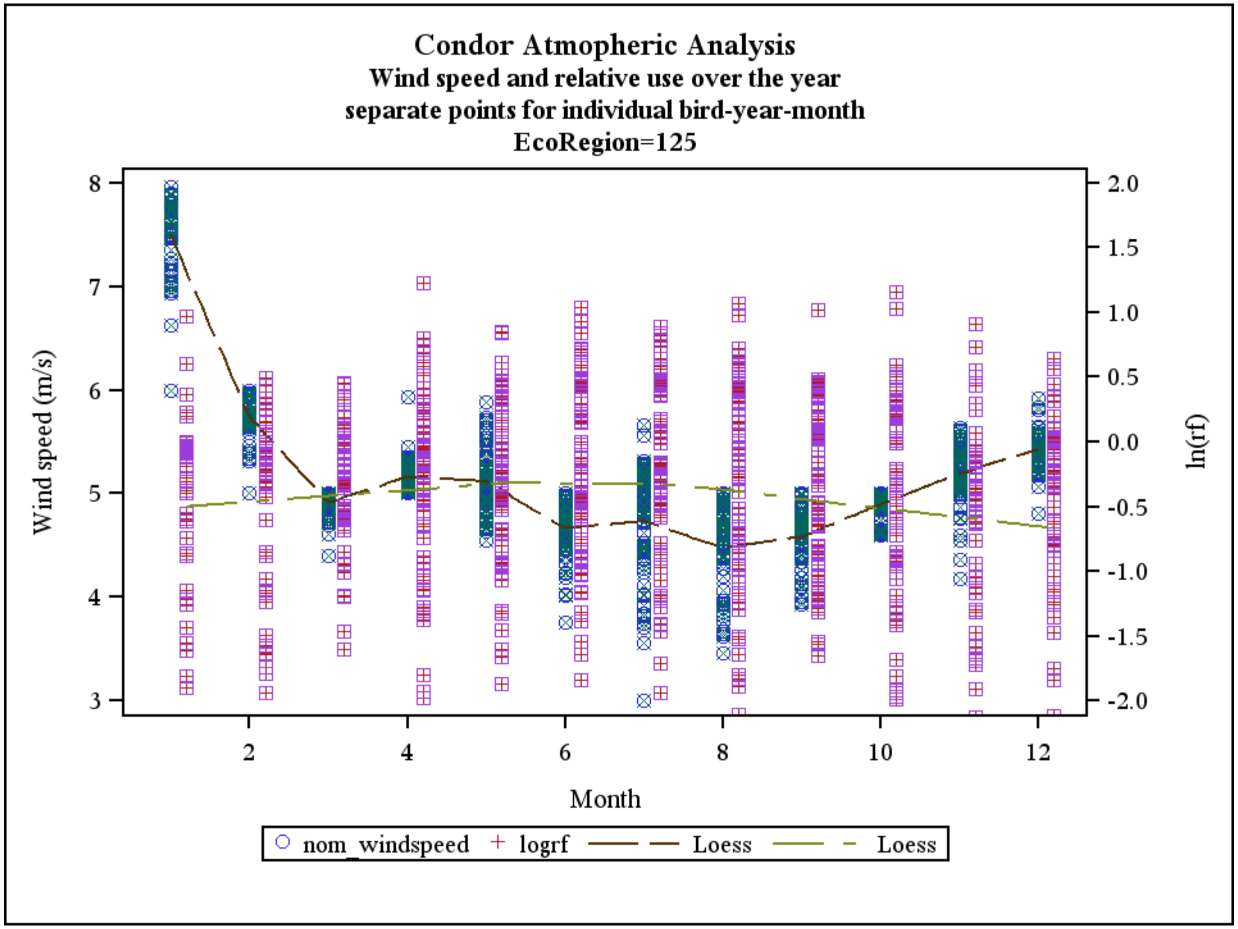

EcoRegion=126

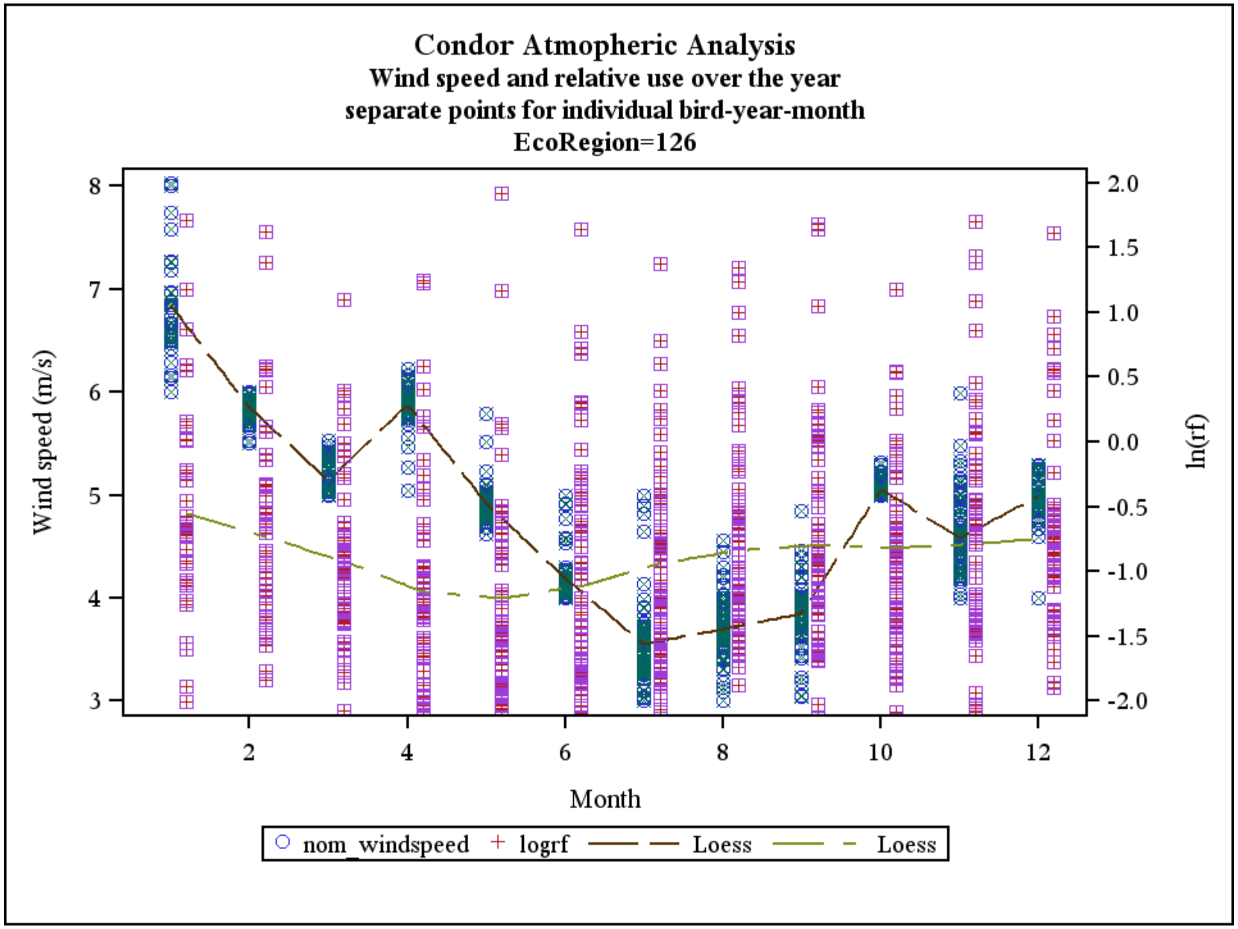

EcoRegion=127

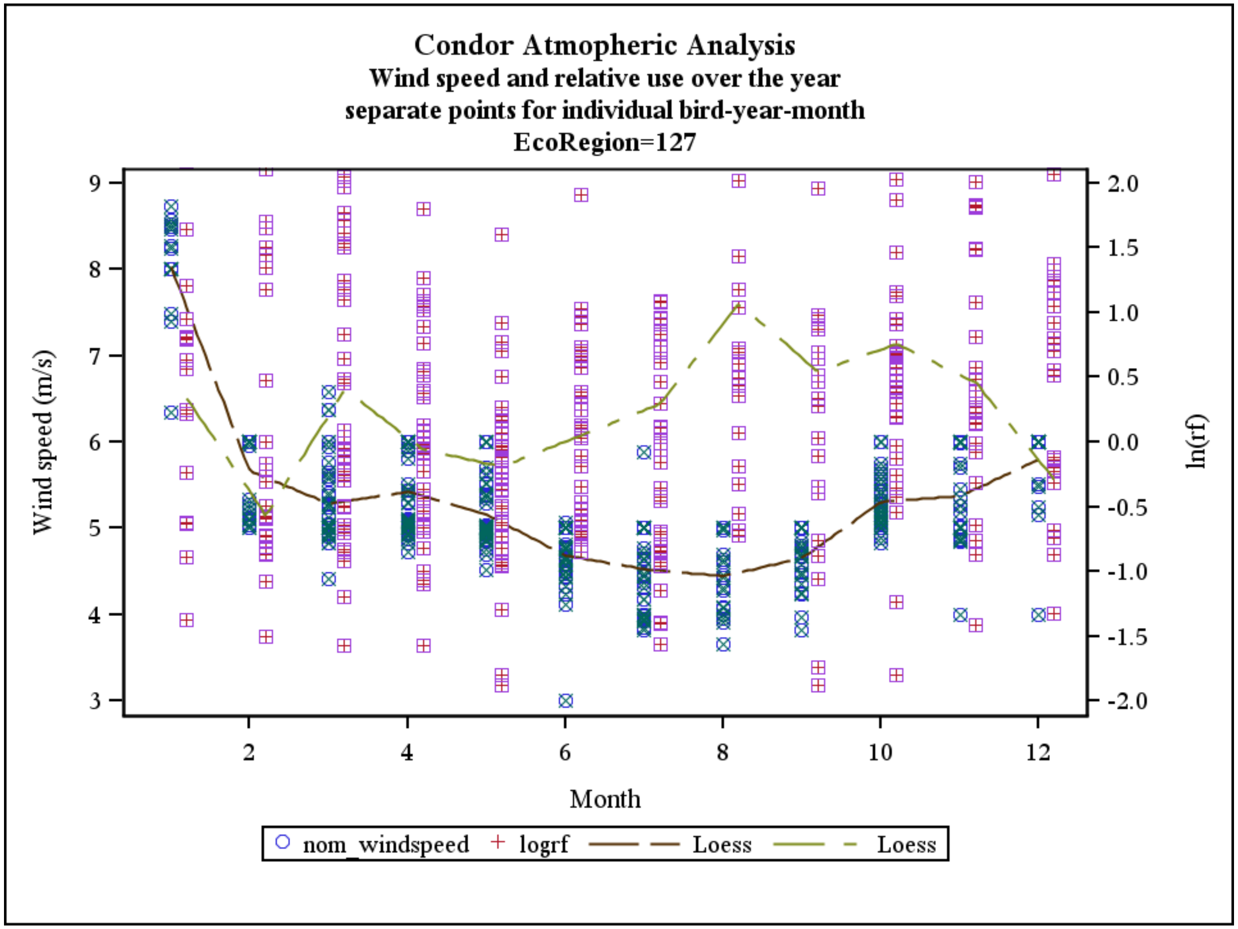

EcoRegion=128

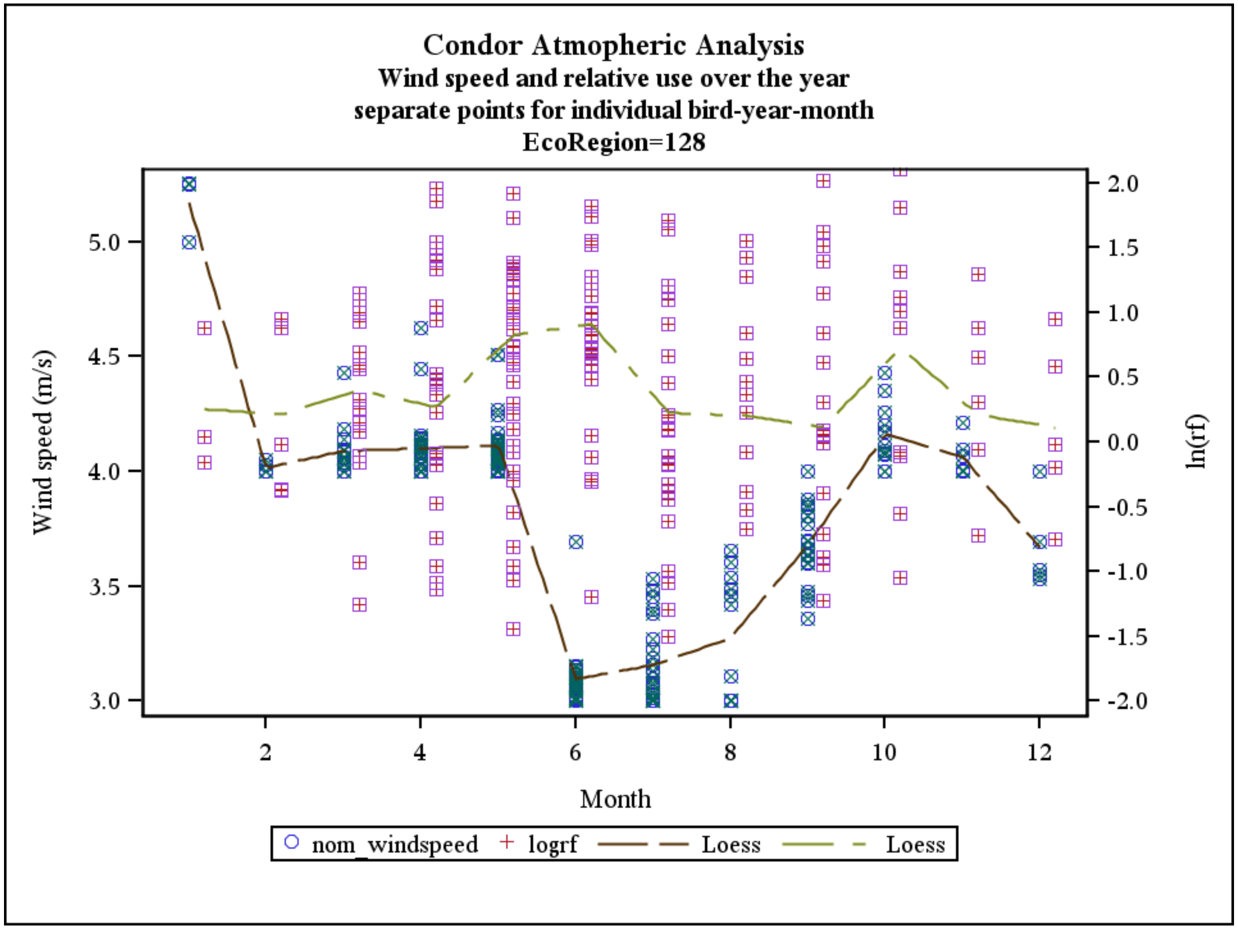

EcoRegion=147

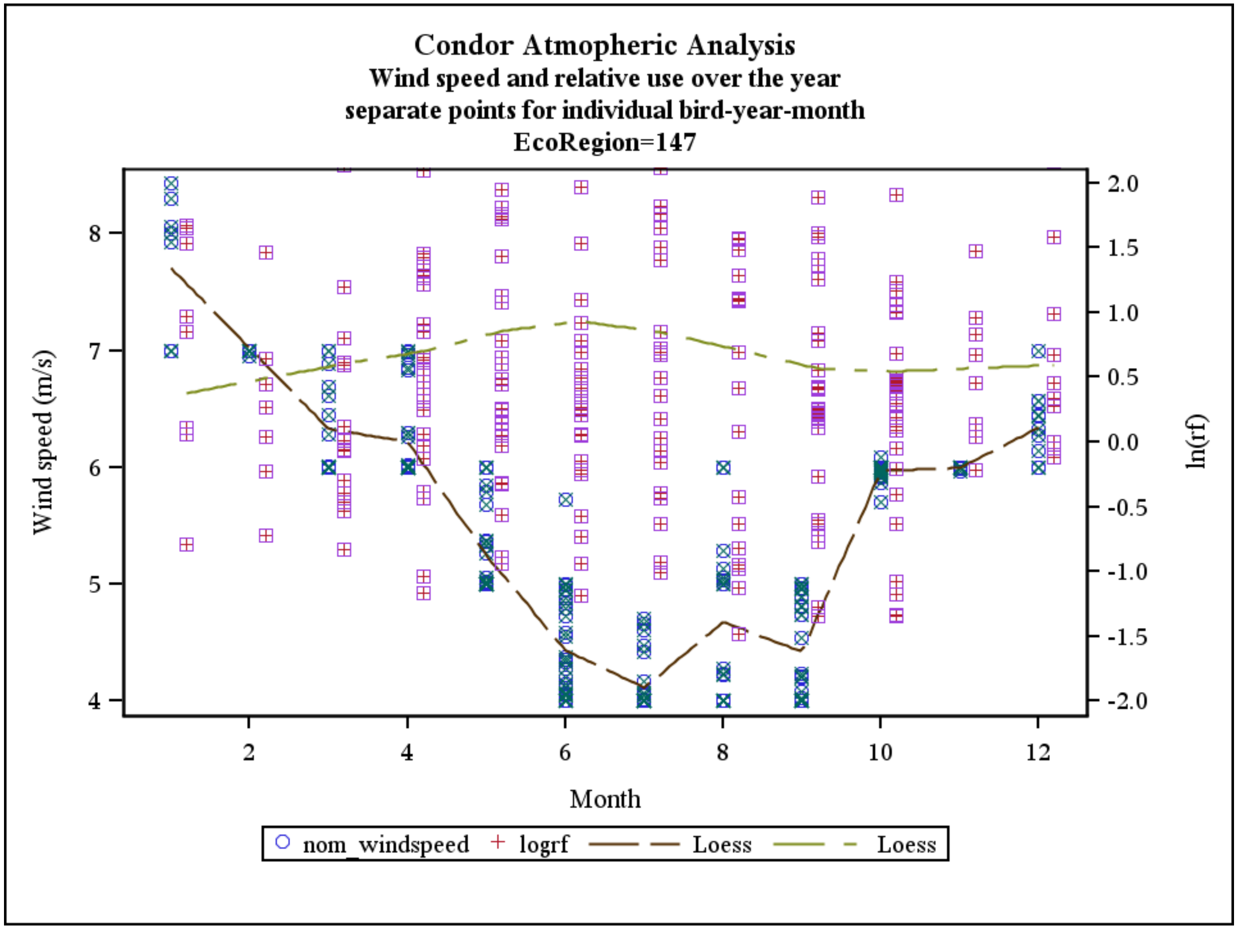

EcoRegion=192

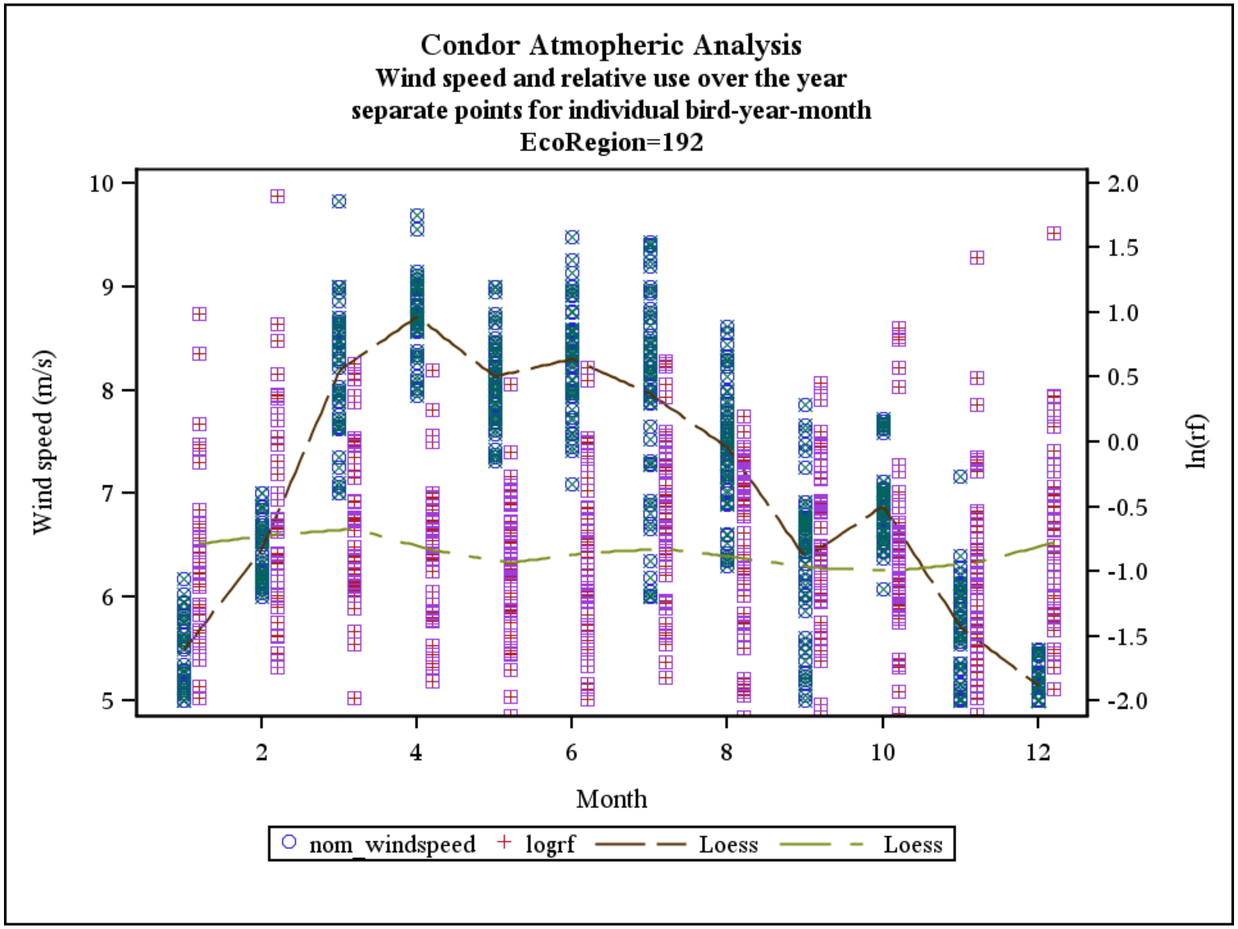

EcoRegion=193

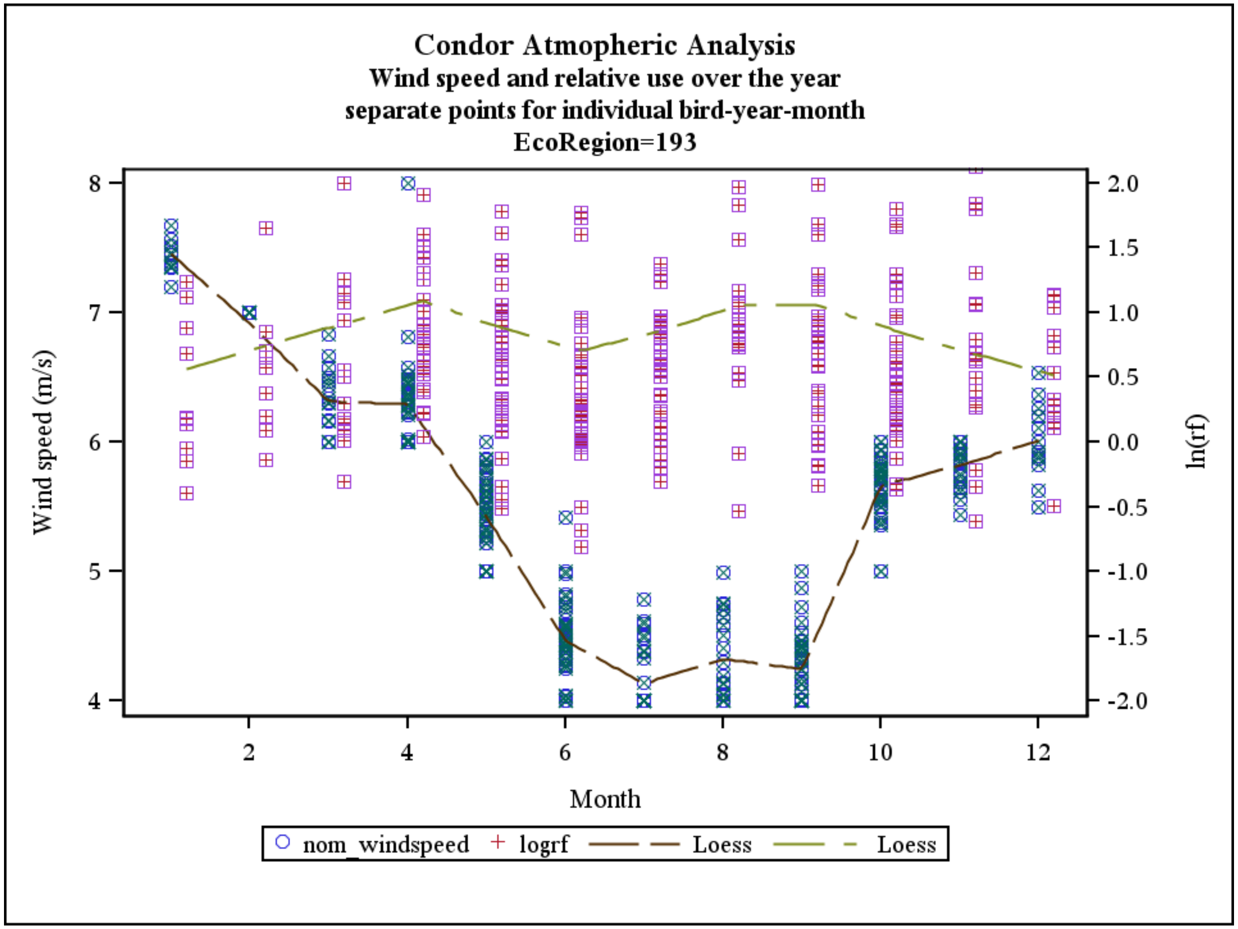

Supplement: Document S6 — Plots for three meteorological parameters and raw ln (rf) values plotted against months in the annual cycle for each of the 25 California ecoregions examined in the study. (PDF) [file pone.0088430.s006.pdf]
