# Supplementary material for: Resource Selection by the California Condor (Gymnogyps californianus) Relative to Terrestrial-Based Habitats and Meteorological Conditions
Source: PLoS One. 2014 Feb 11;9(2):e88430. doi: 10.1371/journal.pone.0088430 (PMC3921182; doi:10.1371/journal.pone.0088430)

Document S7. The following figures contain plots for each of three meteorological parameters (i.e., thermal height, thermal velocity, and wind speed; left axis) and mean ln(rf) values (right axis) plotted against months in the annual cycle for each of the 25 California ecoregions examined. Monthly estimates were adjusted for individual bird attributes, and the gray band is the 95% confidence interval for the mean ln(rf) values.

EcoRegion=8

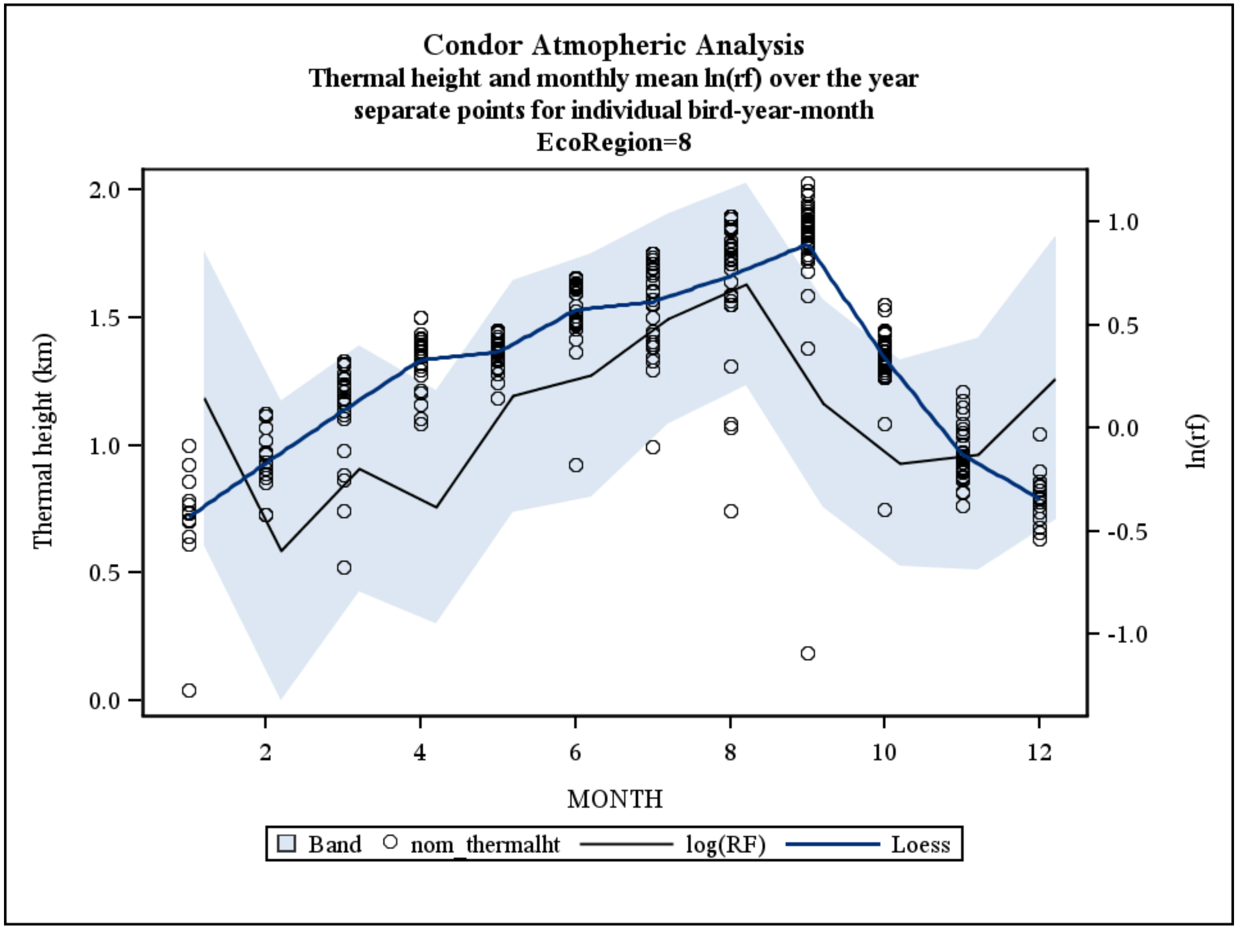
Condor Atmopheric Analysis
Thermal height and monthly mean ln(rf) over the year
separate points for individual bird-year-month
EcoRegion=8
Thermal height (km)
0.0
0.5
1.0
1.5
2.0
ln(rf)
1.0
0.5
0.0
-0.5
-1.0
2
4
6
8
10
12
MONTH
Band
nom_thermalht
log(RF)
Loess

EcoRegion=9

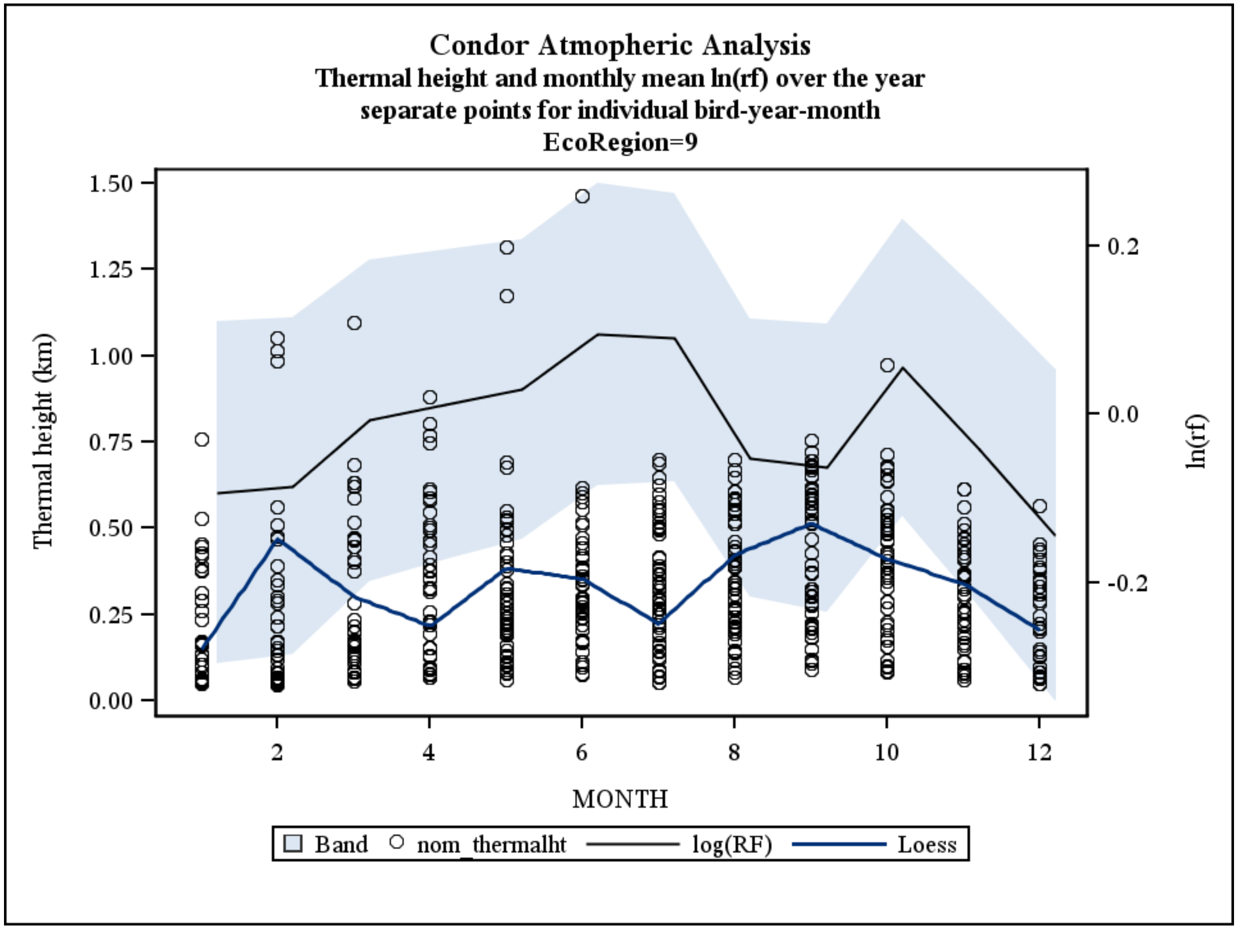
Condor Atmopheric Analysis
Thermal height and monthly mean ln(rf) over the year
separate points for individual bird-year-month
EcoRegion=9
Thermal height (km)
1.50
1.25
1.00
0.75
0.50
0.25
0.00
ln(rf)
0.2
0.0
-0.2
2
4
6
8
10
12
MONTH
Band
nom_thermalht
log(RF)
Loess

EcoRegion=10

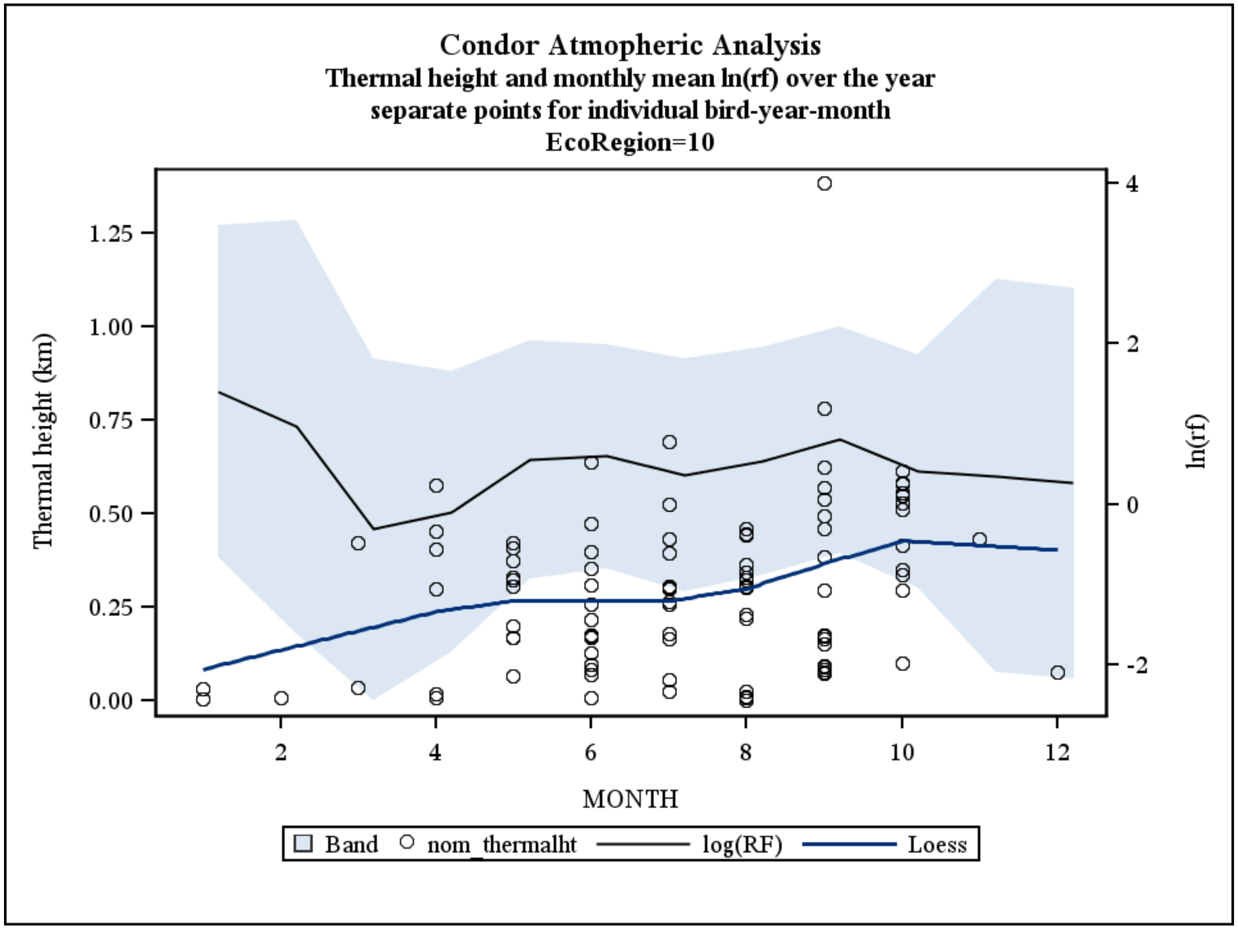

EcoRegion=13

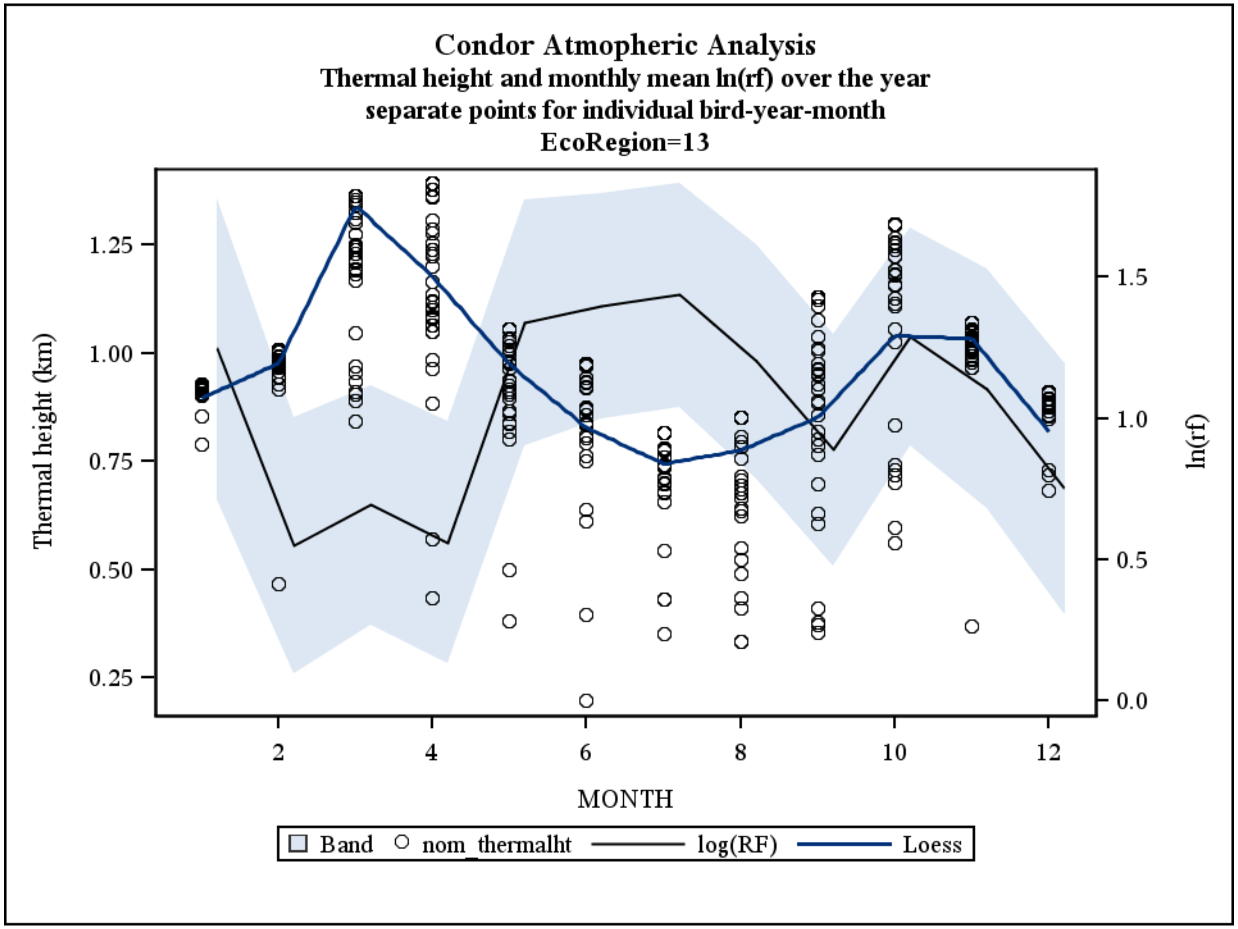

EcoRegion=15

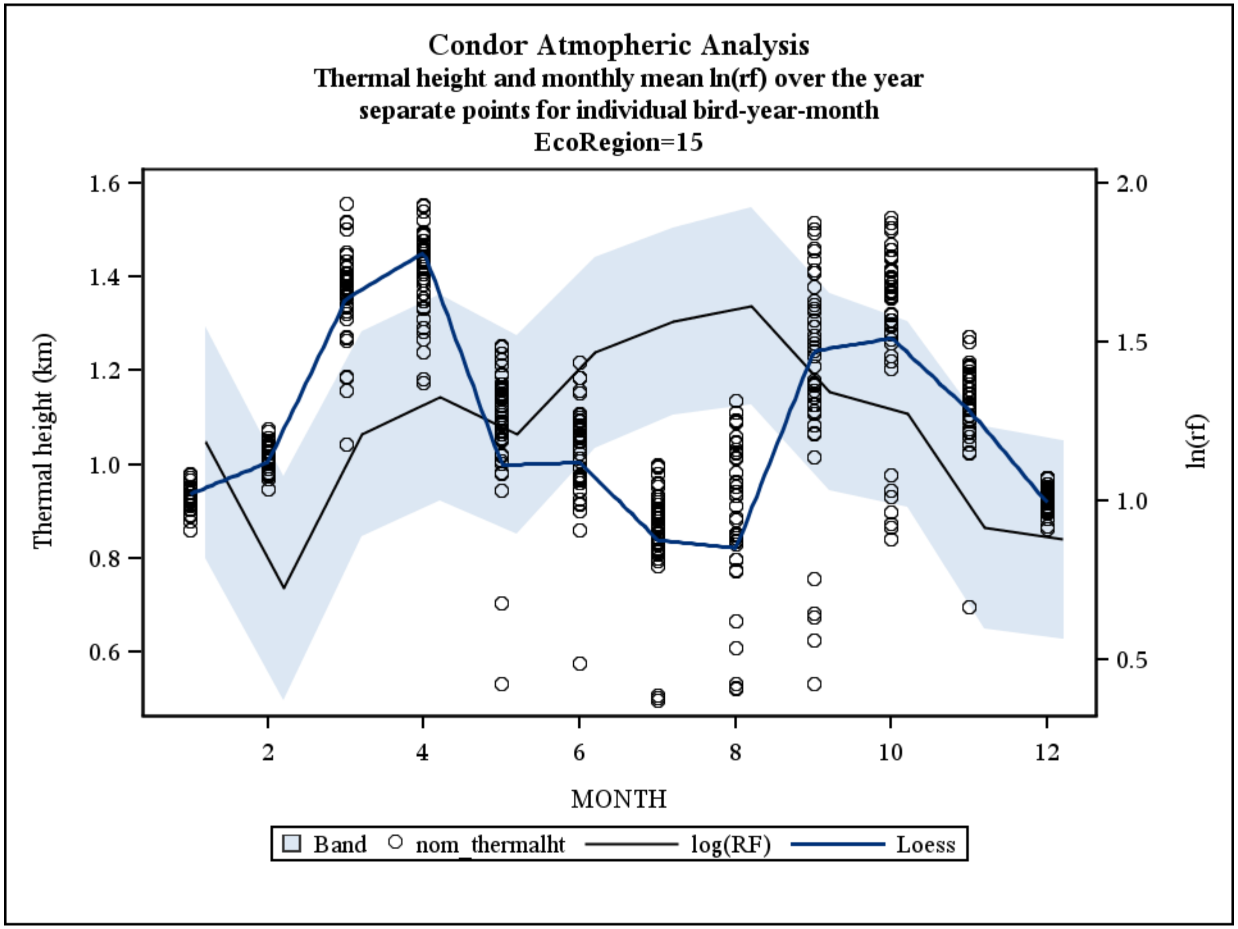

EcoRegion=16

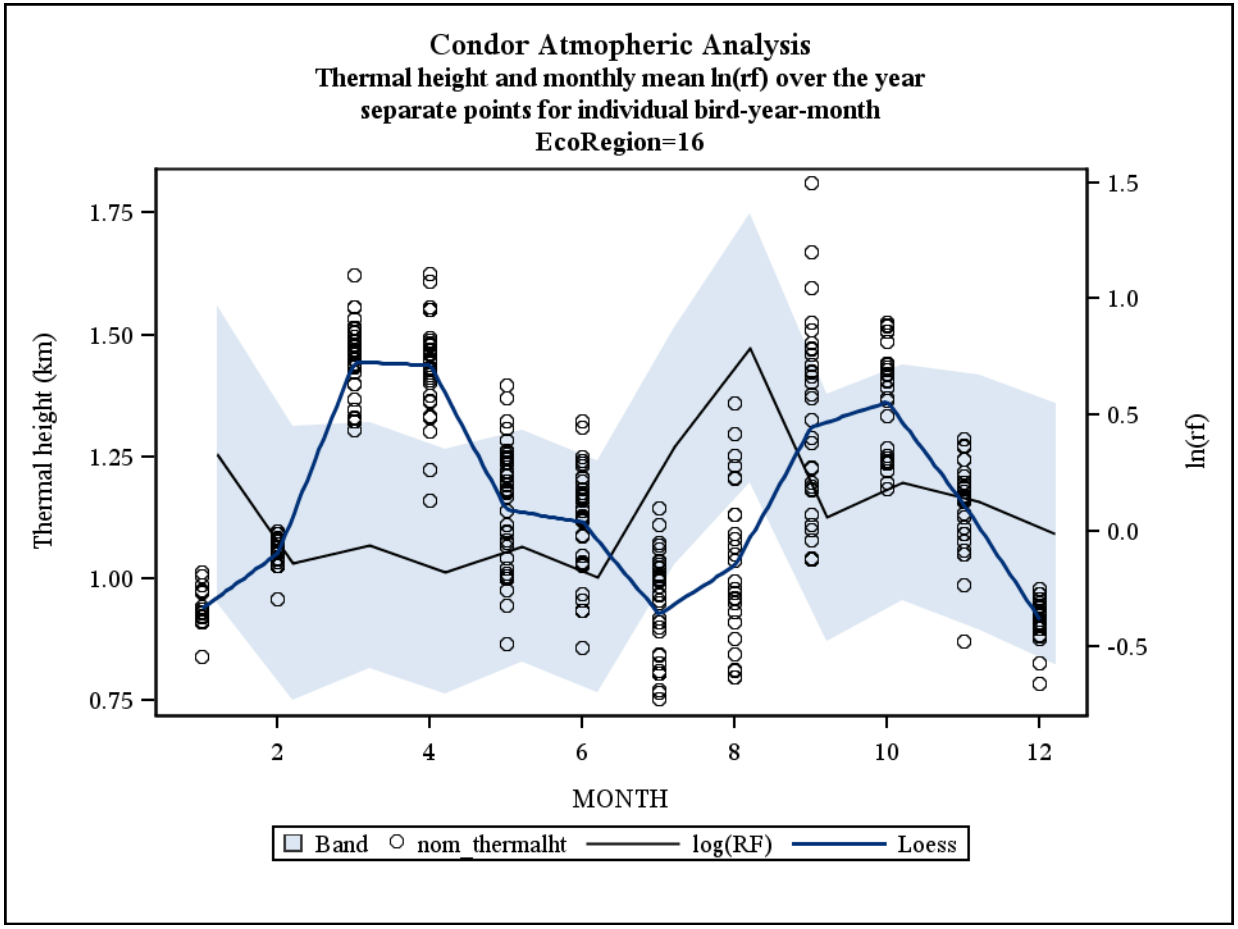

EcoRegion=18

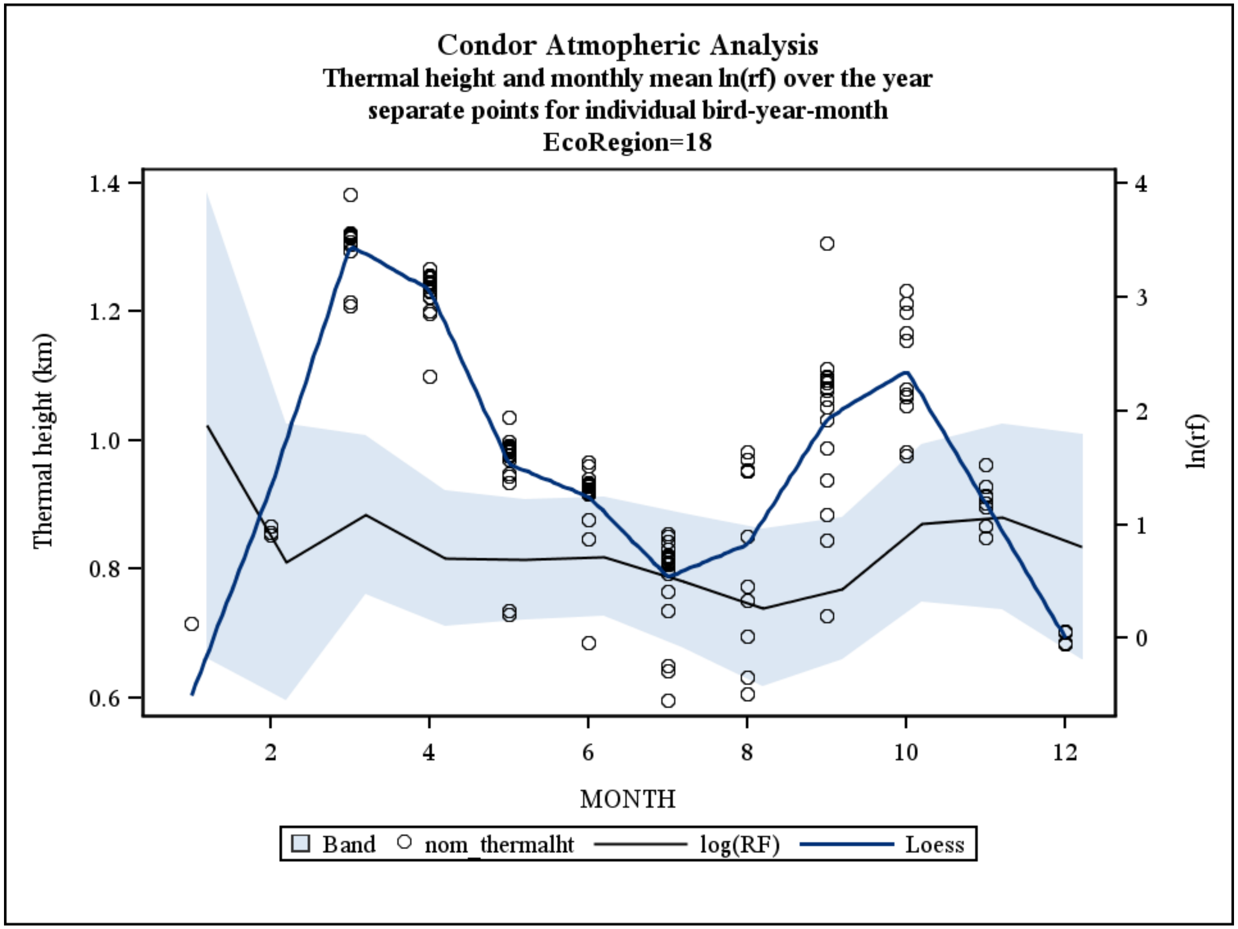

EcoRegion=39

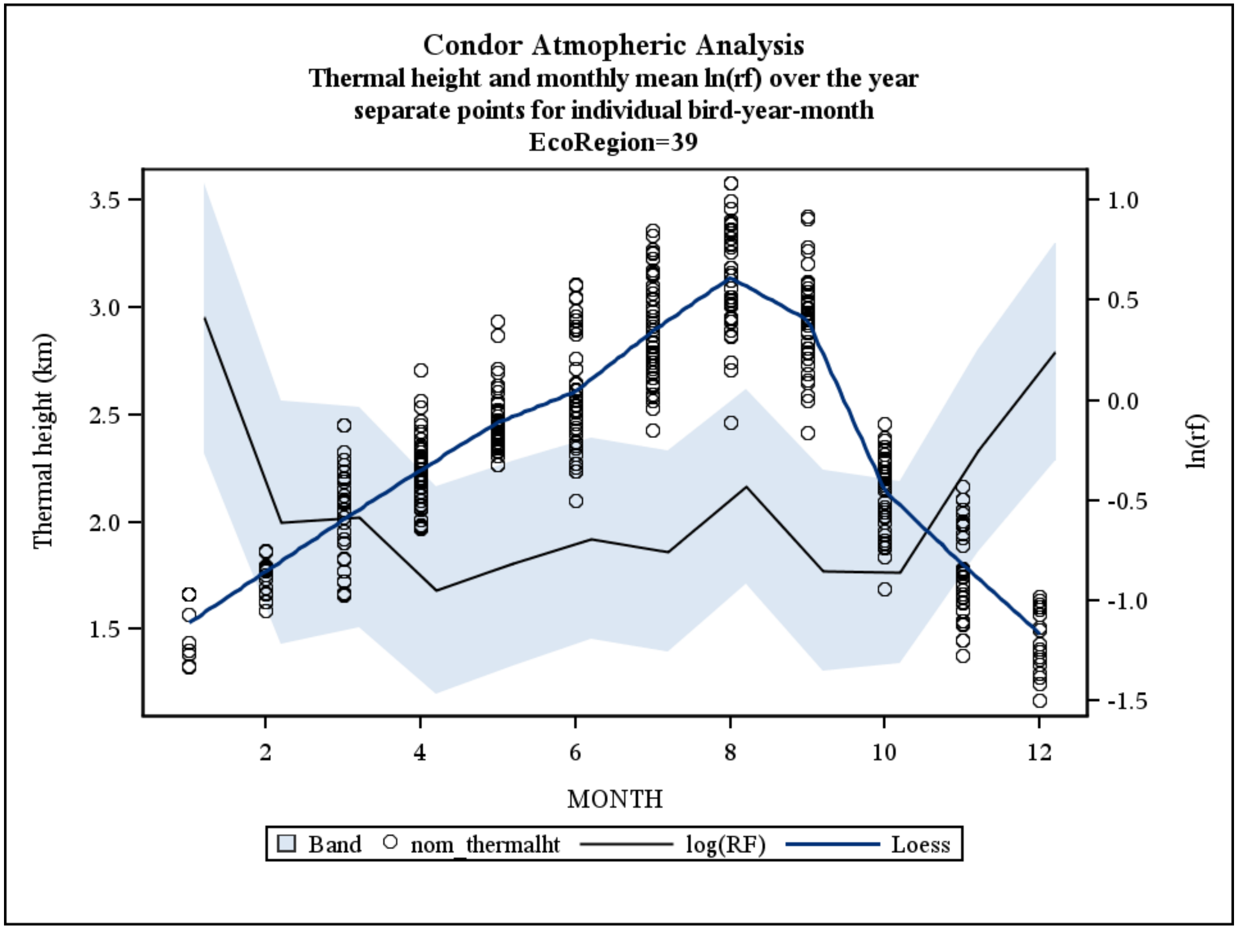

EcoRegion=40

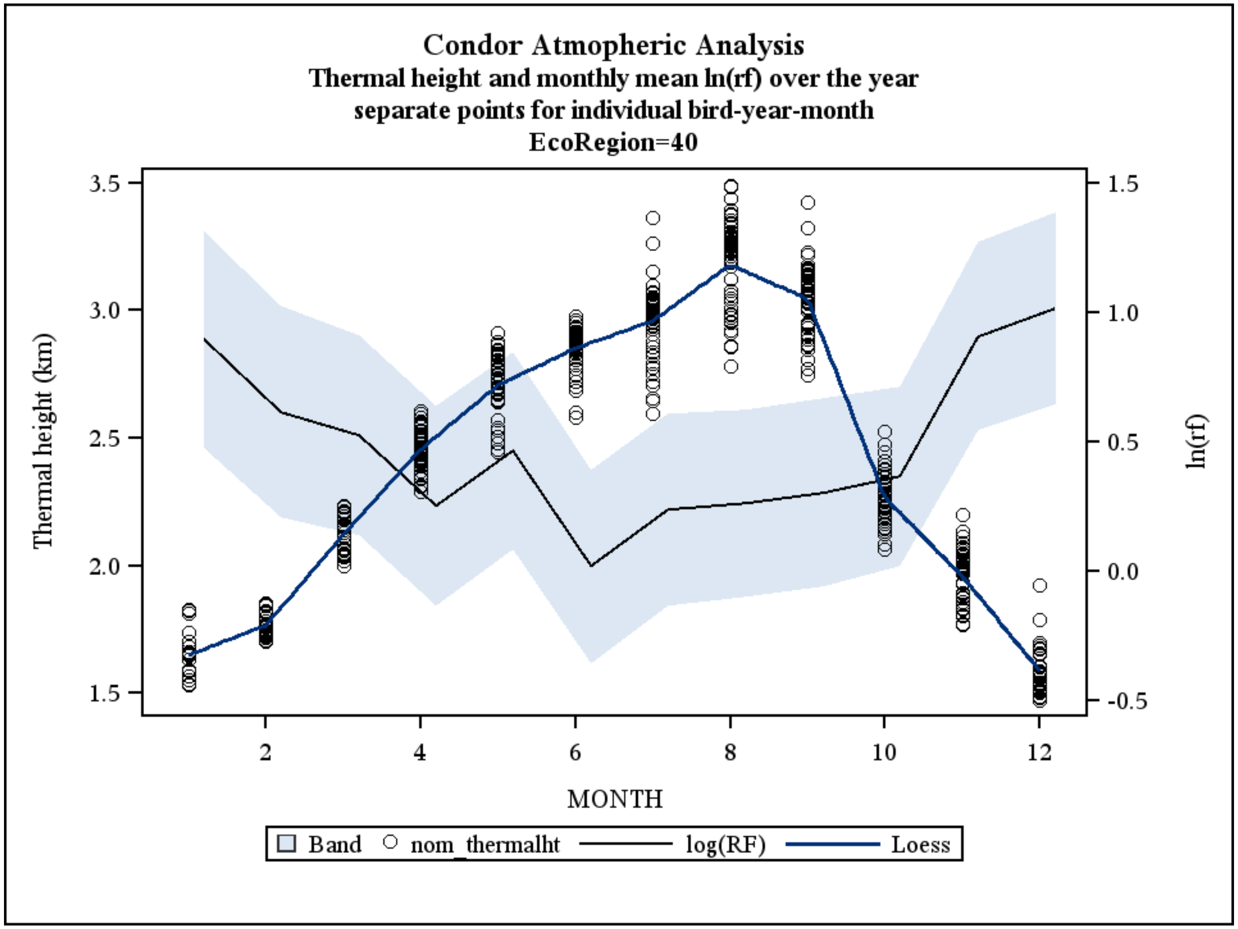

EcoRegion=95

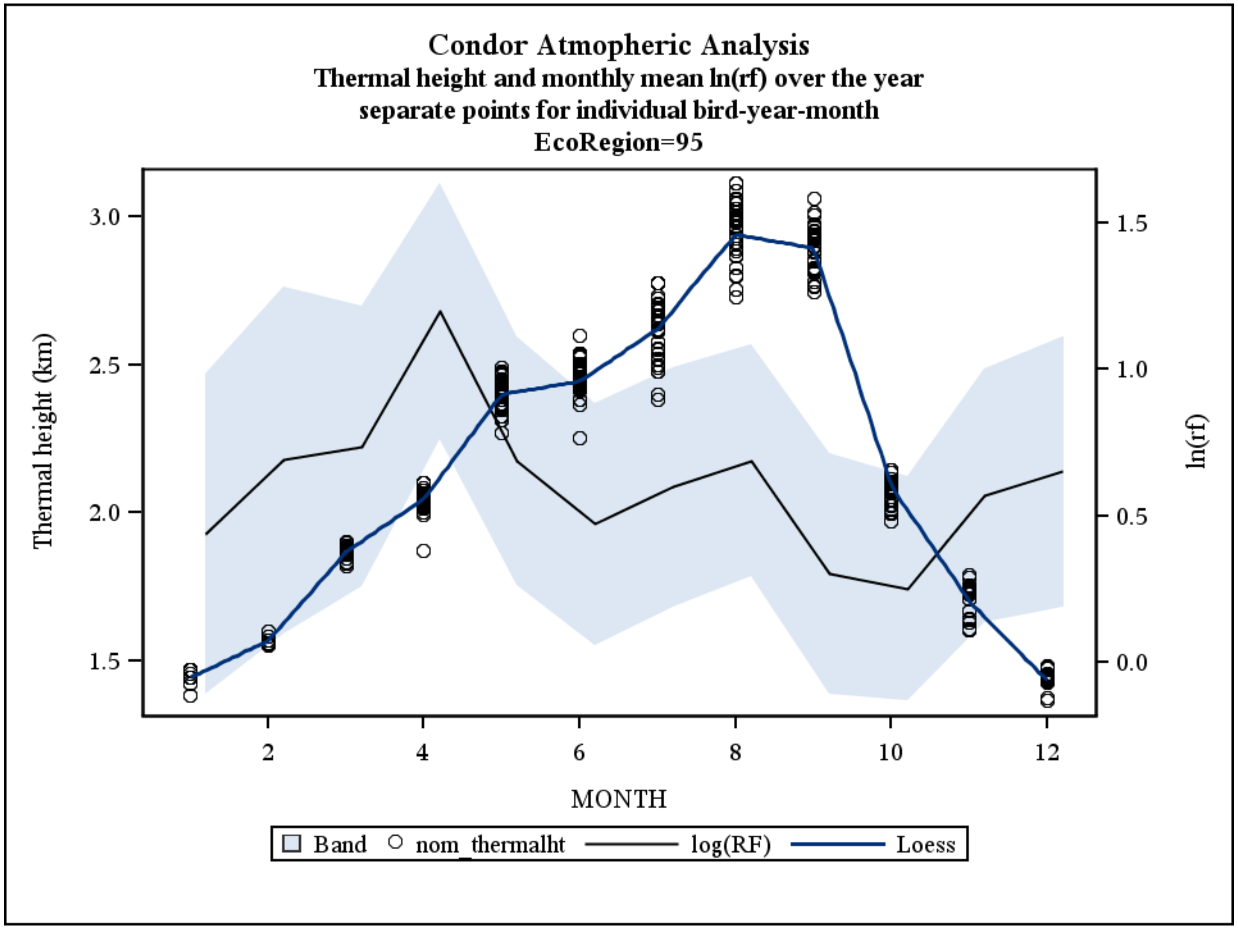

EcoRegion=101

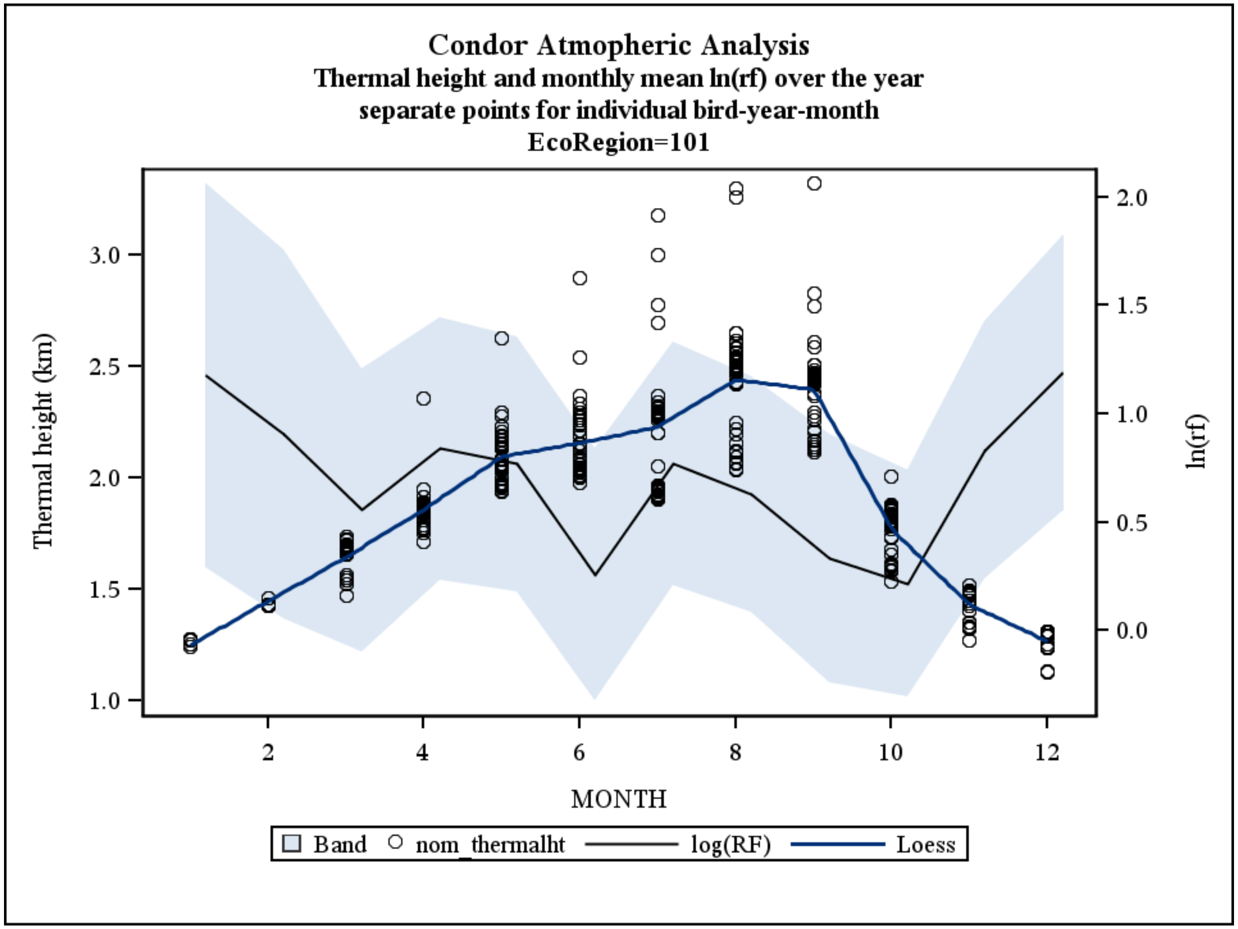

EcoRegion=102

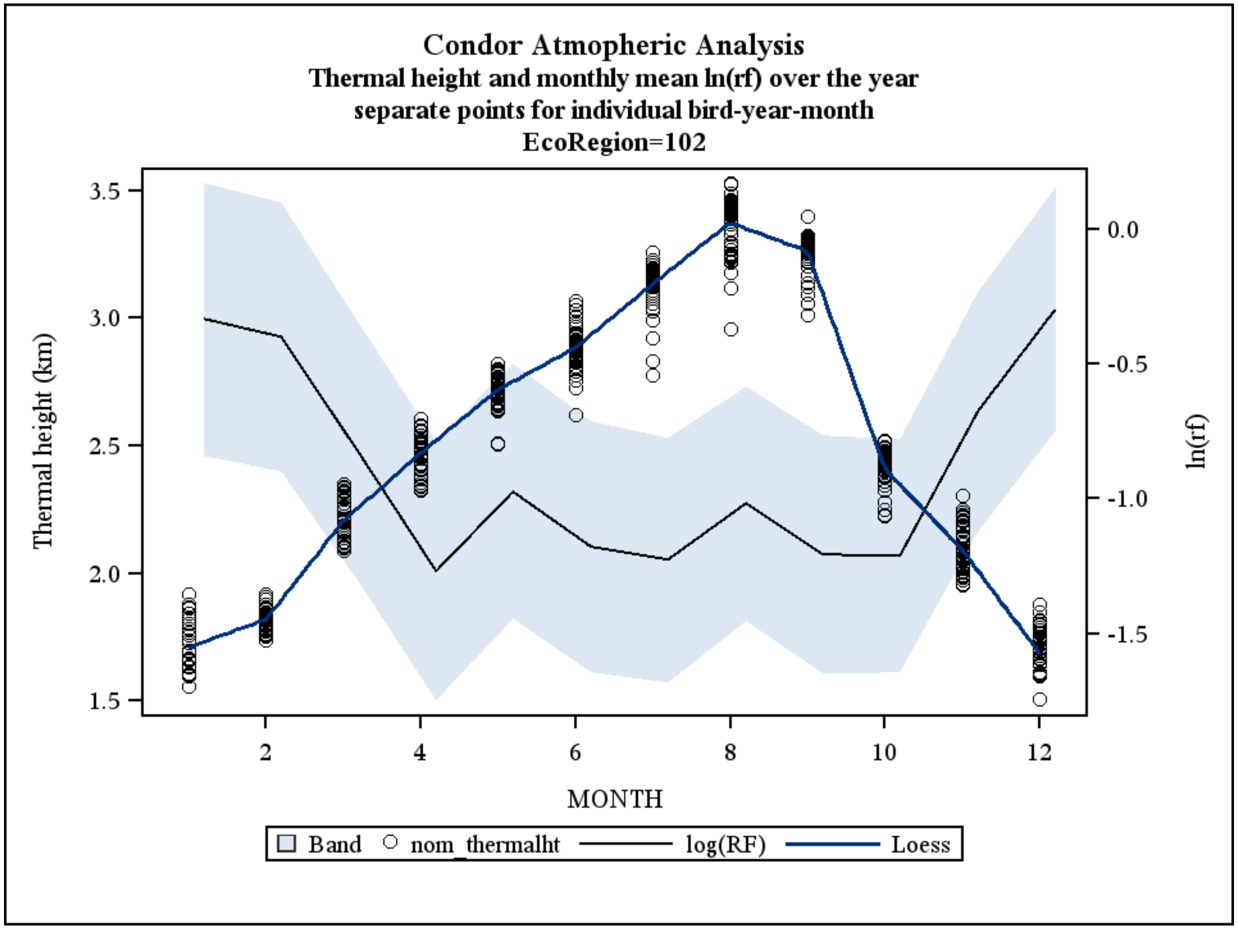

EcoRegion=116

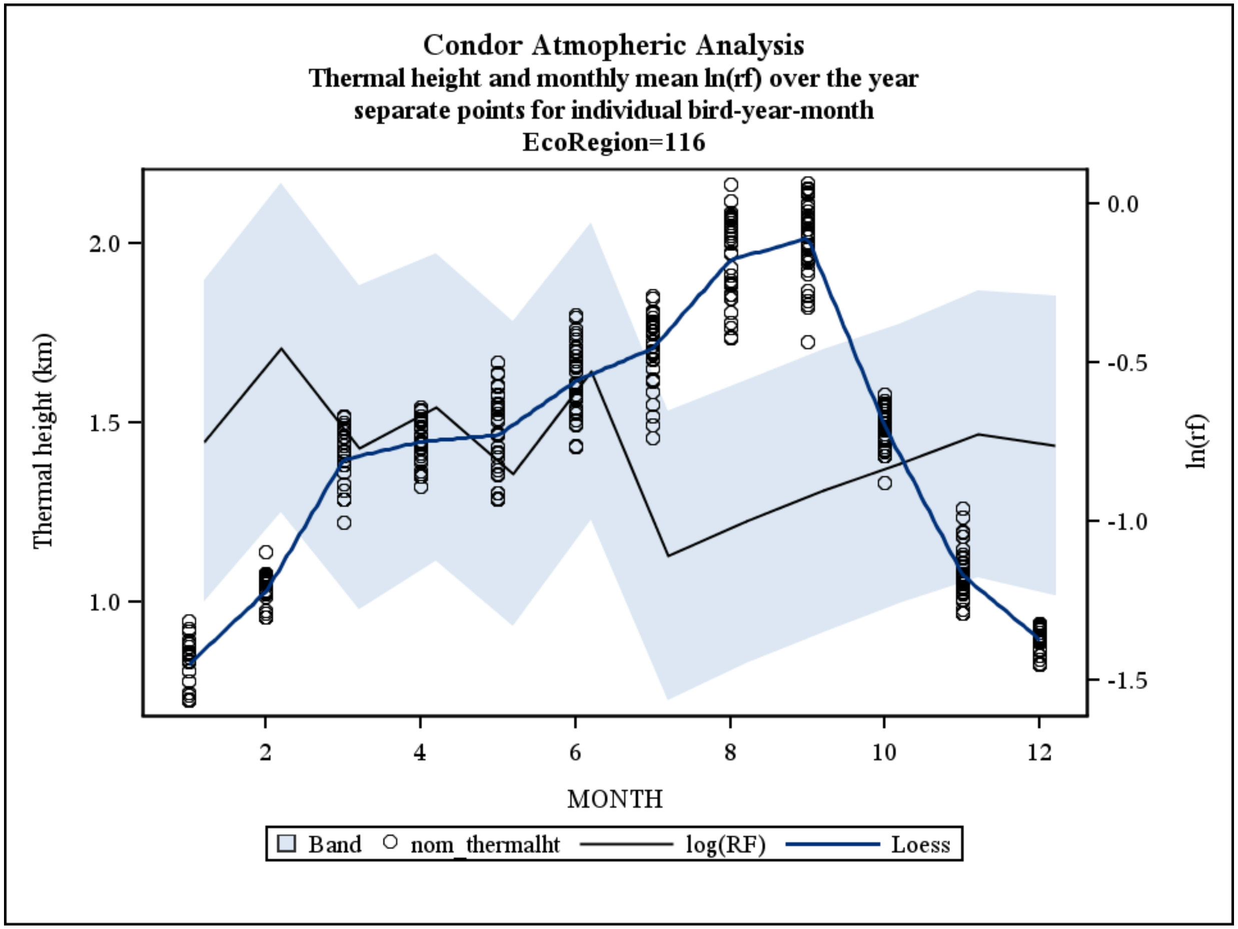

EcoRegion=117

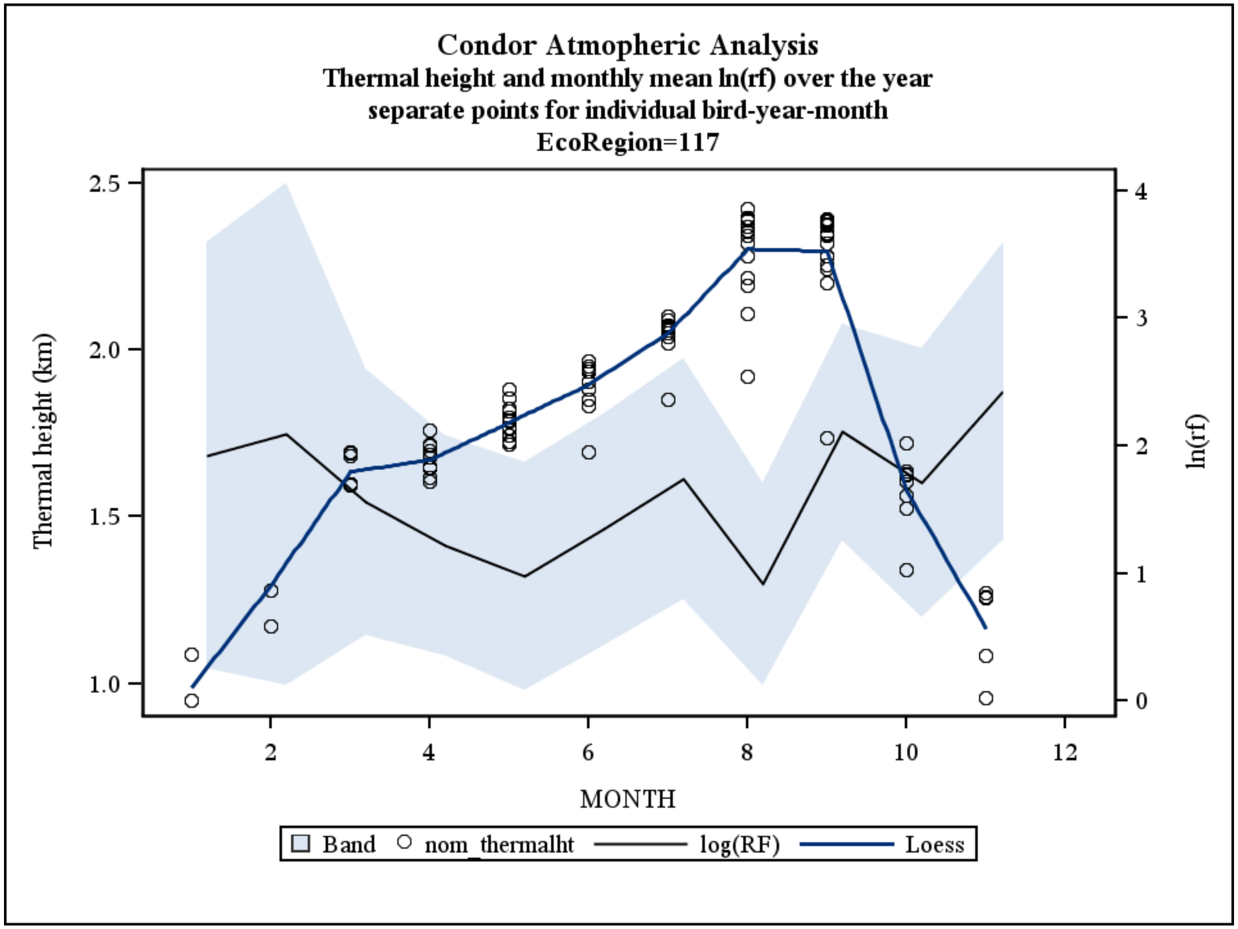

EcoRegion=118

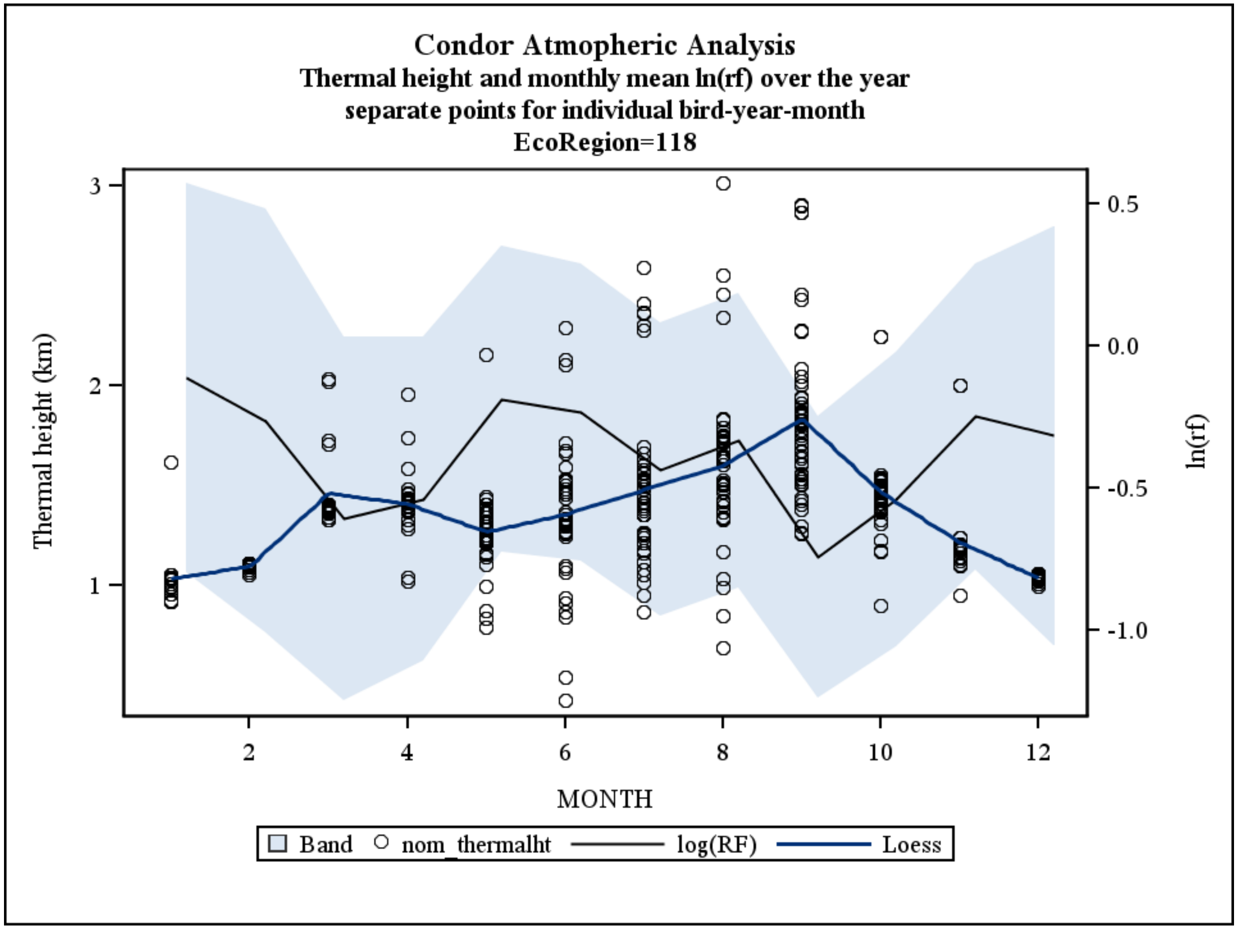

EcoRegion=119

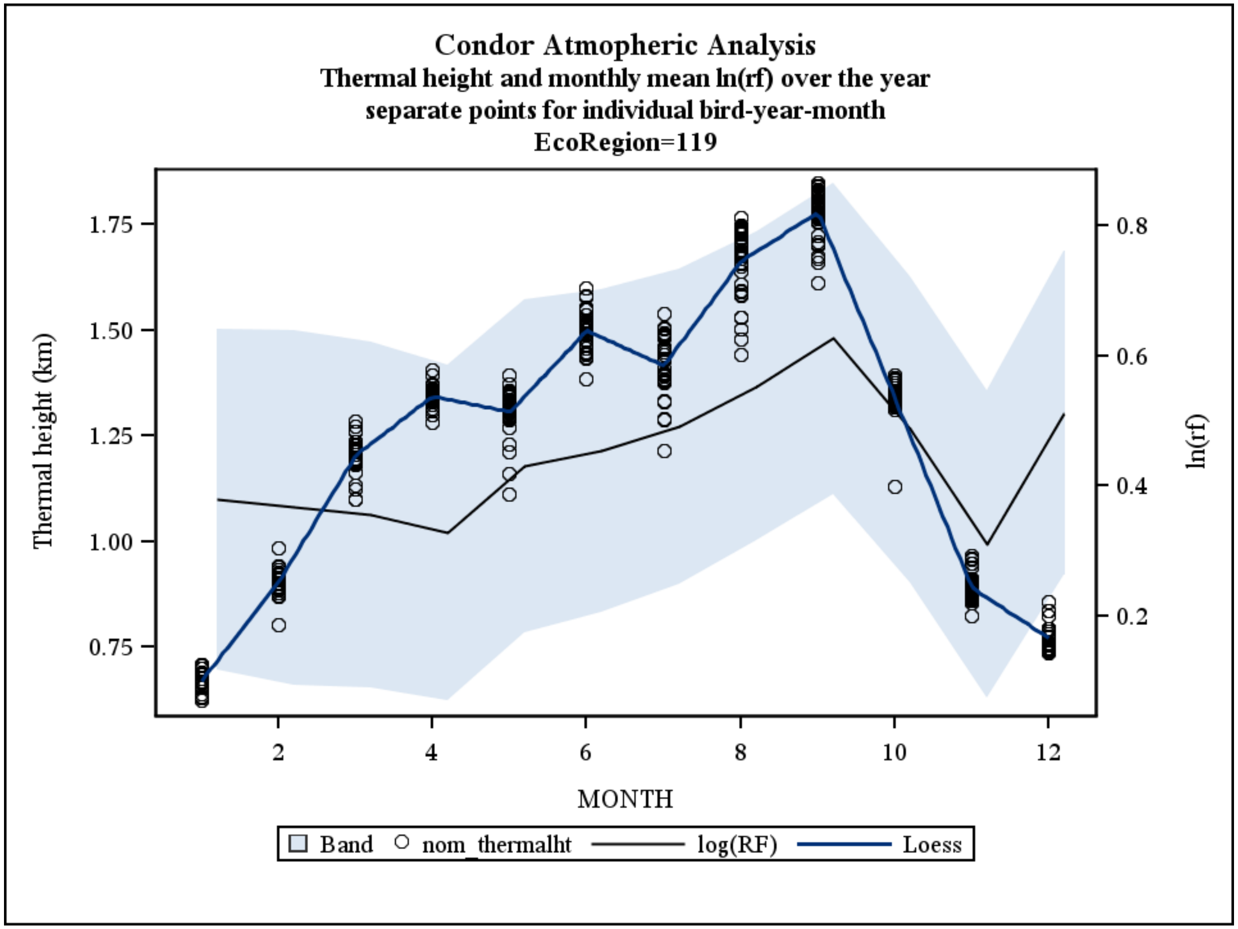

EcoRegion=123

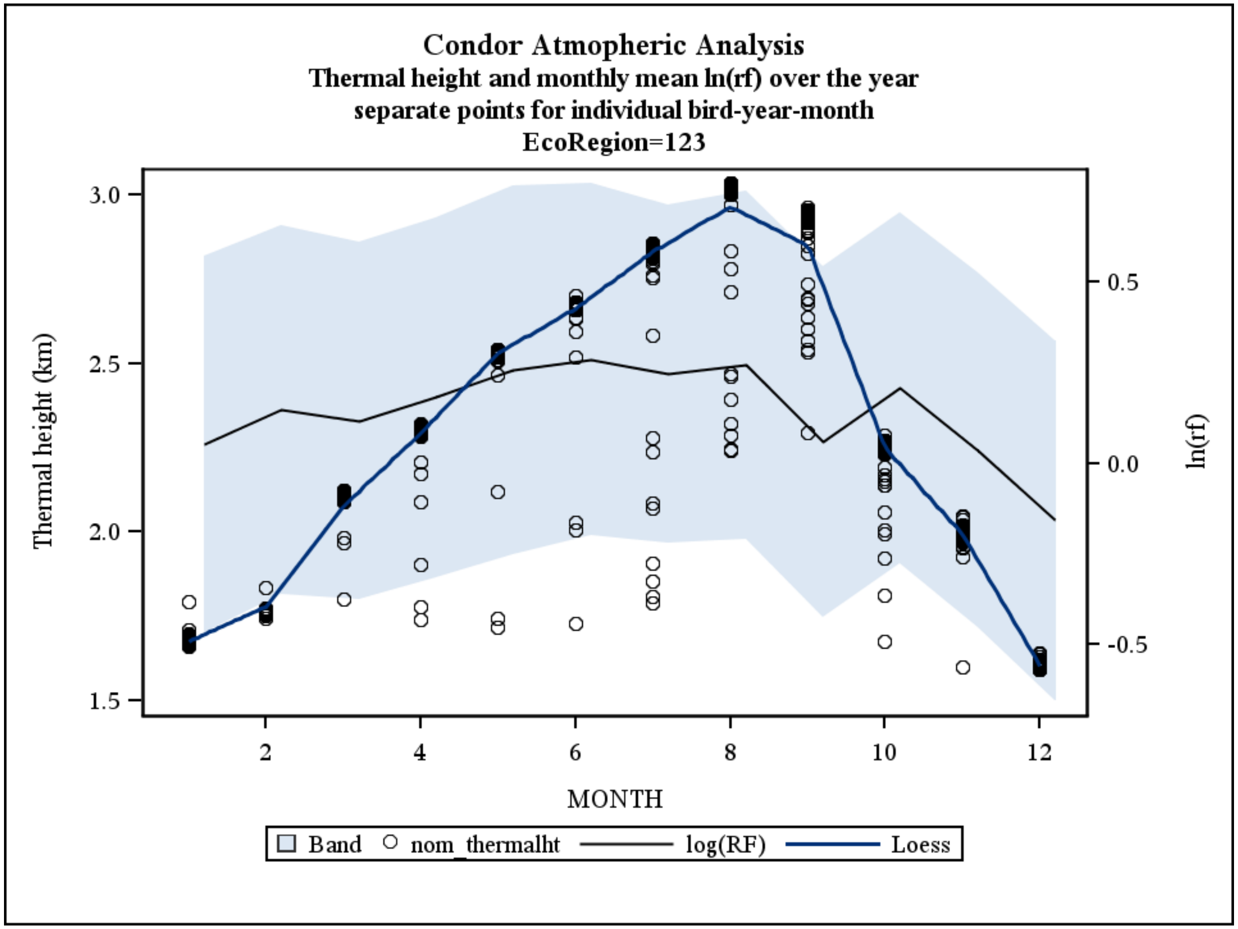

EcoRegion=124

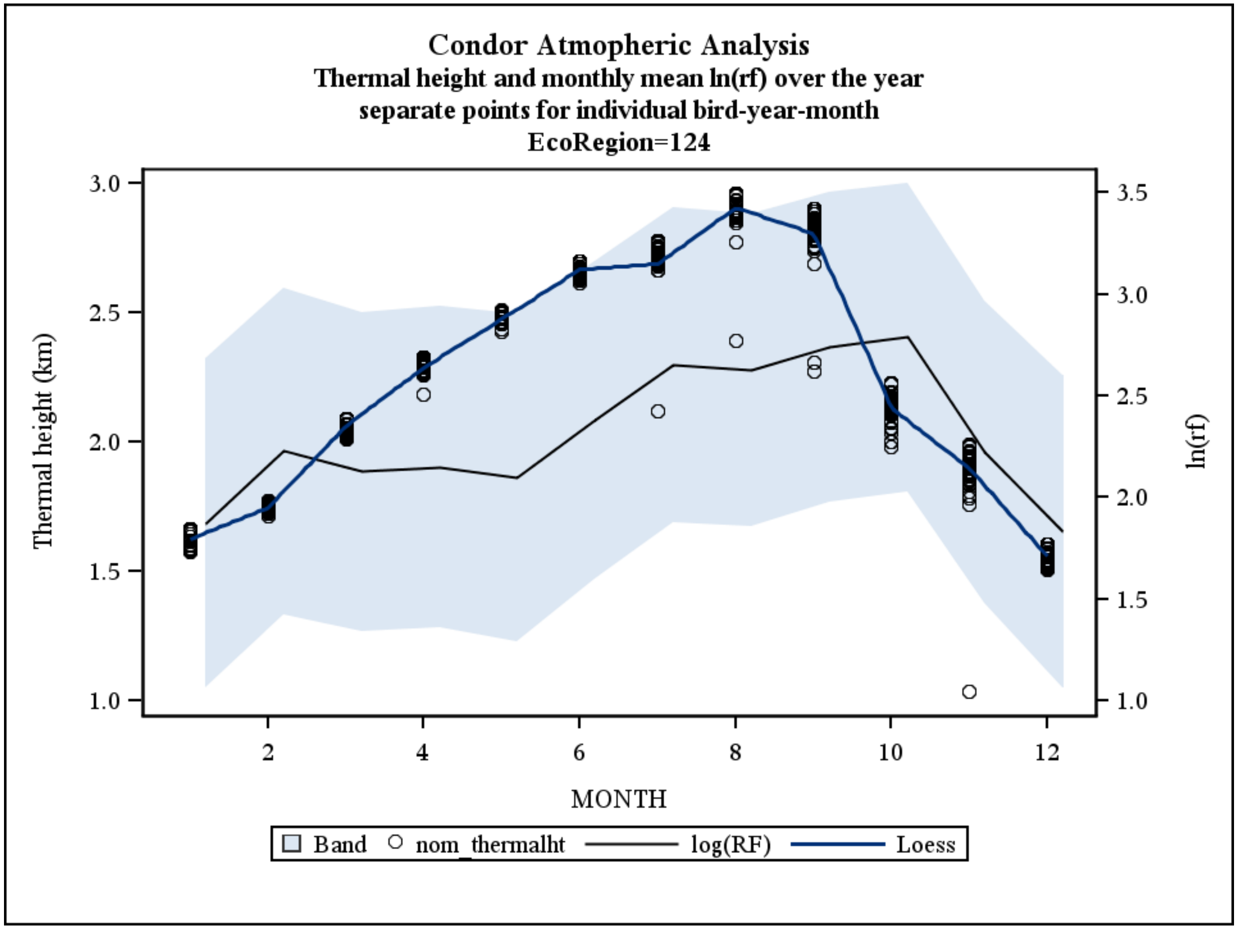

EcoRegion=125

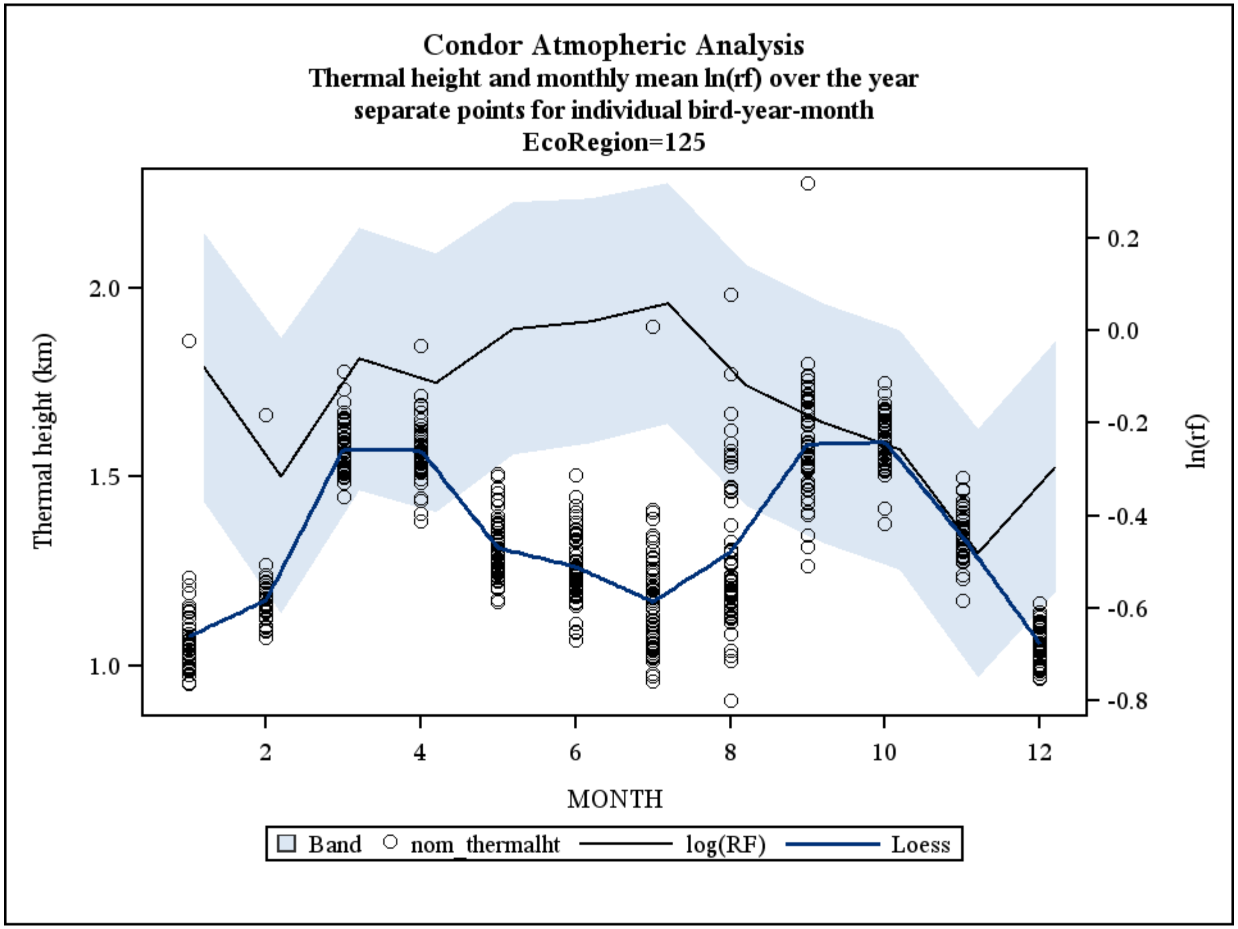

EcoRegion=126

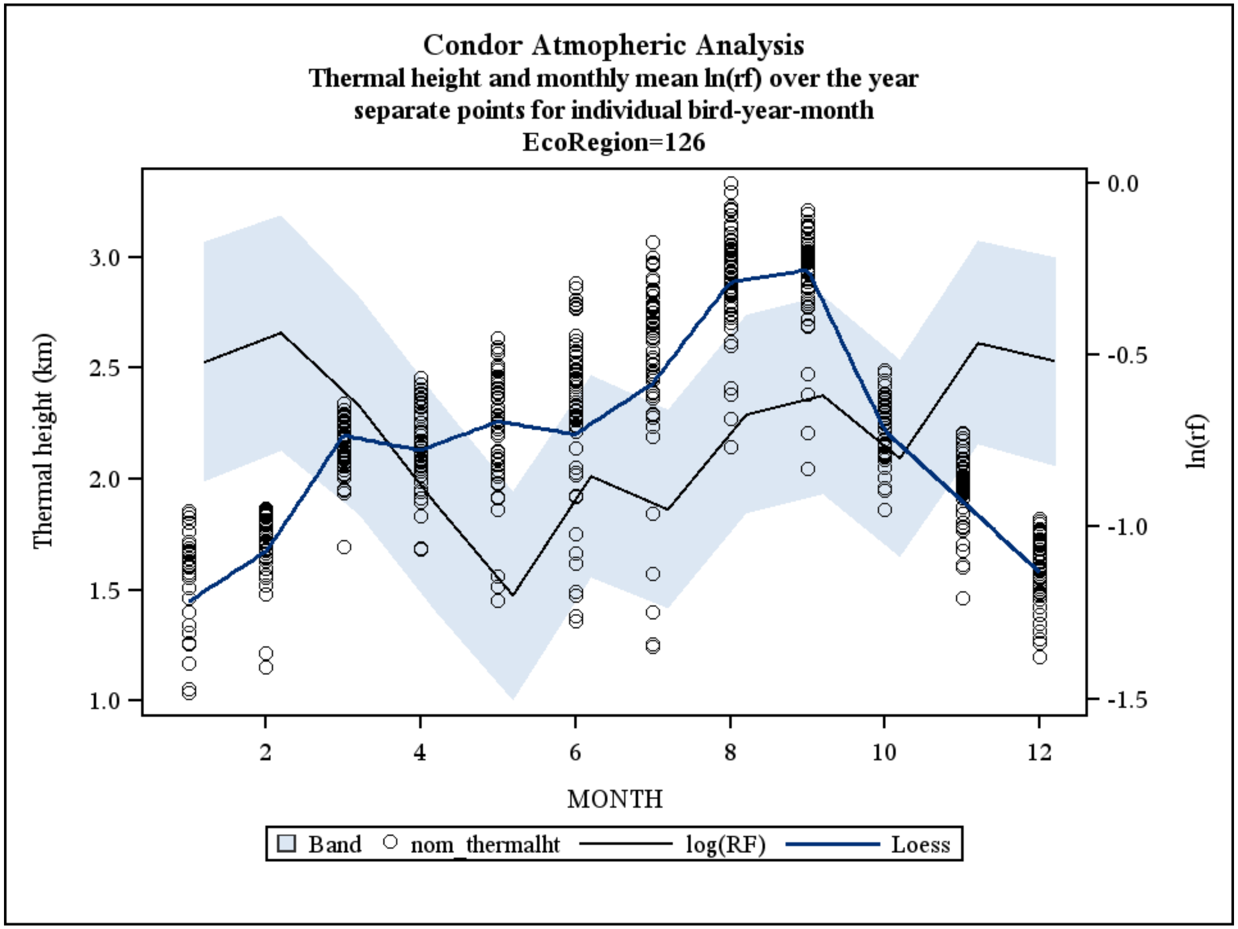

EcoRegion=127

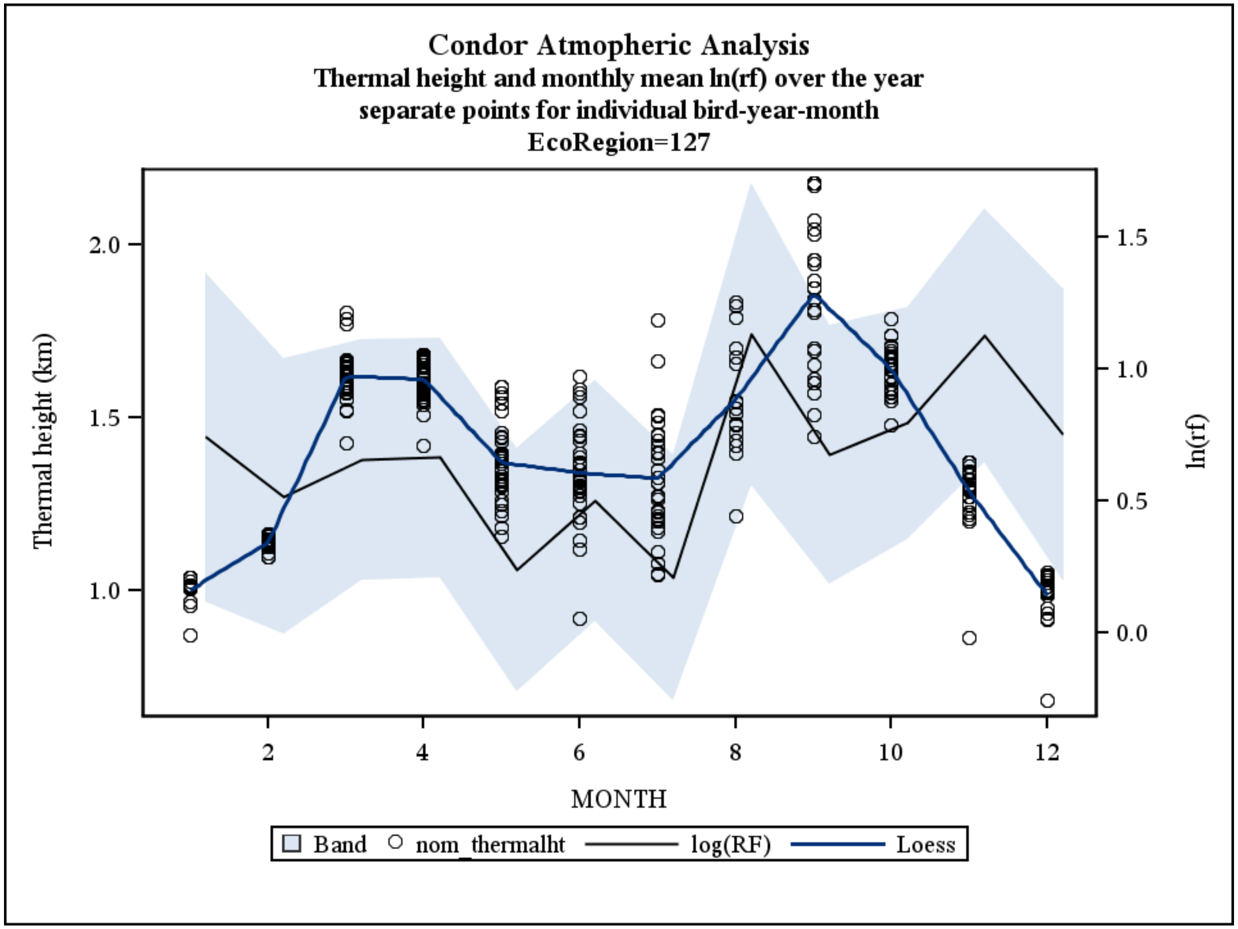

EcoRegion=128

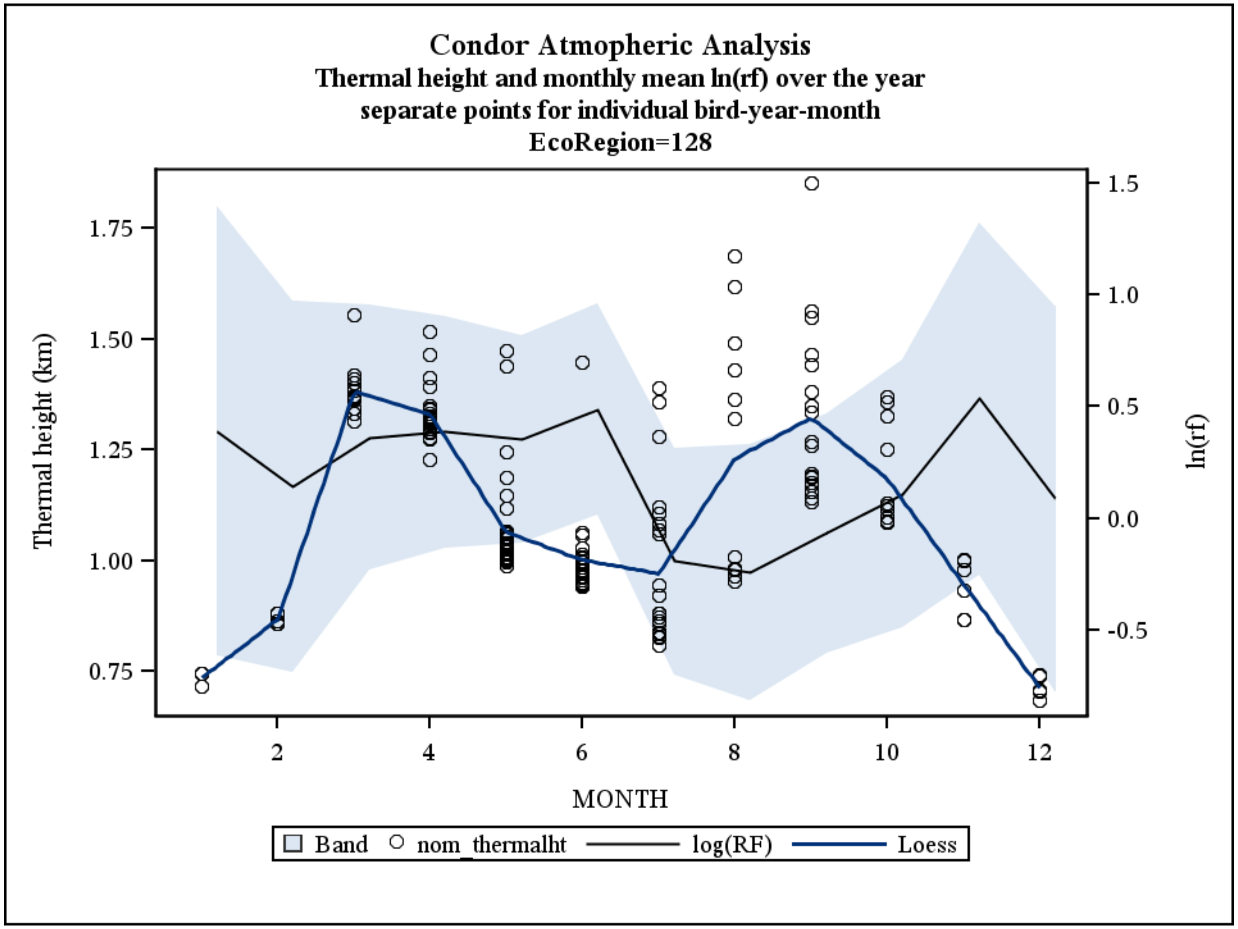

EcoRegion=147

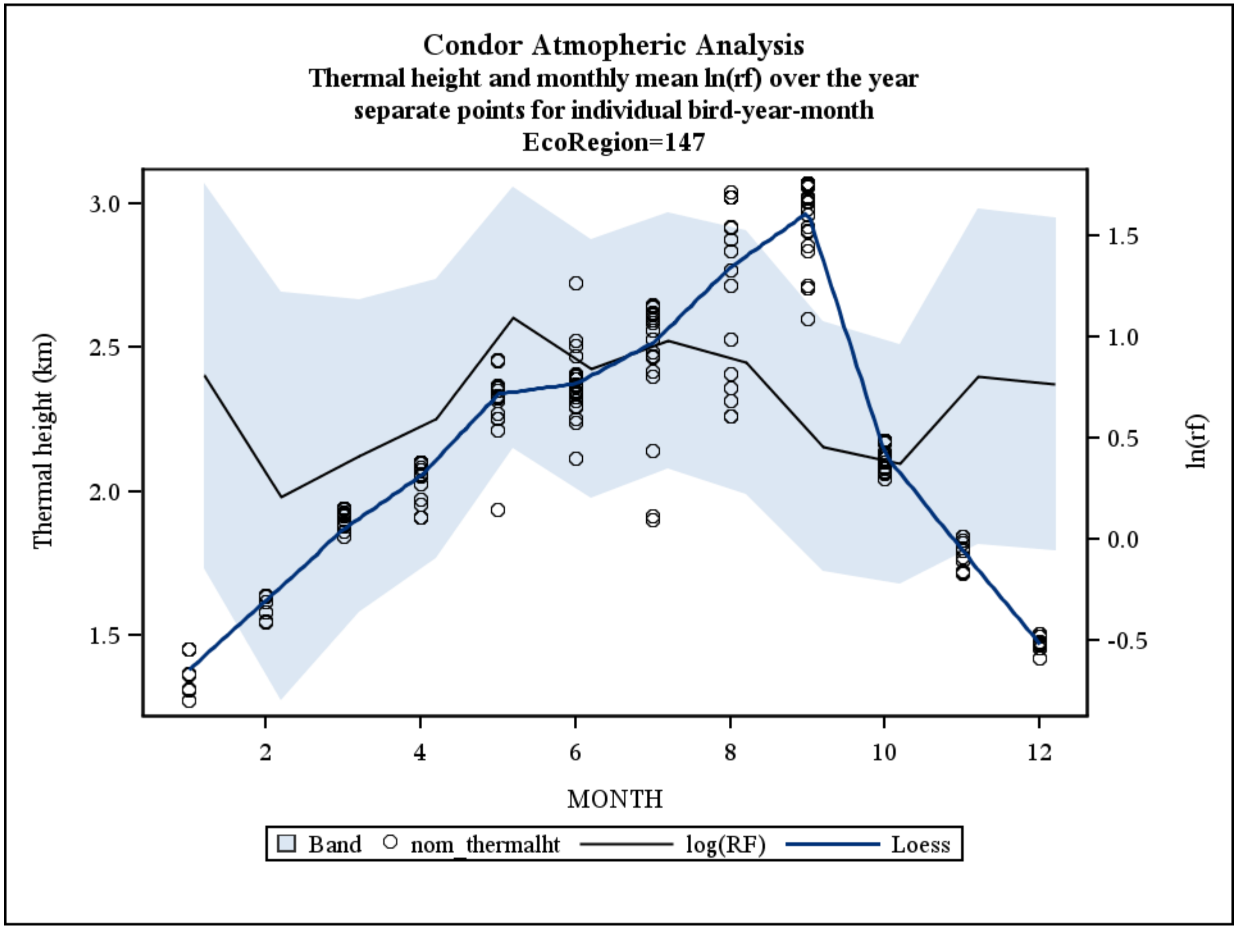

EcoRegion=192

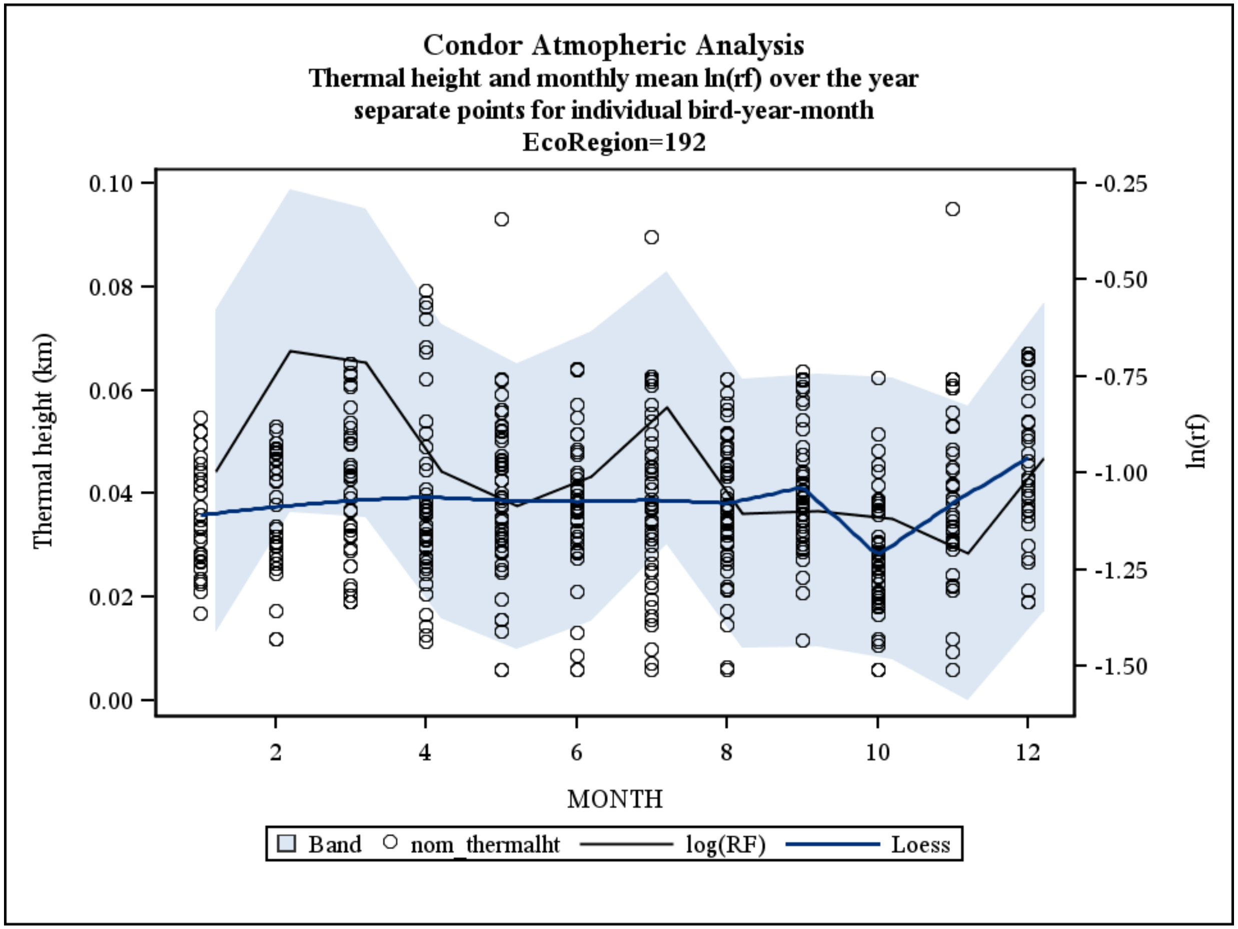

EcoRegion=193

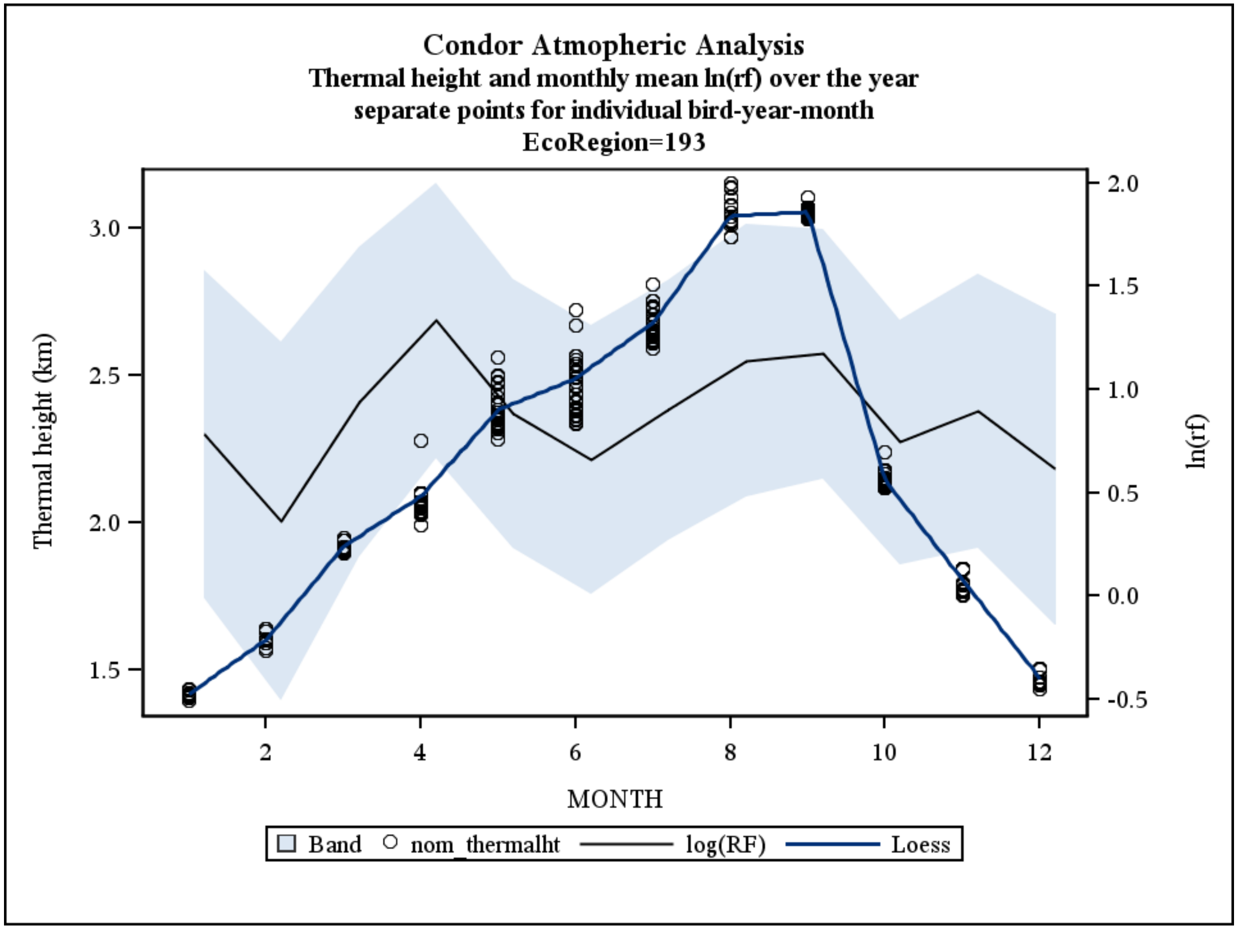

EcoRegion=8

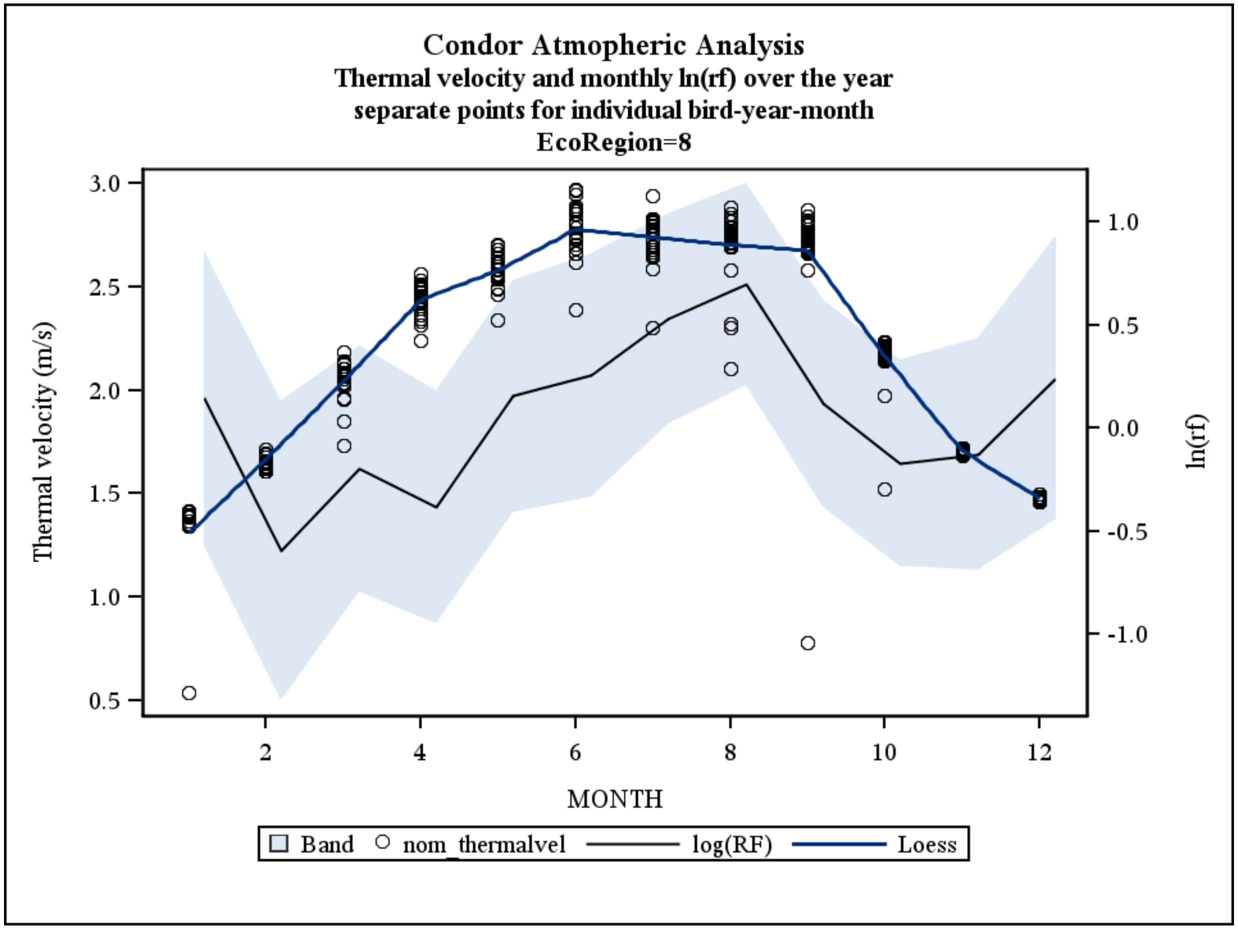
Condor Atmopheric Analysis
Thermal velocity and monthly ln(rf) over the year
separate points for individual bird-year-month
EcoRegion=8
Thermal velocity (m/s)
3.0
2.5
2.0
1.5
1.0
0.5
ln(rf)
1.0
0.5
0.0
-0.5
-1.0
2
4
6
8
10
12
MONTH
Band
nom_thermalvel
log(RF)
Loess

EcoRegion=9

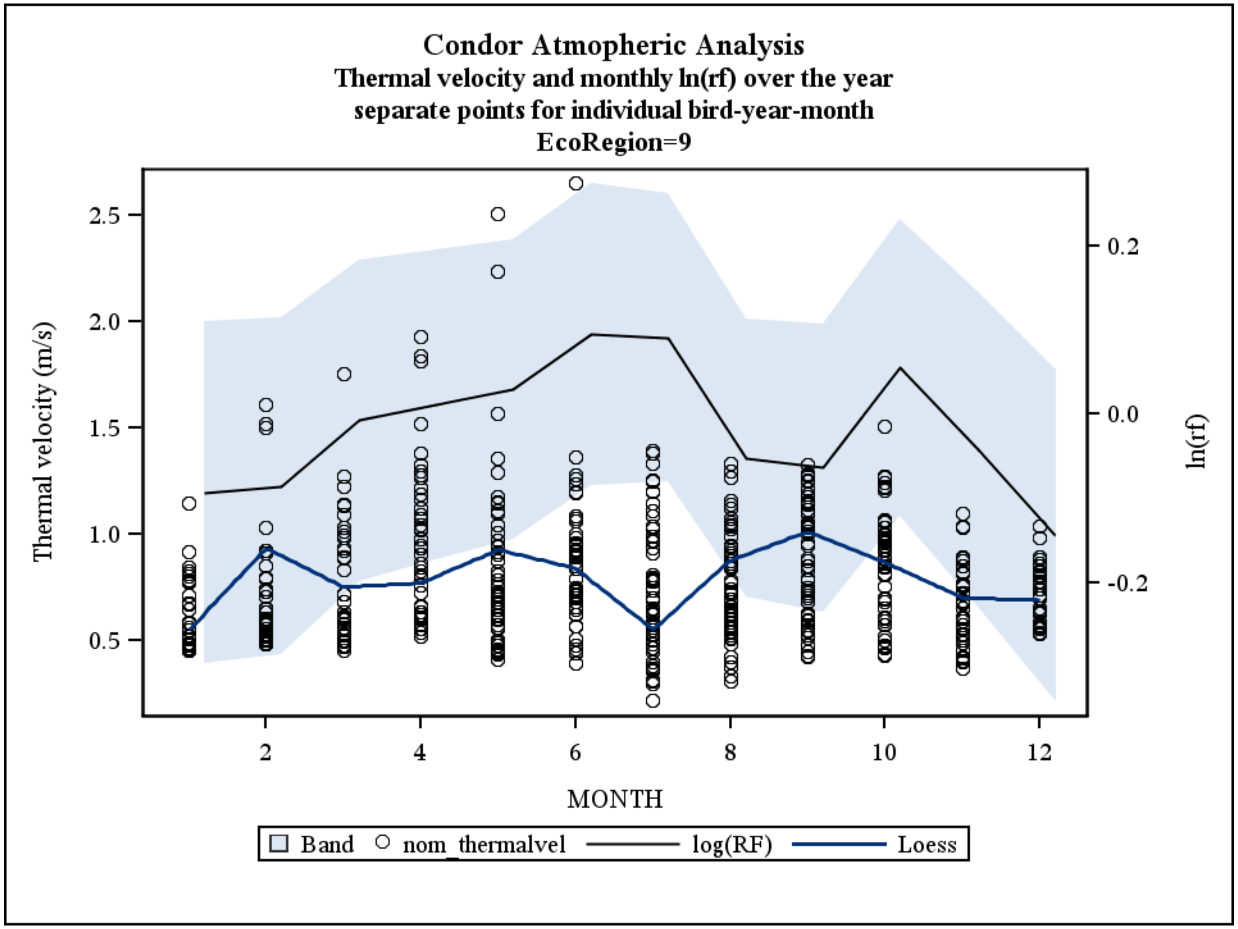
Condor Atmopheric Analysis
Thermal velocity and monthly ln(rf) over the year
separate points for individual bird-year-month
EcoRegion=9
Thermal velocity (m/s)
2.5
2.0
1.5
1.0
0.5
0.2
0.0
-0.2
ln(rf)
2
4
6
8
10
12
MONTH
Band
nom_thermalvel
log(RF)
Loess

EcoRegion=10

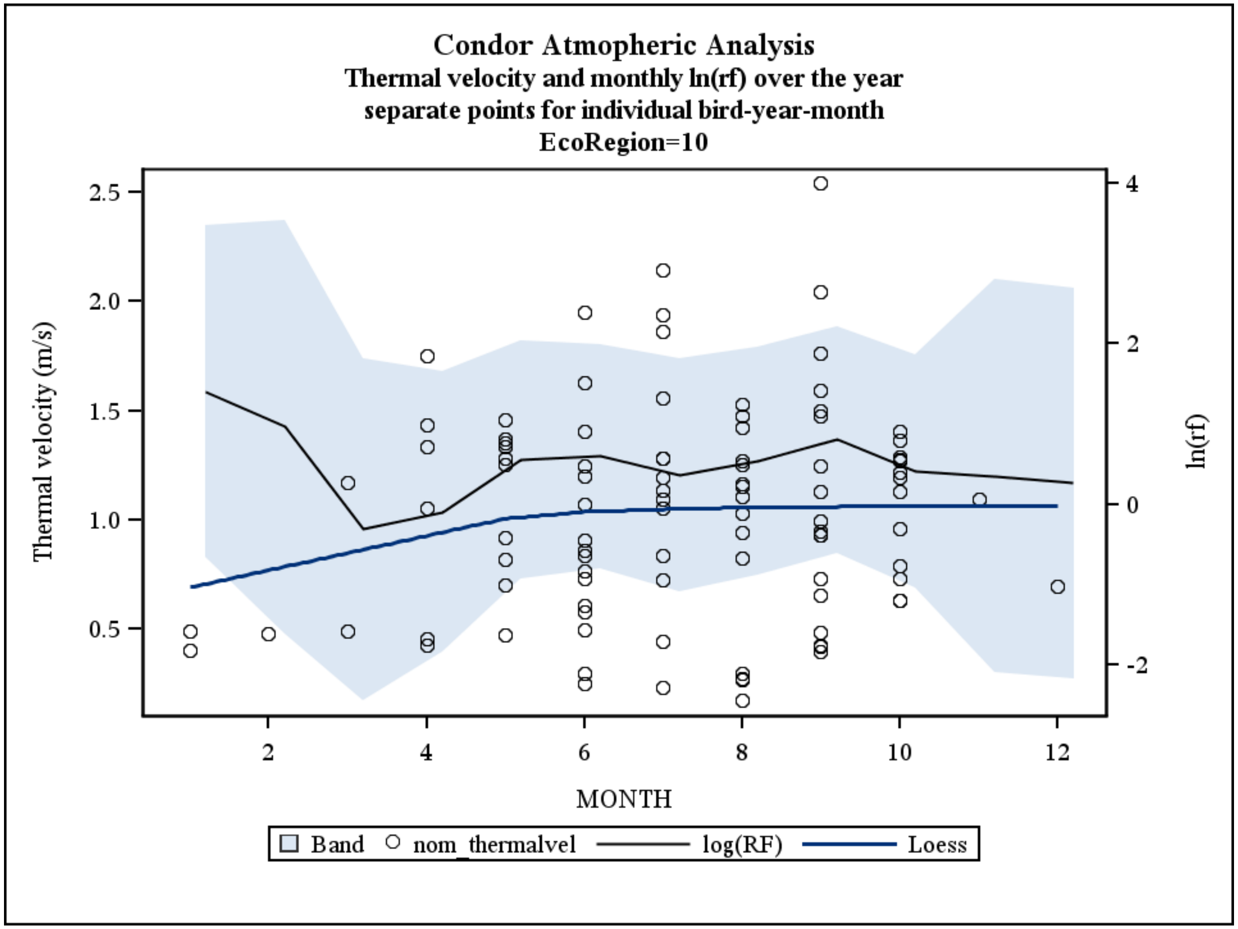

EcoRegion=13

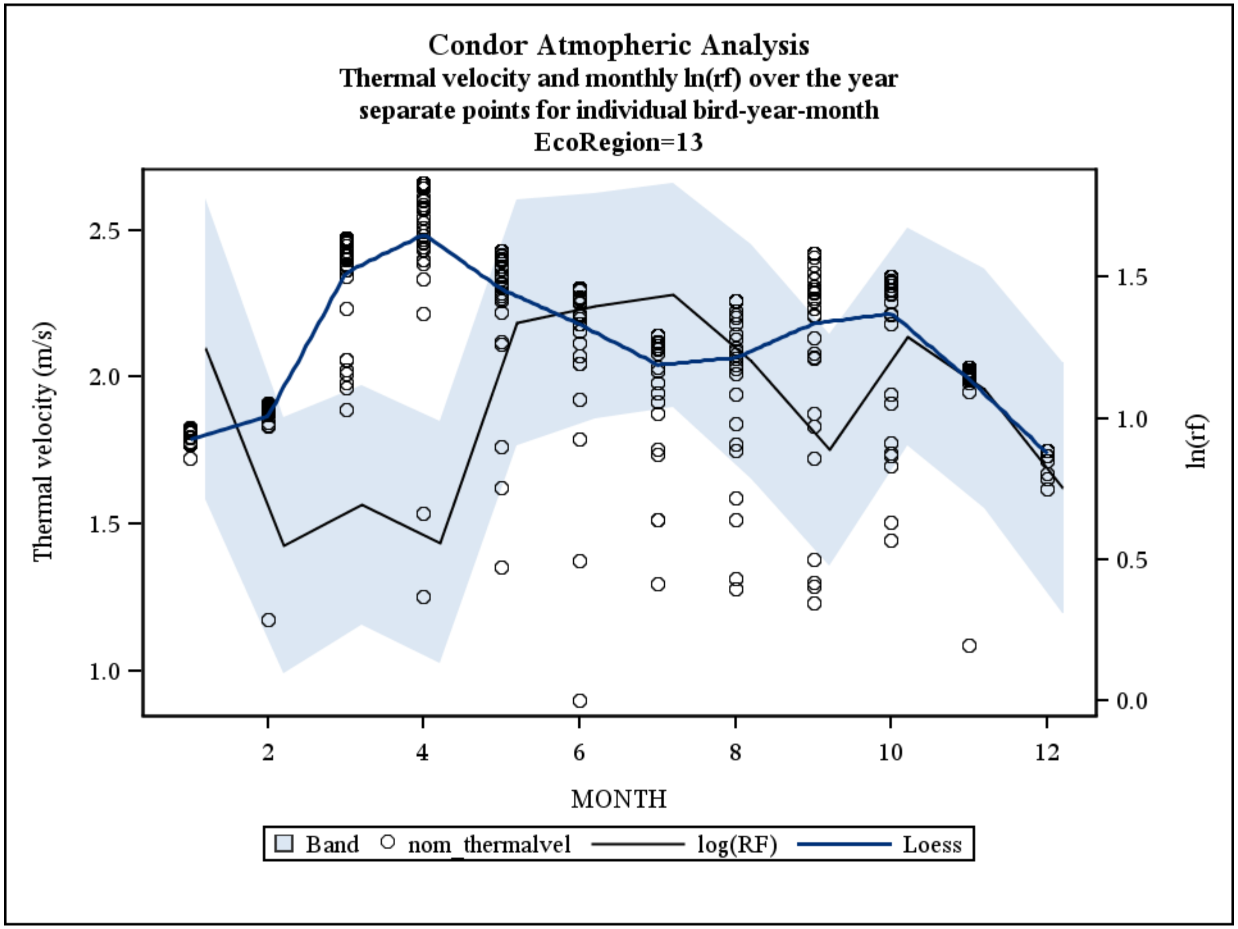

EcoRegion=15

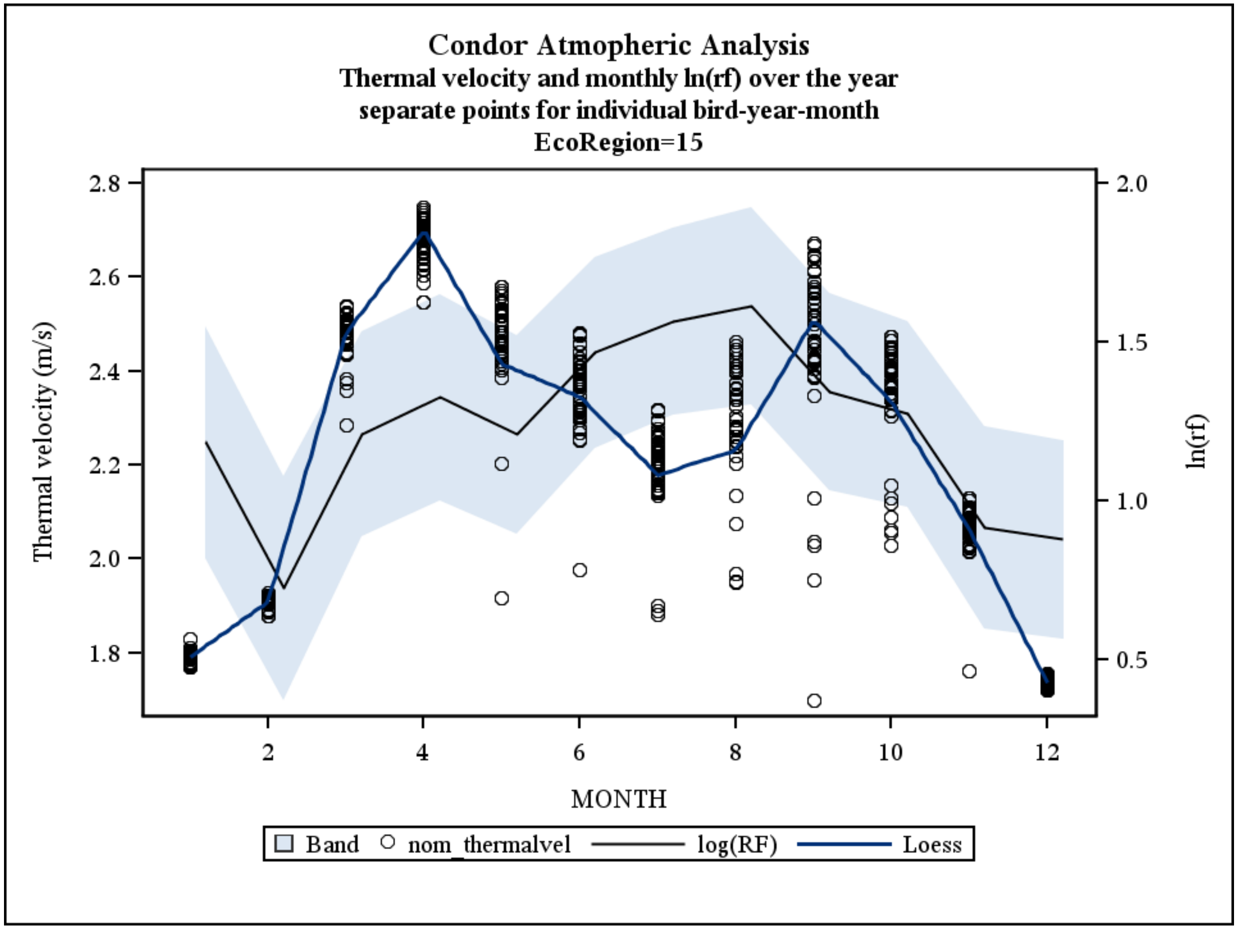

EcoRegion=16

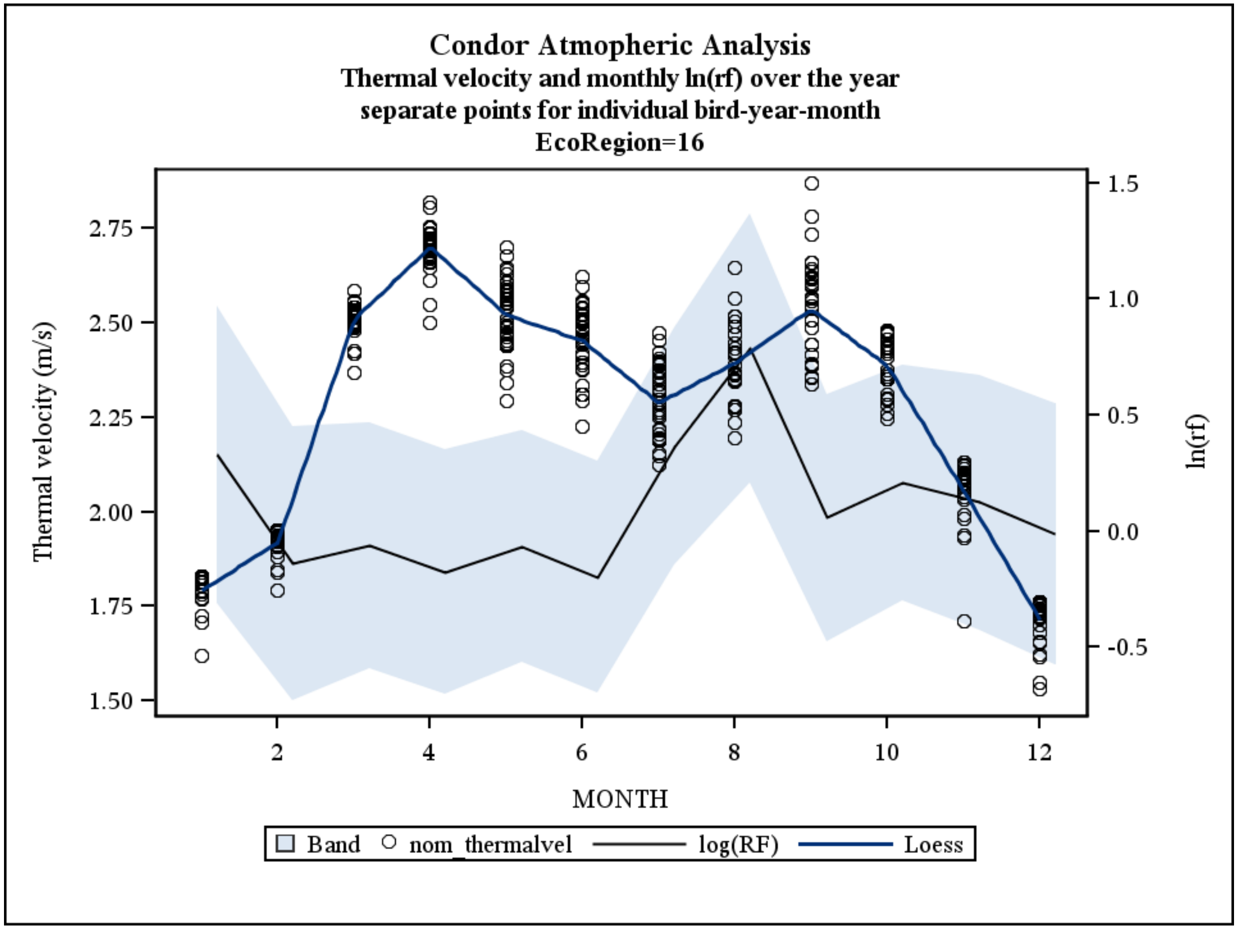

EcoRegion=18

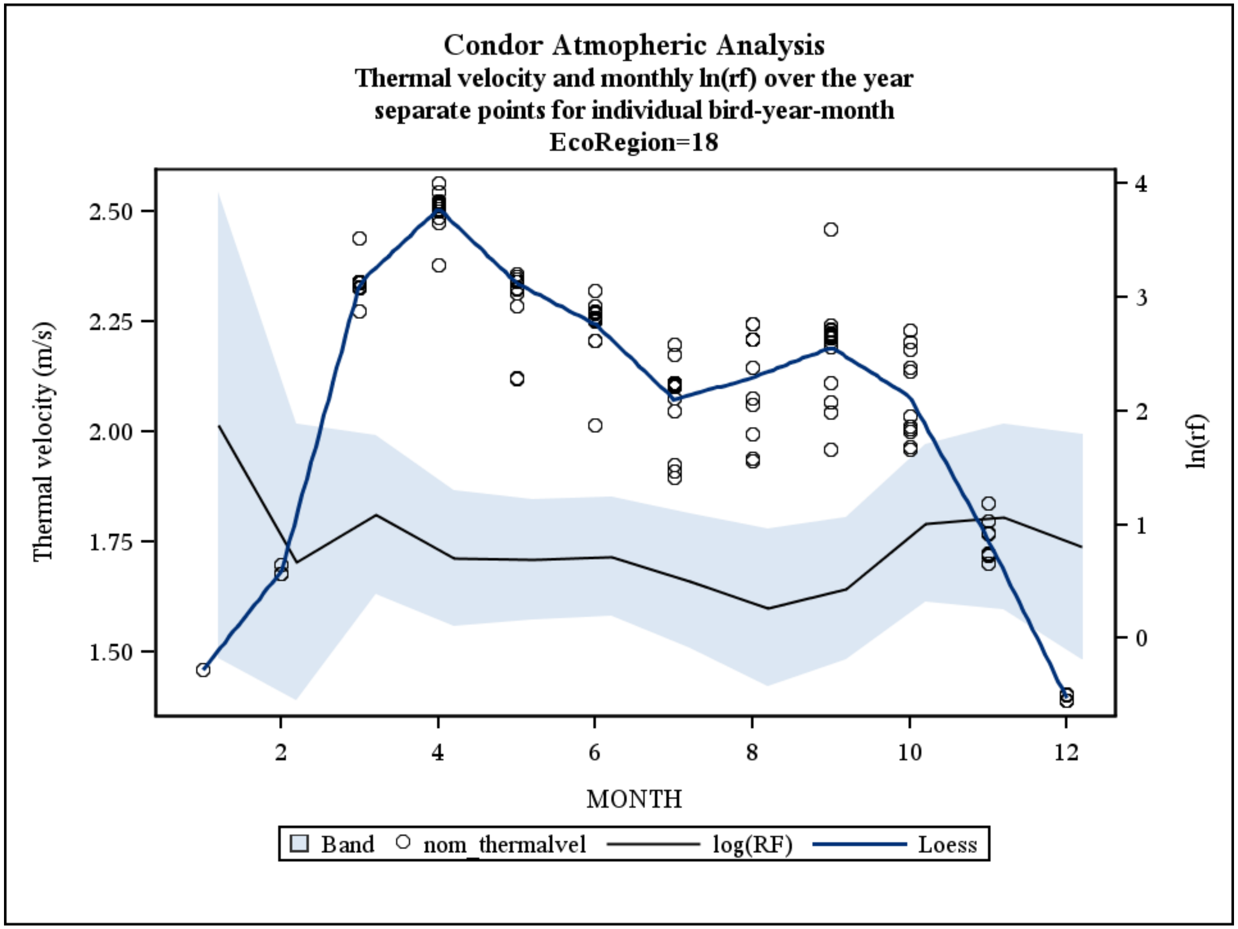

EcoRegion=39

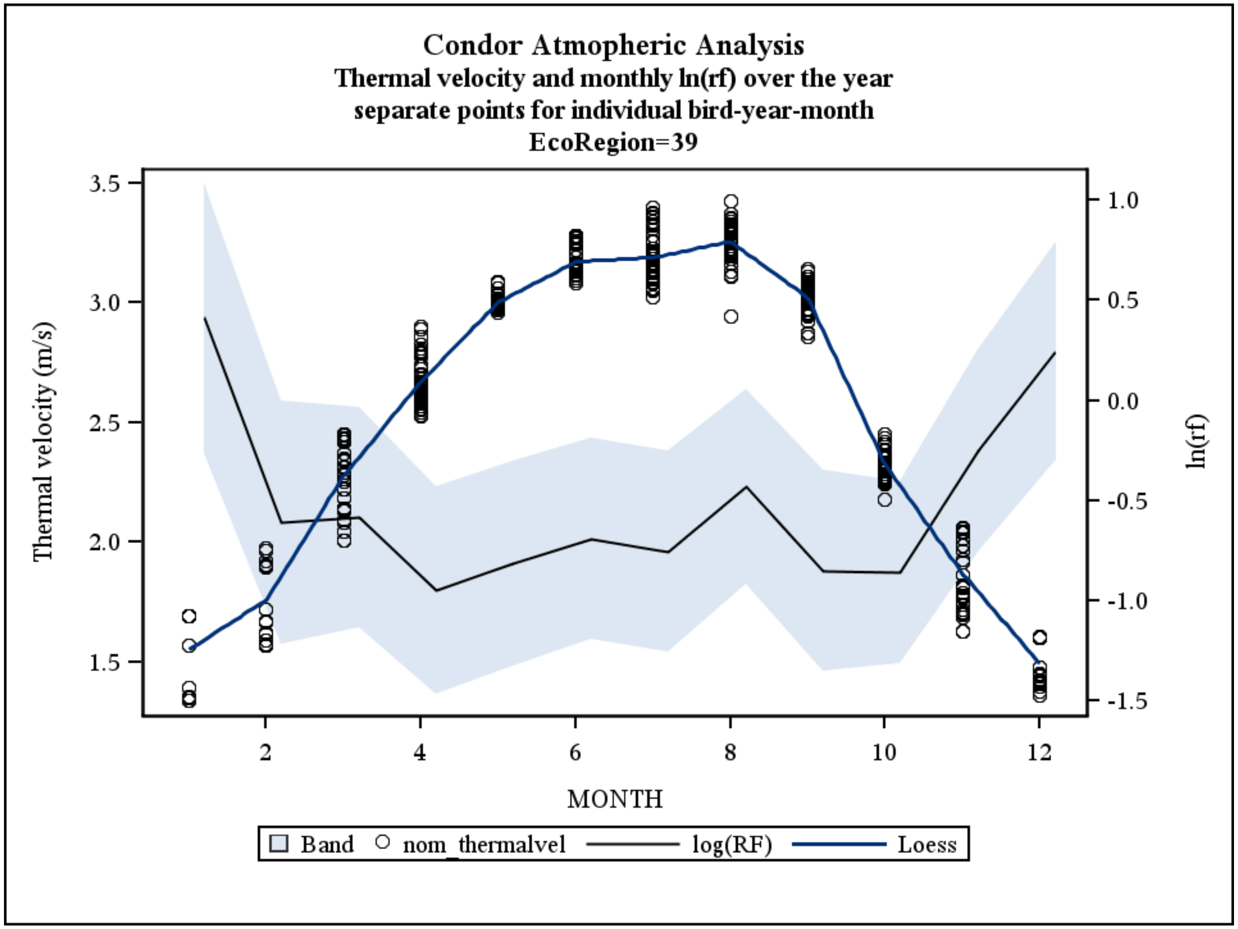

EcoRegion=40

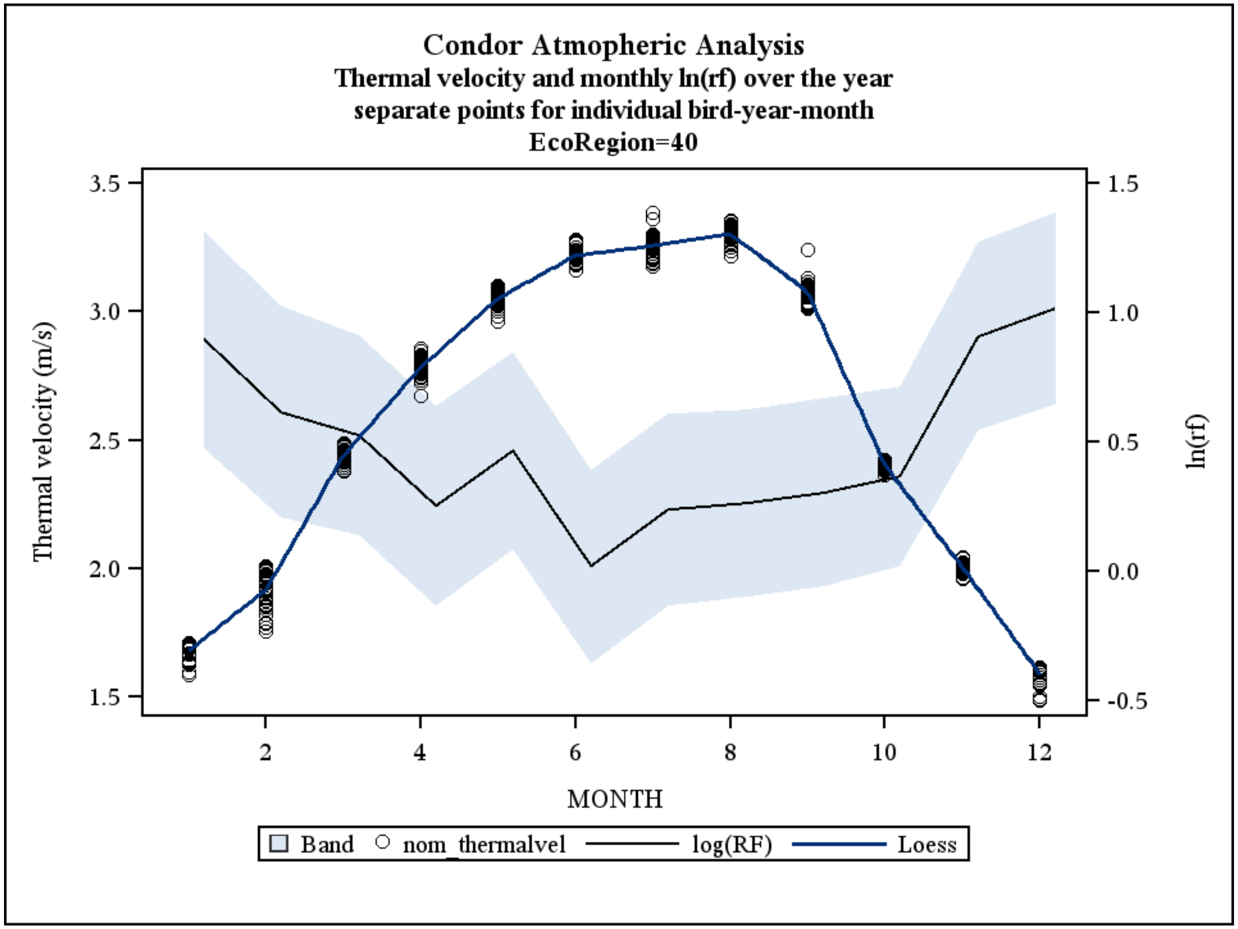

EcoRegion=95

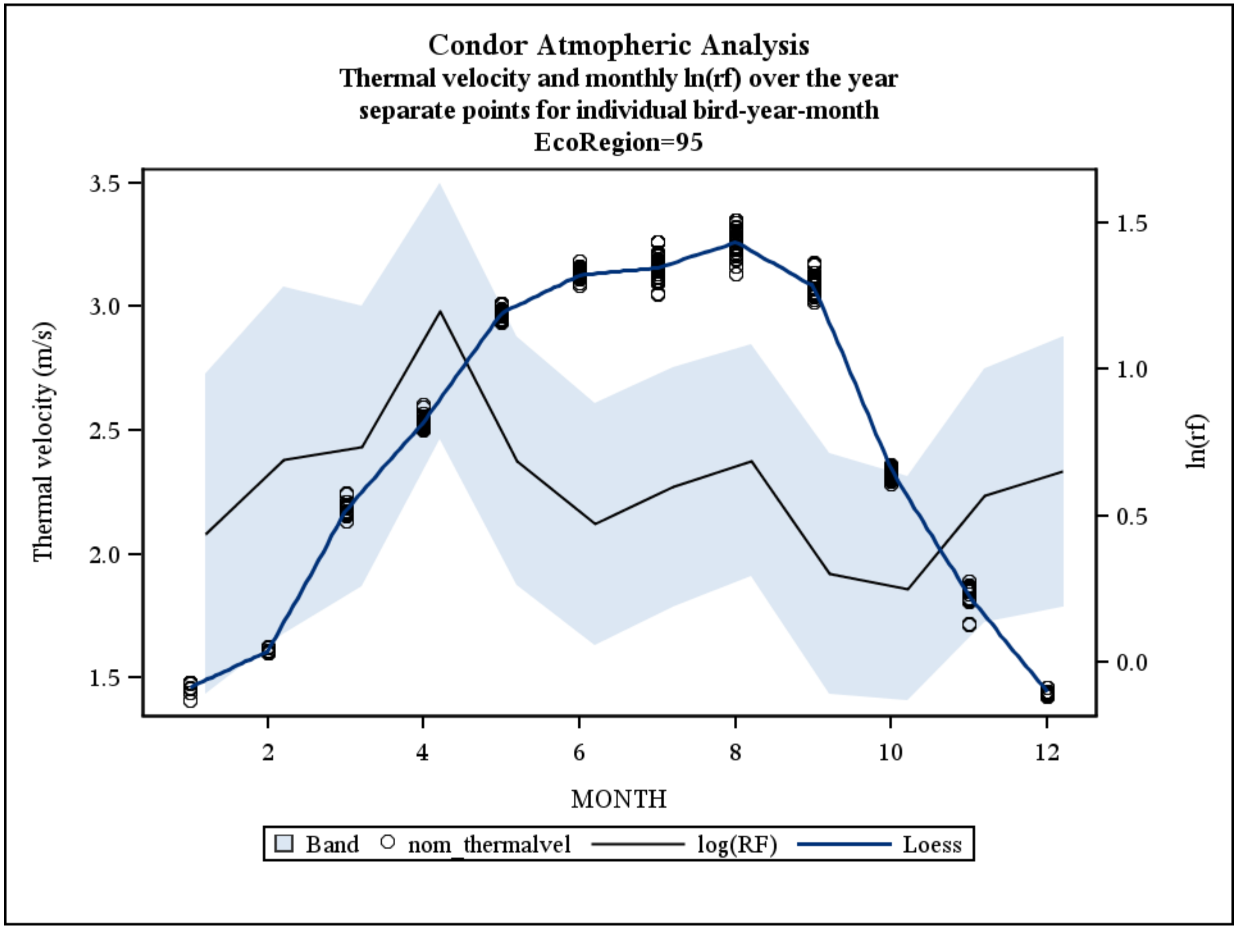

EcoRegion=101

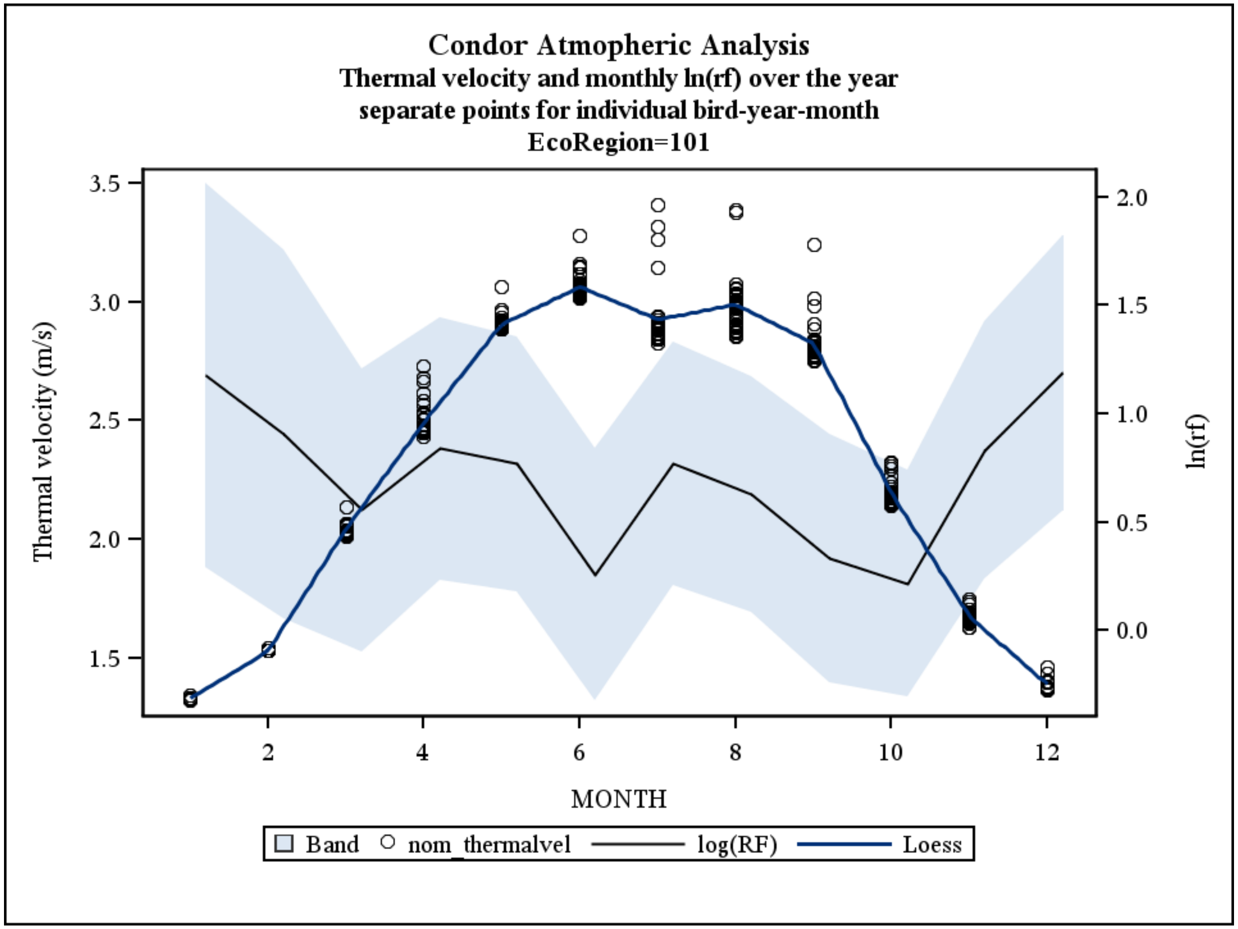

EcoRegion=102

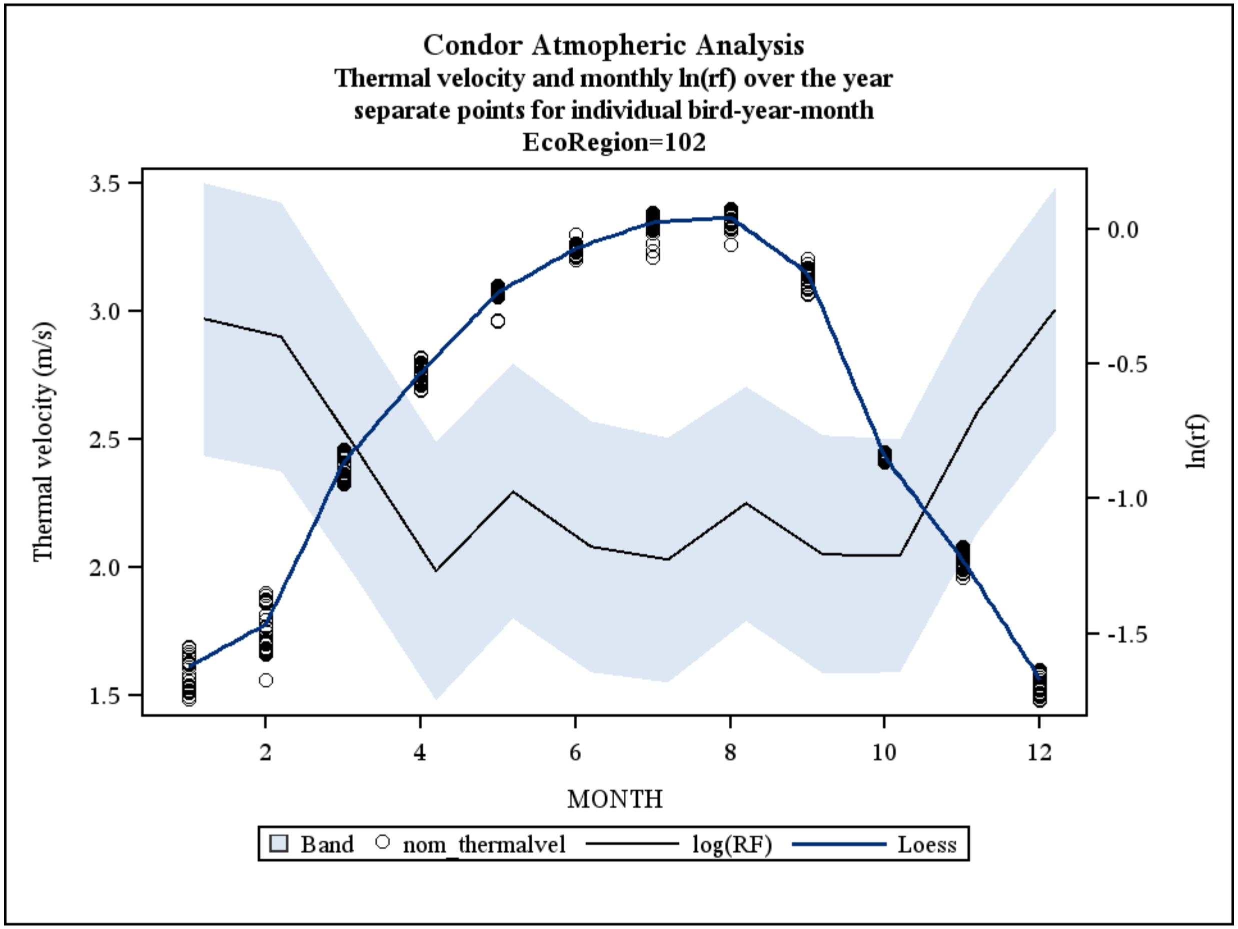

EcoRegion=116

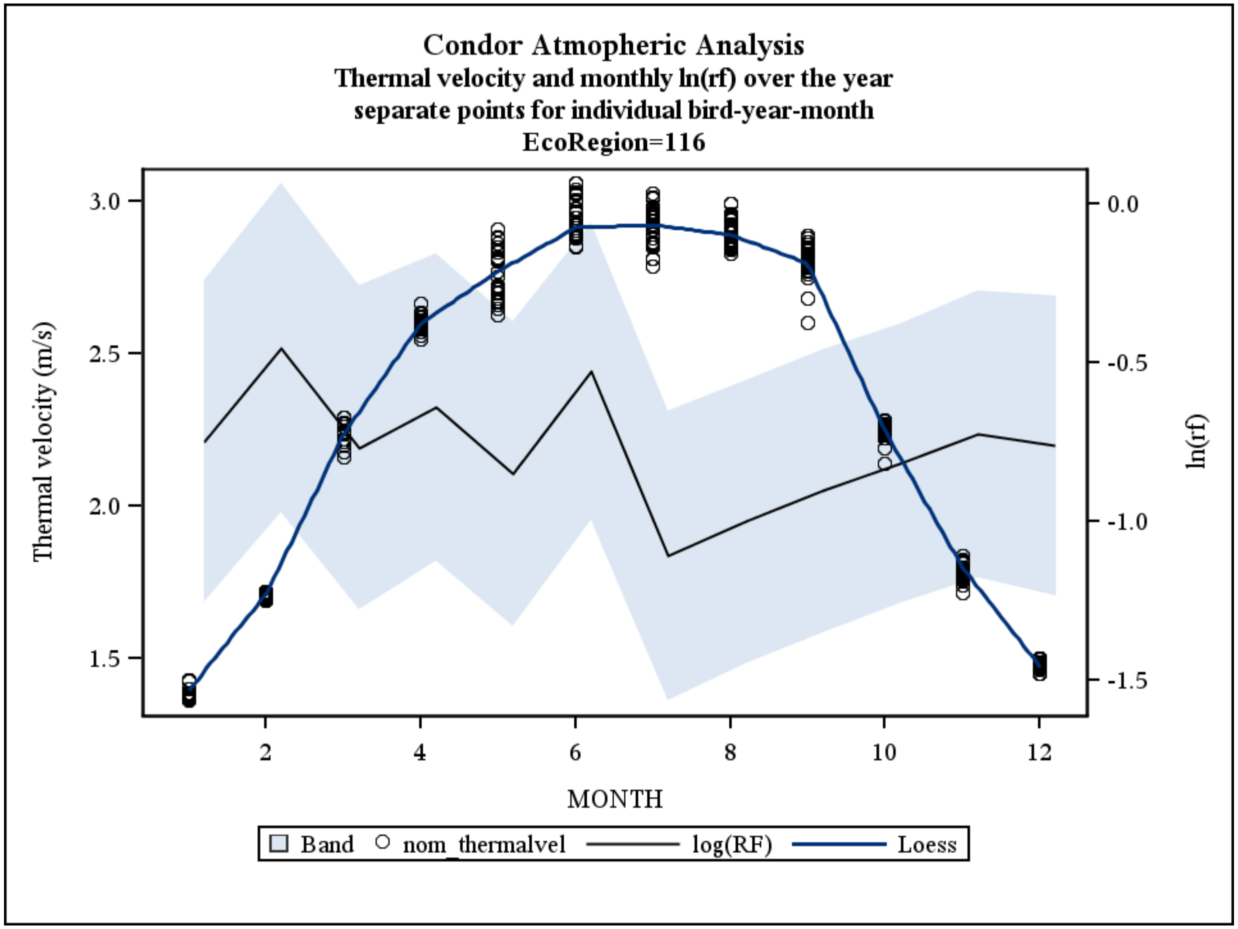

EcoRegion=117

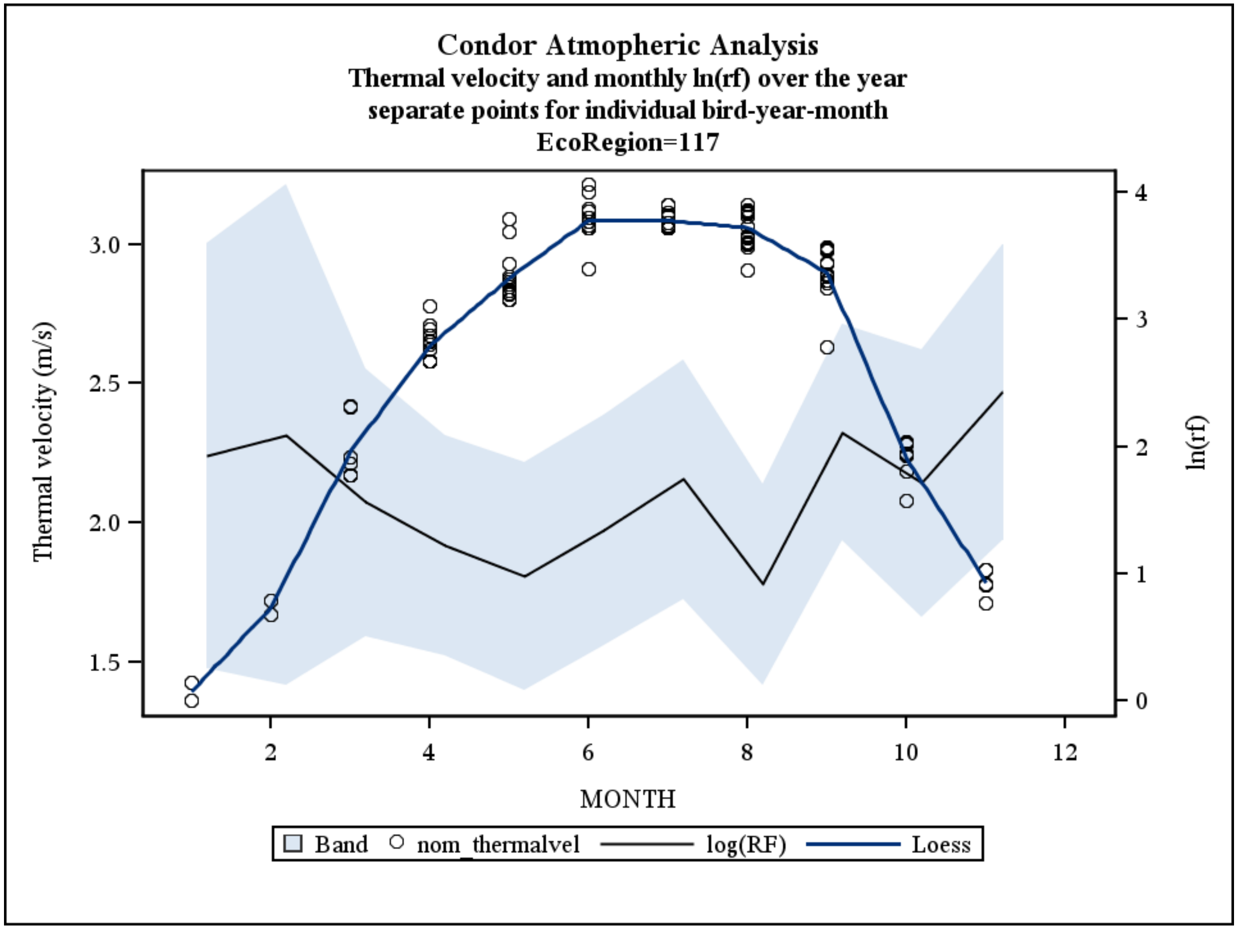

EcoRegion=118

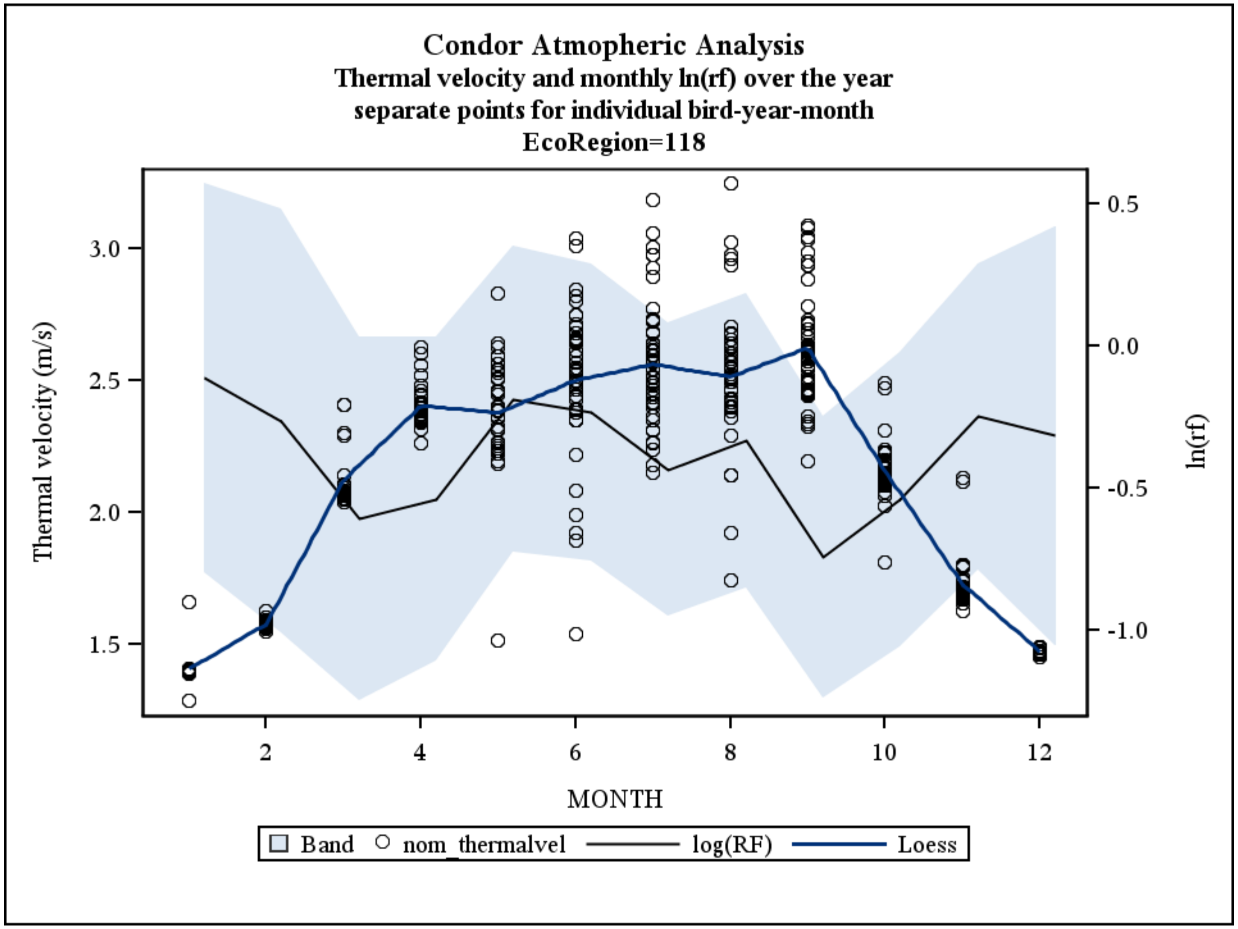

EcoRegion=119

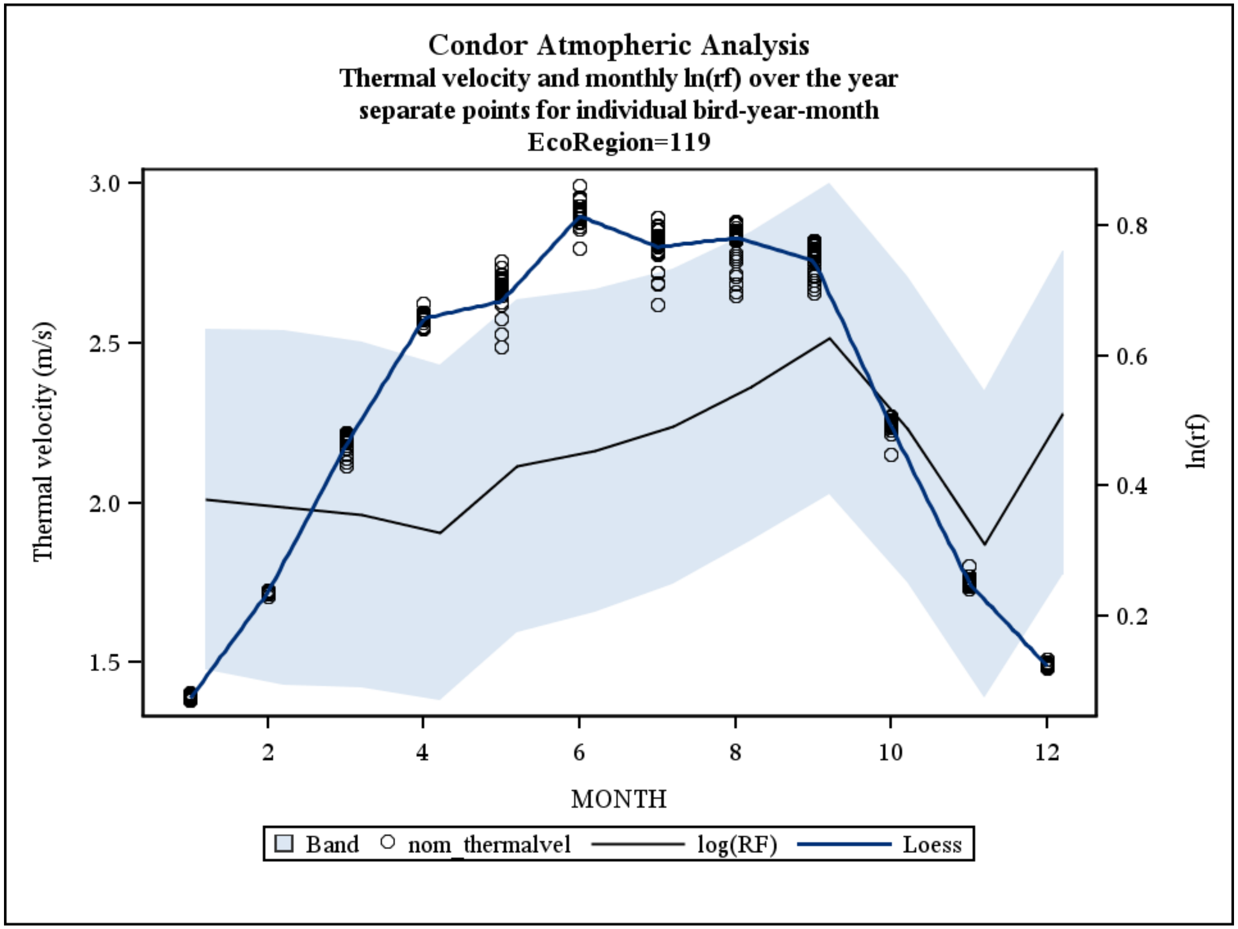

EcoRegion=123

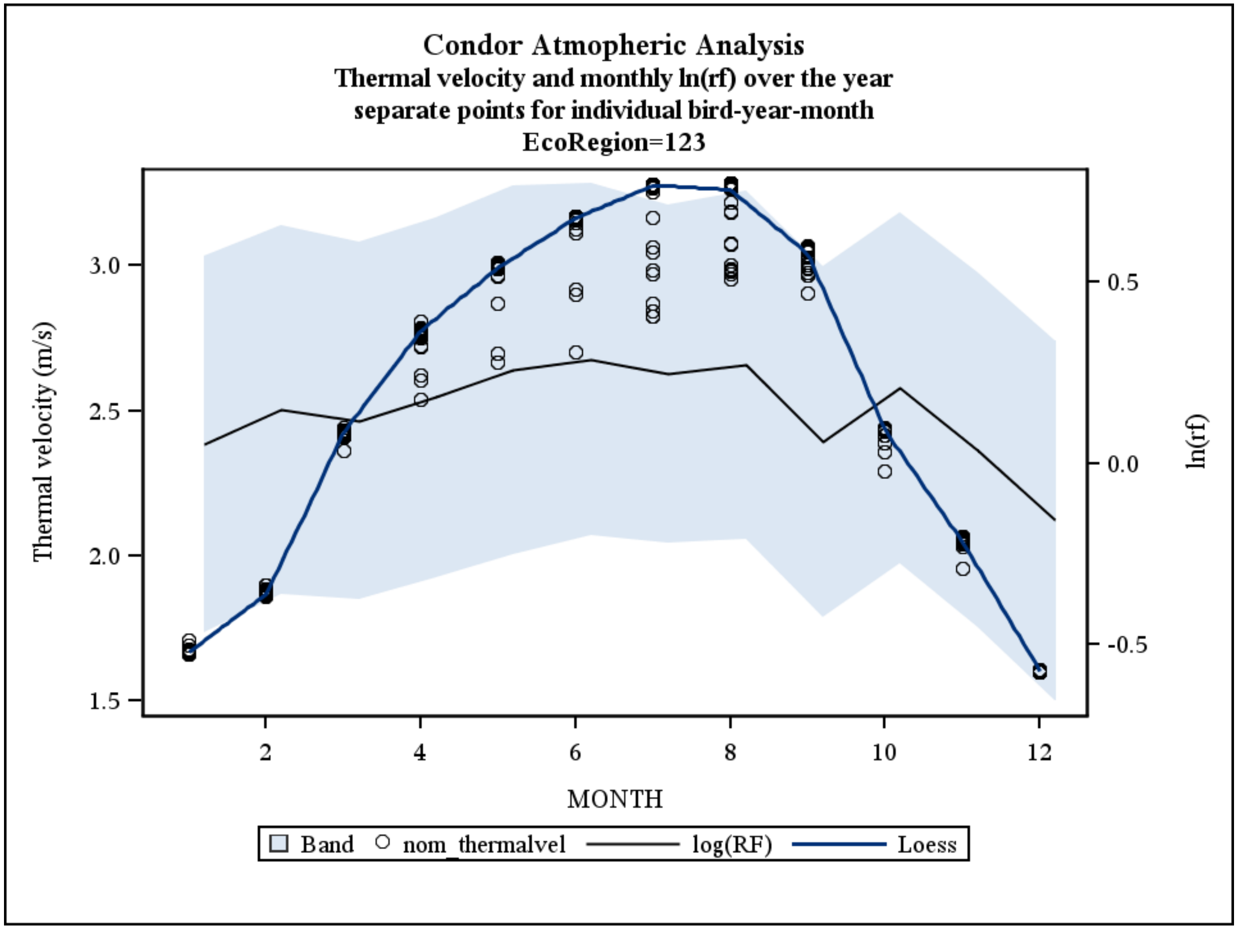

EcoRegion=124

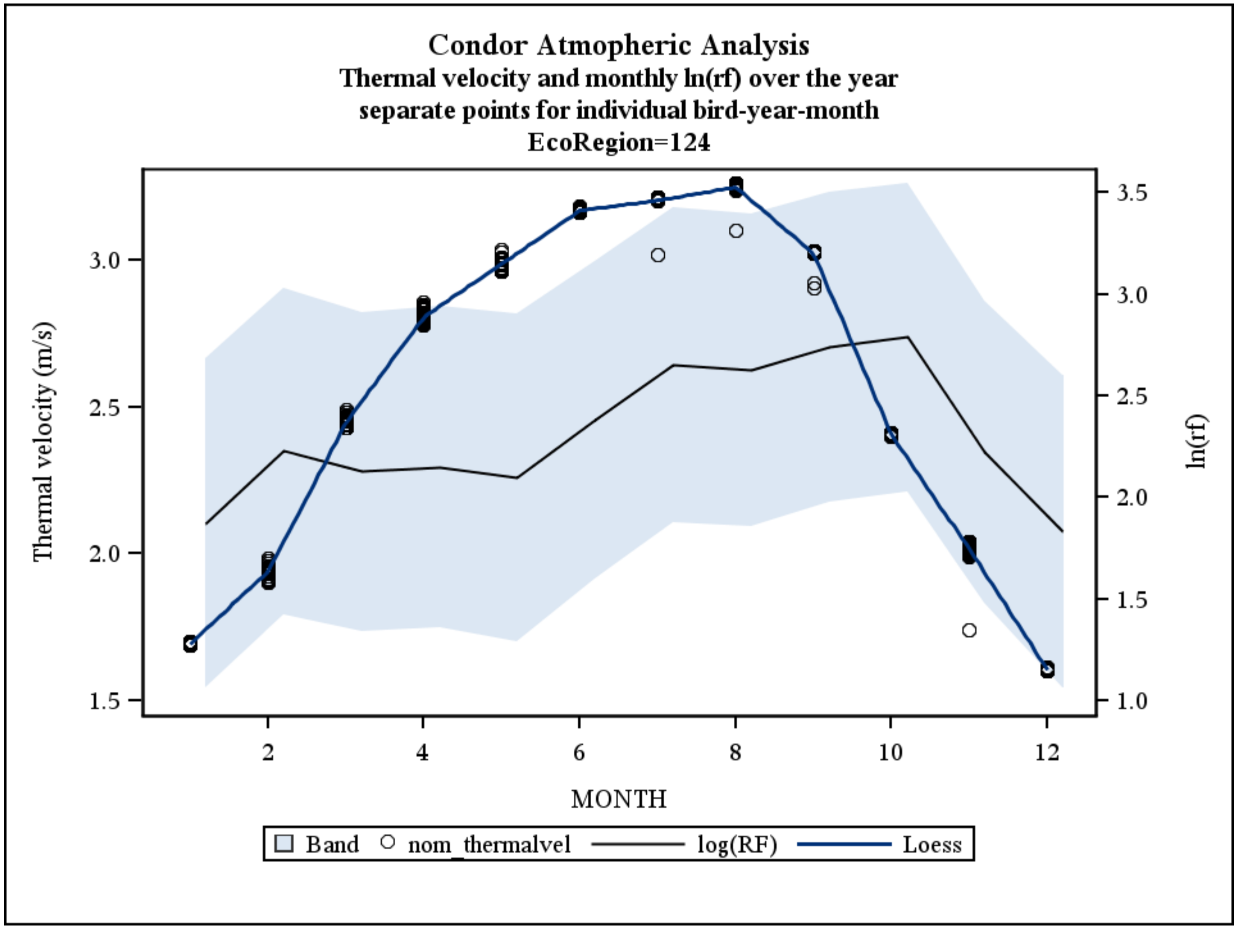

EcoRegion=125

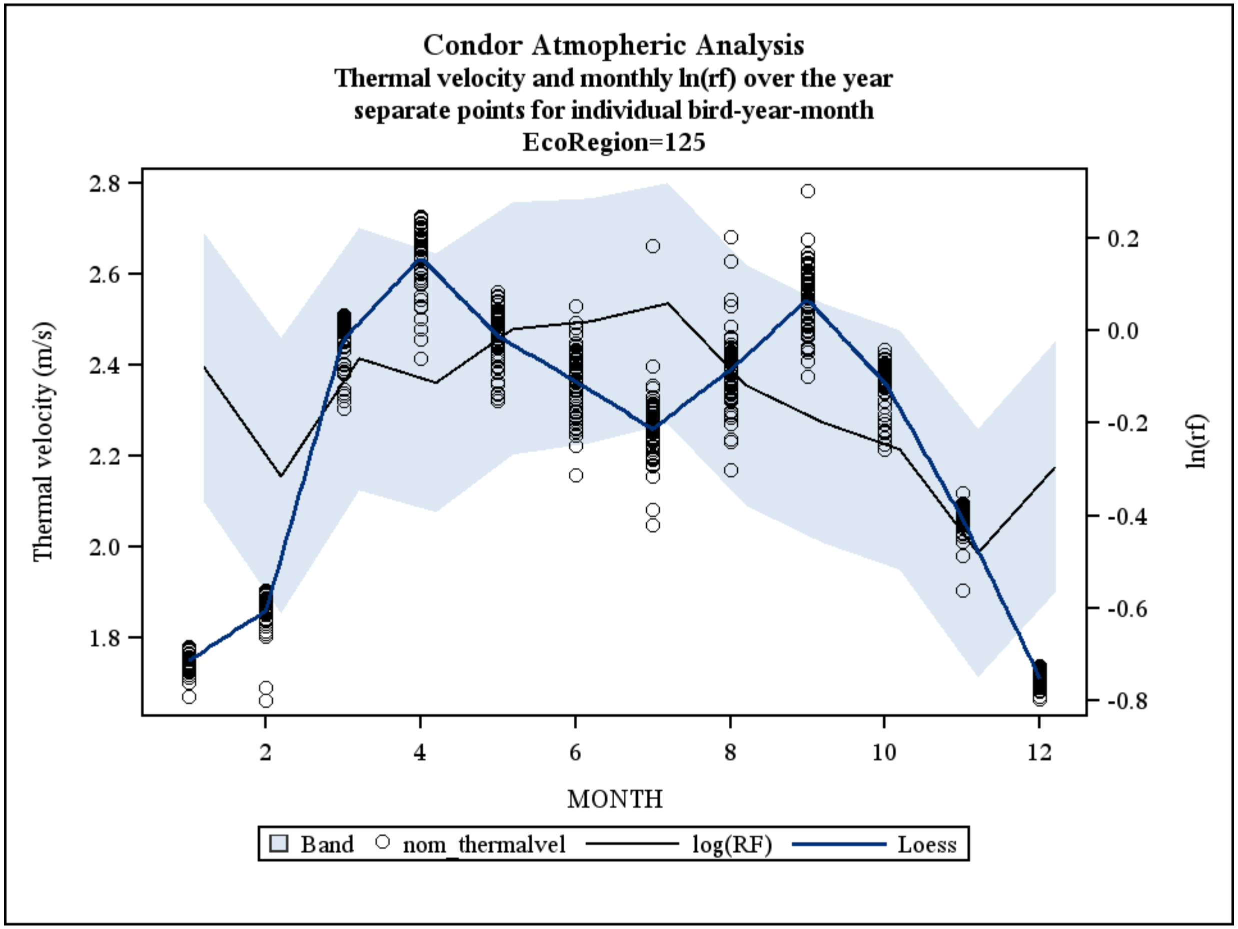

EcoRegion=126

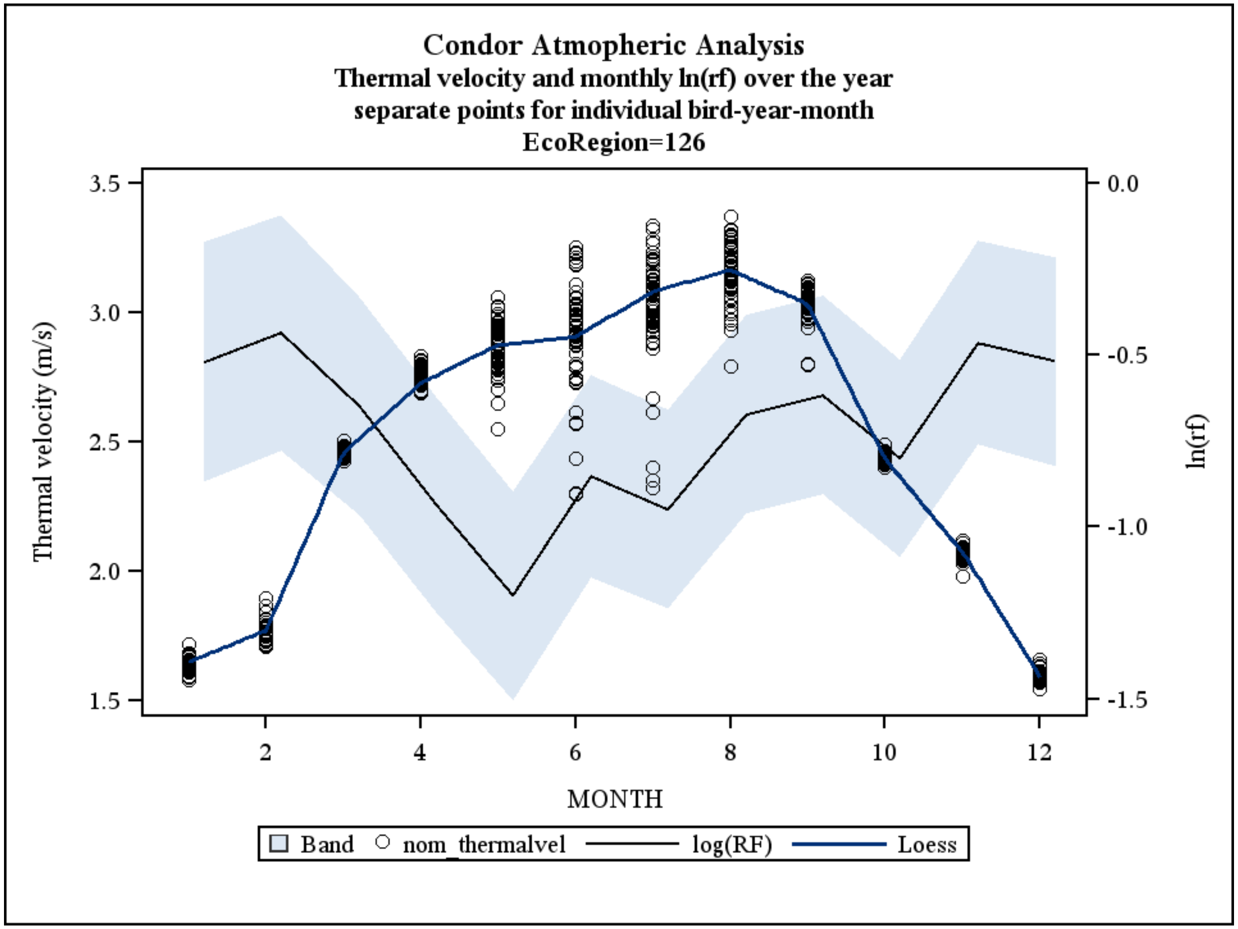

EcoRegion=127

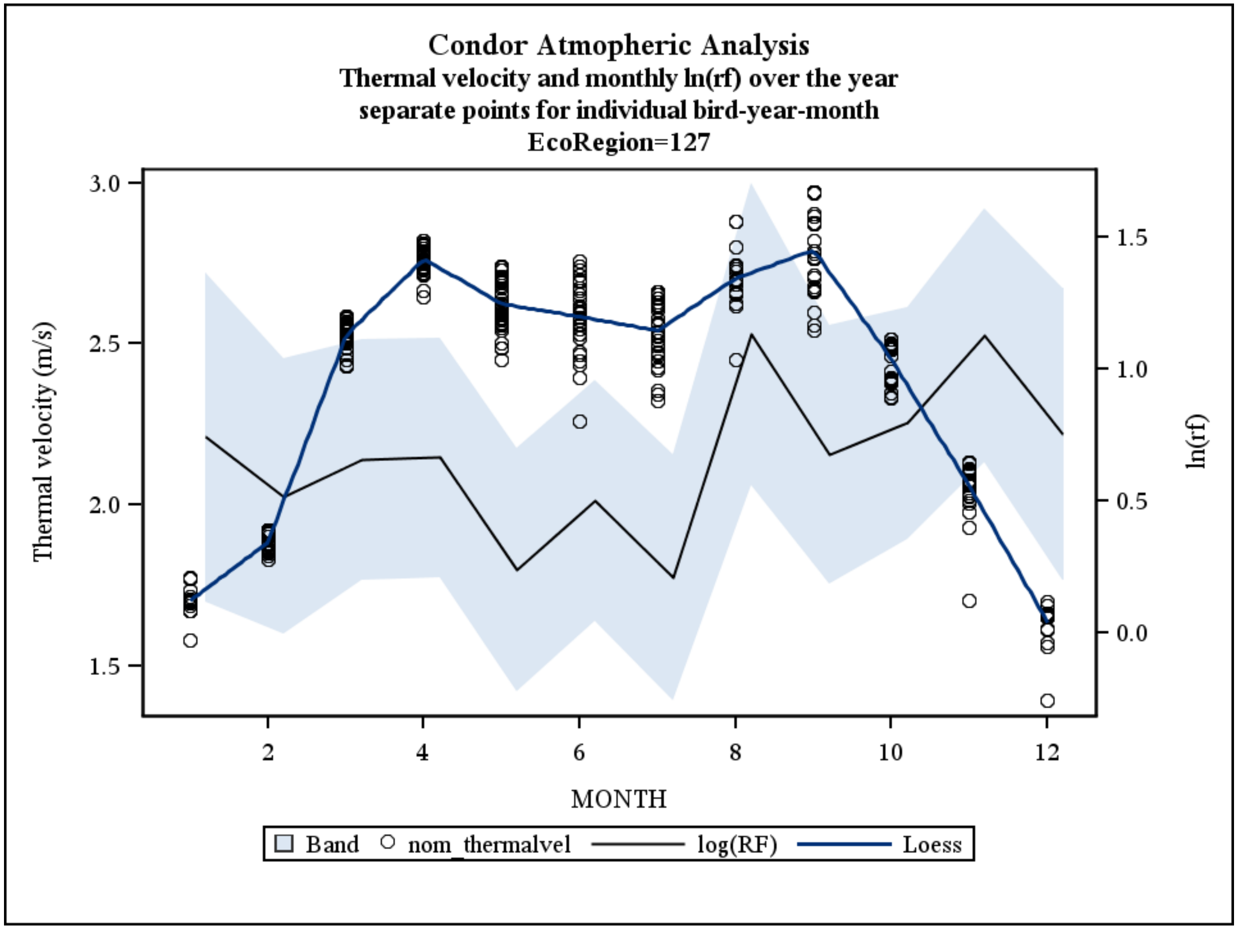

EcoRegion=128

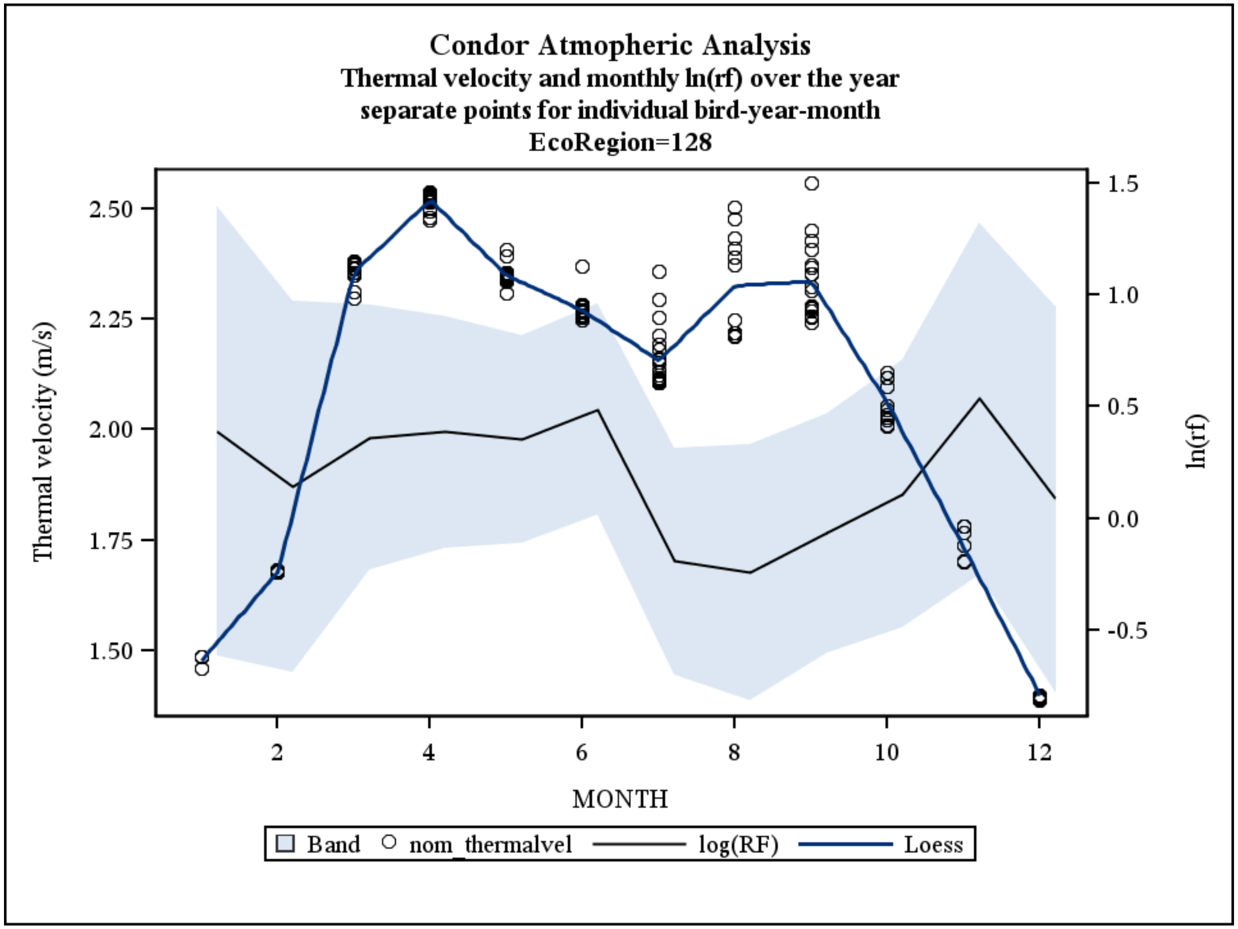

EcoRegion=147

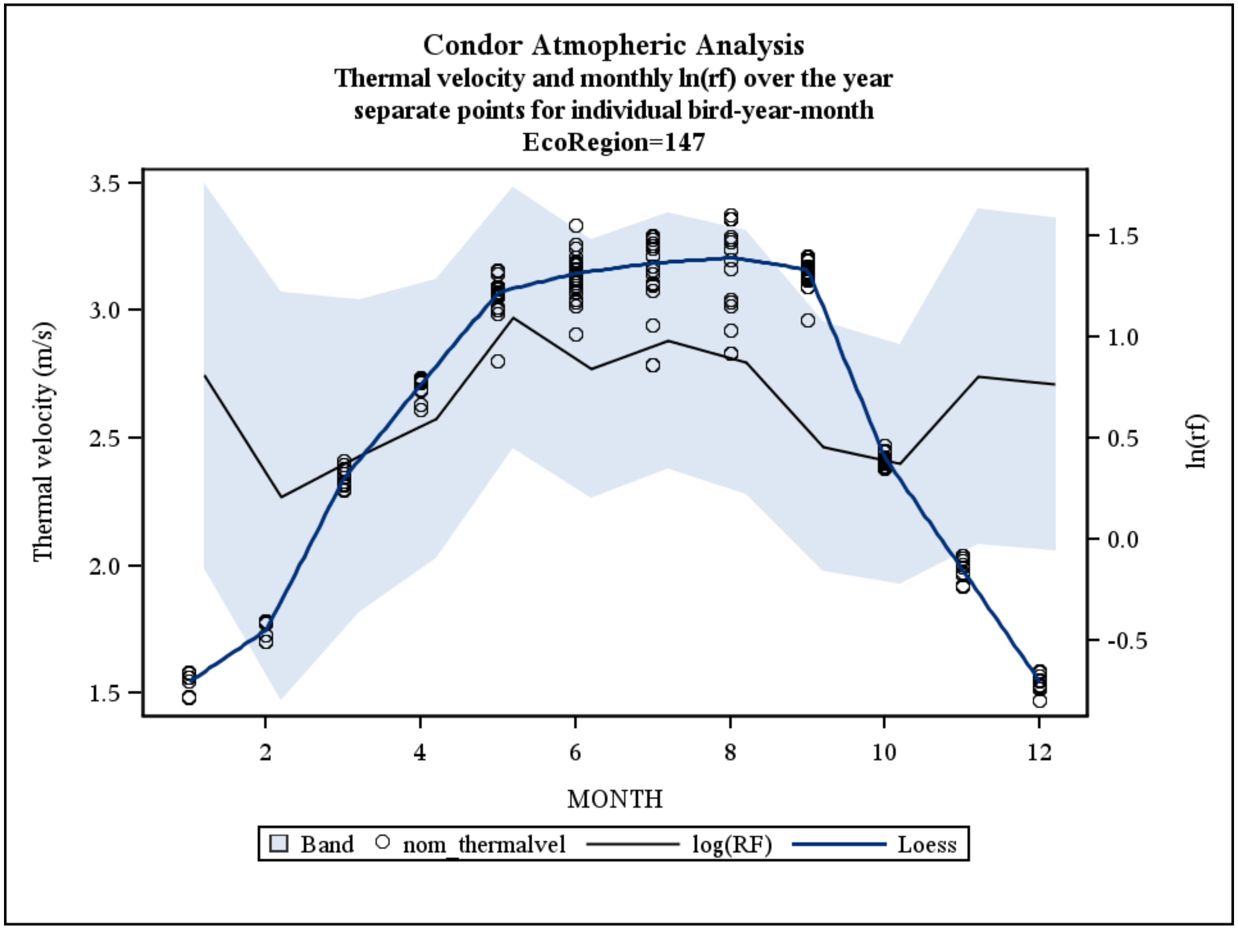

EcoRegion=192

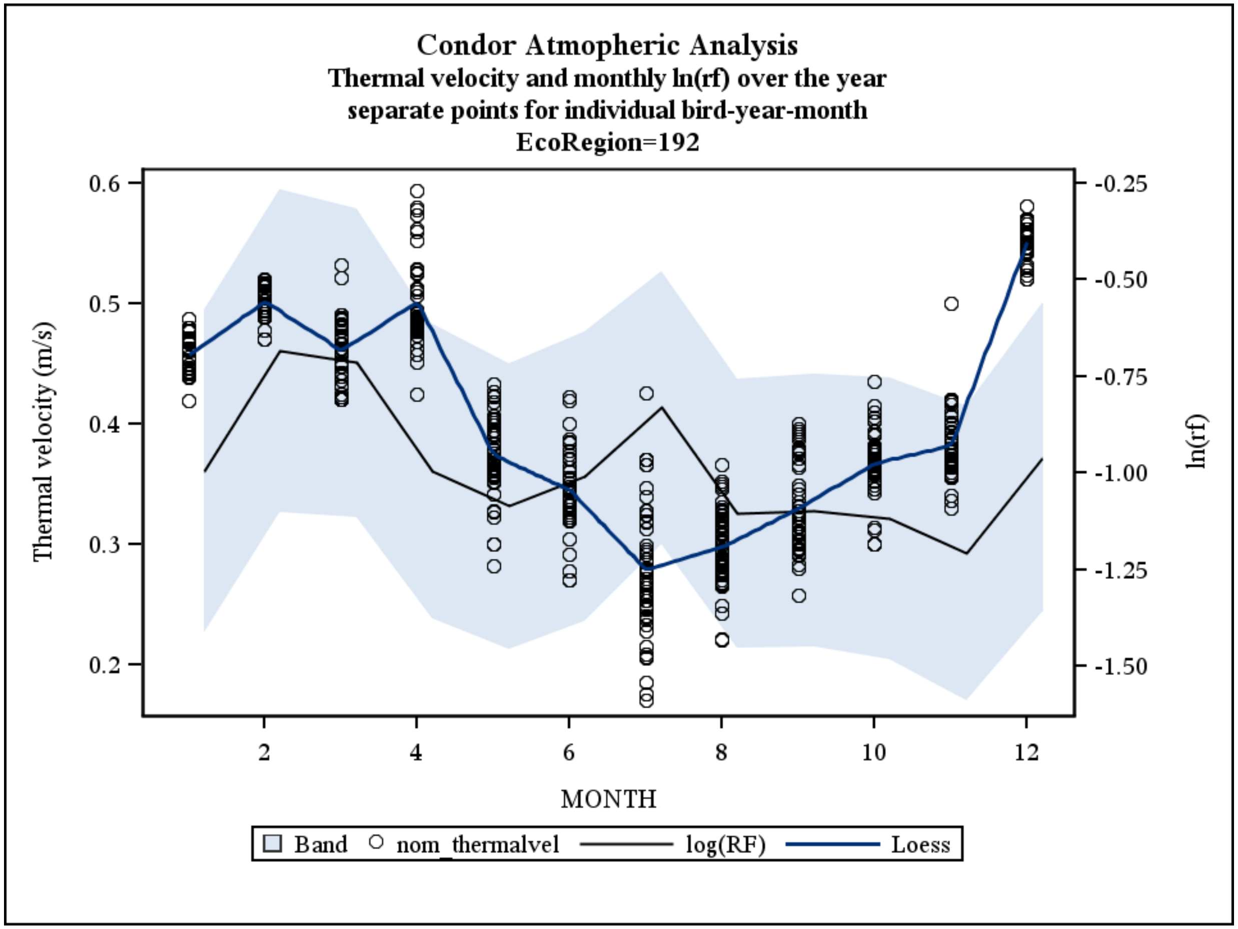

EcoRegion=193

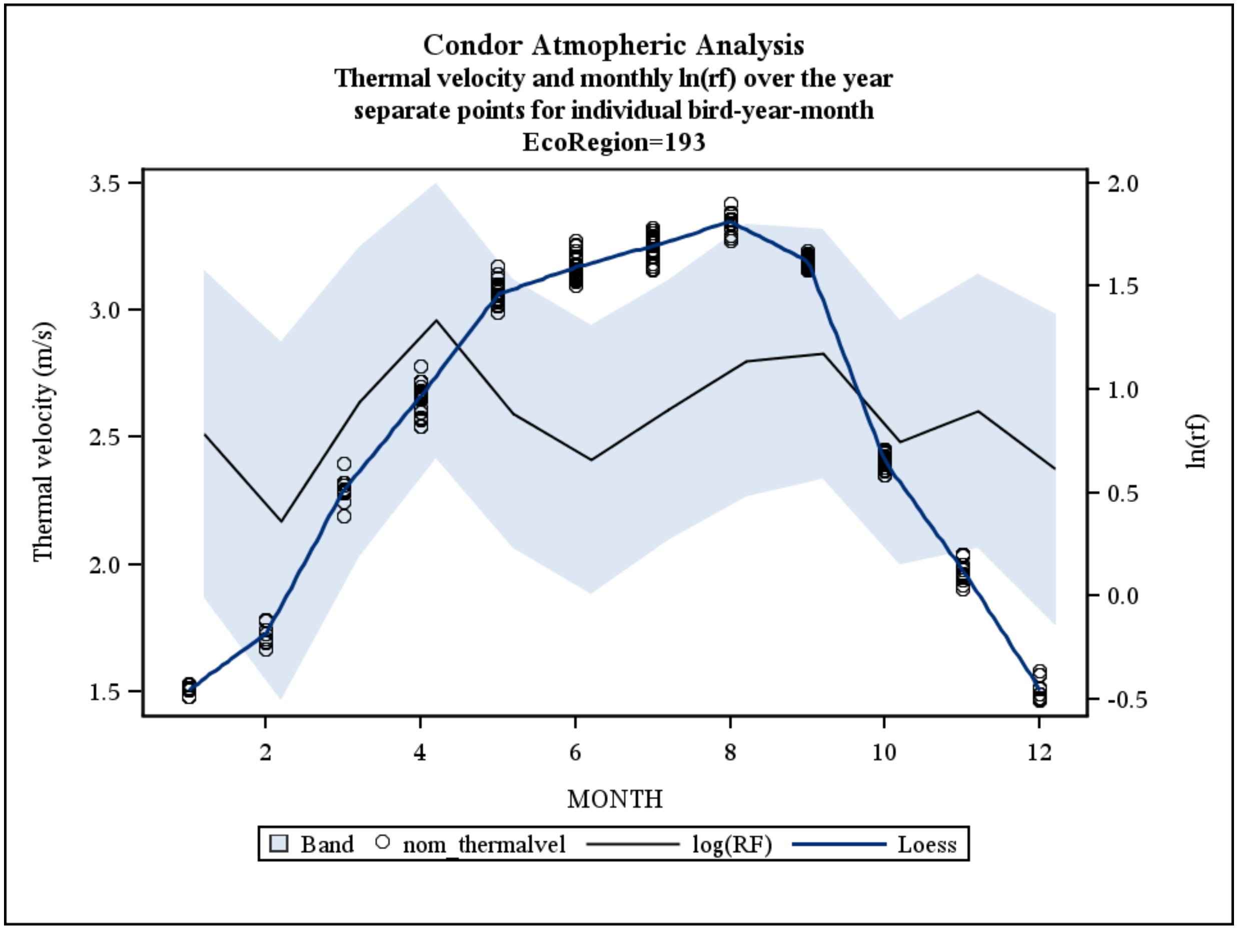

EcoRegion=8

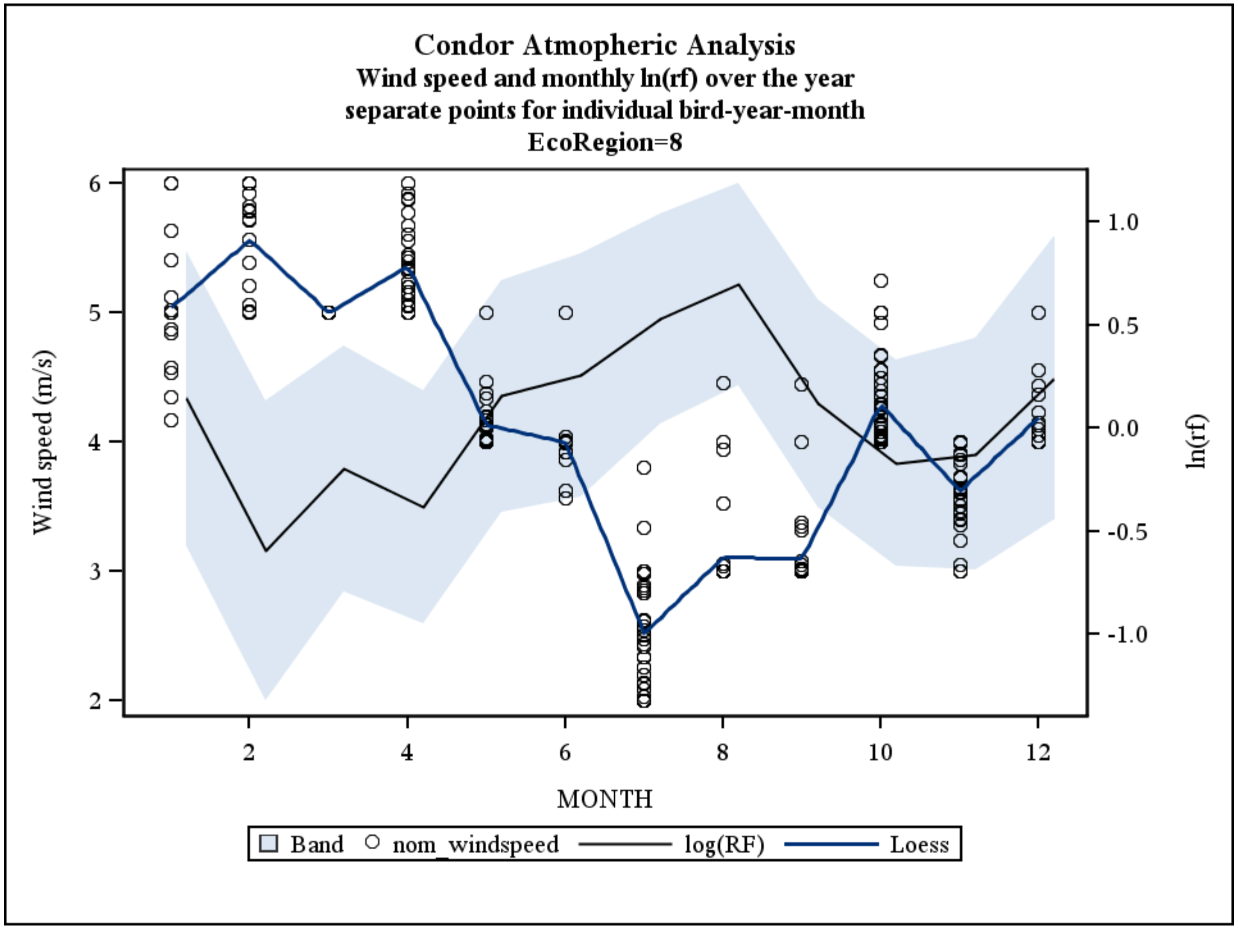
Condor Atmopheric Analysis
Wind speed and monthly ln(rf) over the year
separate points for individual bird-year-month
EcoRegion=8
Wind speed (m/s)
ln(rf)
MONTH
6
5
4
3
2
1.0
0.5
0.0
-0.5
-1.0
2
4
6
8
10
12
Band
nom_windspeed
log(RF)
Loess

EcoRegion=9

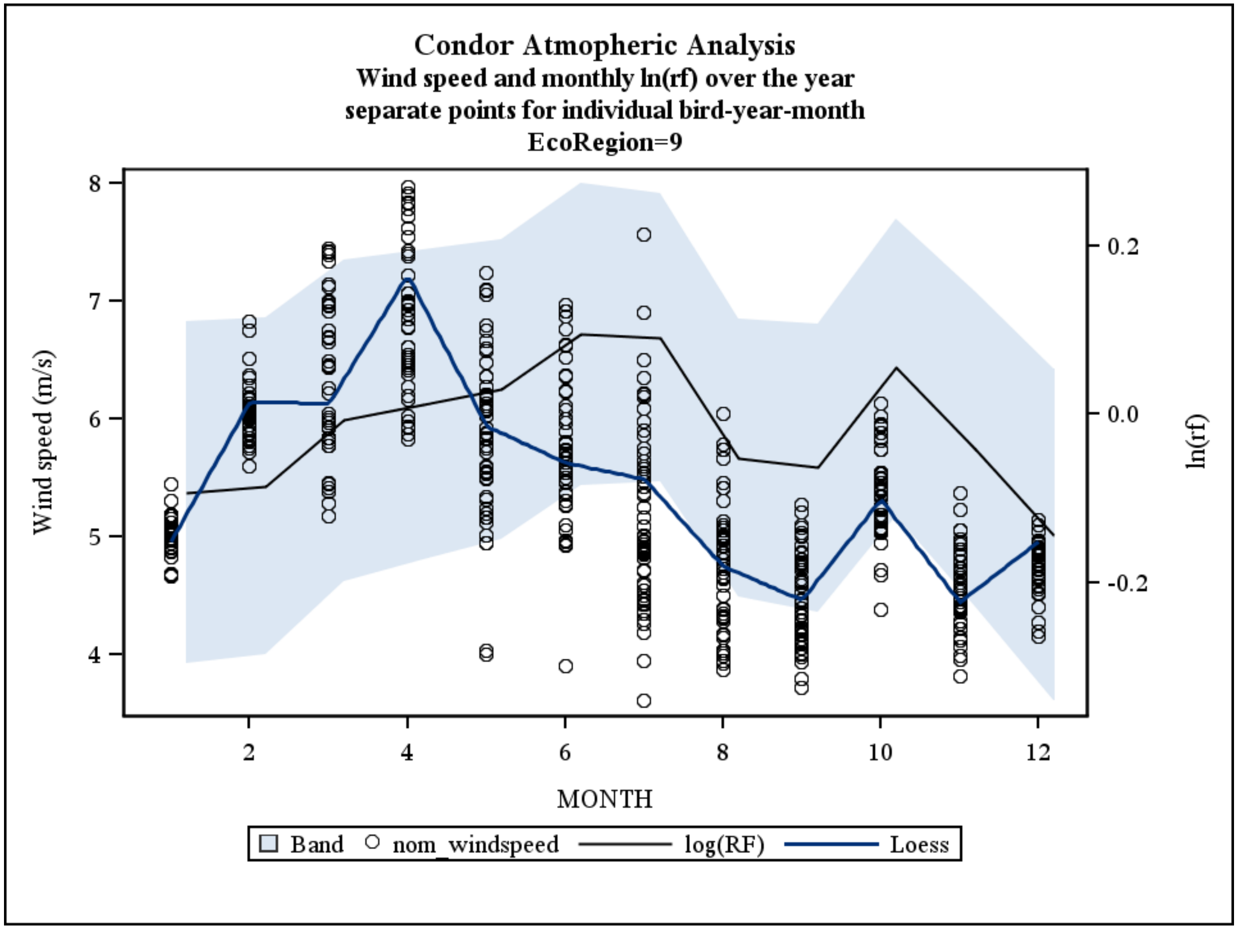
Condor Atmopheric Analysis
Wind speed and monthly ln(rf) over the year
separate points for individual bird-year-month
EcoRegion=9
Wind speed (m/s)
8
7
6
5
4
0.2
0.0
-0.2
ln(rf)
2
4
6
8
10
12
MONTH
Band
nom_windspeed
log(RF)
Loess

EcoRegion=10

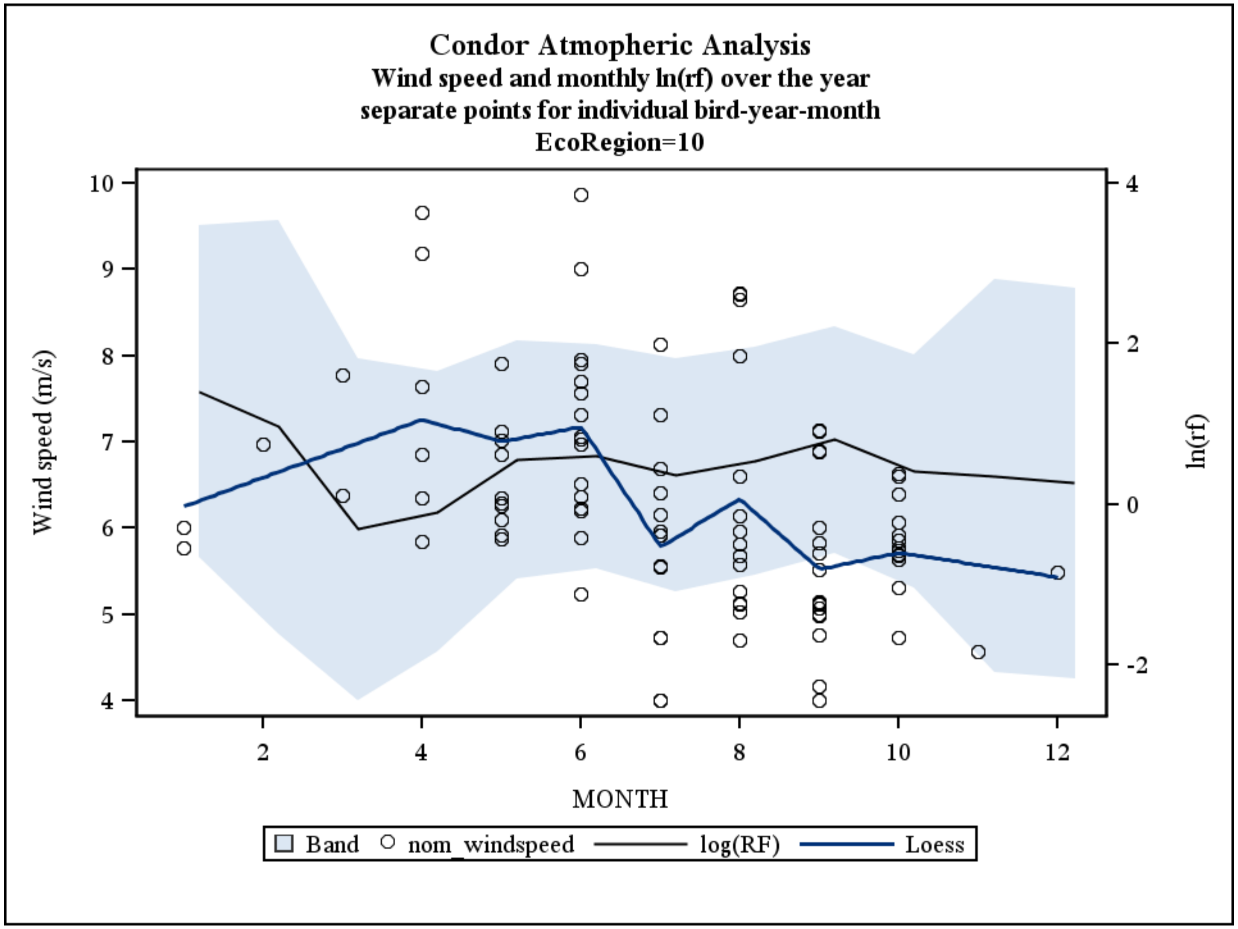

EcoRegion=13

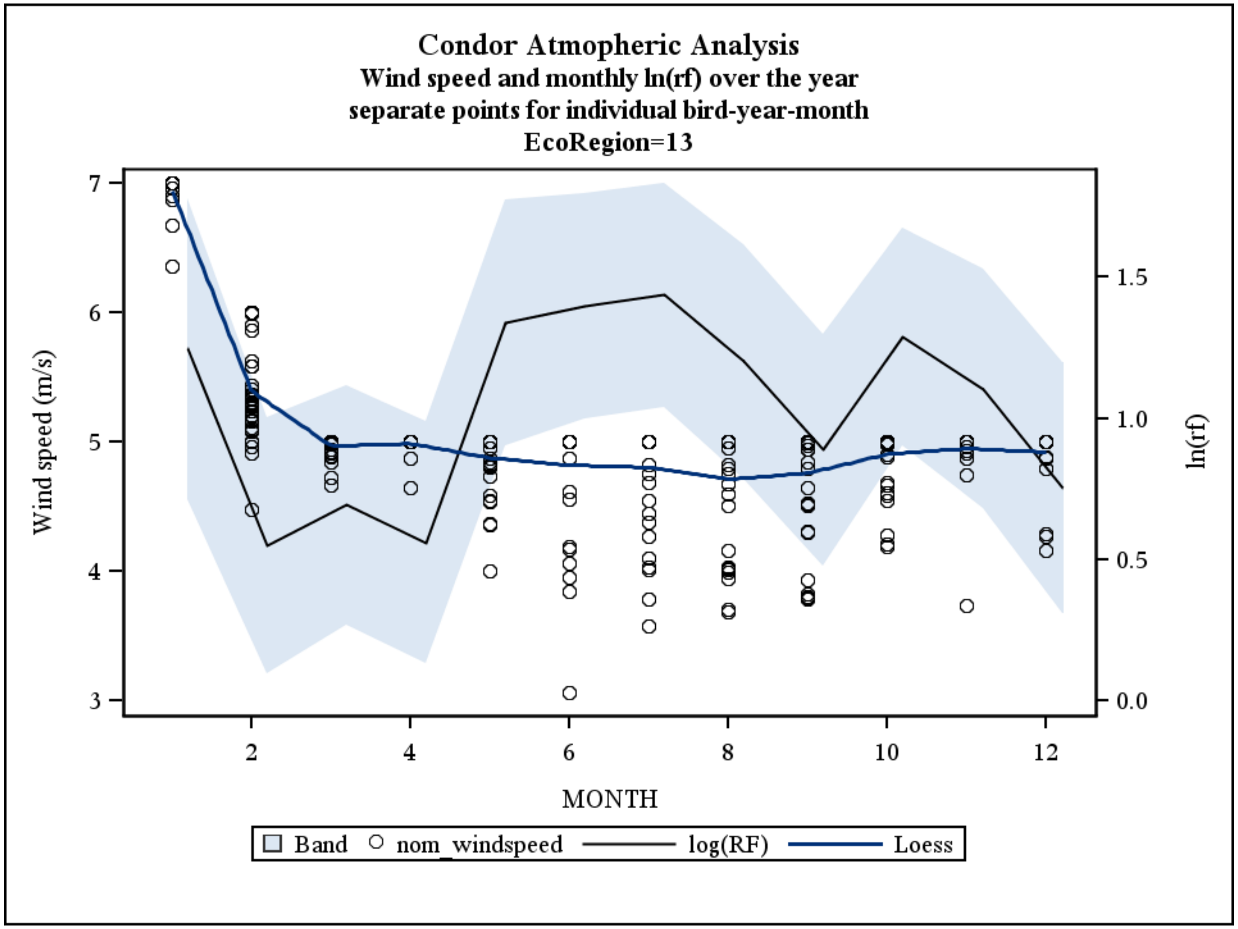

EcoRegion=15

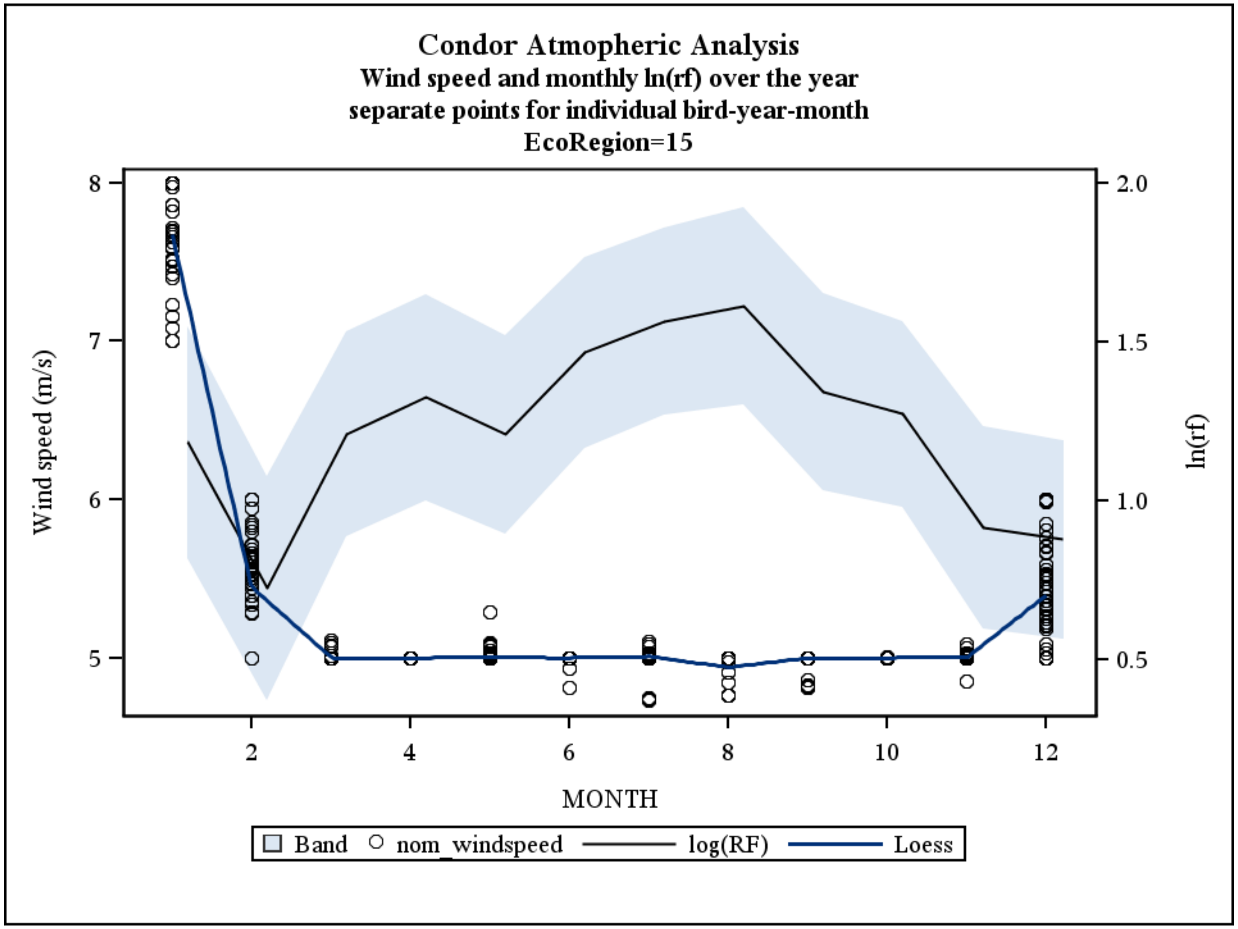

EcoRegion=16

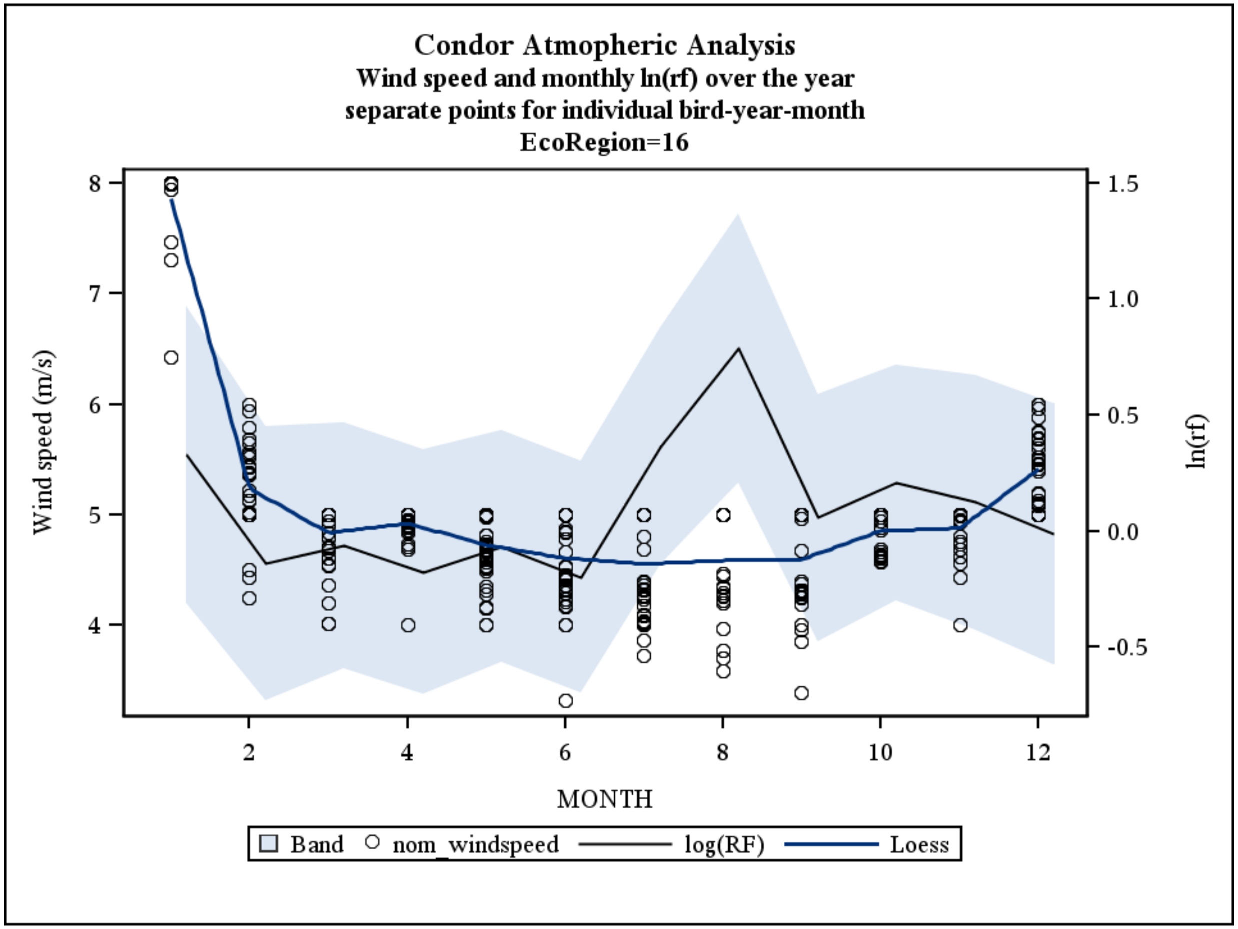

EcoRegion=18

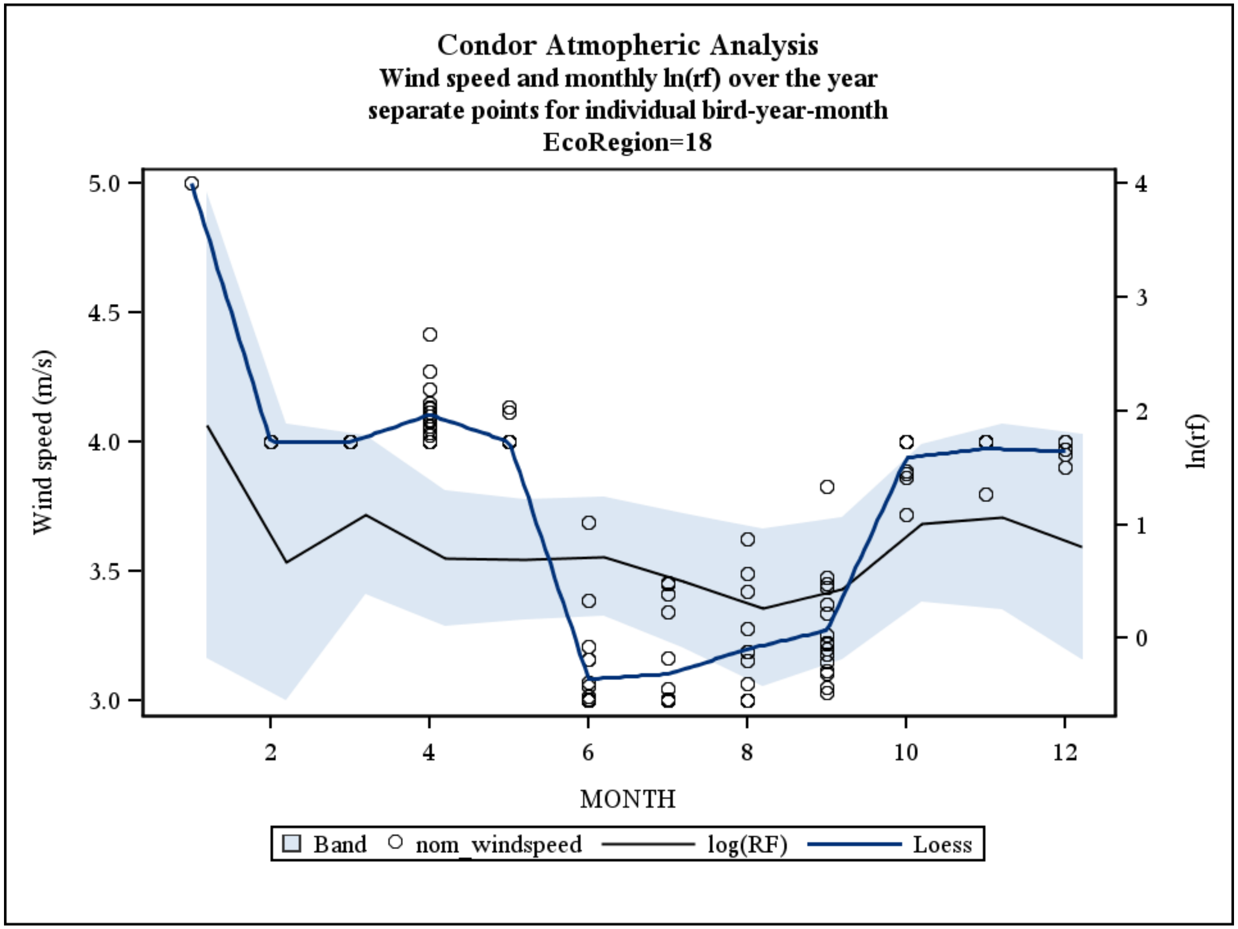

EcoRegion=39

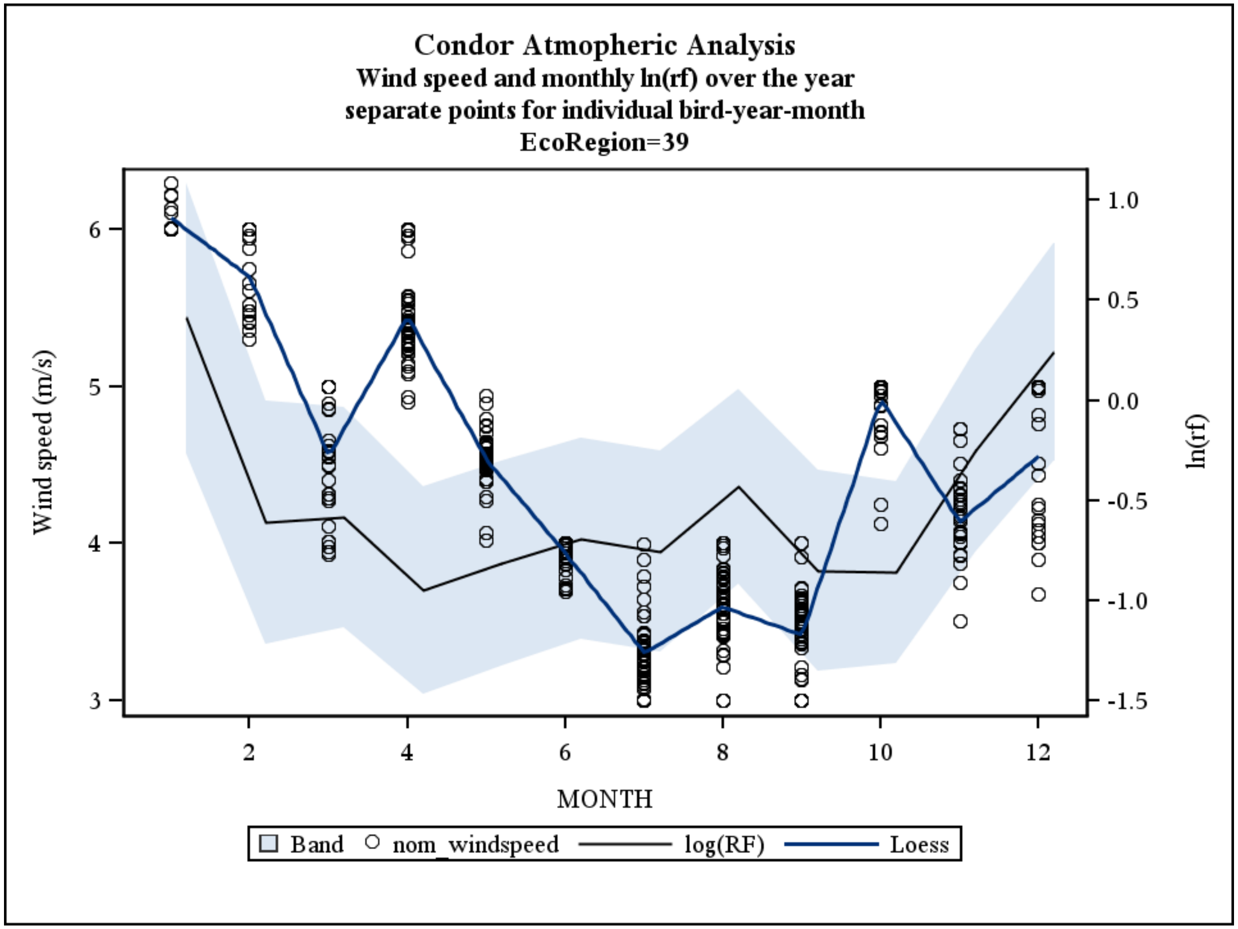

EcoRegion=40

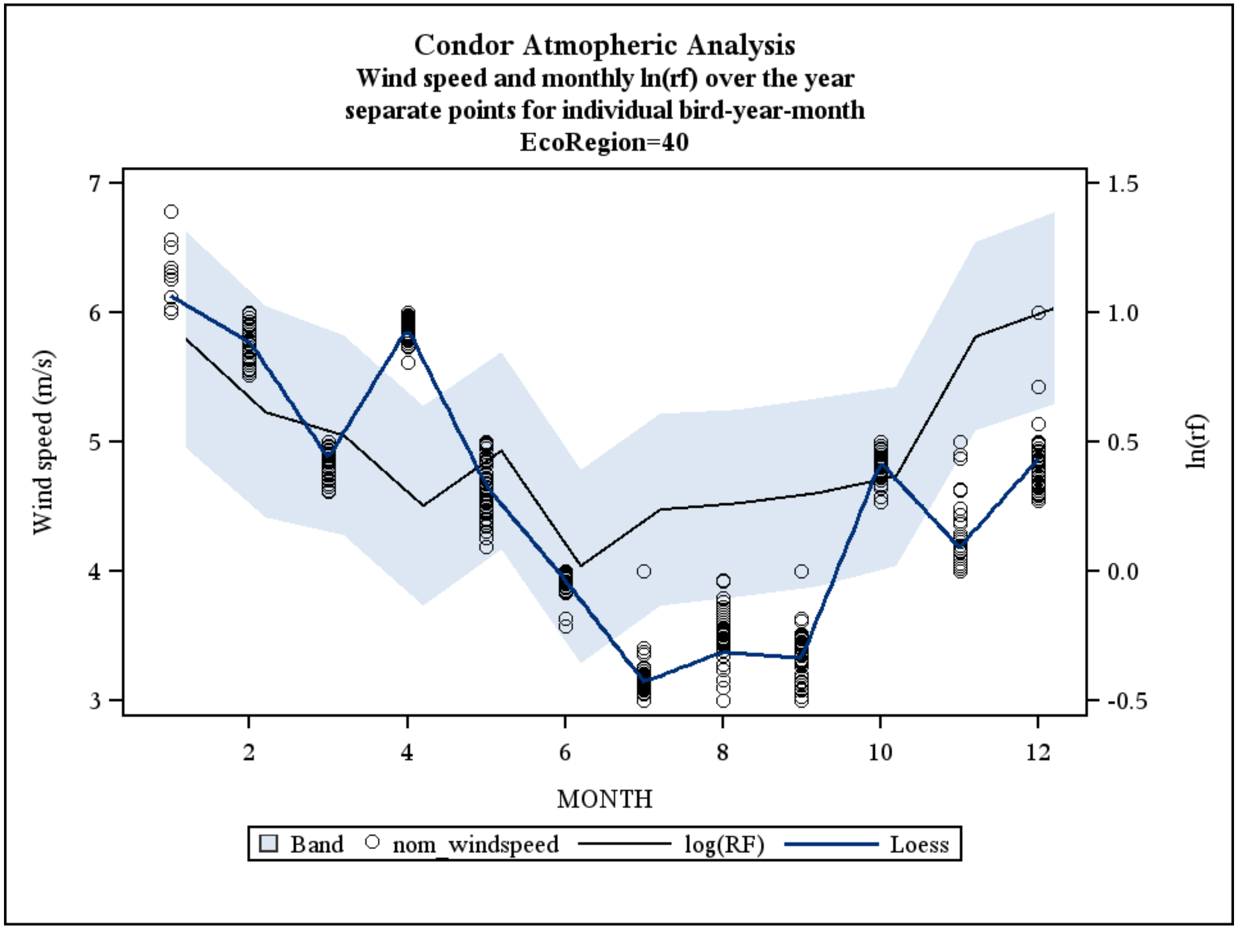

EcoRegion=95

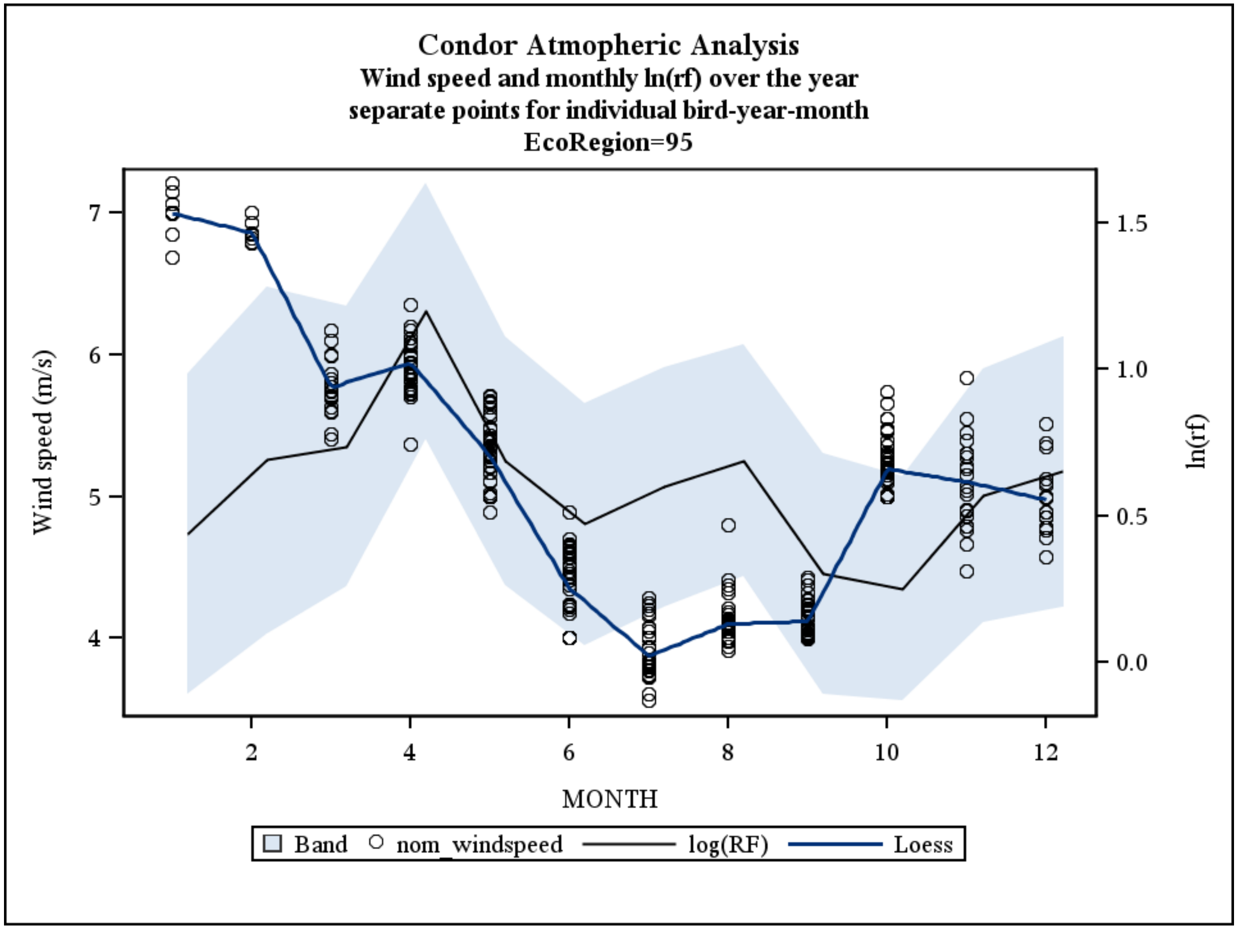

EcoRegion=101

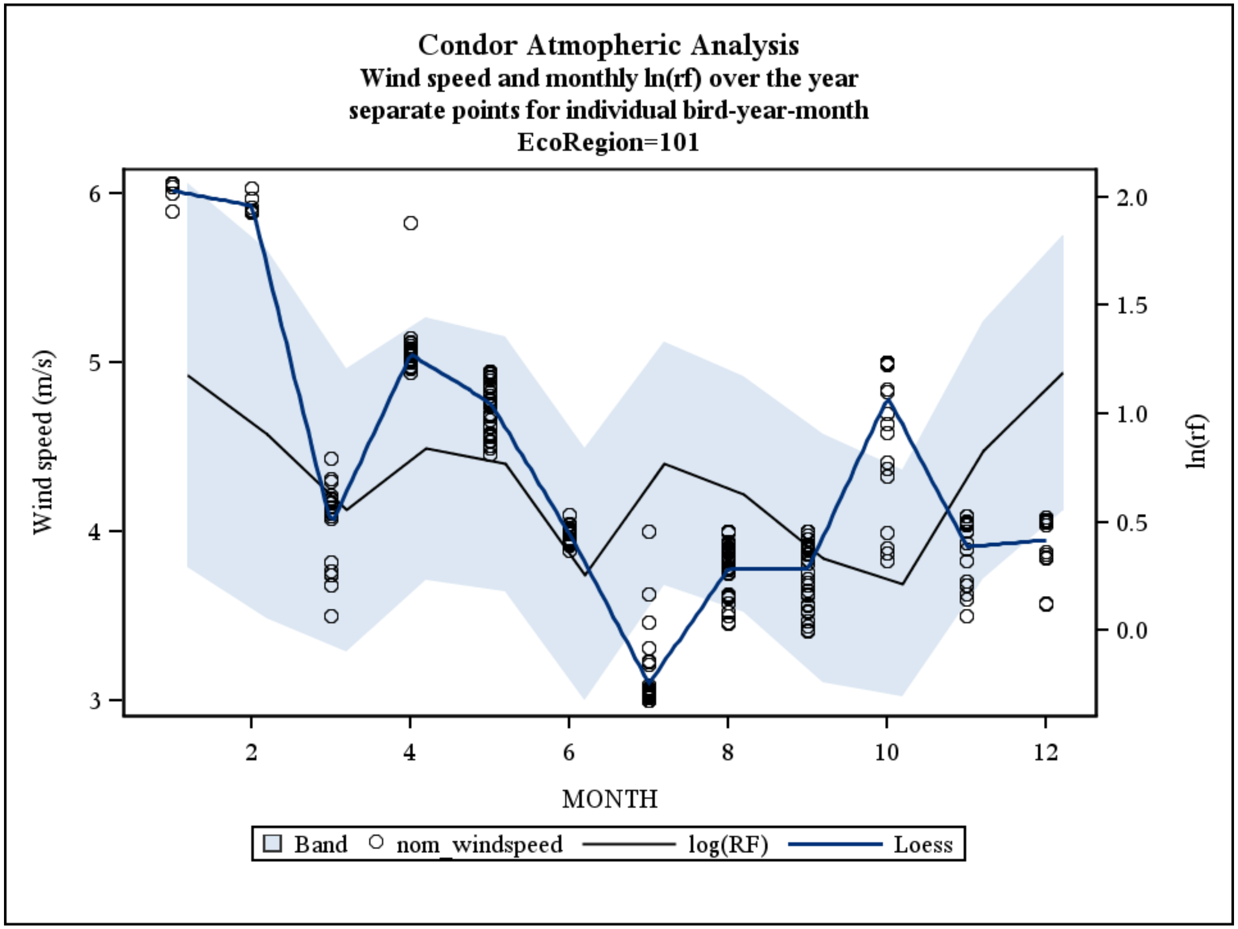

EcoRegion=102

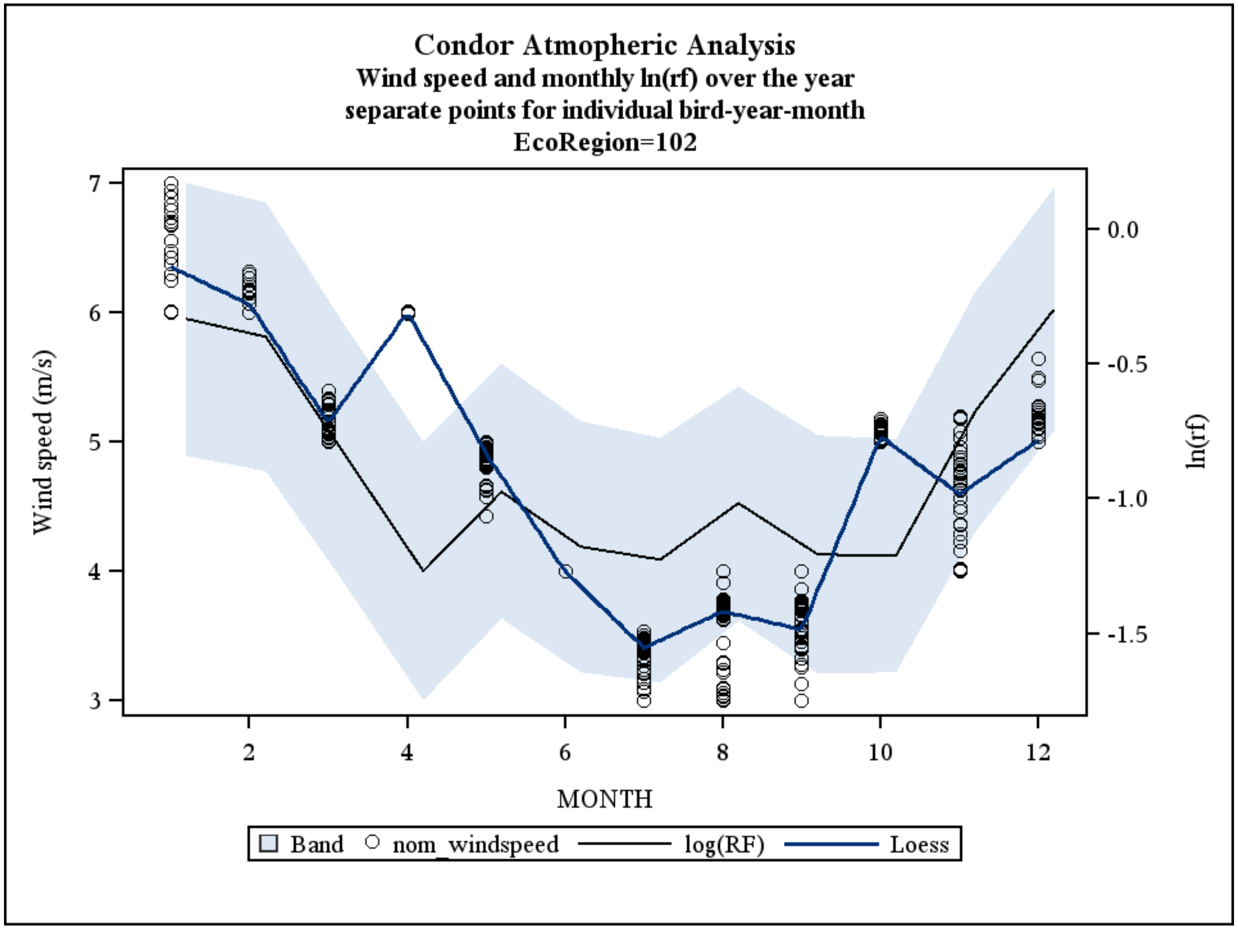

EcoRegion=116

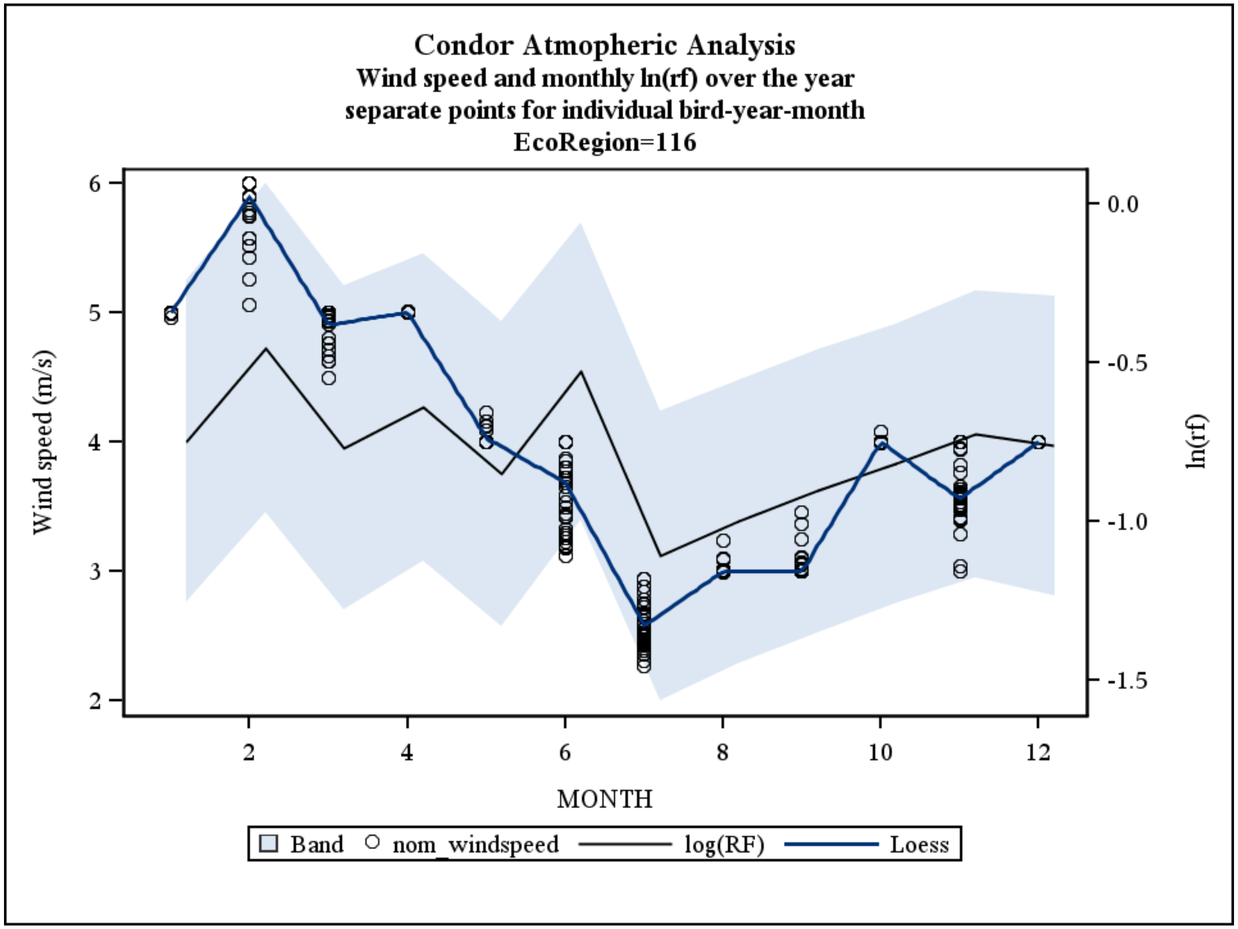

EcoRegion=117

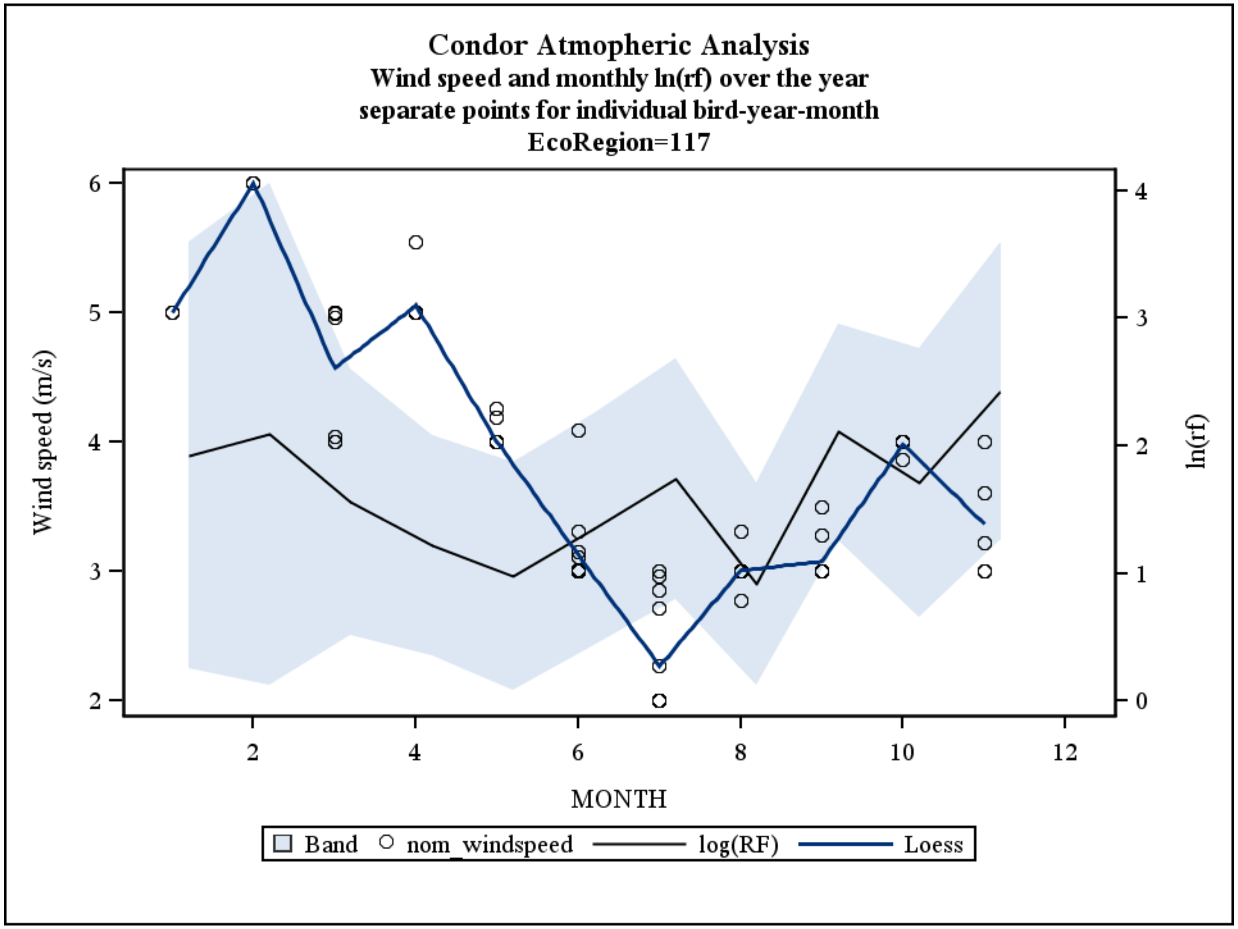

EcoRegion=118

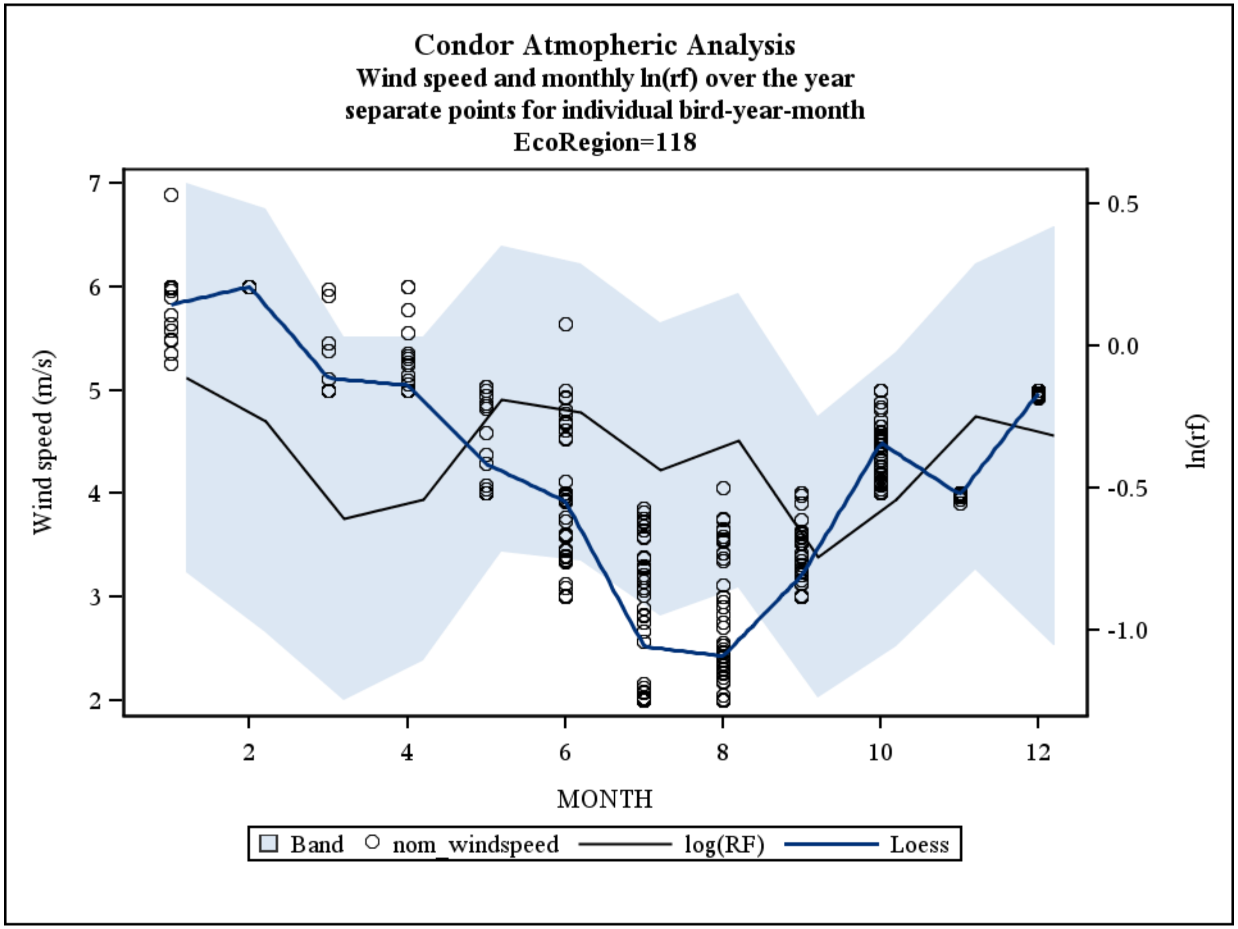

EcoRegion=119

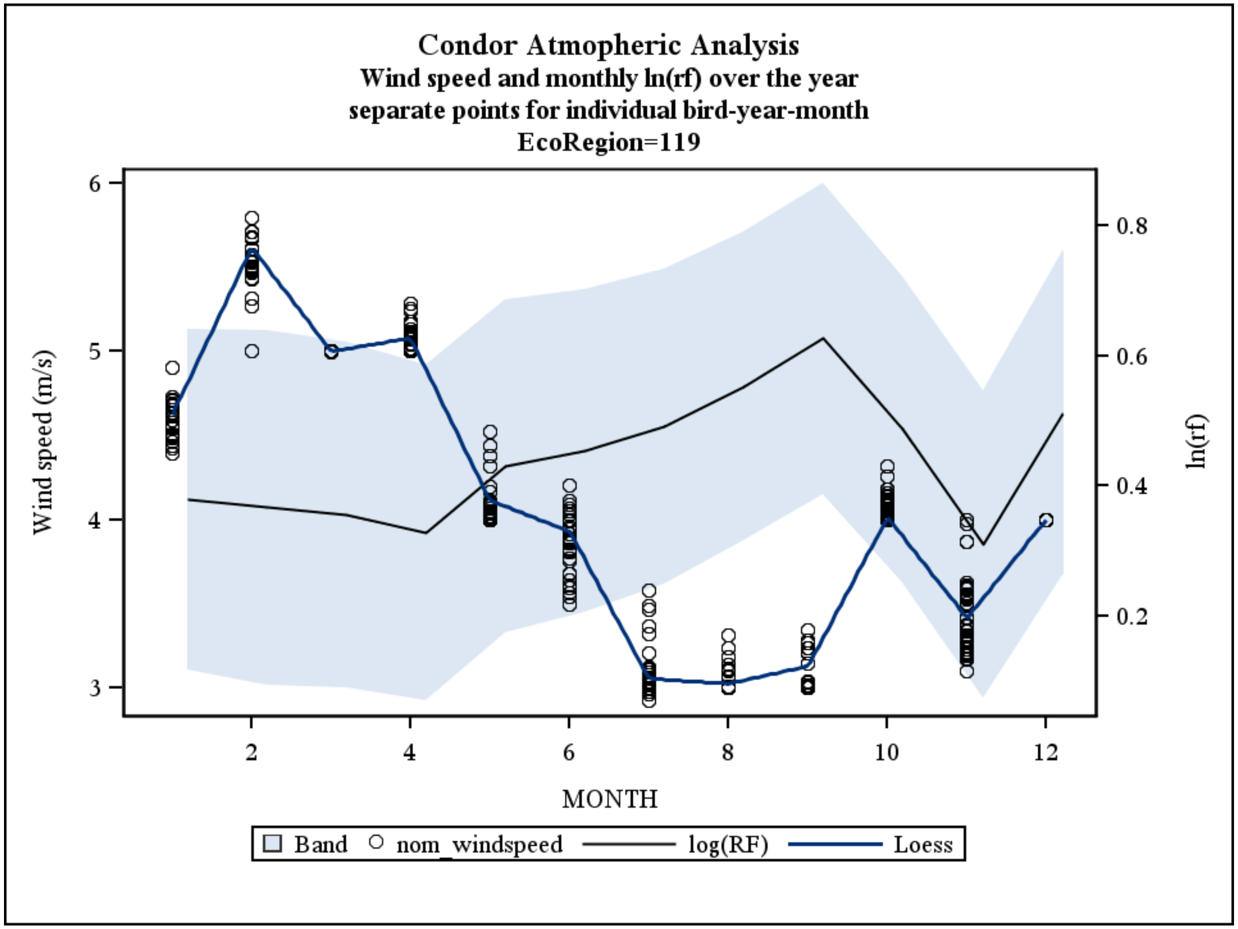

EcoRegion=123

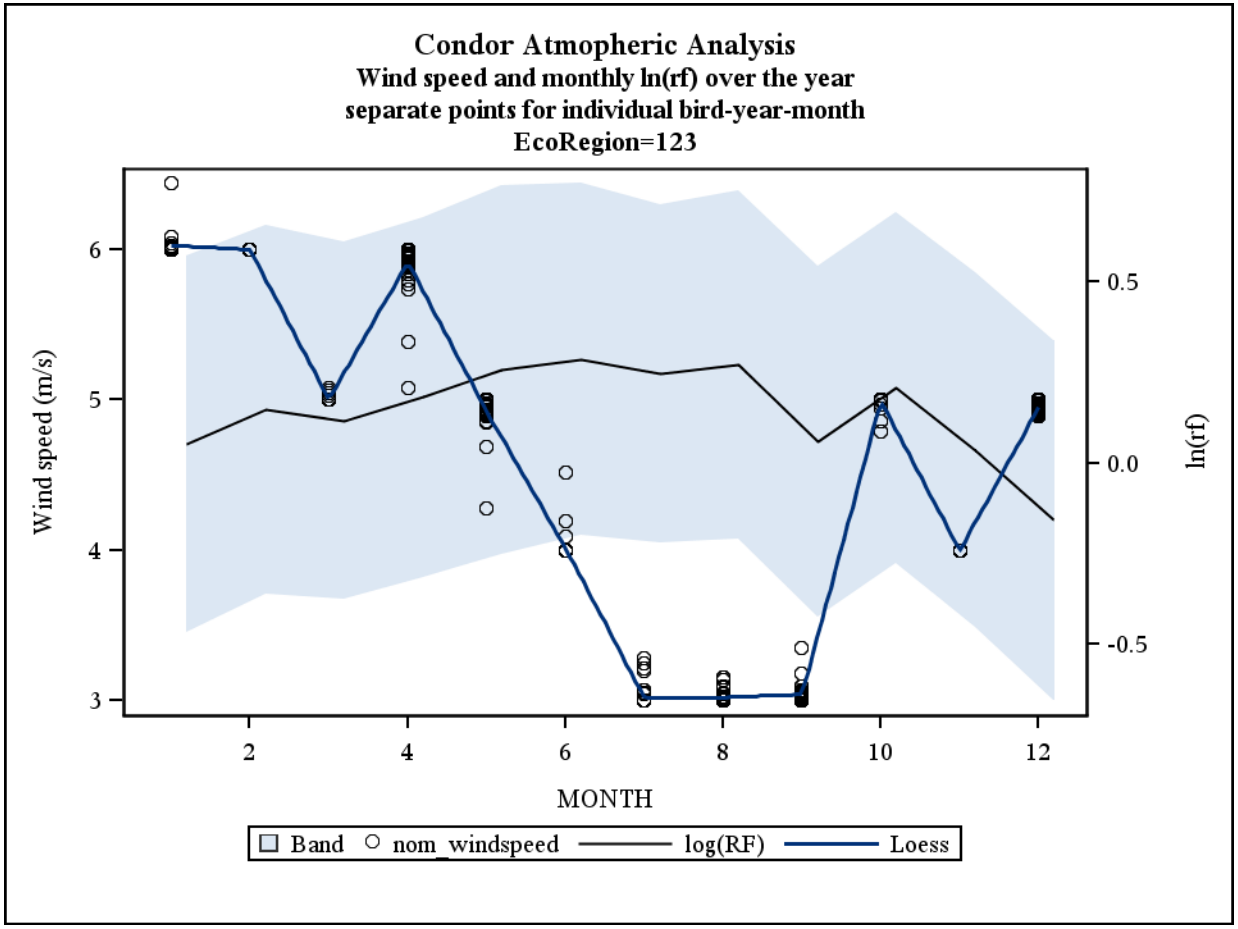

EcoRegion=124

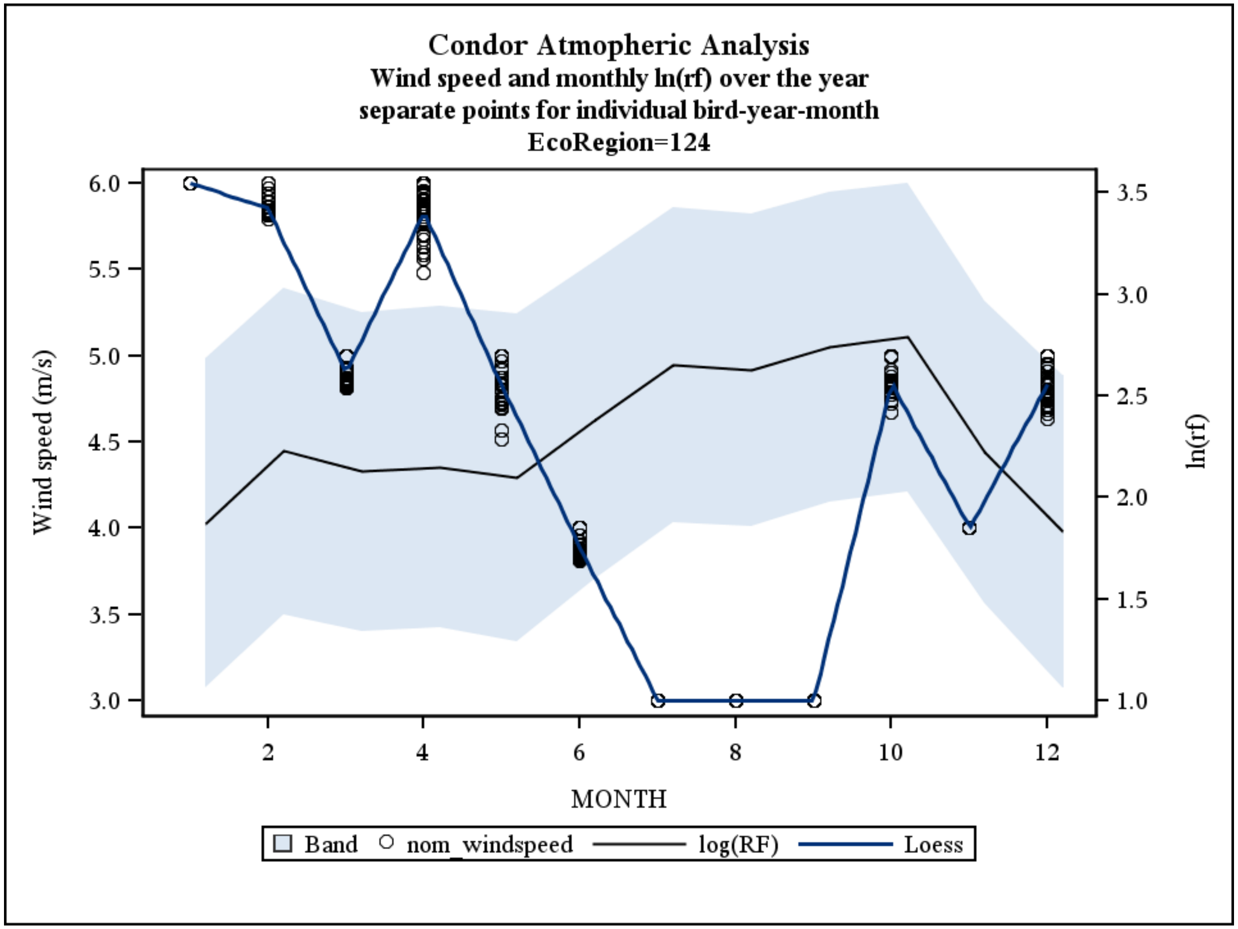

EcoRegion=125

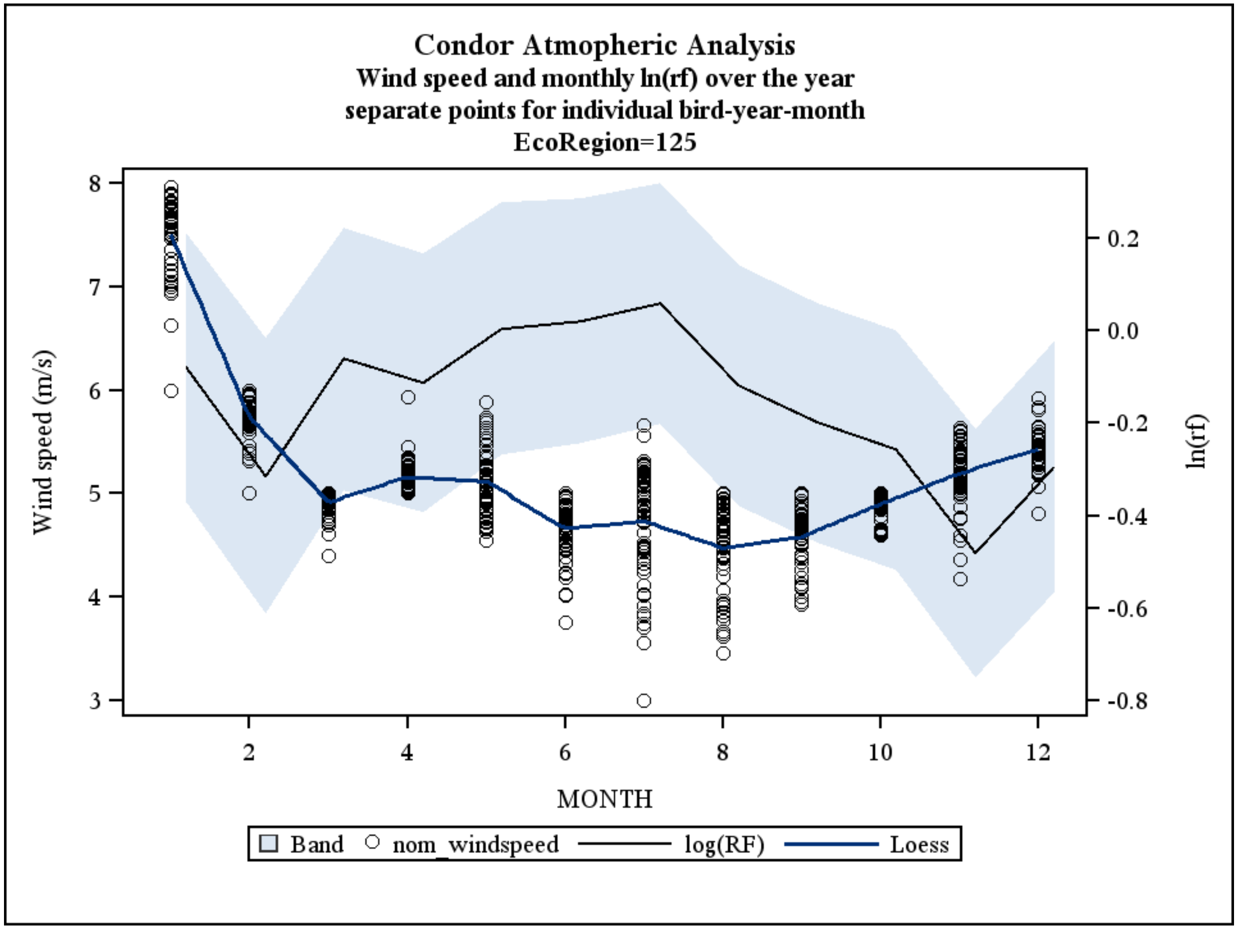

EcoRegion=126

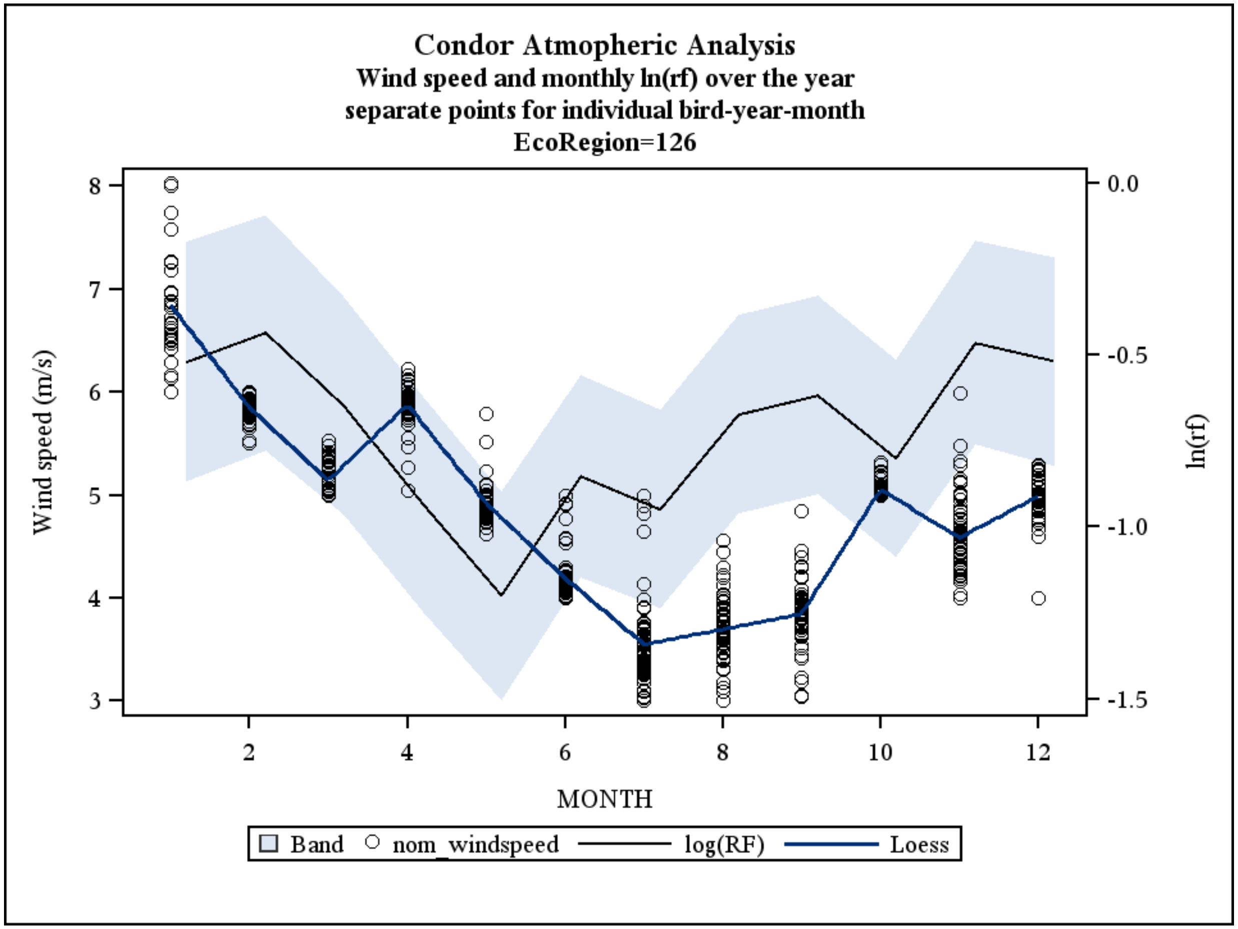

EcoRegion=127

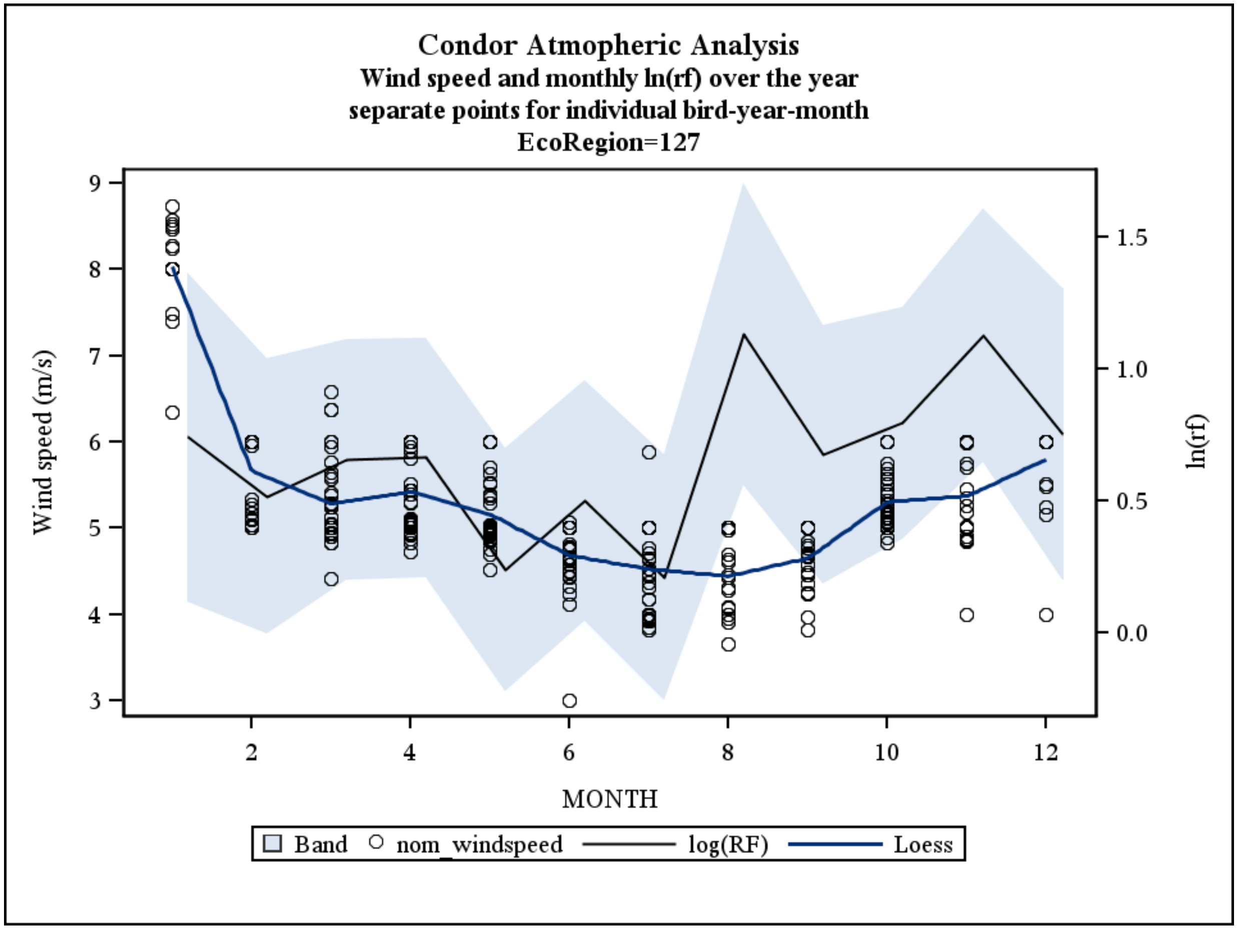

EcoRegion=128

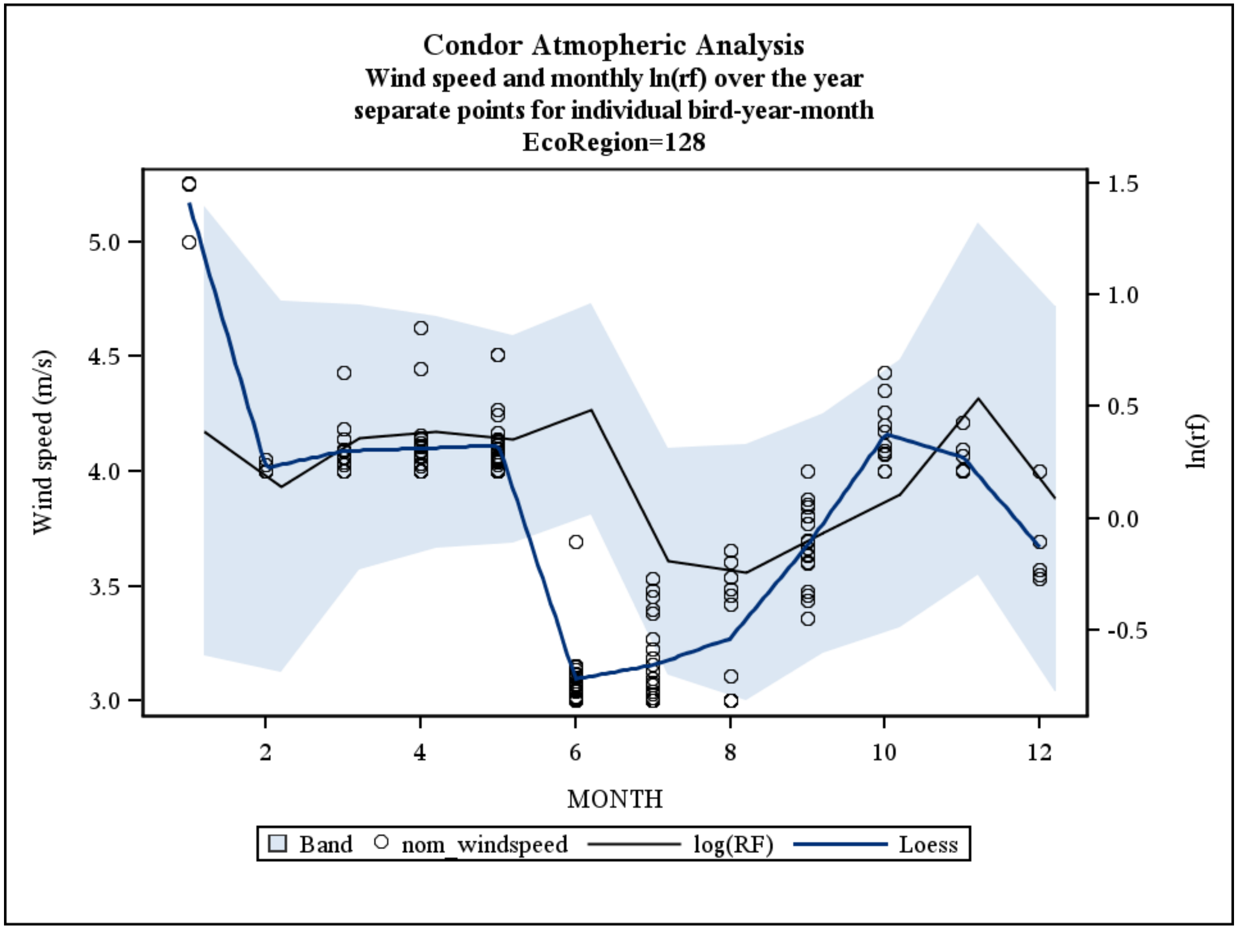

EcoRegion=147

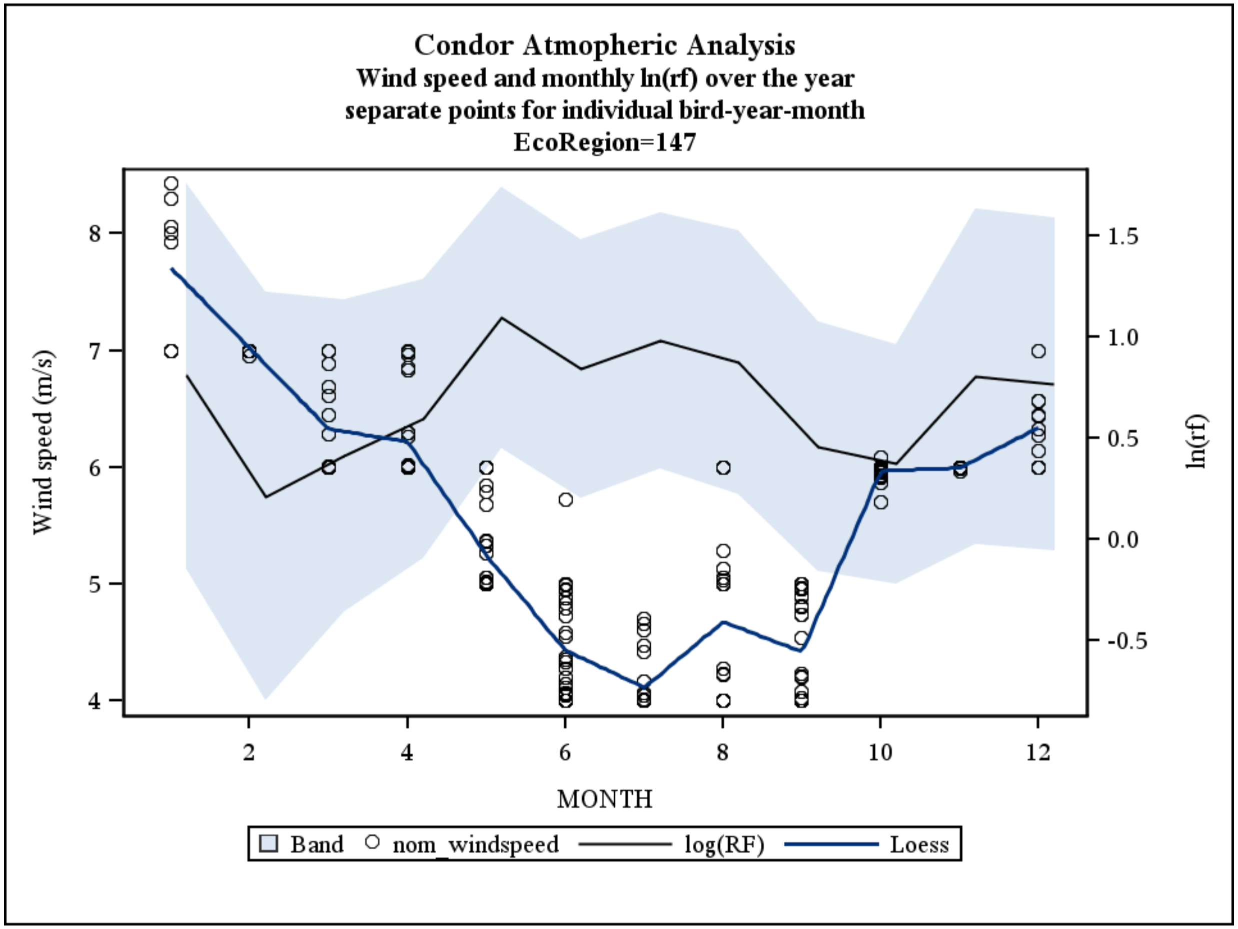

EcoRegion=192

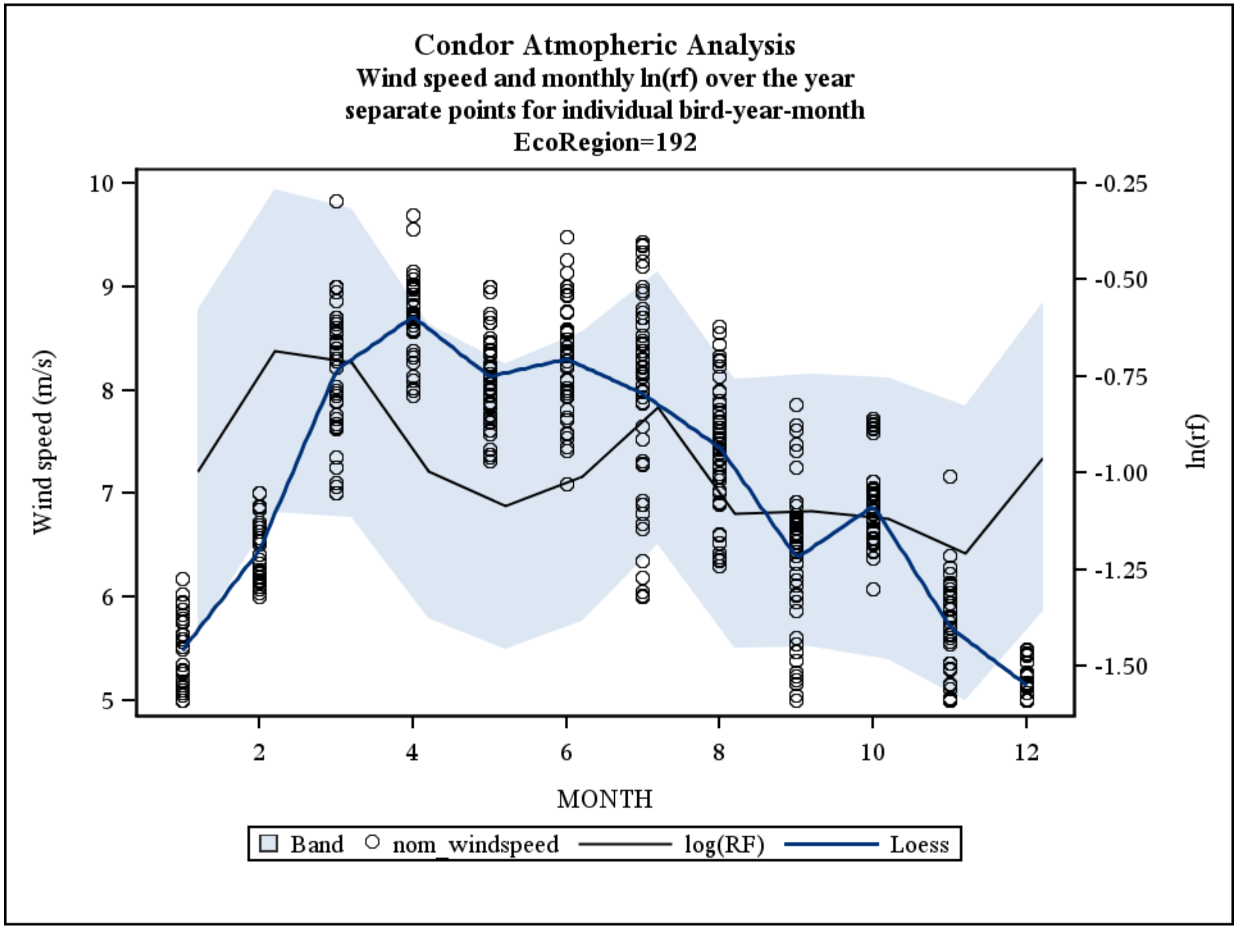

EcoRegion=193

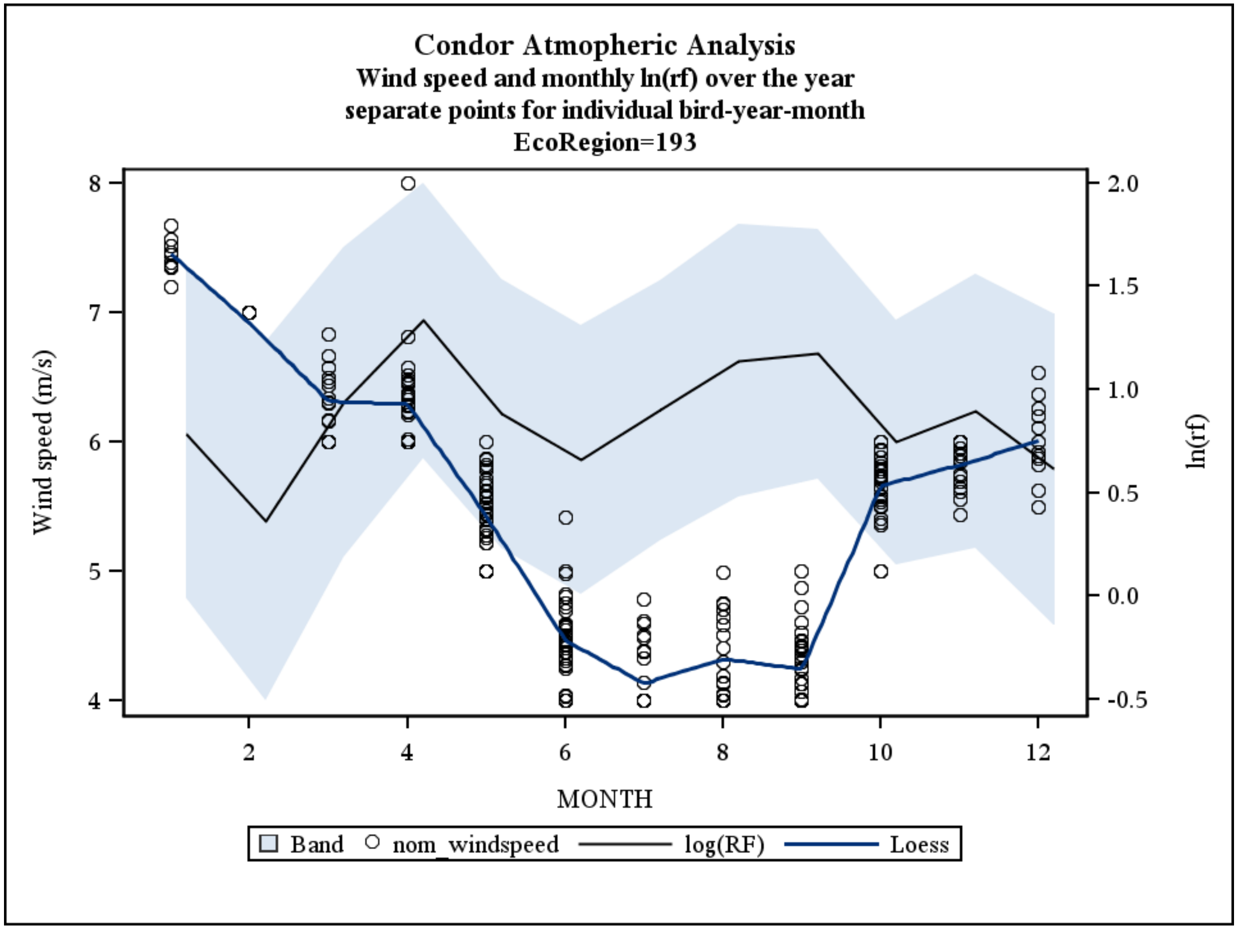

Supplement: Document S7 — Plots for three meteorological parameters and mean ln (rf) values plotted against months in the annual cycle for each of the 25 California ecoregions examined in the study. (PDF) [file pone.0088430.s007.pdf]
